# MDCK Cystogenesis Driven by Cell Stabilization within Computational Analogues

**DOI:** 10.1371/journal.pcbi.1002030

**Published:** 2011-04-07

**Authors:** Jesse A. Engelberg, Anirban Datta, Keith E. Mostov, C. Anthony Hunt

**Affiliations:** 1 UCSF/UC Berkeley Joint Graduate Group in Bioengineering, University of California, San Francisco, California, United States of America; 2 Department of Anatomy, University of California, San Francisco, California, United States of America; 3 Department of Bioengineering and Therapeutic Sciences, University of California, San Francisco, California, United States of America; University of California San Diego, United States of America

## Abstract

The study of epithelial morphogenesis is fundamental to increasing our understanding of organ function and disease. Great progress has been made through study of culture systems such as Madin-Darby canine kidney (MDCK) cells, but many aspects of even simple morphogenesis remain unclear. For example, are specific cell actions tightly coupled to the characteristics of the cell's environment or are they more often cell state dependent? How does the single lumen, single cell layer cyst consistently emerge from a variety of cell actions? To improve insight, we instantiated in silico analogues that used hypothesized cell behavior mechanisms to mimic MDCK cystogenesis. We tested them through in vitro experimentation and quantitative validation. We observed novel growth patterns, including a cell behavior shift that began around day five of growth. We created agent-oriented analogues that used the cellular Potts model along with an Iterative Refinement protocol. Following several refinements, we achieved a degree of validation for two separate mechanisms. Both survived falsification and achieved prespecified measures of similarity to cell culture properties. In silico components and mechanisms mapped to in vitro counterparts. In silico, the axis of cell division significantly affects lumen number without changing cell number or cyst size. Reducing the amount of in silico luminal cell death had limited effect on cystogenesis. Simulations provide an observable theory for cystogenesis based on hypothesized, cell-level operating principles.

## Author Summary

Epithelial cells perform essential functions throughout the body, acting as both barrier and transporter and allowing an organism to survive and thrive in varied environments. Although the details of many processes that occur within individual cells are well understood, we still lack a thorough understanding of how cells coordinate their behaviors to create complex tissues. In order to achieve deeper insight, we created a list of targeted attributes and plausible rules for the growth of multicellular cysts formed by Madin-Darby canine kidney (MDCK) cells grown in vitro. We then designed in silico analogues of MDCK cystogenesis using object-oriented programming. In silico components (such as the cells and lumens) and their behaviors directly mapped to in vitro components and mechanisms. We conducted in vitro experiments to generate data that would validate or falsify the in silico analogues and then iteratively refined the analogues to mimic that data. Cells in vitro begin to stabilize at around the fifth day even as cysts continue to expand. The in silico system mirrored that behavior and others, achieving new insights. For example, luminal cell death is not strictly required for cystogenesis, and cell division orientation is very important for normal cyst growth.

## Introduction

Epithelial morphogenesis is fundamental to the development and functional specialization of tissues and organs. Tight regulation of tissue size, shape and polarization is critical for normal organ development and function. Disruption of these regulatory mechanisms leads to an array of diseases including autosomal dominant polycystic kidney disease, stenosis, and cancer. Epithelial cells, such as Madin-Darby canine kidney (MDCK) cells, cultured in a 3D matrix of natural basement membrane components, can recapitulate in vitro many of the in vivo growth characteristics of epithelial organs. They are thus valuable model systems for studying the cellular mechanisms of in vivo epithelial morphogenesis. Their phenotypic simplicity coupled with accumulated knowledge of their molecular biology provide excellent case studies for gleaning needed insight into how molecular events and environmental feedback pathways at subcellular levels lead to cell- and cyst-level phenotype. These model systems lend themselves to computational analysis and modeling as the means to gain that insight and improve our understanding of organogenesis.

To achieve that goal, we must first develop explanatory and easily challenged computational, mechanistic models. In biological research, explanatory mechanistic models generally precede predictive mechanistic models. The operating principles of explanatory mechanistic models of the type described herein are hypotheses about how we think phenomena are generated. The models are part of frameworks for generating and testing mechanistic hypotheses, as described in [1], [2].

While many aspects of MDCK cyst formation are well understood, quantitative data for cystogenesis has been lacking. The most recent computational models [1]-[4] relied on previously published quantitative data that described a few aspects of MDCK cyst growth in collagen cultures [5]. There is limited data available on the dynamics of cell number, cyst and lumen size, and mean cell size in Matrigel cultures. That caused previous models to assume that cell size remains constant. The presented data demonstrate that cell size varies during the course of cyst growth.

An objective of the project was to couple in vitro and in silico model systems to achieve a deeper understanding of cell behavior during MDCK cystogenesis within 3D Matrigel cultures. Of specific interest were the roles played by, and the timing of polarization, apoptosis, and lumen expansion. In order to improve our understanding of the link between individual cell behavior and cystogenesis, we proceeded in parallel on two fronts. We undertook new in vitro experiments designed to provide a more temporally and spatially fine-grained record of cell-level events during the first ten days of MDCK cystogenesis. These experiments and their results are described in this report. A thorough quantitative analysis of these results revealed a third stage of cyst growth after cyst initiation and lumen creation and expansion. That stage was characterized by the presence of a new cell state marked by a decrease in cell division rate and cessation of the decrease in cell size observed in previous stages. We refer to a cell in that state as being “stabilized”.

We also developed and iteratively refined abstract, spatially fine-grained, multi-attribute, mechanistic, in silico, MDCK cell analogues (ISMAs) capable of cystogenesis. To create and validate ISMAs, we merged two modeling techniques while introducing several novel features. Following rounds of iterative mechanism refinement (including falsification and validation), time-dependent measures of several in silico cystogenesis phenomena, including sizes of cells, cysts, and lumens, cell number, and lumen number, became quantitatively indistinguishable from corresponding in vitro measures. The process led to two successful ISMAs that had similar operating principles but relied on different mechanistic hypotheses for how cells stabilized. In one, cells relied on information about the lumen. In the other, transition to the stabilized state was a simple timed event. Independent in vitro experiments [6], which used molecular interventions to alter the axis of cell division in two different ways, provided data that challenged ISMA mechanisms and the predictions of the cystogenic consequences of such interventions. ISMA mechanisms survived the falsification challenge: measures of cystogenesis during simulation experiments mimicking both interventions were quantitatively similar to in vitro data. This further supported our hypothesis that the cause-and-effect relationships (mechanisms) occurring within ISMAs during in silico cystogenesis (and thus their morphogenic agenda) have in vitro counterparts, both in the presence and absence of mechanistic interventions. By challenging these in silico mechanisms we better understand their in vitro cellular counterparts.

## Results

### Quantitative in vitro results

**Figure 1:**
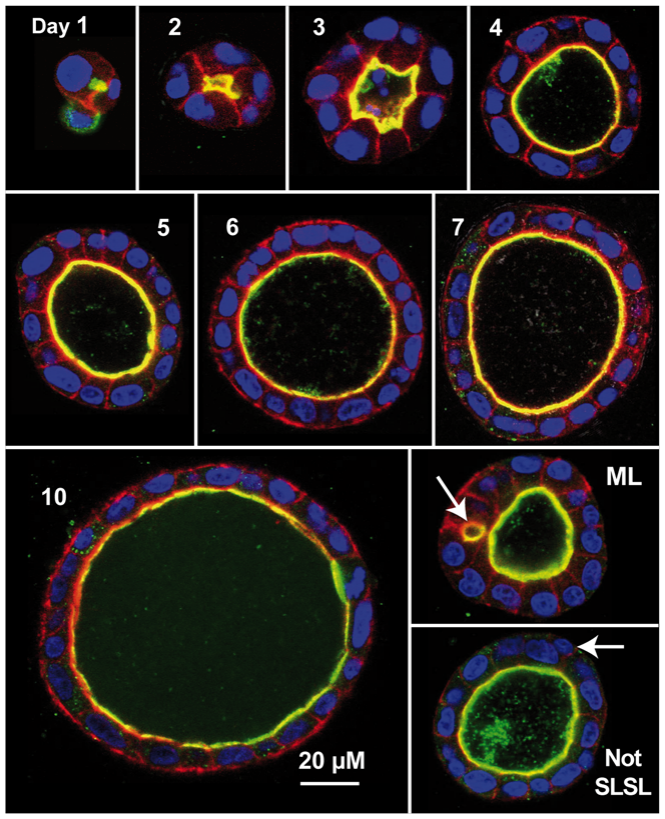
In vitro MDCK cyst cross-sections. Culture conditions were as described in the text. Confocal images were recorded on the indicated day during cystogenesis. Colors reflect component staining as follows: red: actin; green: gp135/podocalyxn; yellow: red and green colocated; blue: nuclein; black: Matrigel. ML: a multi-lumen cyst. The arrow indicates a second, small lumen. Not SLSL: this single lumen cyst does not have a single layer of cells. The arrow indicates a cell not in contact with lumen.

In order to study the process of cyst development in detail, MDCK cells were grown and observed in 3D Matrigel culture for one to ten days and analyzed quantitatively each day. As shown in Figure 1, cysts developed in a manner consistent with previous observations [7]-[9]. A suspension of mostly single MDCK cells divided to form small clusters during the first 24 hours. Most cells polarized (defined by podocalyxin localization at the nascent apical surface of the cell) during the first two days of growth and all cells polarized by day 3. Cysts developed single (11 of 20) or multiple (9 of 20) lumens by the end of day 2. Most cyst cross-sections appeared circular. The deviation from a circle ranged between 2 and 5%.

**Figure 2:**
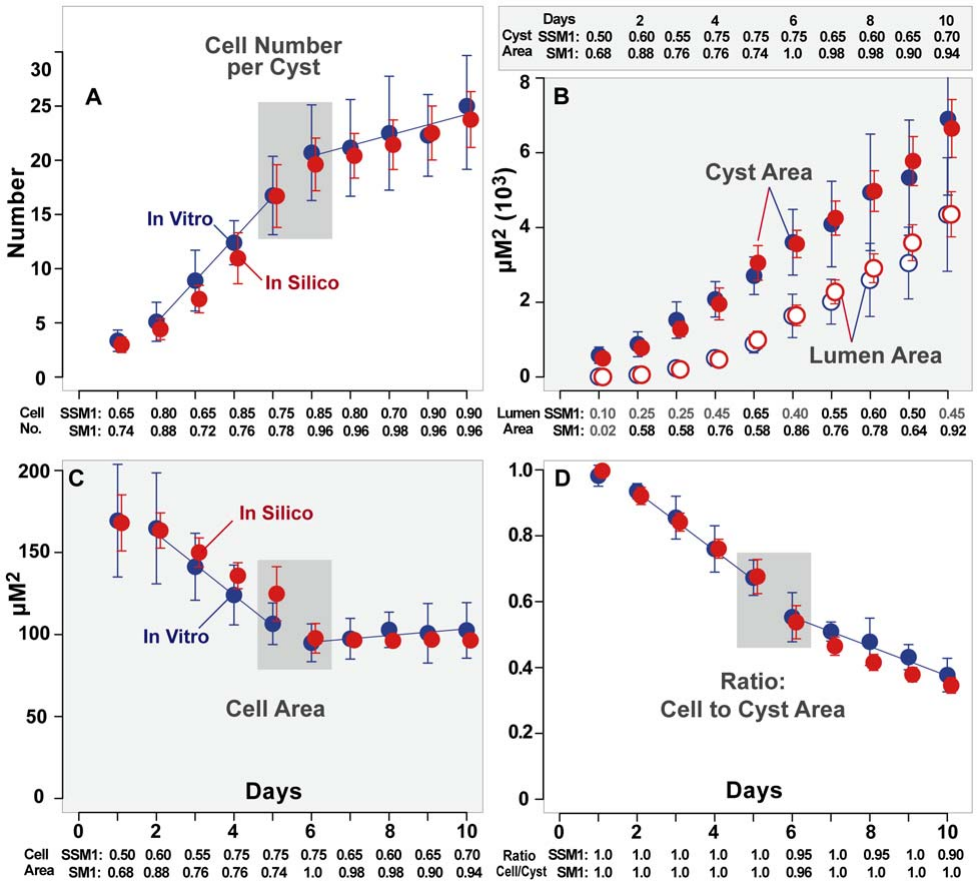
Quantitative measures of in vitro and in silico cystogenesis. Mean values and standard deviations for (A) cell number per cyst, (B) cyst and lumen area, (C) mean individual cell area and (D) ratio: cellular to cyst area. Blue: in vitro data taken each day for ten days from 20 cysts. Red: data taken from 50 cysts over ten days using the parameter values in Table 2. Gray boxes: noted changes in behavior. Blue lines: slope of in vitro growth illustrating changes in rate. SSM1: Self -Similarity Measure of in vitro growth; SSM1 indicates the percentage of in vitro values each day that fell within ±25% of the mean in vitro value for that day. SM1: Similarity Measure for ISMA growth. SM1 indicates the percentage of ISMA values each day that fell within ±25% of the mean in vitro value for that day. The target was that SM1>0.5 for nine of ten days. When the target was met, we posited that ISMA measures were experimentally indistinguishable from in vitro measures. Gray SM values did not achieve targeted values.

**Figure 3:**
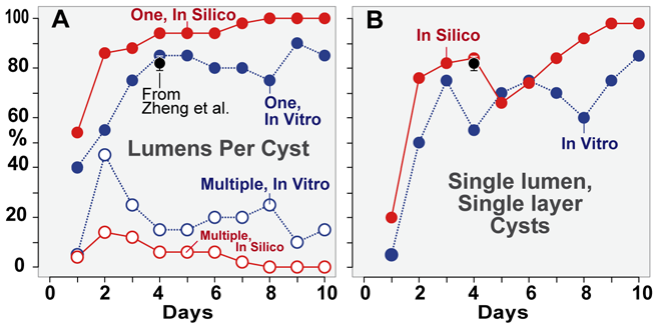
Percentage of cysts with different numbers of lumens. (A) Percentage of cysts that have single (solid circle) or multiple (open circle) lumens. (B) Percentage of SLSL (single-layer, single-lumen) cysts. Blue: in vitro data for 20 cysts taken each day for ten days. Red: in silico data for 50 cysts using parameters values from Table 2. Black: mean and standard deviation for “normal” MDCK cysts observed by Zheng et al. [6] as described in the text. Solid lines represent continuous growth of ISMA cysts. Dotted lines represent discrete growth of MDCK cysts.

We measured and recorded cyst and lumen area and perimeter, cell number, the number of single and multiple lumen cysts, and the number of single-lumen, single-(cell) layer (SLSL) cysts. Results are graphed in Figures 2 and 3. We calculated mean cell area and the ratio of total cellular area to total cyst area.

Cell number increased exponentially through day 5. It slowed and increased at a constant rate after day 6. Coincident with that shift, the variance in cell number per cyst increased (Figure 2A). Cyst and lumen area increased monotonically (Figure 2B). Mean cell size decreased at a constant rate through day 6 (Figure 2C) and then leveled off at roughly the same time that cell division slowed. Mean cell size increased slightly following the shift. Cell size variance was smallest on days 5–8. We did not find a strong correlation between mean cell size and other cyst measurements, including cell number, lumen size, lumen number, or lumen perimeter/cell number. The ratio of total cellular area to cyst area (Figure 2D) indicated that the portion of cyst occupied by cells decreased as cysts expanded (and thus the portion occupied by lumen increased). The ratio decreased quite steeply between days 5 and 6 with very little overlap; the majority of cysts at day 5 had a ratio higher than 0.6 and the majority of cysts at day 6 had a ratio lower than 0.6. These observations taken together indicated a shift in cell behavior occurred at approximately day 5 (referred to hereafter as simply the shift). The data also supports the idea that cell compression during lumen expansion may be a factor triggering cell entry into the stabilized state.

### Lumen percentages in vitro

During the first day of growth, some cysts developed lumens, while others had no visible lumen. From days 2-10 all cysts had at least one lumen (Figure 3A). Multiple lumens appeared in a number of cysts, but their frequency decreased over time. Previous studies [6] considered cysts to be “normal” if they contained a single layer of actin and apical membrane markers surrounding a single lumen. We distinguished between single-layer, single-lumen (SLSL) cysts, in which all cells contact both extracellular matrix and lumen; cysts with a single lumen where some cells did not touch the extracellular matrix or the lumen; and cysts with multiple lumens (Figure 1). After day 2, the percentage of SLSL cysts ranged between 55% and 85% (Figure 3B), in rough agreement with the 80% of cysts observed by Zheng et al. to be “normal” [6]. In cases where single-lumen cysts did not have a single layer of cells, usually only one or two cells did not contact the lumen or extracellular matrix. These data indicate that the percentage of cysts with multiple lumens decreases over time, likely as smaller lumens merge together into larger. It is possible that a few cysts might increase their lumen number over time even as mean lumen number decreased, but that behavior would only be observed using time-lapse microscopy of individual cysts.

### ISMAs capable of cystogenesis

In order to create and validate ISMAs, we used a number of modeling techniques and approaches, detailed in Methods. To avoid confusion between in vitro and ISMA components and mechanisms with similar names, we use small caps when referring to the latter. Following the Iterative Refinement Protocol (IR Protocol) led to two specifications of cell behavior that achieved all targeted attributes in Table 1 and all prespecified Similarity Measures (SMs; described below). They are the lumen stabilized ISMA (LS ISMA) and the timed stabilization ISMA (TS ISMA). There are only three cell states: unpolarized, polarized, and stabilized. Both LS and TS ISMAs have a common morphogenic agenda. It is a consequence of their operating principles, which are a networked consequence of cell state and micromechanisms. The latter are primarily axiom-dependent, and the axioms, in turn, depend on particular local and temporal conditions. The axioms are placeholders for even more fine-grained micromechanisms.

**Table 1:** Targeted attributes and specifications.

**1**.	**A:** An initial small cluster of 1-4 cells divides and increases in cell number.
	**S:** The ISMA begins with 2-4 cells, which divide after *cycleCounter* reaches 0.
**2.**	**A:** All cells polarize by the second day of growth.
	**S:** Cells change state to polarized after *polarCounter* reaches zero.
**3.**	**A:** One or more lumens develop by the second day of growth.
	**S:** Cells within cysts form lumens after cells polarize.
**4.**	**A:** A multilayer of cells separates multiple lumens.
	**S:** Cells only form lumens when they and their neighbors do not already contact lumen. After a lumen has formed, all neighboring cells contact a single lumen.
**5.**	**A:** Cells can undergo apoptosis whether or not they contact the extracellular matrix.
	**S:** Cells die with specified probability. That value is larger for cells not in contact with matrix.
**6.**	**A:** The increase in cell number over time is similar that shown in Figure 2, leveling off at day 6.
	**S:** When lumen size reaches a critical value, a mechanism causes cells to stabilize.
**7.**	**A:** The increase in cyst size over time is similar to that shown in Figure 2.
	**S:** Cyst size is a function of cell area, cell number, and lumen size.
**8.**	**A:** The increase in lumen size over time is similar to that shown in Figure 2.
	**S:** Lumen size is a function of cell number, cyst perimeter, cell stretch, and time.
**9.**	**A:** Mean cell area decreases over time as shown in Figure 2, and levels off at day 6.
	**S:** Cells have distinct mechanisms for (effectively) calculating TA before and after stabilization.
**10.**	**A:** The decrease in the ratio of cellular to cyst area over time is similar to that in Figure 2, decreasing faster during days 2-6.
	**S:** Cell area, lumen size, and cyst size must be measurable and if these quantities validate, then so must the ratio of cellular to cyst area.
**11.**	**A:** The percentage of single-lumen, multiple lumen, and SLSL cysts each day is similar to that in Figure 3.
	**S:** When cells lack lumen contact, they can create new lumens. Lumen creation occurs at the site of previous cell division. Lumens can expand and merge. Cells that have stabilized cannot create a new lumen.
**12.**	**A:** The percentage of cysts with apoptotic cells each day is similar to that observed in [9].
	**S:** Cells shrink after beginning to die. The percentage of cysts with dying cells is calculated as in vitro.
**13.**	**A:** When the orientation of the cell axis of division is disrupted or reversed, the percentage of normal cysts is reduced as observed in [6].
	**S:** Cells orient their axis of division toward the center of prior division or toward the center of the lumen. Axis orientation can be randomized and reversed.

MDCK cells and cysts are the referent. The model system is called an in silico MDCK analogue (ISMA). **A:** a targeted attribute; **S:** an ISMA specification. All listed attributes were achieved. The early version of the ISMA achieved TAs 1-4, but was falsified by the quantitative data. The refined ISMA achieved all TAs except 11, which was achieved by both the LS and the TS ISMAs.

The only difference between the LS and TS ISMAs is the mechanism used by polarized cells to shift to the stabilized state. Within the LS ISMA, polarized cells use information about the lumen to decide when to stabilize. Within the TS ISMA, transition to the stabilized state is a simple timed event (each cell used its own internal clock). We did not discover any in vitro observations that would provide a basis for selecting one micromechanism over the other.

Cell operating principles require each cell to have knowledge of its internal state and immediate environment, including the size of the neighboring lumen (for the LS ISMA). Cell division is based on factors other than cell size. Early in the process, cyst size can be independent of lumen size. The orientation of cell division is extremely important in influencing the formation and number of lumens within a cyst.

We explored alternative mechanistic variations, but failed to find others of comparable simplicity capable of achieving all targeted attributes and prespecified SMs. For simplicity we present and discuss measures from LS ISMA simulations within the text (Figures 2 and 3) and provide the same simulation measures for TS ISMAs in Figures S1 and S2. Results from earlier ISMA that were falsified because they failed to achieve one or more SMs are also discussed.

### Quantitative results in silico

**Figure 4 pcbi-1002030-g004:**
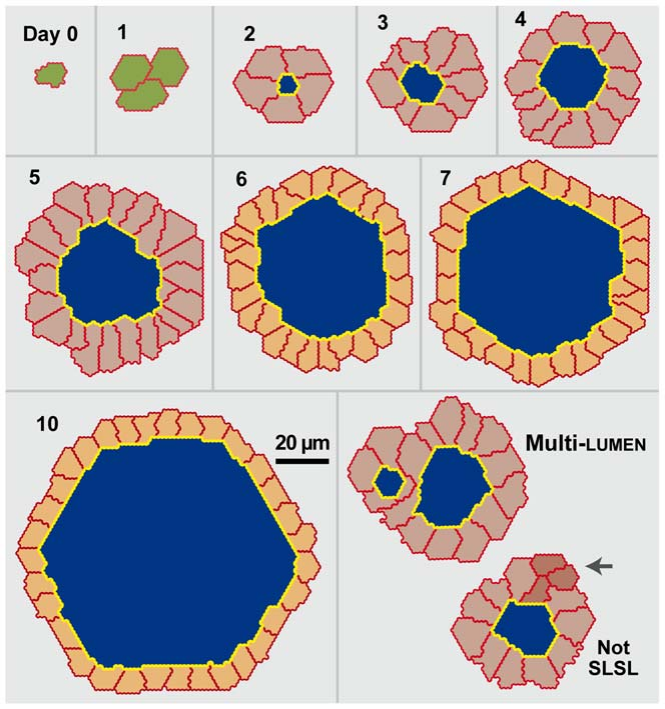
In silico MDCK analogue cyst cross sections. Note that a regular hexagon in hexagonal space maps to a circle in continuous space. Images are from a single simulation run using parameter settings from Table 2. Cells are unpolarized (green), polarized (gray) or stabilized (orange). Cell-cell and cell-matrix borders are red; cell-lumen borders are yellow; lumens are blue. Lower right panel: shown is a multi-lumen cyst. Not SLSL: this single lumen cyst does not have a single layer of cells. The arrow indicates two cells not in contact with lumen.

ISMA cysts were similar to cysts grown within Matrigel (Figure 4). Cysts began with 1-3 cells at day 0. Cells polarized and formed lumens within the first two days (Figure 3 and Video S1). Lumens and cysts expanded at a rate indistinguishable from that observed in vitro. In general, a cyst formed with a single lumen surrounded by a single layer of polarized cells (Figure 4 and Video S1). Occasionally multiple lumens formed, each separated by an independent layer of cells, such that no cell contacted more than one lumen (Figure 4 and Video S2). The ISMA successfully achieved all qualitative and quantitative targeted attributes listed in Table 1.

ISMA cell number also exhibited two growth phases, with the rate of cell division decreasing at day 6 (Figure 2). Lumen and cyst size increased at rates similar to those observed in vitro, but standard deviations were smaller. Cell size also decreased at a rate comparable to in vitro, and its standard deviations were also smaller. As indicated by the values of Similarity Measure 1 (discussed below) in Figures 1 and 2, ISMAs produced quantitative results similar to in vitro values. ISMAs were executed using the parameter settings in Table 2, and cyst and lumen area were scaled by 2.25 µm^2^ and perimeter by 0.75 μm.

**Table 2:** Primary ISMA parameters.

Parameter	Description	Default value	Range used
*wedgeArea*	W: target area of unpolarized cells and ideal wedge area for polarized cells	82 grid points	30-150
*lambdaArea*	Multiplier controls how quickly cells change size to reach their individual target areas	5 grid points	0.5-20
*stableTargetArea*	Target area of stabilized cells	48 grid points	30-150
*cellCycle*	Used to calculate *cycleCounter*, the number of simulation cycles before a cell divides	70 simulation cycles	20-100
*lambdaPerim*	Multiplier controlling how quickly cells change size to reach their target perimeter	2.5	0.5-10
*polarDelay*	Used to calculate *polarCounter*, the number of simulation cycles elapsing before an unpolarized cell polarizes	42 simulation cycles	0-400
*shiftDelay*	In the TS ISMA, used to calculate *shiftCounter*, the number of simulation cycles elapsing before a polarized cell stabilizes	140,000 simulation cycles	0-300
*doublingArea*	When divided by 2, the minimal area a cell must have to divide	41 grid points	20-100
*divisionReg*	How the axis of division is calculated	1	0, 1, 2, 3
*multiplier*	Used to calculate target perimeter of cells	0.6	0-1
*lumenGrowthRate*	Multiplier controlling rate of lumen expansion	0.003	0-1
*deathRateLumen*	Likelihood of cells to die when not touching matrix	0.02	0-1
*deathRateEpi*	Likelihood of cells to die when touching matrix	0.0004	0-1
*clusterProb*	Probability initial two cells will set *cycleCounter* to zero at simulation cycle 1	0.8	0-1
*lgrSubtract*	Multiplied by cell stretch to reduce lumen expansion	27	0-300
*dyingShrinkRate*	Amount subtracted from target area of dying cells each simulation cycle	9 grid points	0-100
*stableRatio*	Critical lumen size (multiplied by 1000) at which cells will stabilize	0.5 grid points	0.1-1
*stableCycleDelay*	(1 – x) = probability a stabilized cell will decrement *cycleCounter*	0.85	0-1

Parameters critical to the operation of the ISMA are listed along with descriptions, default value used for simulation, and the range of values explored. To switch between the LS ISMA and the TS ISMA the values of *shiftDelay* and *stableRatio* are changed from 140,000 and 0.5 to 200 and 1000. All units are relational (e.g., grid points instead of µM, simulation cycles instead of hours).

### Lumen percentages in silico

Simulations produced single and multiple lumen cysts at frequencies comparable to those observed in vitro (Figure 3A), though the percentage of cysts with single lumens was slightly higher than observed in vitro. The percentage of SLSL cysts (Figure 3B) leveled off between days 2 and 6 and then increased steadily to day 10 as lumens merged. Cells that stabilized were not allowed to create new lumens, but could contribute to lumen expansion. If this restriction were to be removed and cells were allowed to create new lumens after they stabilized, the percentage of SLSL cysts might remain steady or decrease.

### Similarity measures

To provide a validation target for ISMA cystogenesis and to compare ISMA and in vitro results, we developed SMs [10], which quantified the similarity within and between the in silico and in vitro data. We posit that, if in silico data satisfies the SMs, then that data would be indistinguishable from data produced by a repeated in vitro experiment.

SM1 compared results from individual simulations to in vitro mean values, indicating the similarity of in silico and in vitro results. SM1 is the percentage of in silico observations that fell within±25% of the mean in vitro value for a given measure. SM1 values are listed in Figure 2. To survive falsification, >50% of simulations must achieve the SM1 target for nine of ten days, as detailed in Methods. For example, the ±25% range for in vitro cell number at day 3 was 6.7 to 11.1 with a mean of 8.9. Seventy-two percent of simulations had cell numbers within that range at day 3. SM1 values for cell number, cyst size, mean cell area, and the ratio of cellular to cyst area exceeded 50% at all days, so a degree of validation was achieved. The SM1 value for lumen size exceeded the 50% cutoff for nine of ten days, although the values were lower.

To facilitate assessing SM1 values and comparing in vitro and in silico data, we specified and used Self-Similarity Measure 1 (SSM1). It measured the similarity between the in vitro mean value and individual in vitro values and thus how closely grouped around the mean the individual in vitro values were. Similar to SM1, SSM1 is the percentage of individual in vitro cyst measures each day that fall within a specified range. SSM1 can be used to evaluate corresponding SM1 values. Large SSM1 values are a characteristic of measures having a small variance. Values of SSM1 were larger than the target for all measures except lumen size, indicating that lumen size in vitro varied more extensively about the mean than other quantities.

SM1 did not consider the variance of the data. To address variance, we specified SM2. It compared the coefficient of variance of in silico and in vitro experiments. SM2 measured the absolute value of the difference between the in vitro and in silico coefficient of variance each day. ISMAs survived falsification if SM2<0.15 for nine of ten days (strong validation) or <0.25 for eight of ten days (medium validation). The current ISMA achieved strong validation for cell number, mean cell area, and the ratio of cellular to cyst area (Table S1). It achieved medium validation for cyst size and lumen size, comparable to SSM1 values.

### Cell death

**Figure 5:**
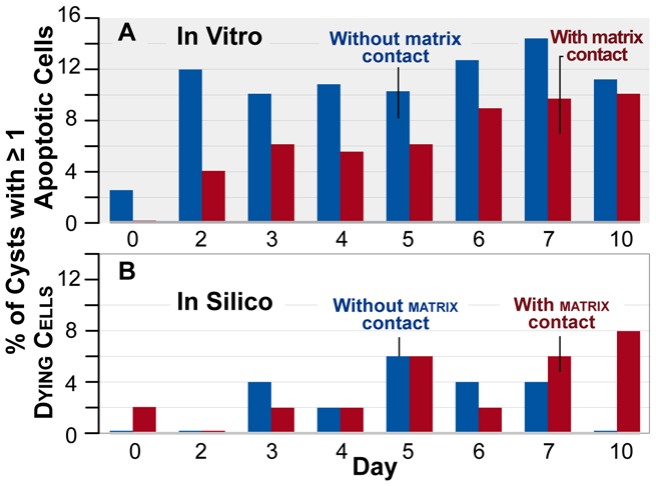
Percentage of cysts with dying cells. (A) In vitro data reproduced from [9]. (B) ISMA data from 50 cysts over ten days. Blue bars: percentage of cysts observed to have apoptotic cells without matrix contact. Red bars: percentage of cysts observed to have apoptotic cells with matrix contact.

When MDCK cells can polarize well, they do not need apoptosis to form cysts with lumens [9]. Consequently, cell death is relatively uncommon during in vitro MDCK cyst development [9]: on a given day, no more than 15% of cysts had one or more apoptotic cells within the lumen and no more than 10% of cysts had one or more apoptotic cells with matrix contact. Cell death did occur during ISMA executions, but at slightly lower frequencies than observed in vitro (Figure 5). In Methods, we specified that the average duration between a cell initiating death and being removed from the simulation to be ten simulation cycles, which maps to five hours. The actual in vitro duration will affect the number of visible apoptotic cells observed each day. When we caused cells to shrink somewhat slower, the cell death values in Figure 5B increased. The experimental results provided in Figure S3 demonstrate that decreasing the value of *dyingShrinkRate* from 9 to 4.5 increased the mean duration of cell death (from 4.6 to 7.4 hours) and increased the percentage of dying cells. It is noteworthy that all validation targets were achieved without requiring stabilized cells to die more frequently than polarized cells. Based on current knowledge, the ISMA accurately mimics in vitro quantitative data, but the duration of apoptosis within MDCK cells in vitro has not been quantitatively established. In order to be certain about the role played by cell death, time-lapse movies using a caspase-3-GFP will be required.

### Altering cell division orientation in silico dramatically alters cyst morphology

**Figure 6:**
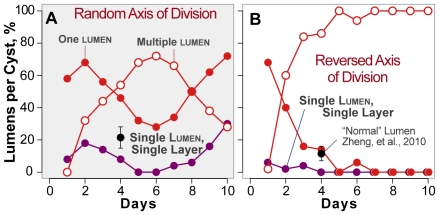
Percentage of ISMA cysts with varied lumen number when the axis of cell division is abnormal. Shown are the percentages of cysts that have single (solid red circles) or multiple (open red circles) lumens when the axis division is (A) random or (B) reversed (rotated 90°) along with the percentage of cysts that are SLSL (purple circles) when the axis of cell division is (A) random or (B) reversed. Black (A and B): mean and standard deviation for “normal” MDCK cysts observed by Zheng et al. [6]. The in vitro control data are shown in Figure 3.

After the ISMAs achieved the above, targeted attributes, Zheng et al. [6] reported measuring the consequences of disrupting cell division orientation on MDCK cyst morphology. Knocking down LGN, which plays a role in spindle orientation during cell division, caused cell division orientation to become random instead of aligning with the axis perpendicular to the cellular plane. The frequency of “normal” cysts decreased from roughly 80% to 20-30%. We added those observations to our targeted attributes list and then explored the degree to which cyst morphology following a comparable ISMA intervention would mimic the in vitro results, thus surviving the challenge. We altered cell division so that all cells divided with a random orientation. The results (Figure 6A) were similar to those of Zheng et al. The altered ISMA produced less than 20% SLSL cysts and more than 30% multi-lumen cysts at days 2 through 9. Additional details are available in Figure S4.

In a second experiment, Zheng et al. targeted LGN to the apical membrane. So doing rotated the axis of division by 90°, thus reversing cell division orientation. The procedure reduced the frequency of normal cysts to roughly 10%. We conducted a similar experiment by modifying ISMAs so that the axis of division was parallel, rather than perpendicular to the lumen edge. That intervention produced SLSL cysts less than 10% of the time (Figures 6B and S5). ISMAs survived both challenges; in both cases, altering the orientation of cell division decreased the percentage of single lumen and SLSL cysts to a degree similar to that observed within in vitro experiments.

### In silico cyst growth with no luminal cell death

**Figure 7:**
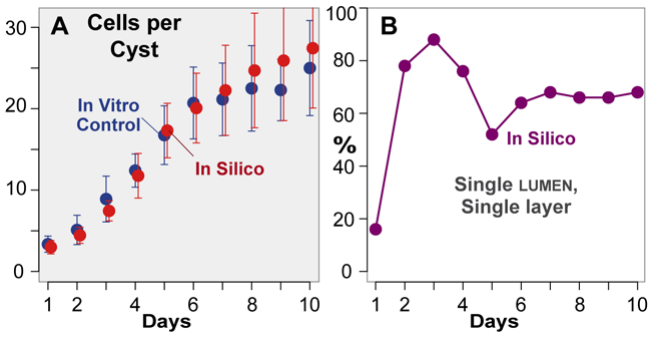
Cystogenesis measures with no luminal cell death. ISMA simulations executed with the parameter values from Table 2 except that luminal cell death was not allowed. (A) Red: mean values and standard deviations for cell number per cyst. Blue: in vitro control data from Figure 2A. (B) Percentage of SLSL cysts.

Cell death contributes to cystogenesis, but it remains unclear to what extent it is essential. In order to explore the consequences of decreased cell death frequency, we executed simulations in which we reduced *deathRateLumen* from 0.02 to 0.0. We did not alter the probability of cell death in cells contacting matrix. We noted no significant difference in cell number during the first six days of growth, but during days 7 through 10 mean CELL number was 10-15% higher than observed during control ISMA growth (Figure 7A). The observed standard deviations also increased. We observed a smaller percentage of SLSL cysts than in control simulations, especially during days 6 to 10 (Figure 7B). Values for cyst area, lumen area, cell size, and the ratio of cellular to cyst area were similar to control values (Figure S6), while the percentage of single lumen cysts decreased slightly (Figure S7).

### Simulated cyst growth with delayed cell polarization

**Figure 8:**
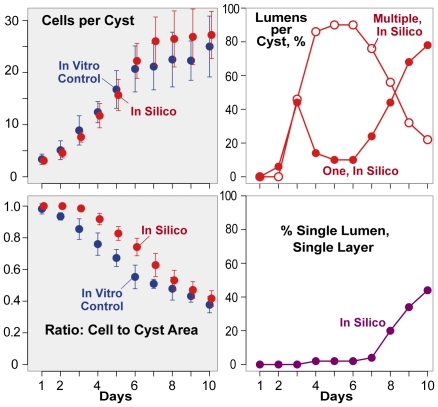
Cystogenesis measures when cell polarization was delayed. ISMA simulations executed with the parameters values from Table 2 except that cell polarization was delayed as described in the text. Left: mean values and standard deviations for cell number per cyst (top panel) and ratio of cellular to cyst area (bottom panel). Right: Percentage of cysts with single, multiple, and SLSL lumens. Designations and symbols are the same as in Figures 2 and 3.

Delayed cell polarization is believed to contribute to the differences in cyst growth in Matrigel and collagen [9], although it is possible that a lower initial rate of cell clustering and a slower growth rate might be factors as well. To explore the effect of delayed polarization on ISMA cystogenesis, we increased the value of *polarDelay* from 42 (equivalent to 21 hours) to 130 (equivalent to 65 hours). Relative to controls, cell number increased at an equivalent rate during the first six days, but was larger during days 7–10 (Figure 8A). Cell polarization (data not shown) and lumen formation occurred later than in controls (Figure 8B). The area taken up by cells remained roughly constant, but the delay in lumen formation and resulting smaller lumens caused the ratio of cellular area to total cyst area to be significantly larger than control values during days 2-8 (Figure S8). Not surprisingly, there were fewer single and multiple lumen cysts during the first three days. When lumen formation began, however, it often resulted in multiple lumens (>80% for days 4–6); SLSL cysts were observed infrequently. As lumens expanded and merged during the later stages of growth, the frequency of SLSL cysts increased. The percentage of dying cells not contacting the matrix was significantly larger at days 4–10, indicating that many of these cells died as lumen expansion occurred (data not shown). Some of these in silico results reflect those observed within growth in collagen, but it seems unlikely that delayed cell polarization in vitro is solely responsible for those differences.

## Discussion

### In vitro observations

Observations reported herein about in vitro MDCK cystogenesis are consistent with those made previously [6], [9], [11]. There is no evidence of behavioral differences between cells within single and multiple lumen cysts. We could not establish a causative connection between the slowing of cell division and the change in cell size. The evidence indicates that initial lumen expansion is somewhat isochoric: early lumen expansion is primarily a consequence of cell shrinkage. After an interval of lumen expansion and cell shrinkage lasting about six days, cell behavior changes: cell size stabilizes and cells begin to stretch as the lumen continues to expand (Figure 1); cell division slows dramatically; the expanding lumen becomes the primary driver of cyst size; and the variance in both cell area and cyst size increases.

### Iterative process

Iteratively constructed ISMAs quantitatively mimicked a targeted set of in vitro data and cell behaviors. Measures of ISMA cystogenesis matched corresponding measures of MDCK cystogenesis over ten days (Figures 2, 3, & 5). The pathways and proteins that play influential roles in cell behavior during MDCK cystogenesis are objects of active research and are increasingly well understood. However, knowledge of how specific cell actions and events are choreographed during cystogenesis is still limited. The latter knowledge is needed to begin establishing causal linkages between molecular level events and systemic phenotype.

Previous analogues [2], [3] used a simple representation of a cell: each cell occupied a single 2D hexagonal grid space. They were falsified when we added qualitative observations about changes in cell size and shape to our targeted attributes list (Table 1). To mimic these newly targeted attributes, we needed cells to be more fine-grained. To generate the current ISMA, we began with an in silico analogue that had achieved a degree of validation and then conducted in vitro experiments designed to challenge and possibly falsify it. We then reengineered the in silico system to reflect, explore, and challenge new insight provided by the fresh in vitro data. We engineered new analogues using the cellular Potts model (CPM), which provided several capabilities, including enabling cell size and shape change. To slow the increase in cell number after day 6, we introduced a stable cell state.

We envision the above in silico-wet-lab cycle continuing indefinitely. It is straightforward to explore the consequences of in silico mechanistic interventions. If these interventions result in altered system behaviors (predictions), it may suggest new in vitro experiments designed to test them. Examples include the effect of delayed polarization on cyst phenotype, the lack of noticeable changes when cell death is inhibited, and the causal link between lumen size and cell stabilization. Furthermore, we expect a change in cell state (cell stabilization at day 6) to be accompanied by measurable changes in gene expression profiles and biochemical signaling.

### Improved analogue

The ISMA illustrated in Figure 4 achieved all targeted attributes. It was preceded by two earlier versions. These ISMAs differed in the mechanism used to initiate cell stabilization. We hypothesized that in vitro cells might use knowledge of their internal geometry to sense their perceived stretch and subsequently stabilize. One early analogue, the geometrical mechanism ISMA (GM ISMA), directly tested this hypothesis; each cell used measures of its area and geometry to determine when to shift to the stabilized state. To achieve a degree of validation required the use of an axiom specifying that stabilized cells would be more likely than polarized cells to die when not in contact with matrix. This axiom was implemented in order to decrease the number of cells within the lumen and thus increase the number of SLSL cysts. The GM ISMA was falsified when targeted SMs for the percentage of single lumen, multiple lumen, and SLSL cysts were strengthened to those achieved in Figure 2 (Figures S9 and S10). It was falsified because the time at which cells stabilized was too variable; some cells stabilized early, others much later, resulting in very few SLSL cysts (data not shown).

A second version, called the timed stabilization ISMA (TS ISMA), used an internal clock to signal cell stabilization, resulting in a uniform stabilization time and reducing the variance in cyst size. The TS ISMA survived falsification (Figure S1), providing evidence that stabilization time influences SLSL cyst percentages. The GM ISMA axiom specifying that stabilized cells would be more likely than polarized cells to die when not in contact with matrix was not needed. The TS ISMA was capable of generating high percentages of SLSL cysts even without this axiom, and so the axiom was removed in that and subsequent ISMAs.

Although the TS ISMA survived falsification, we were not aware of any in vitro evidence suggesting existence of an equivalent internal clock-based mechanism. If such a mechanism does exist, it might be molecularly equivalent to that of cell polarization. Genes that regulate cellular senescence can suppress the cell cycle, and the sirtuin protein SIRT1 is involved in cellular senescence [12], [13]. It is possible a cell-autonomous timing mechanism could exist that depends on the regulation of SIRT1 and its downstream targets, as detailed in Supporting Text S1. We hypothesized that a mechanism that used the geometry of the lumen instead of the geometry of individual cells to signal cell stabilization might bridge that gap and still produce a low variance in stabilization times. We developed the lumen stabilized ISMA (LS ISMA) described within this report to test that hypothesis and discovered that in addition to surviving falsification (Figure 2) it generated stabilization variance between the GM and TS ISMAs. We can surmise a mapping between the lumen-based stabilization mechanism and a functionally equivalent in vitro mechanism in which apical sensory input to each cell provides it with information that correlates to lumen size. Current evidence supports the hypothesis that cells in the cyst wall can sense lumen size. One mechanism utilizes the tension generated at the luminal membrane by membrane stretching. This tensional information is transduced by the subapical F-actin network, which acts both as a scaffold for maintaining luminal integrity, as well as a region for aggregation of recycling endosomes that regulate the protein and lipid composition of the apical plasma membrane. Thus, regulators of this F-actin network can regulate lumen and cyst size. Potential molecular mechanisms are detailed in Supporting Text S1.

We should seek additional, in silico mechanisms that are equally effective in enabling ISMAs to achieve validation targets. Given phenomena, what hypothetical generators (and measures) might generate them? Studying an inverse mapping requires multiple, seemingly plausible hypotheses, which then compete against each other during simulation experiments as done here. After falsification and validation using the IR Protocol, those that survive spawn additional, more refined hypotheses. Having multiple mechanistic options for realizing the same behaviors may be biomimetic in that it marginally increases system robustness. An example of a potential additional in silico mechanism is one that uses time-dependent dynamic parameters, which might assist in the exploration of finer-grained, intracellular molecular behaviors.

ISMAs currently contain a small number of parameters that can have implicitly dynamic values (such as the time that elapses between cell division events). They change when cells change state. In general, however, all parameters are fixed for the duration of the simulation. Expanding the set of targeted attributes may force consideration of time varying parameter values. If, for example, in vitro data were targeted that demonstrated the build-up of certain proteins along the plasma membrane, dynamic variables could be implemented that controlled the amount of the protein counterpart within the analogue.

### Challenging ISMA predictions

ISMAs had already achieved all targeted attribute when the work of Zheng et al. [6] was published. Results from their studies provided an independent challenge to ISMA mechanisms and their robustness. The simulation results in Figure 6 are a consequence of two different simulation interventions: making the cell axis of division random (Figure 6A) and reversing the cell axis of division (rotating it 90°)(Figure 6B). These predictions are fully consistent with the in vitro results of Zheng et al. As previously stated, they defined a normal cyst as one with actin staining at the apical cell surfaces surrounding a single lumen. Included in that definition are our SLSL cysts and cysts with a single lumen. In Zheng et al., when cell division was randomized, the percentage of cysts with single lumens at day 4 dropped from 81.9% to 21.5%, a different of 60.4%. In ISMA simulations, when *divisionReg* was changed from 1 (ordered division) to 0 (random division) the percentage of cysts with a single lumen dropped from 94% to 46%, a difference of 48%, which is quite similar to the decrease observed in vitro (Figure 6A). As seen in Figure 6B, when the axis of division was reversed, the percentage of cysts with a single lumen dropped from 81.9% to 11.5%, a difference of 70.4%. Within the ISMA, when *divisionReg* was changed from 1 to 3 (reversed division), the percentage of cysts with a single lumen dropped from 94% to 14%, a difference of 80%. In addition, the in silico results provide a prediction of in vitro behavior that could be challenged through in vitro experimentation. When division is reversed within the LS ISMA (Figure S5A) cell number continues to increase after day 6, most likely because the numerous small lumens do not reach a sufficient size to cause cell stabilization. In strong contrast, when division is reversed within the TS ISMA (Figure S11-2) cell number stops increasing at day 5 and remains stable thereafter. Future experiments of the type conducted by Zheng et al. that quantify cytogenesis over longer intervals would provide evidence supporting one or the other mechanistic hypothesis.

### Cell-level and intracellular events

A cell-level event is one that is visible at the current level of resolution. An event that maps to an intracellular process (referred to as intracellular) can occur without causing a visible change; it is below the current level of resolution. Of the events listed in Table 3, the two marked (*) only exist within the in silico system and have no specific in vitro counterpart.

**Table 3:** Cell and intracellular events that can occur within a simulation cycle.

Cell-Level Events	Map to Intracellular Events
Cell state (& color) changes	*MCell* point assignment*
Cell division	*CellCycle* updating at simulation cycle 1
Lumen creation	Cell initiates dying
Lumen merging through TJ reorganization	Death advances; cell TA decreases
Lumen expansion through TJ reorganization	Polarity counter (*polarCounter*) begins
Lumen expansion without TJ reorganization	Decrement *cycleCounter*
Isolated point engulfed*	Decrement *polarCounter*
Cell perimeter (but not TJs) changes	Decrement *shiftCounter* (TS ISMA only)
Dying complete: cell disappears	Compute matrix and lumen contact length, A, TA, & TP
Matrix removal	Compute G for a potential index change
Cells change shape	

*This event exists only within the ISMA system and has no specific cystogenesis counterpart.

All cell events produce a visible change within the ISMA visualization. Events that map to intracellular events result in a change within a cell, but do not produce a visible change within the ISMA. Cell-level events map to equivalent events between in vitro MDCK cells, lumen, and matrix, while intracellular events map to events (less well understood) within in vitro MDCK cells.

Beyond simply modeling cystogenesis, a purpose of this research has been to instantiate an in silico system in which cells, matrix, and lumen have in vitro counterparts, and when executed the ISMA produces a variety of measurable phenomena that quantitatively mimic MDCK cystogenesis. At the systemic level, we have excellent cystogenesis similarity over ten days for multiple measures (Figures 3-5). Further analogue improvement will, following additional cycles of the IR Protocol, allow intracellular events to become concretized and increasingly fine-grained, thus enabling quantitative in silico-to-in vitro mappings at multiple levels.

**Figure 9:**
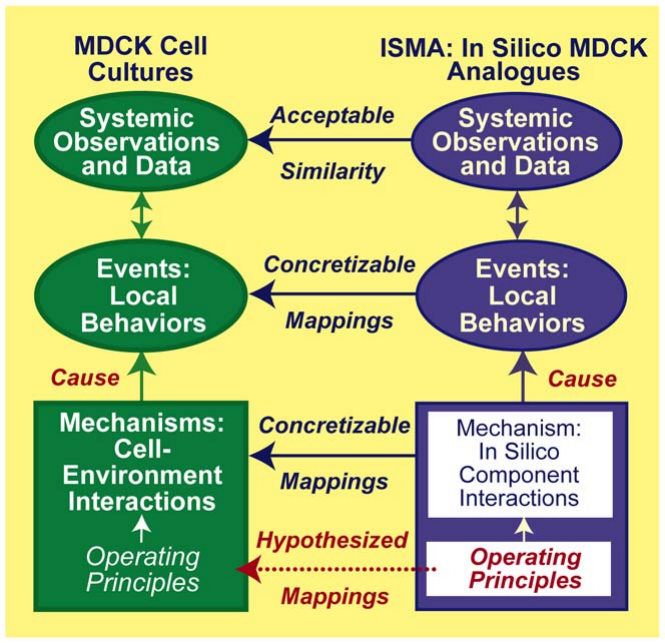
ISMA-to-in vitro cell culture mappings. Left: MDCK cell cultures are the referent wet-lab systems. During experiments, cells draw on genetically controlled operating principles, and cystogenesis is the result. Influential mechanistic details are reflected in the collected data. Right: an abstract mechanistic description, a set of targeted attributes, and specifications paired to those attributes direct analogue design. Software components are designed, specified, coded, verified, and assembled guided by that mechanistic description. The product of the process is a collection of abstract mechanisms rendered in software. A clear mapping is intended between ISMA cells, their axioms and operating principles, and MDCK cell and intracellular details. Relative similarity is controlled in part by parameterizations. Importantly, that mapping can be concretized iteratively. Compilation and source code execution gives rise to a working ISMA. Its dynamics are intended to represent abstractly corresponding dynamics (both observed in movies and believed to occur) within cultures during ten-day experiments. That mapping can also be concretized iteratively. Measures of cystogenesis provide time series data that are intended to be quantitatively similar (according to prespecified criteria) to corresponding measures of MDCK cell cystogenesis. Achieving increasingly stringent SMs provides degrees of validation.

All specified events were necessary and essential for achieving targeted SMs. For cell-level events, the mappings are clear: they are direct and quantifiable. Intracellular events, axioms, and protocols are below the current level of resolution. There is no requirement that a specific intracellular event, axiom, or protocol has a cell-level counterpart. We simply hypothesize that the *set* of intracellular events, axioms, and protocols—a cell's operating principles—has an in vitro counterpart, as illustrated in Figure 9. For some intracellular events, conceptual mappings are clear. Examples include cell initiates dying, death advances, and decrement *polarCounter.* For others, conceptual mappings are less clear. Examples include decrement *shiftCounter* (in the TS ISMA), compute TP, and compute G. The expectation is that, in moving forward, as axioms are replaced by concrete, interacting components (see [14] and the *future experiments* subsection below) clear mappings will be easier to establish and quantify.

A good example of a project in which intracellular events are incorporated and to some degree mapped back to those in vitro, is the IBCell model [15], [16]. It is a biomechanical model of MCF-10A cell cystogenesis in which proteins on the outer cell membrane and the extracellular matrix are specifically simulated. The IBCell model successfully reproduced some aspects of cystogenesis, but it remains unclear whether the intracellular details are necessary or could be replaced by coarse-grained components. The quantitative data used to validate the model lacked the level of resolution necessary to falsify intracellular mechanisms.

### Cell death and the timing of cell polarization

Surprisingly, cysts with little or no cell death can still be well organized with a single lumen. Reducing cell death rates (Figure 7) altered cystogenesis details only marginally, primarily because cell death frequency was already low (Figure 5B). Lin et al. [17] hypothesized that apoptosis is crucial for lumen formation in MDCK cysts, but they reached that conclusion based on observations of cystogenesis in collagen culture only. Martín-Belmonte et al. [9] observed that apoptosis within Matrigel cultures is less frequent than within collagen cultures. Within ISMA simulations, earlier lumen formation results in more organized cyst growth and fewer cells that die after losing contact with the matrix once lumens have formed. It is possible that apoptosis acts simply as a cleanup mechanism within MDCK cysts, but the degree to which it is utilized depends on the environment, the rate of cell growth, and the timing of polarization. Our experiments reducing the rate of cell death showed that although the rate of cell death within cysts during growth is normally quite low, cell death still contributes to controlling cell number and maintaining SLSL cysts. It is possible that environmental adjustments may provide conditions in which MDCK cell cystogenesis produces normal SLSL cysts without requiring cell death, as occurs in human alveolar type II epithelial cells [18], [19].

Relative to cystogenesis in Matrigel, cells grown in collagen produce smaller cysts with fewer cells and delayed polarization. That delay might play a role in formation of smaller cysts. However, ISMA experiments showed that delaying polarization (Figure 8) increased cell number and decreased the percentage of cysts with single lumens. We take those observations as strong evidence that delayed cell polarization alone is insufficient to account for that difference in cystogenesis within collagen and Matrigel cultures.

### Future in vitro experiments

As illustrated in Figure 9, a goal is to build, expand, and validate in silico mechanistic networks that map to plausible causal linkages between intracellular details and features of MDCK cell phenotype in culture. A prerequisite is to have cells capable of achieving increasingly fine-grained and expanding coverage of MDCK cell, cluster, and cyst behaviors under different conditions. Advances in imaging technology have made doing so easier. Similar coverage will be needed of intracellular (subcellular) dynamics, including the behaviors of cell components under different conditions. We anticipate that studies of in vitro MDCK cell cystogenesis using high-resolution, time-lapse microscopy will reveal new behavioral details at each level. Recent studies have employed confocal time-lapse microscopy to understand lumen formation, but only imaged cells for eight hours [20]. Ewald et al. [21] set the standard for long-term time-lapse microscopy in their work on the elongation of mouse mammary ducts, in which they captured individual images every 15 minutes for five days, using high-sensitivity cameras to avoid phototoxicity.

There is ample evidence that tension within the extracellular matrix influences epithelial cell behaviors [22], [23]. Paszek et al. [22] demonstrated that increasing matrix stiffness resulted in tumorigenic behavior in MCF-10A cells. It seems reasonable to expect changes in MDCK cell, cluster, and/or cyst behaviors as Matrigel stiffness, density, and additives are changed. Experiments similar to those within [22] conducted with MDCK cells and for longer durations are needed to expand ISMA coverage of MDCK phenotype in important ways.

Although the underlying in vitro molecular mechanisms to which the TS and LS ISMA map remain unclear, in vitro experiments may indicate one mechanism as being more plausible. Careful analysis of images generated through time-lapse microscopy is expected to be informative. If the elapsed time between individual cyst polarization and stabilization of division rate or mean cell size are similar between cysts, that would be supportive of an internal clock mechanism. However, if the interval varied between cysts, that would falsify such a mechanism. If mean lumen size when division rate and cell size have stabilized are similar between cysts, that would support the shift mechanism based on lumen size. Experiments are suggested in Supporting Text S1 to begin identifying potential molecular counterparts to TS and LS ISMA mechanisms.

### Future in silico experiments

Five directions for in silico experiments present themselves. The first two require seeking contradictory or supportive literature evidence of in silico experiments. 1) Exploring the consequences of parameter changes will provide insight into ISMA's mechanism-phenotype relationships for which there may be biological counterparts [1]. A full suite of parameter change experiments was conducted using the LS-ISMA; results are presented in Figure S11. One example is to explore the consequences of changing *deathRateEpi* and *deathRateLumen* (Figures 7 and S11), including setting both to 0. Another is to vary *lumenGrowthRate* to explore the effect of increased or decreased lumen expansion on in silico cystogenesis (see Figure S11). Addition of any of several compounds to the culture media in vitro will stimulate cyst expansion. Examples include cholera toxin and forskolin. 2) Modify axioms and operating principles to simulate targeted mechanistic interventions. One example (see Results) is to modify the way in which cells calculate their axis of division. Another is to modify how matrix is represented in order to explore consequences of altered matrix properties on cystogenesis. Currently, matrix is simply a grid space state. Matrix could be represented using a CPM “cell” that offers resistance to cell advancement. So doing opens the door to exploration of a variety of matrix-cell interactions that could map to proteins altering local matrix properties. 3) Systematically expand the targeted attributes while keeping cells atomic. Movies, such as Video S1 from [9] along with the current literature, contain examples of many behaviors beyond the scope of the current ISMAs. Adding any one of the following to the list of targeted attributes will falsify the current ISMAs. At the cell level: when cells undergo mitosis, they enlarge temporarily and then return to a smaller size; some cells (and cysts) move around during the early stages of cystogenesis; some cells migrate toward each other and cluster together before initiating division; typically, when cells die in contact with matrix, they are flushed into the luminal space where they shrink and disappear. At the cyst level: cysts spin. The process was described in [20] and recently modeled in [24]. Cyst growth may have an additional later stage characterized by significantly slowed expansion, rather than continuing to grow steadily as predicted by the ISMA. The dynamics of lumen merging are more complex than the merging events that occur during simulations. Also, lumens change shape and move within cysts during the initial stages of growth.

4) Increase realism by transforming cells from atomic to composite objects. The axioms used by cells are placeholders for more fine-grained micromechanisms. The latter can be instantiated in future ISMA descendents. Before we can turn our attention to intracellular processes, we need new ISMAs in which cells are composite (and eventually hierarchical) analogues that can achieve essentially the same, targeted SMs as the current ISMAs (Figures 2 and 3). Previous reports [14], [19], [25] explained that an in silico analogue (such as the current ISMA) that quantitatively mimics many cell-level phenomena can be used to begin the sequential process of drilling down and establishing plausible, causal linkages between phenotype and molecular level details. Using cross-model validation procedures, the atomic cell is replaced by a composite cell where phenomenal axioms are replaced by concrete micromechanisms involving interacting objects that map to subcellular processes and/or components in the referent. 5) Once we have the preceding composite cells, we can expand the list of targeted attributes to include subcellular and intracellular behaviors. Alternatively, expanding the list of targeted attributes can require transforming cells from atomic to composite objects. Examples of subcellular and intracellular behaviors include the amount and location of polarization proteins, organelle movement, the organization of the mitotic spindle, formation of a pre-apical patch, location-dependent lipid compartments within the membrane, etc. During cell polarization (as detailed in [8]), PTEN moves to the apical membrane, where it converts PIP3 to PIP2, which binds to Anx2 and assists in the recruitment of Cdc42 to the apical membrane. The task at this stage, while adhering to a strong parsimony guideline, is to add new mechanisms and details that enable validation against the new, targeted attributes, while retaining all of those mechanisms and behaviors that enabled validation during earlier cycles of the IR Protocol. So doing will enable the in silico exploration, falsification, and validation of increasingly complex in vitro MDCK cell behaviors, which will ultimately correlate to in vivo phenotypes of developing epithelial organs.

We hypothesize that the local cause-and-effect relationships (mechanisms) occurring in ISMAs during execution, and thus their morphogenic agenda, have in vitro counterparts. Challenging these alternative hypotheses can be a focus for future in vitro experiments and ISMA refinements.

### Summary

Through careful application of the IR Protocol, analogues of MDCK cystogenesis in cultures (ISMAs) were developed, falsified, refined, and validated against novel, multi-attribute quantitative data. ISMAs were based on software specifications that enabled in silico behaviors during simulation to achieve degrees of validation: to be mapped quantitatively to measures of cystogenesis (targeted attributes). Those specifications also enabled hypothesizing that ISMA operating principles, axioms, components, events, and mechanisms have in vitro counterparts. Predictions of substantive mechanistic changes were verified by independent experiments. ISMAs were used to explore and test hypotheses about cell and cyst dynamics. The above, coupled in vitro and in silico experiments led to four insights. 1) The axis of cell division significantly affects lumen number without changing cell number or cyst size. 2) Reducing the amount of luminal cell death had limited effect on cystogenesis. 3) Later stages of cystogenesis, marked by a decrease in the rate of cell division and cessation of the decrease in mean cell size, can be explained by the presence of a new cell state (called stabilized), which differs in a few key behaviors. 4) The same, multi-attribute phenotype can be a consequence of two fundamentally different mechanisms that, in silico, only alter the mechanism of cell stabilization. By providing a new way of thinking about cystogenesis, ISMA simulations have provided an impetus to explore novel aspects of epithelial morphogenesis.

## Methods

### In vitro methods

A single cell suspension of MDCK cells was plated in duplicate on a layer of 100% Matrigel basement membrane (BD Biosciences) in the presence of 2% Matrigel in the media. Cysts were allowed to grow for the indicated duration then fixed with 4% paraformaldehyde. The cells were then stained as described in [11], [26]. Briefly, cells were stained with a monoclonal antibody against gp135/podocalyxn, and a polyclonal antibody against β-Catenin. F-actin and nuclei were stained with Alexa-labeled phalloidin and Hoechst 33342 respectively. Each day, 20 cysts from the duplicate plates were selected at random and imaged using a Zeiss 510 laser scanning confocal microscope (Carl Zeiss Inc.). Images were acquired sequentially in four separate channels.

Cell number was determined by counting the nuclei, when visible, and actin borders when not. Cyst and lumen perimeter were traced using ImageJ and the size of the cyst and lumen within each cross section was calculated using the analyze tool. Cellular area was found by subtracting lumen area from cyst area; mean cell area was found by dividing cellular area by the number of cells; and the ratio of cellular area to cyst area was found by dividing cellular area by cyst area. Standard deviations and Similarity Measure values (defined in Results) were calculated using R. The number of lumens in each cyst was found by counting the discrete spaces within the cyst bordered by gp135/podocalyxn and actin.

The data generated by the in vitro experiments was quantitatively consistent with results from previous studies [6], [9], [27], as well as being internally consistent. The goal of conducting the in vitro experiments was to provide a particular quantitative perspective on MDCK cystogenesis. We sought an abstract mechanistic explanation of one set of cytogenic trajectories. Repeated in vitro experiments using a different batch of cells could result in distinct cytogenic trajectories, which might not be explained by the current ISMAs. Understanding and simulating such different trajectories is outside the scope of this project.

### ISMA uses

An early task in any modeling effort is to state near- and long-term uses; one must then strive to follow a model development path intended to achieve those uses. When dealing with biology, having explanatory mechanistic models necessarily precedes having predictive mechanistic models. This project is an important, early step in developing explanatory mechanistic models of cystogenesis. A truly useful explanatory mechanistic model is one in which we can observe putative cause-effect events at several layers as they unfold. Given those considerations, we envisioned six near-term ISMA uses. 1) Instantiate and challenge hypotheses about mechanisms of cystogenesis by MDCK cells under different culture conditions. 2) Make it easy to follow mechanistic processes and trace cause-effect relationships. 3) Achieve measures of cystogenesis during ISMA executions of increasingly autonomous cells that are quantitatively similar to referent measures (i.e., they achieve targeted SMs). 4) Achieve increasing overlap of an MDCK cell culture's phenotype by an ISMA phenotype. 5) For validated ISMAs, explore the consequences of mechanistic interventions on measures of cystogenesis. 6) Expose possible gaps in our knowledge of MDCK cell cystogenesis. Implicit in these uses is the ability of ISMA behaviors under different conditions to stand as predictions of MDCK cell and cyst behaviors under comparable conditions.

The preceding are prerequisites for achieving six long-term ISMA uses. 1) Enable replacing ISMA operating principles with concrete mechanisms composed of interacting components. So doing is required to enable hierarchical linkage of molecular level details with specific phenotypic attributes. 2) Execute in silico experiments that test the effect on ISMA cystogenesis of simulated chemical and genetic interventions that affect cell behaviors. 3) Enable continuous refinement of increasingly trustable, complex, biomimetic mechanisms that stand as plausible explanations for increasingly large sets of multi-attribute, multi-source wet-lab data. 4) Represent uncertainty at multiple levels, including uncertainty in mechanistic hypotheses; provide plausible representations of sources of variability in referent data and phenomena. 5) Enable straightforward redeployment and adaptation of ISMA components to represent other cell types and their behaviors; examples include MCF-10A and primary mouse breast organoids. 6) Enable concrete translations between in vitro knowledge and epithelial diseases such as autosomal dominant polycystic kidney disease and cancer.

### In silico methods

Components and mechanisms mapped as closely as possible to components and mechanisms in the referent system. ISMAs were composed of cells, luminal space, and extracellular matrix. We set parameters such as the rate of cell division and the initial size of cells to map to quantities within the in vitro system. Simulation began with 2-4 cells (to mimic the observed number of initial cells in vitro) on a 2D 100×100 hexagonal grid. Cells expanded in size and divided using the CompuCell3D [28] cellular Potts model architecture and customized code. Each cell occupied multiple locations on a hexagonal grid, thus allowing cells to expand, divide, change shape, and move in a realistic manner (Rejniak et al. [16] used an alternative method for enabling cell shape change). We coupled that with features of the agent-oriented modeling approach used successfully by [14], [29]-[31].

**Figure 10:**
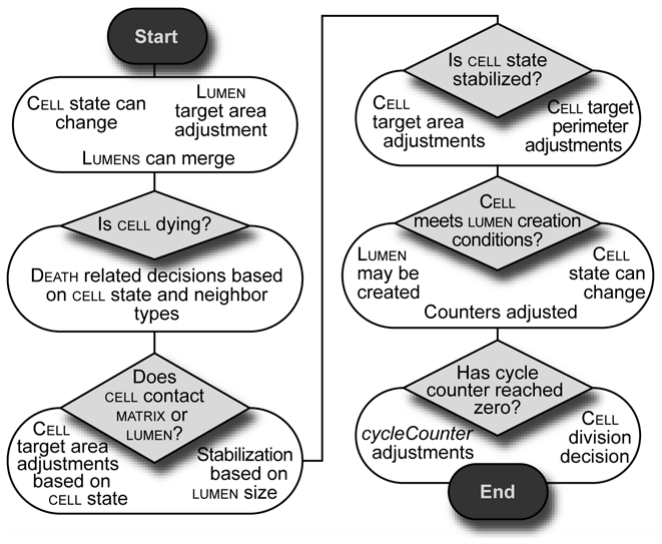
Key features of ISMA logic and decision control flow. During a simulation cycle, each cell steps through five logic modules sequentially to decide which actions to take based on its local environment and internal state. A lumen's target area is adjusted; lumens can merge with each other. Cells that are not dying may begin to do so. Cells adjust their area based on their state and the state of neighboring cells; they stabilize if the lumen has reached a critical size. Cells can create new lumens. Under specified conditions they can divide to form new cells. Future versions of ISMA logic may randomize action control in order to simulate the parallel nature of event occurrence both within MDCK cultures and within each cell. See Figure S12 for complete details of the logic within each of the five modules.

Each cycle, cells stepped through the same decision flow (Figures 10 and S12); they applied the operating principles described below to change shape, divide, change state, create lumens, and die. Logic design and implementation was constrained by the specifications in Table 1. Note that cells are atomic objects: they have no internal parts. All of their micromechanisms are in the form of axioms. Some axioms add behavior variability to ISMAs, as noted in Table S2.

Except as noted, simulations ran using the parameter values in Table 2. A simulated day mapped to an in vitro day and consisted of 48 simulation cycles, equivalent to 30 minutes per cycle. Drawing on several years of prior experience experimenting on MDCK cultures, we specified that when SM1 (defined in Results) >0.5 for nine of ten days, the results can be considered to be within the range of experimental and biological variability. Specifically, when SM1 was achieved, simulation results were taken to be experimentally indistinguishable from values obtained from an independently repeated in vitro experiment. Empirical parameter tuning was used to obtain frequencies of SLSL cysts comparable to that observed in vitro. When SM targets were not achieved, that specific mechanism was falsified. SMs also allowed for ISMA validation and falsification when new attributes were added to the target list (discussed below).

### Iterative Refinement Protocol

The Iterative Refinement Protocol (IR Protocol), described in [14], [19], [25], [29], provided the foundation of our methods. Based on the results of prior experiments and literature review, we selected an initial group of qualitative attributes to target and simulate (the first few in Table 1). We implemented a simple ISMA that reproduced them, thus achieving an initial degree of validation. We then added new data, expanding the set of targeted attributes. So doing falsified the simple analogue. That judgment was based on observation (for qualitative attributes) and values of the prespecified SMs (for quantitative attributes). The manner in which the first analogue was falsified informed us how to develop an improved version that would survive falsification. During subsequent cycles, we added new data or features from Table 1 to the targeted set. So doing often resulted in falsification of the then-current ISMA. On some occasions, it was clear that an incrementally more fine-grained set of mechanisms and/or components would be needed to achieve the specified SMs. On other occasions, we undertook an empirical search of parameter space in search of new sets of parameter values that would reestablish validation. When that search failed, new mechanisms, sometimes more fine-grained, were developed. That iterative process ended with the attributes in Table 1 and the corresponding in silico specifications.

The IR Protocol has a number of benefits. Chief among them is that once an ISMA is validated against targeted data, additional data can be added and the analogue reengineered without invalidating existing mechanisms. The new data will falsify the current ISMA by design, but a successful revision will survive falsification by both new and existing data. Because in silico components and mechanisms map to their in vitro equivalents, it is often the case that only a subset of ISMA components and/or operating principles must be modified to mimic both new and original phenomena. Examples include adding a new cell state and replacing one axiom with two more specific axioms. Because of the networked nature of all mechanistic details, each ISMA change requires some retuning of the parameterizations of several already existing (unmodified) ISMA features.

The IR Protocol consists of the following steps: first, specify a list of targeted attributes, which forms the basis for experimental hypotheses. Devise a specification that maps in silico components and operating principles to cell culture counterparts. The operating principles are expected to enable cells to exhibit behavior that is closely analogous to that observed in vitro. Implement the analogue in code and execute it to deduce predictions about the in silico and in vitro system. As stated in [29], analogue execution is a form of deduction, where the behavior of the analogue follows logically from the premises embodied by its initial conditions and input data. In some cases, this deduction will yield obviously invalid results, which falsifies the current list of operating principles and prompts the modification of mechanistic hypotheses. Once the analogue cannot be falsified by data specific to the current list of targeted attributes, add one or more new, targeted attributes and repeat the IR Protocol.

The process facilitates mechanism exploration, leading toward deeper insight into biological counterparts. Undertaking a series of tightly coupled in silico and in vitro experiments further increases the confidence that the results of ISMA intervention experiments can stand as useful predictions of MDCK counterparts. When there is sufficient ISMA and MDCK cystogenesis similarity, we hypothesize there is corresponding mechanistic similarity. Consequently, results of ISMA intervention experiments will stand as predictions of in vitro phenomena following corresponding in vitro interventions. Some of those predictions will merit in vitro follow-up.

### Agent-oriented approach

An advantage of using targeted attributes and specifications is the flexibility of their implementation. We chose to implement the ISMAs using an agent-oriented approach as explained below and described in [25], but their key aspects include object-orientation, component mapping, spatial orientation, relational grounding and striving for component autonomy. Agent-oriented models are frequently implemented using object-oriented programming techniques, which allow the designer to create individual computational objects corresponding to agents and components within the specification. Components and mechanisms are mapped to analogous components and mechanisms within the referent. So doing makes translating in vitro and in silico observations back and forth more intuitive and less complex. Individual agents can serve as analogues for in vitro components. Agents are quasi-autonomous and they possess their own internal control flow and execute actions independent of enclosing agents. Grounding is defined as the units, dimensions, and/or objects to which a variable or model constituent refers. When grounding is relational, variables, parameters, and I/O are in units defined by other model components. When grounding is absolute, variables, parameters, and I/O are in real-world units like seconds and µg/ml. One advantage of using an agent-oriented approach with relational grounding [25] is that fewer assumptions are required to create or validate the ISMA, and those that are must be clearly specified.

The ISMA contains five agents:

1. The experiment agent calls the MDCK plug-in agent and the Potts agent.
2. The MDCK plug-in agent cycles through cell agents each simulation cycle.
3. The Potts agent executes the index change step: pseudorandom index change attempts and energy calculations.
4. The cell agents change their state and perform other actions.
5. The screenshot agent, called in a separate thread, records a screen shot at the end of the execution of the simulation cycle.

### The cellular Potts model

ISMAs were developed using the CompuCell3D (CC3D) architecture [32], [33], an implementation of the Glazier-Graner-Hogeweg [34] or cellular Potts model (CPM). A CPM “cell” is not limited to a one-to-one correspondence between objects and grid locations. The CPM extends cellular automata so that each grid location contains an index specifying which simulation object contains that location. A CPM with 100 grid locations can contain anywhere from 1 to 100 cells. This modification allows simulations to address cell size, shape change, and cell-cell adhesion. During a simulation cycle, the Potts agent calls a pseudorandom index change algorithm that randomly selects a user-specified number of locations and evaluates whether each will remain indexed to its current cell or change to be indexed to another cell. If the location remains indexed to the current cell, the grid remains unchanged. When a location's index changes, that location and the “energy” of the system are updated.

To calculate whether a location changes index from one cell to another, ΔG is calculated; it is the change in “energy” if that location changes its index to the new cell. An acceptance function generates a probability *p* based on the value of ΔG, and then checks if the pseudorandom number *r*[0,1]<*p*. When *r*<*p*, the change is accepted and the location is assigned to the new cell, and if not the change is rejected. When accepted, the energy of the system changes.

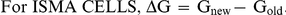



It calculates the value of G_new_ and G_old_ using a Hamiltonian equation: 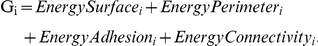



Each of these terms is calculated through a separate equation, detailed below.

### Surface area and perimeter

The energy calculation for *EnergySurface* depends on *LambdaArea* (λ_A_) and the difference between the target surface area (TA) and the current surface area (A):

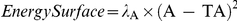



The larger *LambdaArea* is the more changes in TA will affect the overall energy of the system and the faster these changes will be reconciled. *LambdaArea* for cells is a user-set parameter, while for lumen it is fixed at 20 to represent the large outward force of the expanding lumen.

The calculation of *EnergyPerimeter* is similar:

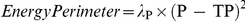



### Adhesion, connectivity, and tight junctions

The “energy” of adhesion depends on the cell type and its location. For location (i, j), the energy is the sum of values calculated between (i, j) and all neighboring points residing in separate cells. If, for example, two of the six neighboring points reside in another cell, then the energy of adhesion would be 2·X_1–2_, where X_1–2_ is a parameter controlling the adhesion energy between cells of type 1 and type 2. Separate adhesion energy parameters are specified for each pair of cell types (Table S3).

The “energy” of connectivity is generally 0, but if changing the cell index of a location results in a location being isolated from the rest of the cell, an energy penalty is assessed by setting *EnergyConnectivity* to be very large. As a result, cells cannot split into pieces except when they undergo cell division.

In addition to maintaining connectivity between all points in a cell, an ISMA maintains integrity between tight junctions, preventing them from being remodeled in the index change step during a simulation cycle. If the ISMA detects that the change in a point would result in a tight junction being remodeled, it assesses an energy penalty by setting *EnergyConnectivity_i_* to be very large. A detailed explanation of tight junction remodeling is provided in Text S1.

### CompuCell3D and custom code

**Figure 11:**
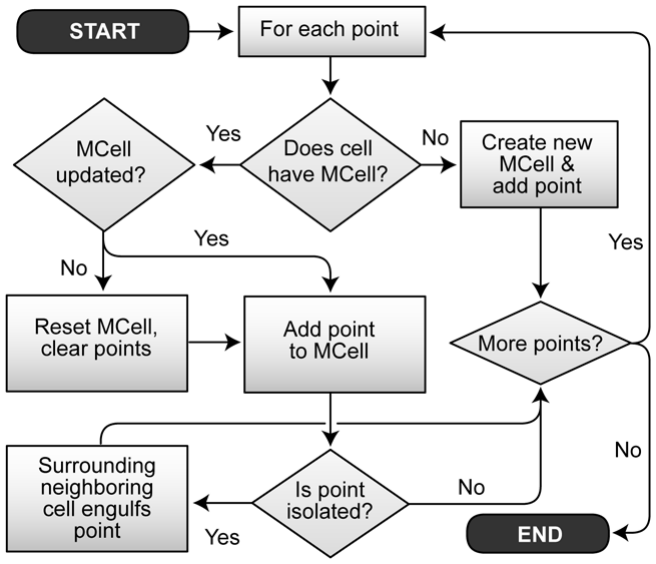
*MCell* point assignment flow chart. An *MCell* point has no specific cystogenesis counterpart. Once per simulation cycle, each point is assigned to the *MCell* agent associated with the cell enclosing that point. *MCell* point lists are initialized during each simulation cycle. Additionally, surrounding cells engulf isolated points.

CC3D is designed from a system-based perspective. Each simulation cycle, each aspect of the system is executed, from the index change step that selects random points, to the plug-ins that update aspects of the system. CC3D was not designed from an agent-oriented perspective, so it was necessary to expand it to gain required capabilities. *MCell* objects were added to cell objects to create a bi-directional mapping between individual points and the cells that contained them. These objects and their control flow were executed in sequence by the MDCK plug-in to grant full agency to cells, which previously only executed after a location within the cell boundary changed its index. Every simulation cycle all points in the grid are surveyed to assess which cell they are indexed to and a reference is stored in an *MCell* object corresponding to that cell, as shown in Figure 11. Thereafter that *MCell* can be queried to find out what points are located within its corresponding cell object.

The version of CompuCell3D used to develop this project has been superseded (see Text S1) by the current available version. The capabilities provided by the current version were not judged necessary for the ISMA, especially due to the significant addition of custom code. The project was not adapted to the updated version.

### Cells compute their target size using a value of ideal area

As shown in Results, we observed that prior to cells stabilizing in vitro, their size correlated with the size of the cyst and its cell number. We hypothesized that operation of yet-to-be identified micromechanisms provides each cell with a target size. We speculated that a cell might use information such as the tension between it and neighboring cells, lumen pressure, and the ratio of lumen and matrix contact area in order to update its target size. To mimic the decrease in mean cell area observed in vitro, we developed and used an algorithm that is a placeholder for yet-to-be-designed, concrete micromechanisms that can be implemented in a future ISMA. Each individual cell adjusted its size and shape so that a target area W, the projected wedge area (a wedge that includes the portion of the perimeter in contact with matrix and terminates at the cyst center), would move toward or equal an ideal value. The parameter *wedgeArea* was a value based on the early (pre-stabilization) 169 µm^2^ area observed in vitro. An ISMA calculated W using the following formula:



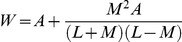



A is the area of the cell, M is one-half the number in cell grid edges in contact with matrix, and L is one-half the number in cell grid edges in contact with lumen. This formula assumes that cysts are somewhat circular. Variations in actual cell size caused by non-circular cysts resulted in variance in cell area similar to that observed in vitro. The cell subtracted W from *wedgeArea* and set its target change in area to the resulting value (with a final maximum value of *wedgeArea*). Use of this algorithm during early simulation cycles caused mean cell area to decrease and cyst area to increase, mimicking observed in vitro data (Figure 2C).

Once cells stabilized, they no longer used the above equation. Instead, cells strove to maintain an area that increased only slightly as contact with the lumen increased. We speculated that cells within cysts in vitro must maintain a minimal cell height even as they are stretched by the expanding lumen. We specified that ISMAs use a similar guideline.

### Cells compute a target perimeter

From in vitro observations, it seems likely that cells have genetic and environmentally imposed targets for the areas occupied by different surfaces (cell-cell interfaces, basal, and apical). We specified that 2D cells have a target perimeter value (TP) that is computed using the cell's current area. For simplicity, we specified that a cell compute TP using the perimeter P of a circle having an area A equal to its own:
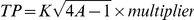



K is a scaling factor and *multiplier* is user-specified. Two cells having identical areas will have identical TP values, so if one has a larger P the difference between P and TP will also be larger, causing that cell to move toward circularity faster.

### Cell polarization and stabilization

The value of *polarCounter* was set to equal a pseudorandom value *r*[*polarDelay* · 0.75, *polarDelay* · 1.25] when a cell first contacted matrix. Thereafter, it decreased by one each simulation cycle. Upon reaching 0, cell state changed from unpolarized to polarized. Consequently, *polarCounter* is the cell's counterpart to a cell, having established matrix contact, changing and moving around its components in a process that ends when tight junctions have formed and the apical surface is isolated and complete.

A correlation was observed between mean cell size and the rate of cell division in vitro, but a causal link was not apparent. Individual cells may sense the area of matrix contact in part through β1-integrin signaling [26]. They may sense the area of lateral cell-cell contact in part using catenins and cadherins [35]. That information may influence whether a cell divides or not. As stated in Discussion, tension transduced by the subapical F-actin network could allow cells to sense the size of the lumen. Such information supported our decision to use lumen size as a signal for cell stabilization. Each simulation cycle, a cell bordering matrix and lumen queried the lumen for its size. When that value ÷ 1000 was greater than the parameter *stableRatio*, the cell changed to the stabilized state.

### Cell division

**Figure 12:**
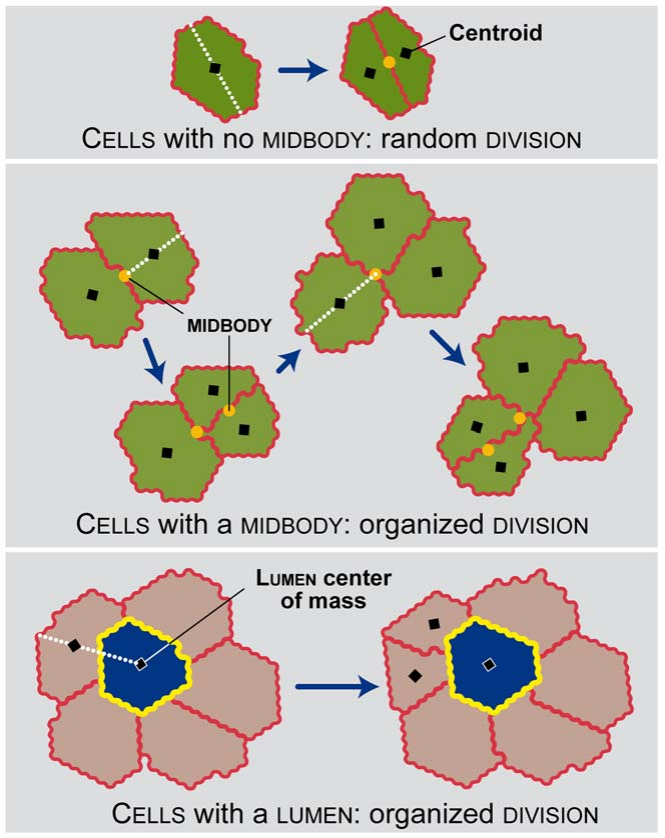
ISMA cell division. Cell division depends on the cell neighborhood. Single isolated cells (top) that have not divided have no midbody and divide with a random axis of division. When cells divide they find their centroid and store it as the midbody of their daughter cells. Cells that have previously divided and have a midbody utilize it for subsequent divisions. For these organized divisions (top), the axis of division is determined by a line drawn from a cell's current centroid to the stored midbody. Cells in contact with a lumen will also divide in an organized fashion (bottom), using a line between their centroid and that of the lumen to determine the axis of division. When the axis of division is determined, all points on one side of the line are assigned to a new cell while all points on the other remain assigned to the original cell.

Decrementing *cycleCounter* is a cell's counterpart to moving through the phases of the cell cycle. *CycleCounter* is a variable that is initialized based on *cellCycle* (a user-specified parameter that controls the duration of the cell cycle) and decremented thereafter. Cells implemented the following method of cell division. For the first cell and for daughter cells after division, the value of *cycleCounter* was set to a pseudorandom value *r*[0.75·*cellCycle*, 1.25·*cellCycle*] and then decremented in each cell in every simulation cycle in which the cell had an area > *doublingArea*/2. When *cycleCounter* reached zero, a cell divided (Figure 12), splitting its area in half on an axis, and using the parameter *divisionReg* to determine the method of calculating the axis of division.

When *divisionReg*  =  0, cells chose the axis of division randomly. If it was 1, cells used oriented division, finding their axis of division as shown in Figure 12. Cells recorded the location of their midbody as a point. When dividing, the cell connected the midbody and the centroid with a line. The cell assigned all points above the line to a new cell and all points below to the old cell. It then set the midbody of the both cells to the centroid of the just-divided cell. When *divisionReg* =  2, cells divided randomly until they reached the polarized state and then used oriented division. *DivisionReg*  =  3 specified reversed division, where cells would find the axis of division as stated above, but then add 90 degrees, reversing division orientation.

After a cell divided, the value of *cycleCounter* for both daughter cells was reset to a new random value as detailed above. Its value of *polarCounter* did not change. The new cell inherited all values from the parent cell, except *polarCounter*, which was set to *r*[0.5·*polarDelay*, 1.5·*polarDelay*] – *polarDelay* + *polarCounter*(parent). So doing made the newly created cell have a *polarCounter* value close to but not identical to that of the parent cell.

### Cell clustering and cell death

In vitro data analysis revealed that when the cultures began growing, the mean in vitro cell number was about 2 (Table S4), indicating that a small amount of clustering took place after the cells were plated. That was expected because in Matrigel culture suspended cells settle on the layer of 100% Matrigel and thus most cysts grow in the same plane. Accordingly, simulations began with a single cell, but at simulation cycle 1, the *cycleCounter* of that cell was reduced to 1, causing it to divide during the following simulation cycle. In addition, since in vitro cells are not always at the beginning of their cell cycle when plated, the value of *cycleCounter* for the two cells was changed to equal a pseudorandom number *r*[(1 – *clusterProb*) x *cellCycle*, *cellCycle*]. So doing allowed the amount of clustering to be increased without changing the cell division rate, simply by increasing *clusterProb.*

Cell death is an important factor in MDCK cystogenesis. However, it is not clear that it is required for cyst formation. In order to validate that cysts did not ignore or excessively rely on cell death for normal lumen formation, the amount of cell death observed in silico was quantified and compared to that observed in vitro. In vitro analysis of cell death was conducted in [9]: MDCK cysts were cultured as in this report and fixed and stained with an antibody for activated caspase-3 (cleaved in apoptotic cells).

Within the ISMA, a cell began dying when a pseudorandom number *r*[0, 1] was less than *deathRateEpi* if the cell contacted matrix or *deathRateLumen* if it did not. Once a cell entered the dying state it shrank until its area reached zero. It was then removed from the simulation. Each day, the number of cysts with dying cells was recorded and the percentage calculated (Figure 5). The data was separated based on whether cells were in contact with the matrix or not.

Drawing on literature evidence [36]-[38] and expert opinion we estimated the average time between apoptotic bodies first being visible and a dying cell breaking up into pieces to be roughly five hours. The value of the parameter *dyingShrinkRate* specified the amount that the TA of a dying cell was lowered each simulation cycle (Table 2). Mean dying time ranged from 6.5 to 13 simulation cycles, with an overall mean value of 9.2, which maps to 4.6 hours when one simulation cycle is grounded to 30 minutes.

### Lumens and their creation

Polarized cells create a new lumen when two conditions are met. 1) The cell contacts matrix, but is not in contact with an existing lumen. 2) The location chosen for lumen creation is adjacent to another polarized cell also not in contact with an existing lumen. The point chosen for lumen creation is the cell's midbody (Figure 12), which was the centroid of the parent cell that previously divided to create the current cell.

Lumen formation involves cells creating and secreting fluid. Having cells create and release units of lumen content could simulate that. One unit could correspond to a single grid space. Those units could merge with other units or with an existing lumen object. However, absent validation evidence for the other ISMA mechanisms, implementing such a fine-grained (somewhat complicated, multi-parameter) mechanism simply because it seems biomimetic would have been contrary to the IR Protocol's strong parsimony guideline. We took advantage of CC3D capabilities and elected to use a more abstract, simpler approach. There is no disadvantage in doing so because a strength of this class of analogues is that a simple mechanism that achieves a degree of validation can later be replaced with a more detailed and realistic counterpart. Using cross-model validation [25], this can be done without compromising other ISMA mechanisms that have also achieved degrees of validation [14].

Within ISMAs, lumens are a different class of “cell” object. Their only action options are to expand and merge. After a lumen is created, it expands using the following axiom.

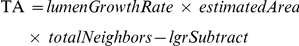



*LumenGrowthRate* is a user-specified parameter; *estimatedArea* is the area of cells in contact with the lumen added to the lumen's area; *totalNeighbors* is the number of cells in contact with the lumen; and *lgrSubtract* is a quantity based on a user-specified parameter and the degree cells are stretched. Cells that are more stretched have a higher *lgrSubtract* value, reducing the rate of lumen expansion. A lumen does not have a target perimeter value—its perimeter is determined entirely by the perimeter of the cells surrounding it. Lumens can merge when their tight junctions are reorganized.

### Tight junction maintenance

Tight junctions (TJs) were implemented in order to simulate aspects of MDCK lumen expansion. TJs exist where two cells contact each other and a lumen. A TJ is two points—one in each neighboring cell—adjacent to a point within a lumen (see and Figure S13). TJs control lumen expansion and merging and prevent cells from contacting multiple lumens. At the end of a simulation cycle, when a TJ is adjacent to a different TJ, the TJs are reorganized and the index of the two TJ points is transferred to the neighboring lumen. Then the two lumens, now in contact with each other, merge together. In addition, TJs can reorganize to allow lumen expansion. To do so, at the end of a simulation cycle all points within TJs execute the following algorithm. A TJ point first surveys its neighboring points to verify they are not in contact with another lumen and that they are not in the matrix or within an unpolarized cell. It then determines if any of its neighboring points are in different cells but are not also TJs. To reorganize, the TJ point computes the free energy change if its index changes to the neighboring lumen and then uses the acceptance function to accept or reject that change. If the change is accepted, the TJ becomes lumen, and the neighboring point becomes a new TJ.

### Scaling observations from 2D to 3D

We recorded aspects of in vitro cyst growth by obtaining cross-sectional images taken through the center of cysts. These images were necessarily a 2D representation of a 3D structure. Based on the symmetry observed within the cross section, in addition to separate analysis of 3D structures, we believe that cysts were roughly symmetrical in 3D. Using this information, we extrapolated 3D values for total cell number, cyst volume, and lumen volume from the measured values of cross-sectional cell number, cyst area, and lumen area. We found that the trends observed for cell number and mean cell area held when the system was projected into 3D. If future targeted attributes required specific modeling in 3D, we could take advantage of the 3D capabilities of CompuCell3D, addressing considerations raised in [39].

### Data storage

The in silico system recorded data about cells and cysts into a MySQL database as specified within Text S1.

## Supporting Information

Figure S1 Cystogenesis measures for TS ISMA. Experiments followed the same experimental design as described in the text. Measures (red) were taken during cystogenesis. In vitro data are provided (blue) for comparison. Designations and symbols are the same as in Figure 2. TS ISMA used the parameter values in Table 2, except for *stableRatio*, which was set to 1000 and *shiftDelay*, which was set to 200.
(TIF)

Figure S2 Percent of cysts with different numbers of lumens for TS ISMA. The experiments are the same as in Figure S1. Designations and symbols are the same as in Figure 3.
(TIF)

Figure S3 Percentage of cysts with dying cells when *dyingShrinkRate* was reduced. (A) In vitro data reproduced from [9]. (B) ISMA data from 50 cysts over ten days using parameter settings from Table 2, except for *dyingShrinkRate*, which was changed from 9 to 4.5. Blue bars: percentage of cysts observed to have apoptotic cells without matrix contact. Red bars: percentage of cysts observed to have apoptotic cells with matrix contact.
(TIF)

Figure S4 Cystogenesis measures when the axis of cell division is random. Experiments followed the same design as in Figure S1. Measures, designations, and symbols are also the same as in Figure S1. LS ISMAs used the parameter values in Table 2, except for *divisionReg*, which was set to 0.
(TIF)

Figure S5 Cystogenesis measures when the axis of cell division is reversed. Experiments followed the same design as in Figure S1. Measures, designations, and symbols are also the same as for Figure S1. LS ISMAs used the parameter values in Table 2, except for *divisionReg*, which was set to 3.
(TIF)

Figure S6 Cystogenesis measures with no luminal cell death. Experiments followed the same design as in Figure S1. Measures, designations, and symbols are the same as for Figure S1. LS ISMAs used the parameter values in Table 2, except for *deathRateLumen*, which was set to 0.
(TIF)

Figure S7 Percent of cysts with different numbers of lumens with no luminal cell death. The experiments are the same as in Figure S4. Designations and symbols are the same as in Figure 3.
(TIF)

Figure S8 Cystogenesis measures when cell polarization was delayed. Experiments followed the same design as in Figure S1. Measures, designations, and symbols are also the same as for Figure S1. LS ISMAs used the parameter values in Table 2, except for cell polarization, which was delayed as described in the text.
(TIF)

Figure S9 Cystogenesis measures for GM ISMA. Experiments followed the same design as in Figure S1 except that GM ISMAs were used. Measures, designations, and symbols are also the same as for Figure S1. Top: note the large variances after day 5.
(TIF)

Figure S10 Percent of cysts with different numbers of lumens for GM ISMA. The experiments are the same as in Figure S9. Designations and symbols are the same as in Figure 3.
(TIF)

Figure S11 Parameter sweeping results. Experiments followed the same design as in Figures 2 and 3. Designations are the same as in Figures 2 and 3. Parameters changed from settings in Table 2 are listed within each Figure. S11-1 to S11-5: TS ISMA. S11-6 through S11-80 used the LS ISMA. Except for S11-1 to S11-5, all parameters were fixed except the single parameter being varied. S11-6 to S11-9: varied *wedgeArea.* S11-10 to S11-13: varied *lambdaArea*. S11-14 to S11-17: varied *stableTargetArea*. S11-18 to S11-23: varied *cellCycle*. S11-24 to S11-27: varied *stableCycleDelay*. S11-28 to S11-31: varied *lambdaPerim*. S11-32 to S11-35: varied *polarDelay*. S11-36 to S11-39: varied *shiftDelay* with high *stableRatio*. S11-40 to S11-44: varied *lgrSubtract*. S11-45 to S11-48: varied *doublingArea.* S11-49 to S11-52: varied *multiplier*. S11-53 to S11-60: varied *lumenGrowthRate.* S11-61 to S11-64: varied *deathRateLumen*. S11-65 to S11-68: varied *deathRateEpi*. S11-69 to S11-72: varied *dyingShrinkRate*. S11-73 to S11-76: varied *clusterProb*. S11-77 to S11-80: varied *stableRatio*.
(PDF)

Figure S12 Full ISMA logic and control flow. Shown are the details of the five components of Figure 10.
(TIF)

Figure S13 Tight junction reorganization. Tight junctions (TJs) prevent cells from contacting multiple lumens. A) TJ counting when a point is within a cell (left) or lumen (right). B) During the index change step, index changes that result in a different number of TJs before and after the change will be rejected. C) When pairs of TJs are adjacent and meet requirements, lumens will merge together. D) TJ reorganization cannot occur if it will result in a cell contacting multiple lumens. E) Allowed TJ reorganization results in lumen expansion.
(TIF)

Table S1 SM2 values for the ISMA. An SM2 value is the absolute value of the coefficient of variance (for a specific measure) subtracted from the in vitro coefficient of variance. Values over 0.25 (black) did not achieve the validation target described in the text. Values between 0.15 and 0.25 (gray) achieved the moderate validation target. Values less than 0.15 (white) achieved the strong validation target.
(DOC)

Table S2 Sources of stochasticity within ISMAs. The listed events or variable assignments provide behavior variability during cystogenesis. Note that index changes, which are random, contribute to many cell level events, including shape change and lumen expansion.
(DOC)

Table S3 Additional ISMA parameters. Parameters used for the ISMA system and for the adhesion plug-in are listed along with descriptions, default values used for simulation, and parameter ranges that are expected to give normal results.
(DOC)

Table S4 Mean cell number per day for cysts grown in Matrigel. Numbers in bold italic are measured, mean values and non-bold numbers are projected values. Projected values were found by multiplying or dividing the measured mean values by the scaling factor of 1.56. During the first four days of growth, the number of cells increased by a constant factor of 1.4 to 1.8 per day, with a value of 1.56 minimizing the percent error between projected and measured mean values. Using that scaling factor, the number of cells at day 0 was estimated to be 2.1, indicating that some clustering took place within the Matrigel culture. To reflect this observation, ISMAs implemented cell clustering.
(DOC)

Text S1 Supporting methods.
(PDF)

Video S1 ISMA time-lapse movie of typical cyst development. This experiment followed the experimental design described in the text and used Table 2 parameter settings. Cells begin to polarize at 0:07, two lumens form by 0:11 and merge at 0:15. Sample cell death observed between 0:35 and 0:38. Cell stabilization occurs at 0:39. Frame rate: 6 simulation cycles per second.
(MOV)

Video S2 ISMA time-lapse movie of multiple lumen formation. This experiment also followed the experimental design described in the text and used Table 2 parameter settings. Cell polarization begins at 0:08 and lumen formation occurs at 0:09. (Note cells without lumen contact near the top of the cyst from 0:09 to 0:34.) Second and third lumens form at 0:35 and 0:36, delaying stabilization of neighboring cells. Most cells stabilize at 0:42, while cells not in contact with primary lumen stabilize at 0:44. Lumens merge at 1:08 and 1:09. Frame rate: 6 simulation cycles per second.
(MOV)
