# Supplementary figures and images for: MDCK Cystogenesis Driven by Cell Stabilization within Computational Analogues
Source: PLoS Comput Biol. 2011 Apr 7;7(4):e1002030. doi: 10.1371/journal.pcbi.1002030 (PMC3072361; doi:10.1371/journal.pcbi.1002030)

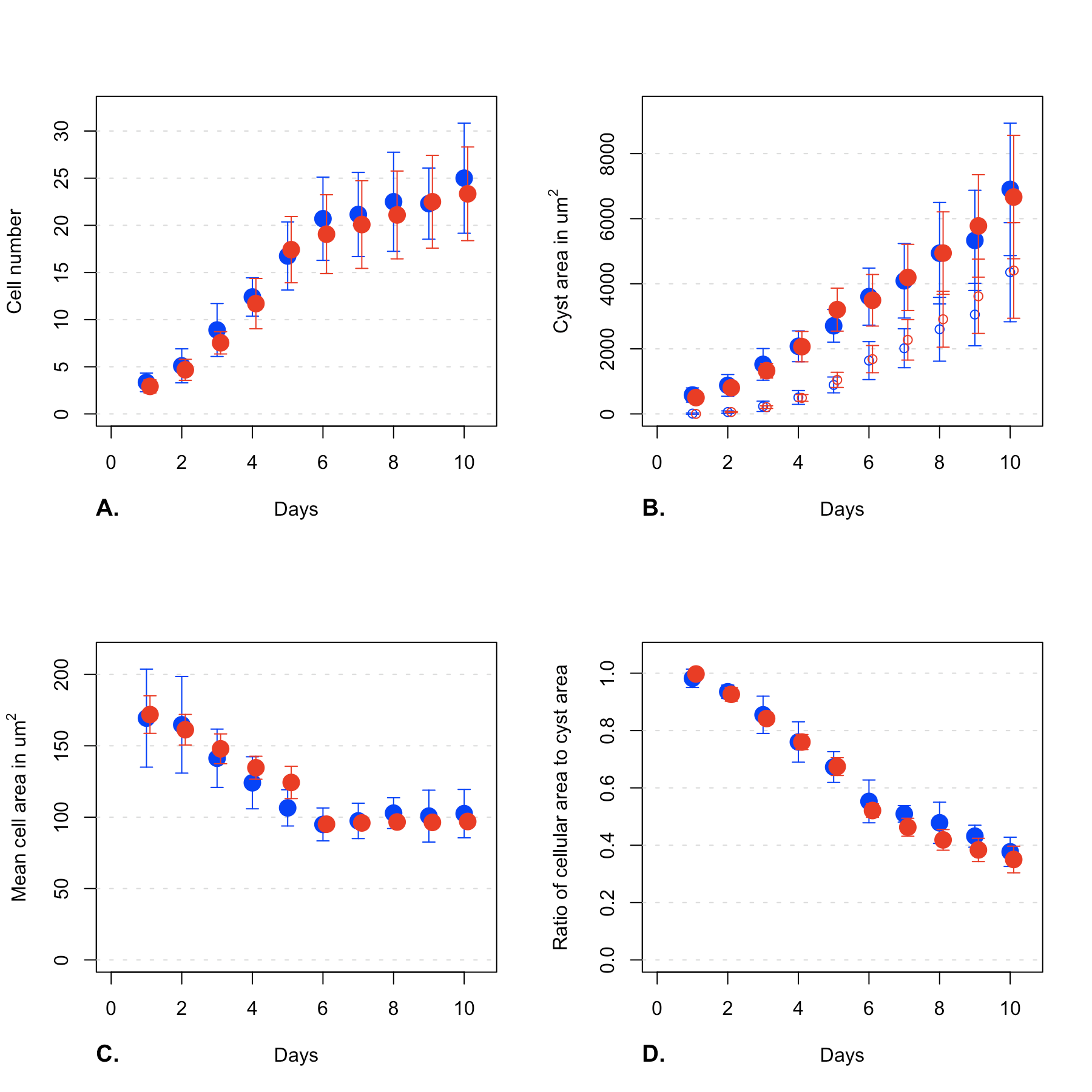

Supplement: Figure S1 — Cystogenesis measures for TS ISMA. Experiments followed the same experimental design as described in the text. Measures (red) were taken during cystogenesis. In vitro data are provided (blue) for comparison. Designations and symbols are the same as in Figure 2. TS ISMA used the parameter values in Table 2, except for stableRatio, which was set to 1000 and shiftDelay, which was set to 200. (TIF) [file pcbi.1002030.s001.tif]

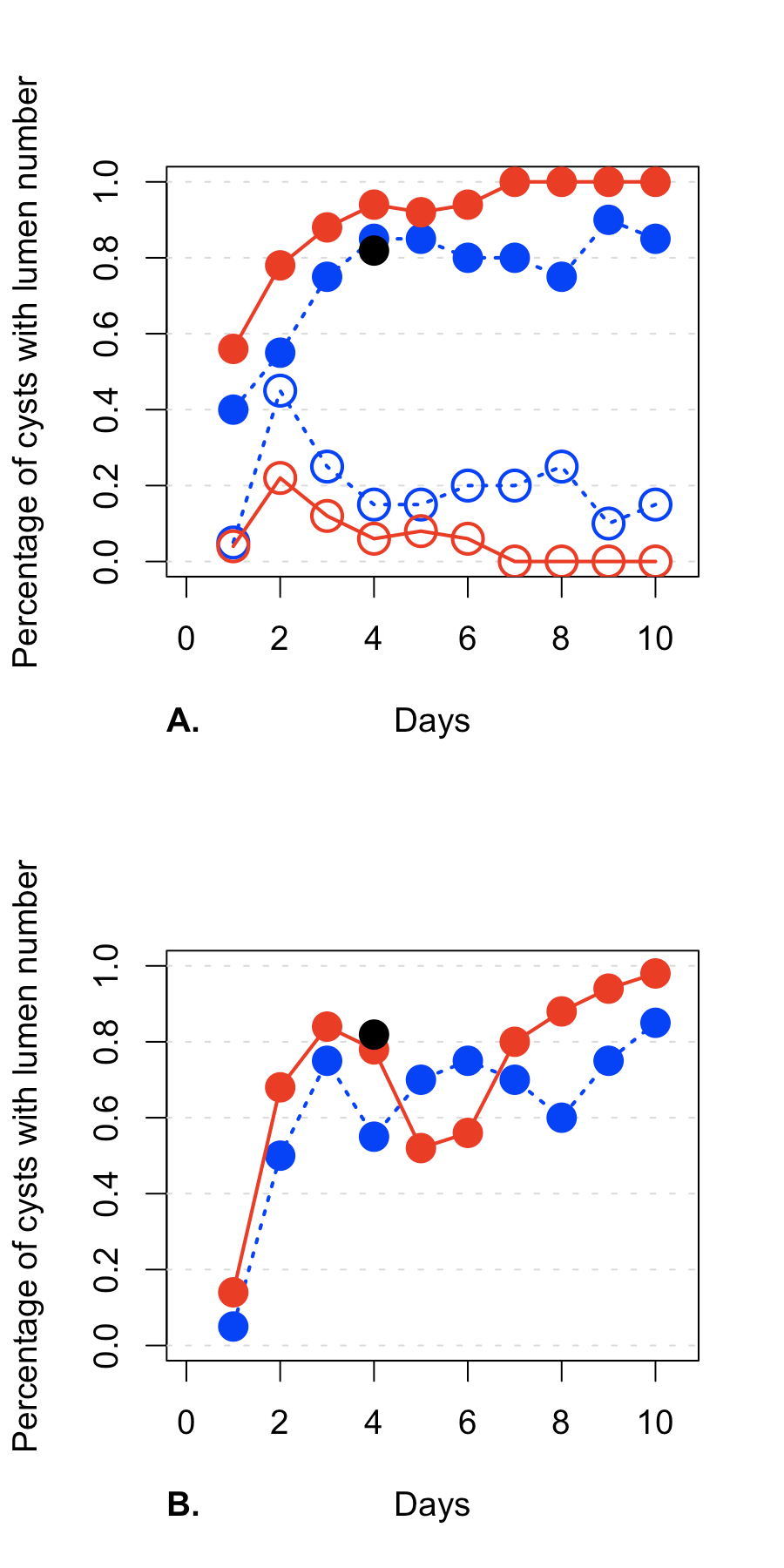

Supplement: Figure S2 — Percent of cysts with different numbers of lumens for TS ISMA. The experiments are the same as in Figure S1. Designations and symbols are the same as in Figure 3. (TIF) [file pcbi.1002030.s002.tif]

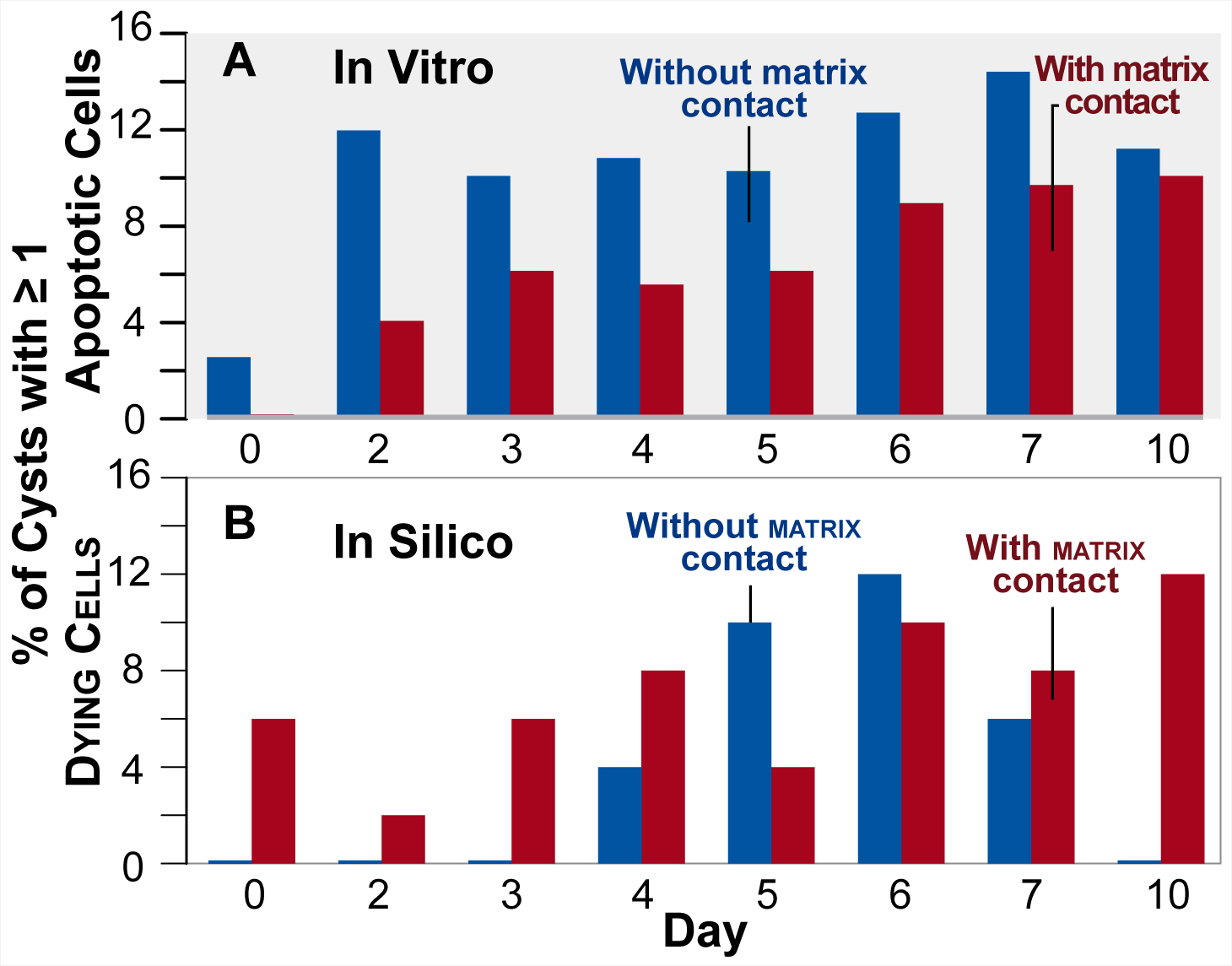

Supplement: Figure S3 — Percentage of cysts with dying cells when dyingShrinkRate was reduced. (A) In vitro data reproduced from [9]. (B) ISMA data from 50 cysts over ten days using parameter settings from Table 2, except for dyingShrinkRate, which was changed from 9 to 4.5. Blue bars: percentage of cysts observed to have apoptotic cells without matrix contact. Red bars: percentage of cysts observed to have apoptotic cells with matrix contact. (TIF) [file pcbi.1002030.s003.tif]

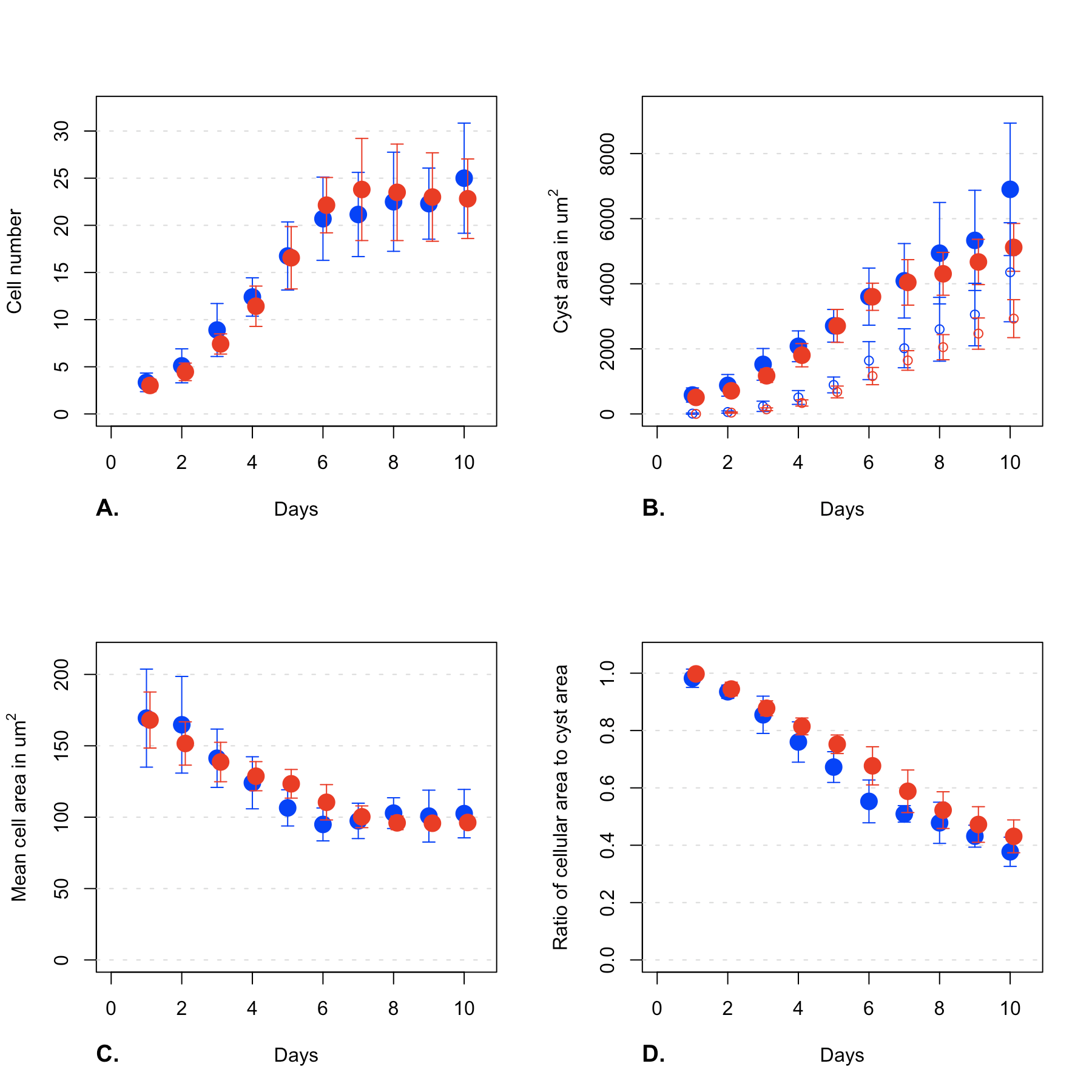

Supplement: Figure S4 — Cystogenesis measures when the axis of cell division is random. Experiments followed the same design as in Figure S1. Measures, designations, and symbols are also the same as in Figure S1. LS ISMAs used the parameter values in Table 2, except for divisionReg, which was set to 0. (TIF) [file pcbi.1002030.s004.tif]

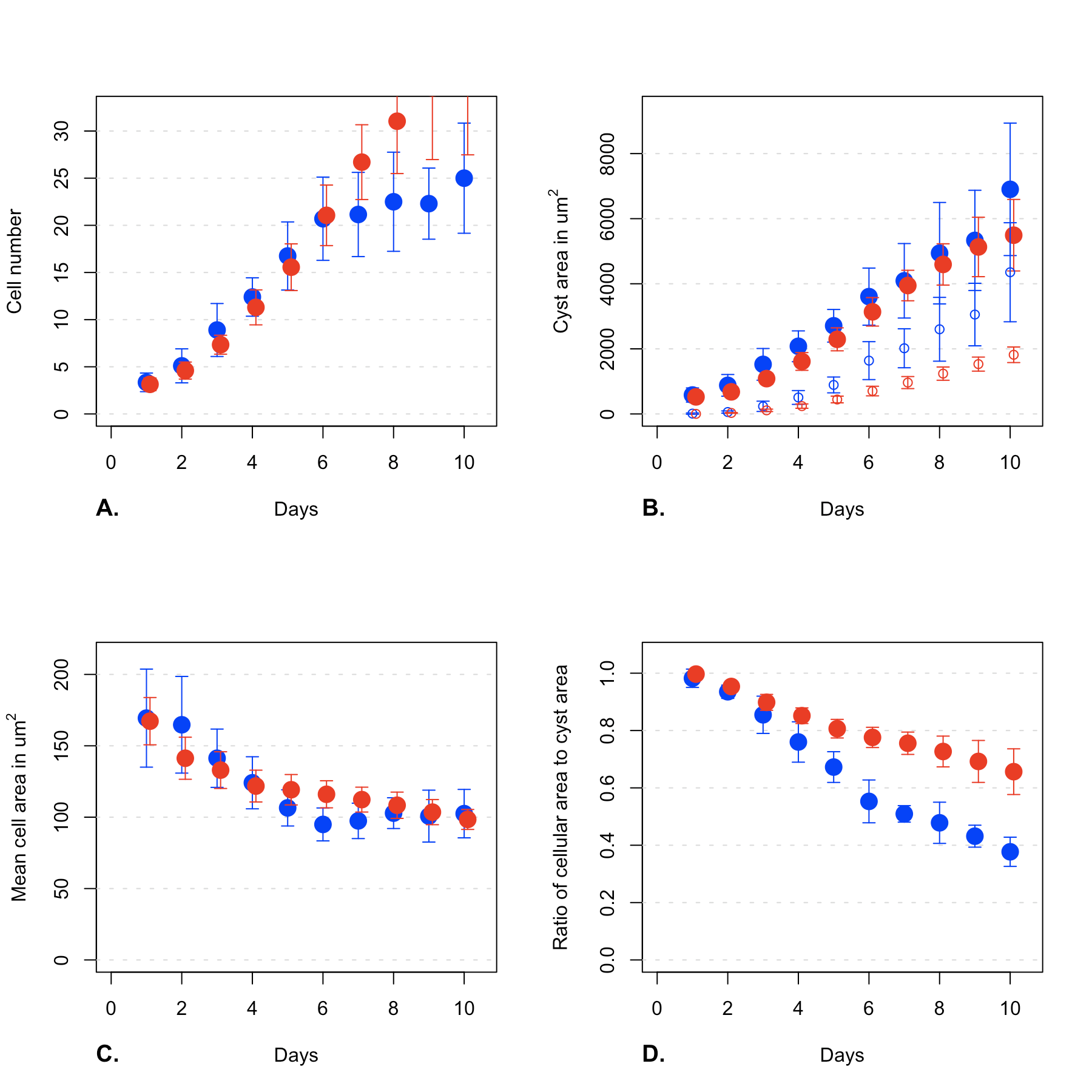

Supplement: Figure S5 — Cystogenesis measures when the axis of cell division is reversed. Experiments followed the same design as in Figure S1. Measures, designations, and symbols are also the same as for Figure S1. LS ISMAs used the parameter values in Table 2, except for divisionReg, which was set to 3. (TIF) [file pcbi.1002030.s005.tif]

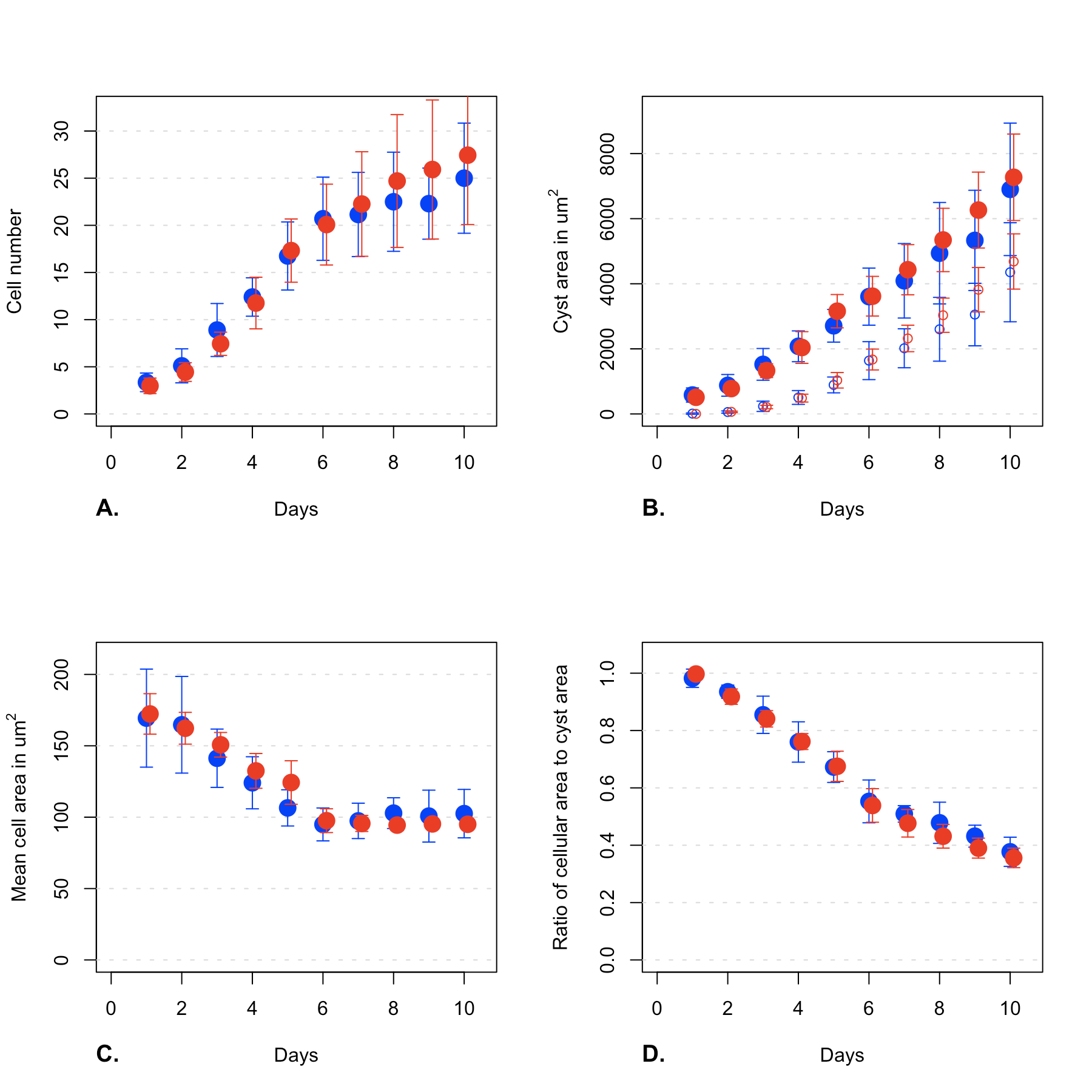

Supplement: Figure S6 — Cystogenesis measures with no luminal cell death. Experiments followed the same design as in Figure S1. Measures, designations, and symbols are the same as for Figure S1. LS ISMAs used the parameter values in Table 2, except for deathRateLumen, which was set to 0. (TIF) [file pcbi.1002030.s006.tif]

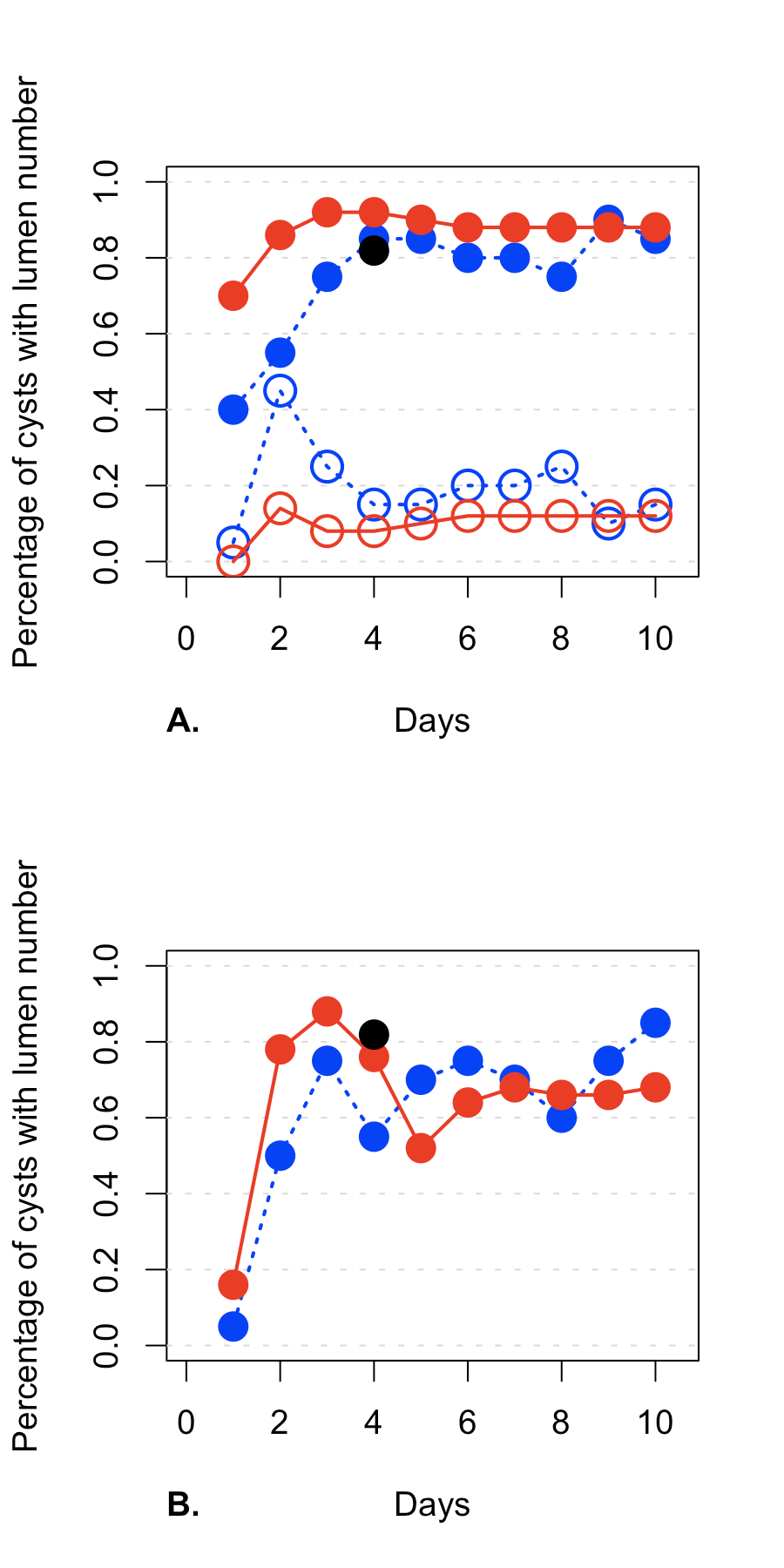

Supplement: Figure S7 — Percent of cysts with different numbers of lumens with no luminal cell death. The experiments are the same as in Figure S4. Designations and symbols are the same as in Figure 3. (TIF) [file pcbi.1002030.s007.tif]

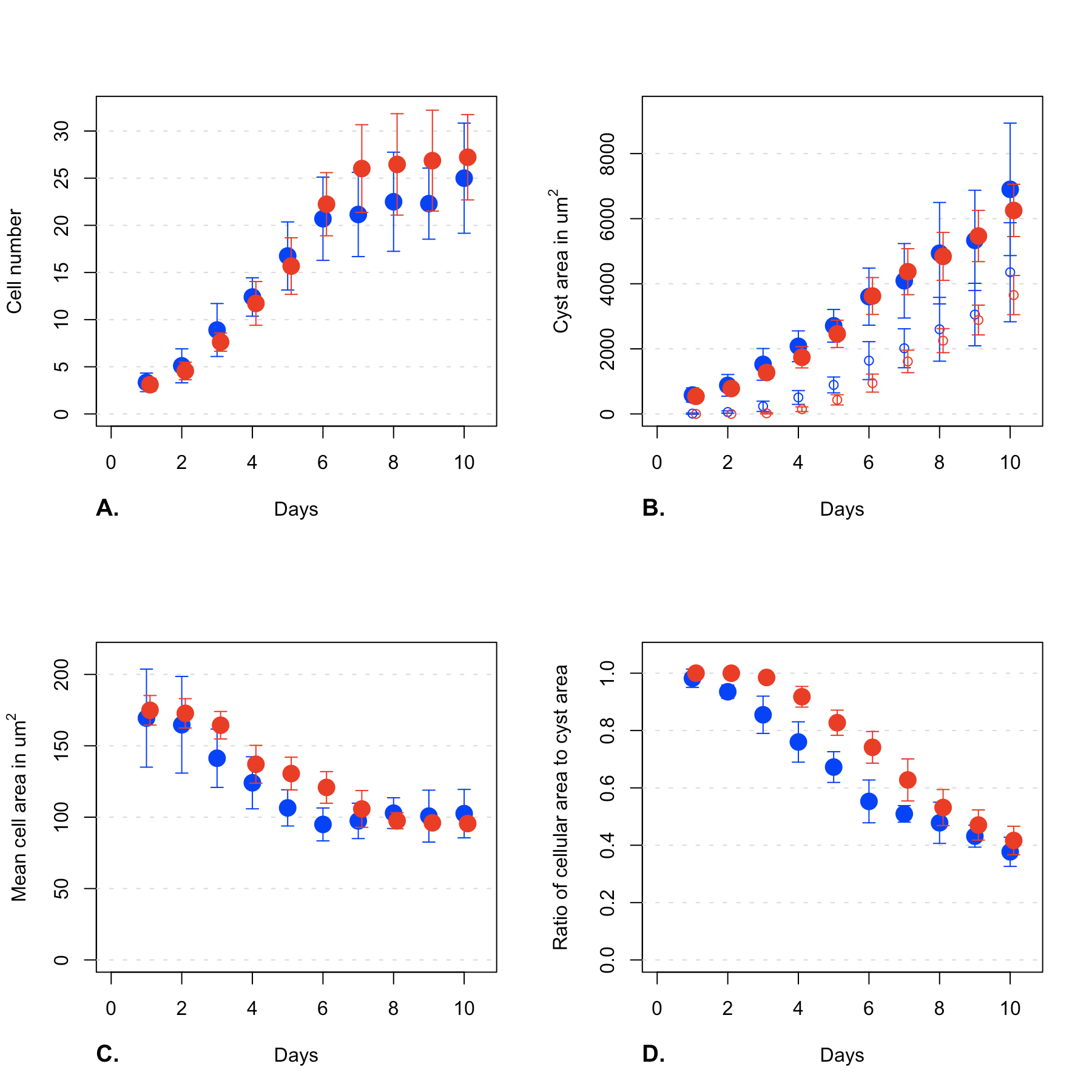

Supplement: Figure S8 — Cystogenesis measures when cell polarization was delayed. Experiments followed the same design as in Figure S1. Measures, designations, and symbols are also the same as for Figure S1. LS ISMAs used the parameter values in Table 2, except for cell polarization, which was delayed as described in the text. (TIF) [file pcbi.1002030.s008.tif]

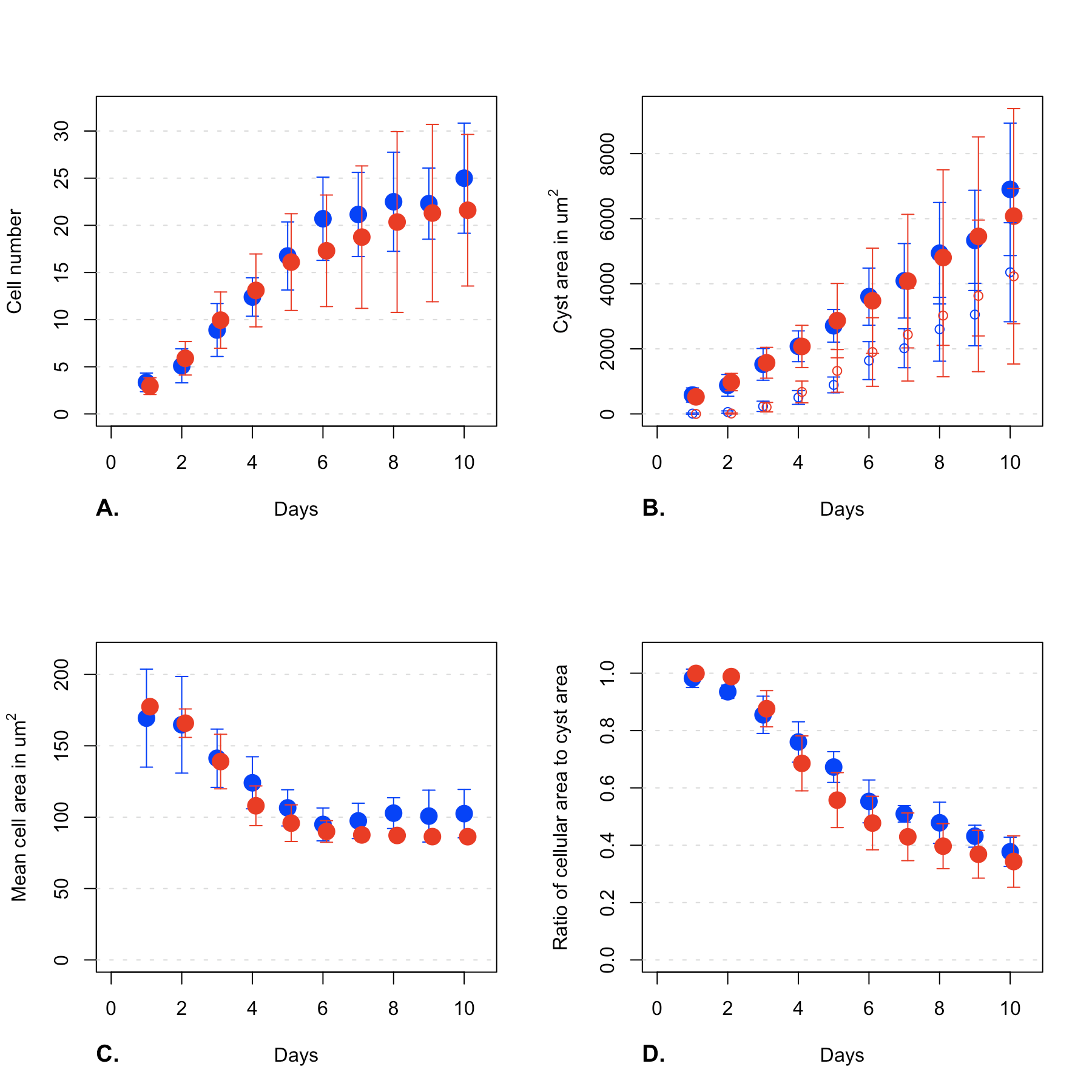

Supplement: Figure S9 — Cystogenesis measures for GM ISMA. Experiments followed the same design as in Figure S1 except that GM ISMAs were used. Measures, designations, and symbols are also the same as for Figure S1. Top: note the large variances after day 5. (TIF) [file pcbi.1002030.s009.tif]

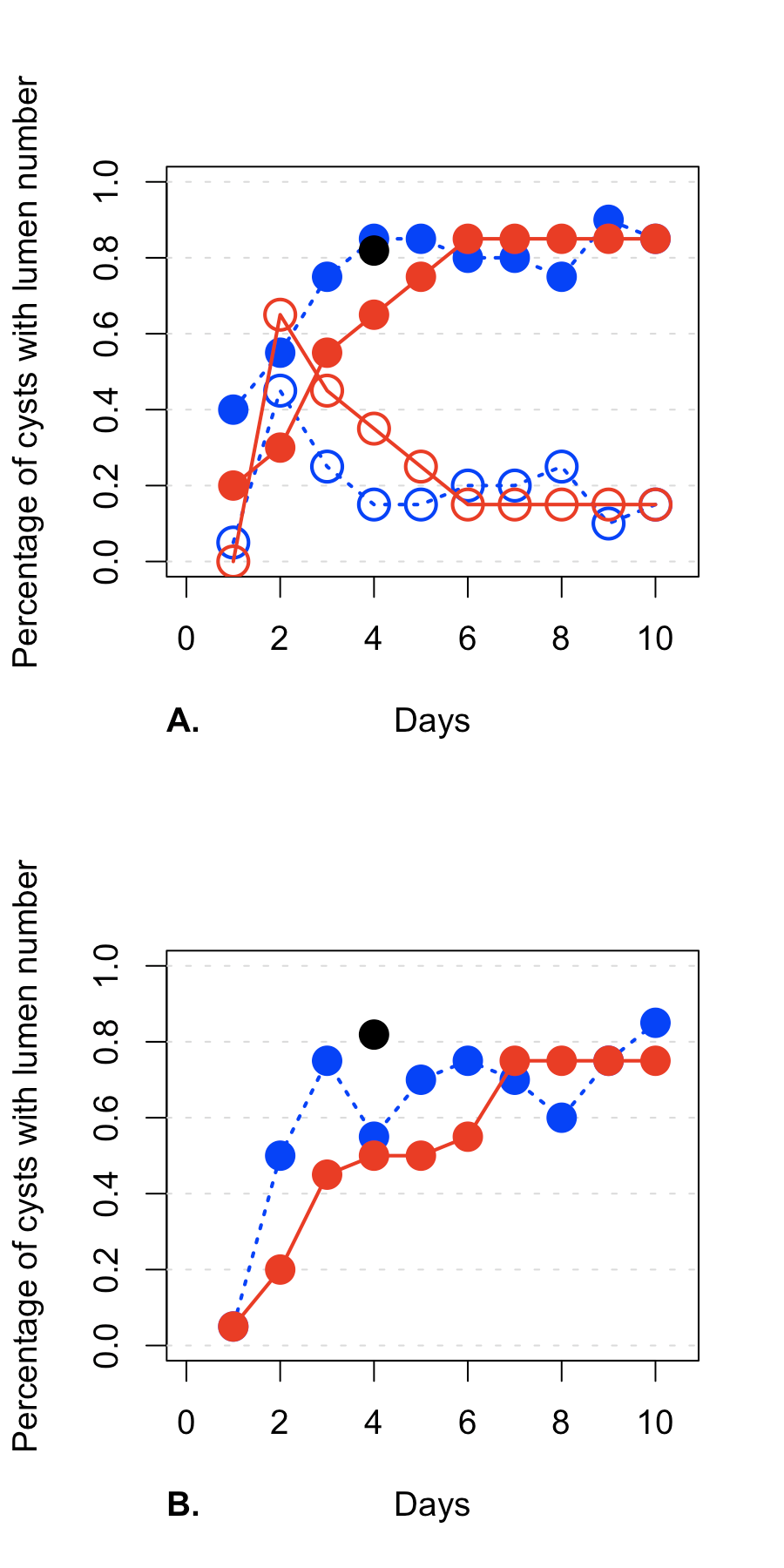

Supplement: Figure S10 — Percent of cysts with different numbers of lumens for GM ISMA. The experiments are the same as in Figure S9. Designations and symbols are the same as in Figure 3. (TIF) [file pcbi.1002030.s010.tif]

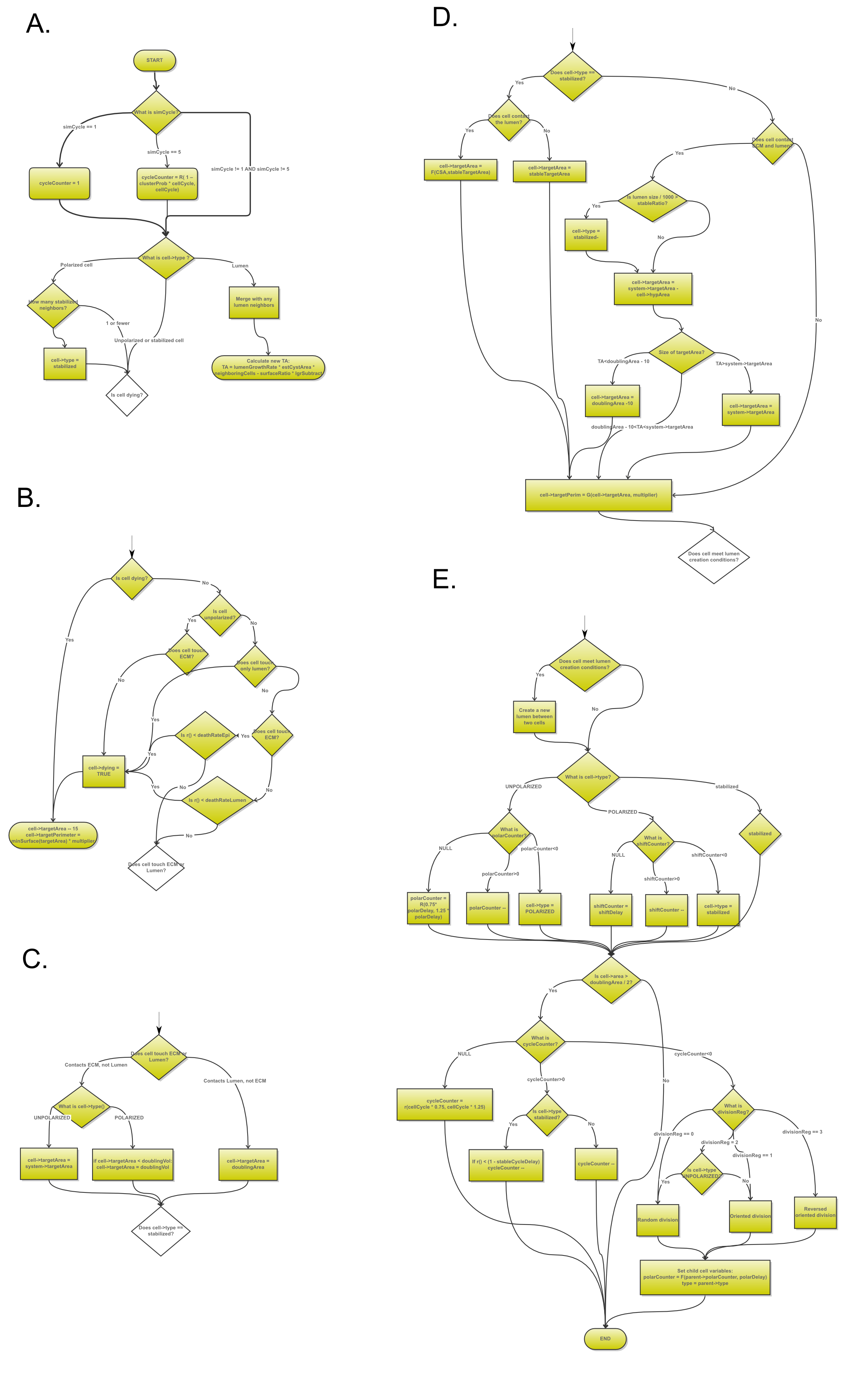

Supplement: Figure S12 — Full ISMA logic and control flow. Shown are the details of the five components of Figure 10. (TIF) [file pcbi.1002030.s012.tif]

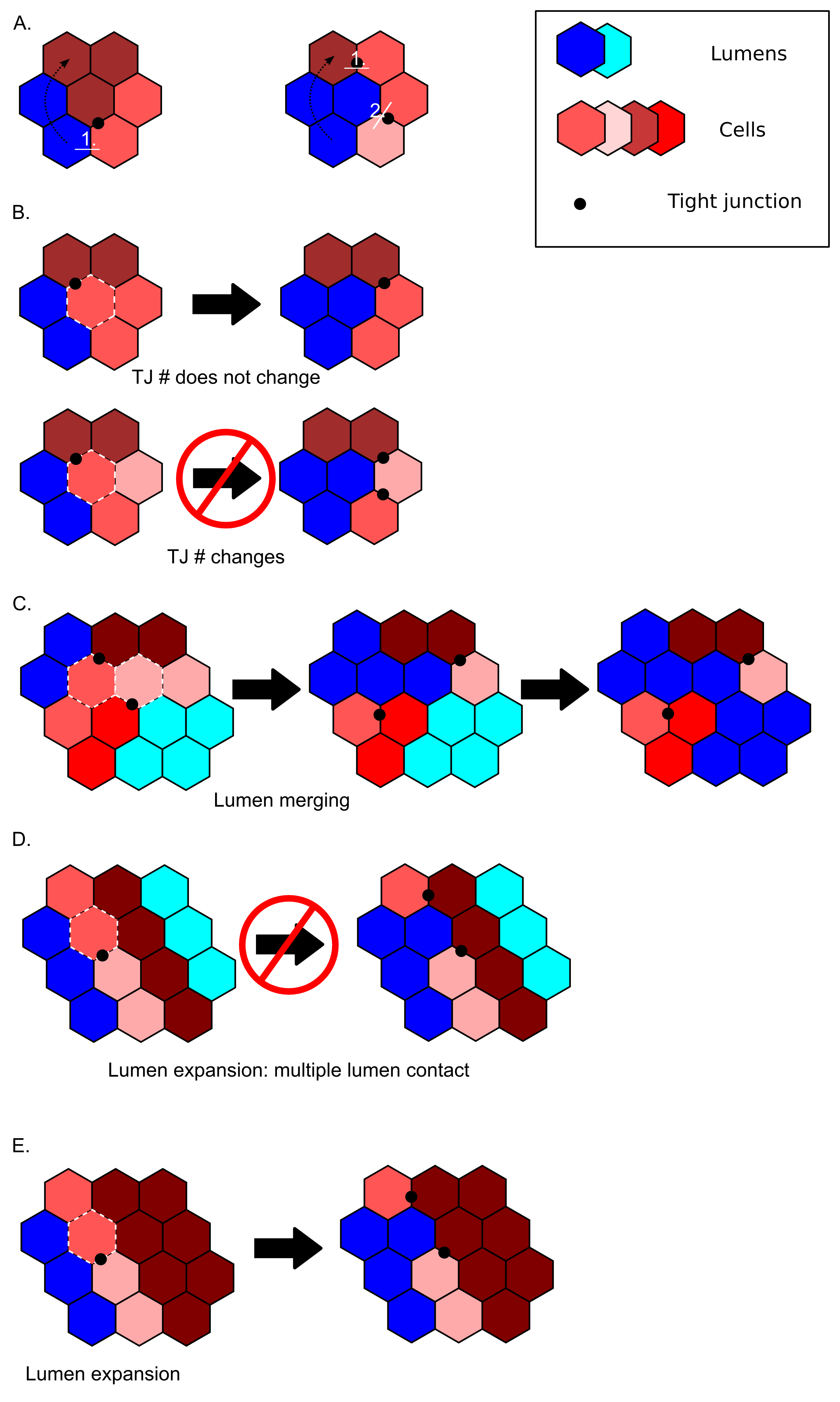

Supplement: Figure S13 — Tight junction reorganization. Tight junctions (TJs) prevent cells from contacting multiple lumens. A) TJ counting when a point is within a cell (left) or lumen (right). B) During the index change step, index changes that result in a different number of TJs before and after the change will be rejected. C) When pairs of TJs are adjacent and meet requirements, lumens will merge together. D) TJ reorganization cannot occur if it will result in a cell contacting multiple lumens. E) Allowed TJ reorganization results in lumen expansion. (TIF) [file pcbi.1002030.s013.tif]
