# Supplementary material for: MDCK Cystogenesis Driven by Cell Stabilization within Computational Analogues
Source: PLoS Comput Biol. 2011 Apr 7;7(4):e1002030. doi: 10.1371/journal.pcbi.1002030 (PMC3072361; doi:10.1371/journal.pcbi.1002030)

**Figure S11−1) TS ISMA with random DIVISION**

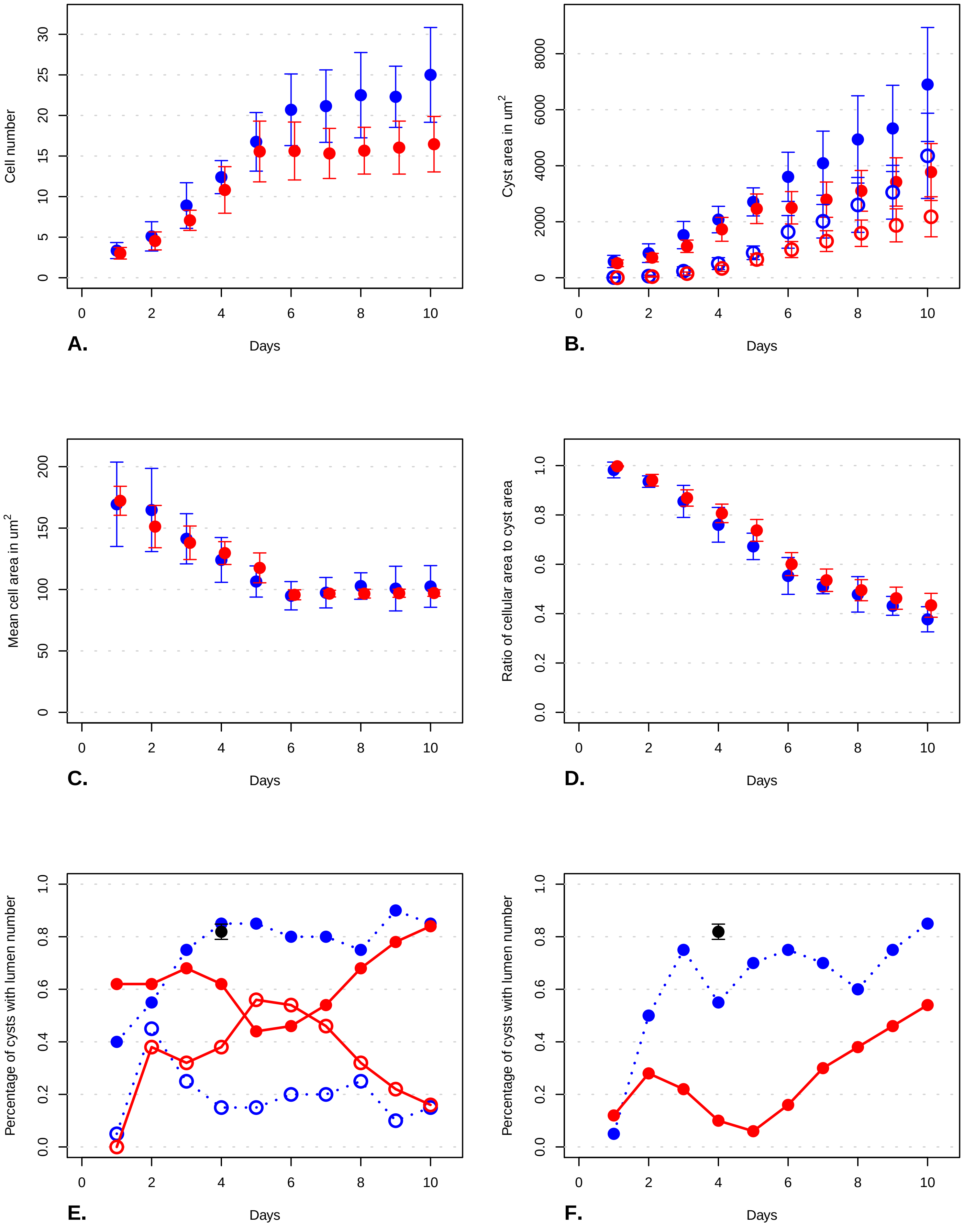

Figure S11−2) TS ISMA with reversed DIVISION

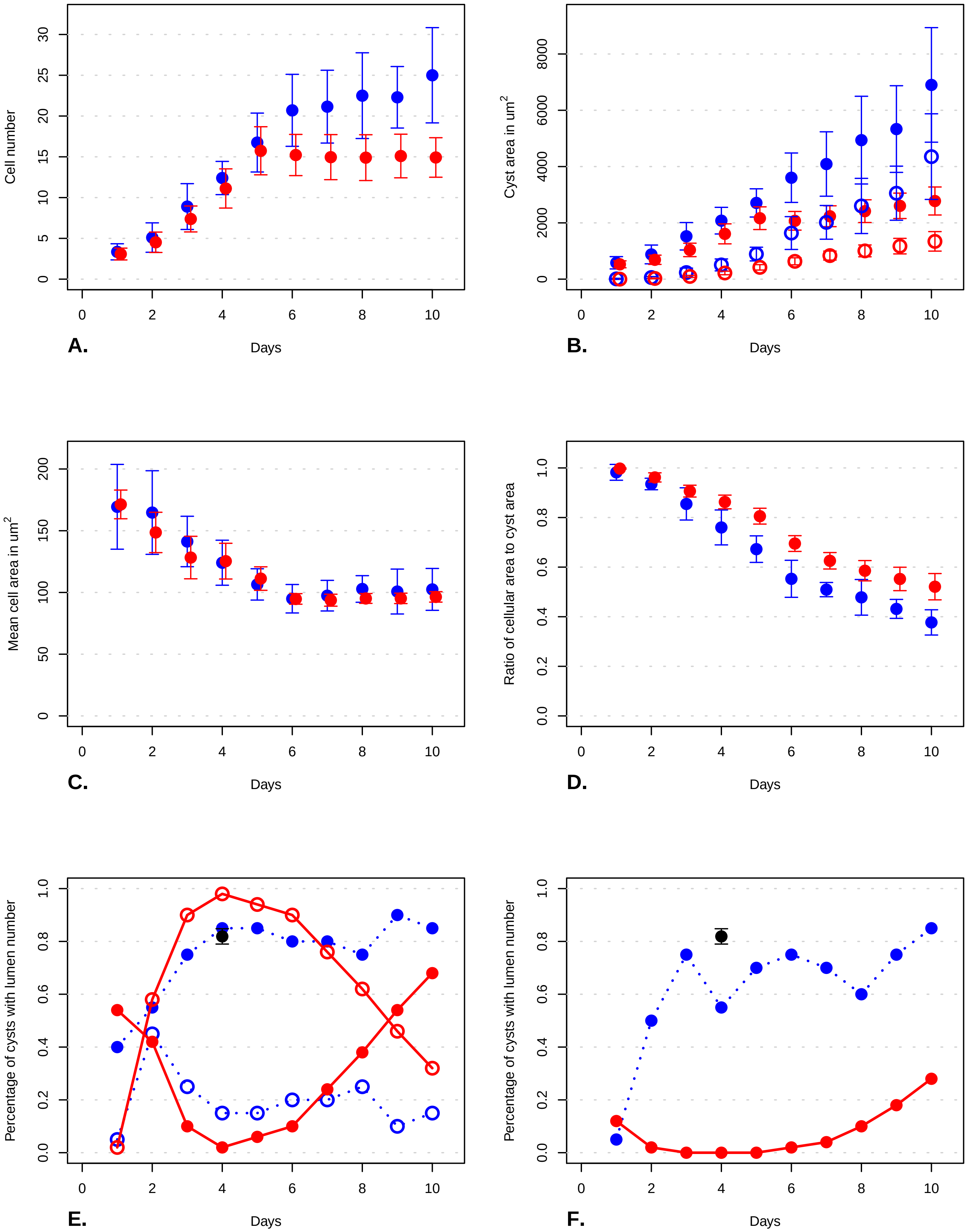

Figure S11−3) TS ISMA with no LUMINAL CELL DEATH

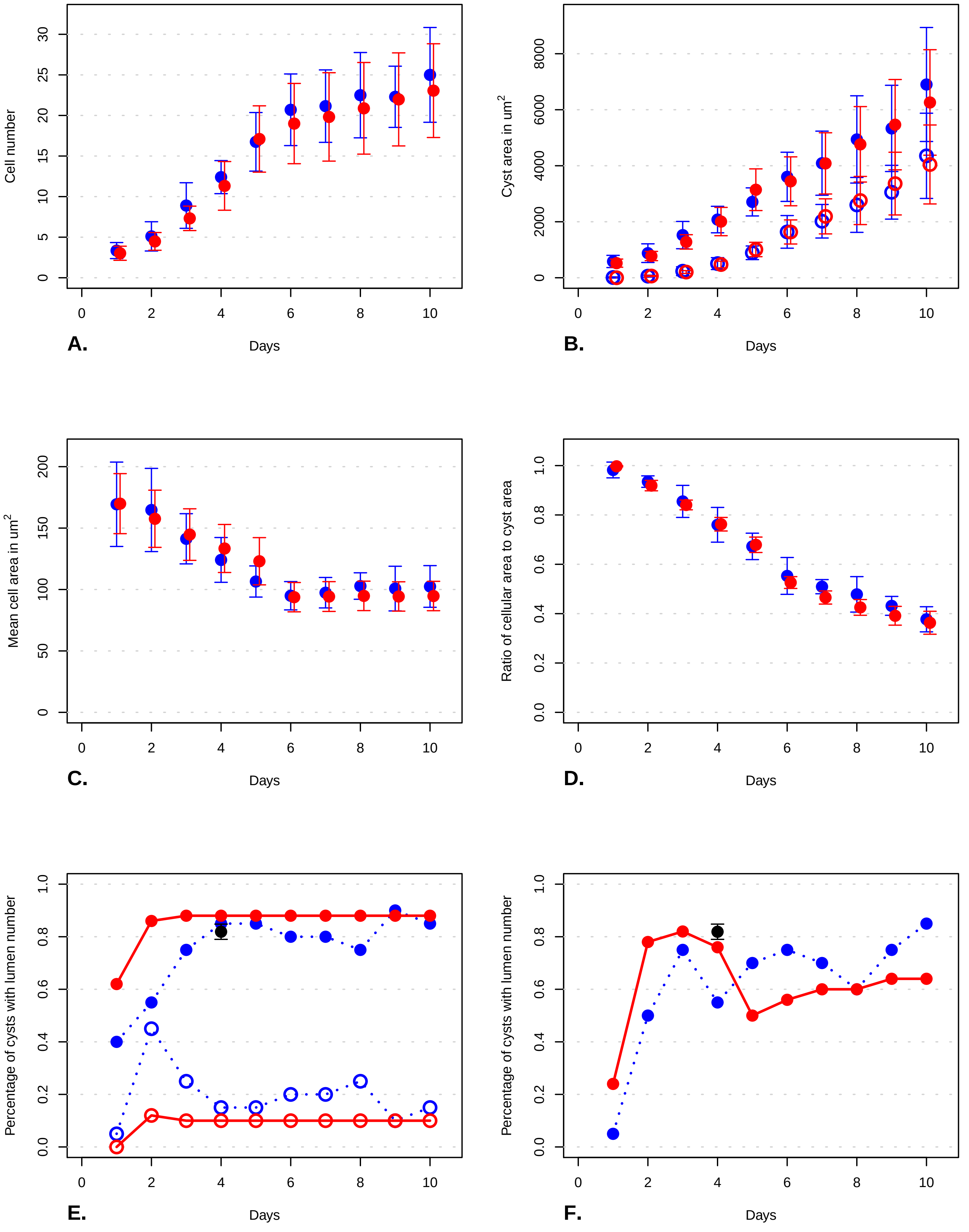

## Figure S11−4) TS ISMA with polarDelay = 130

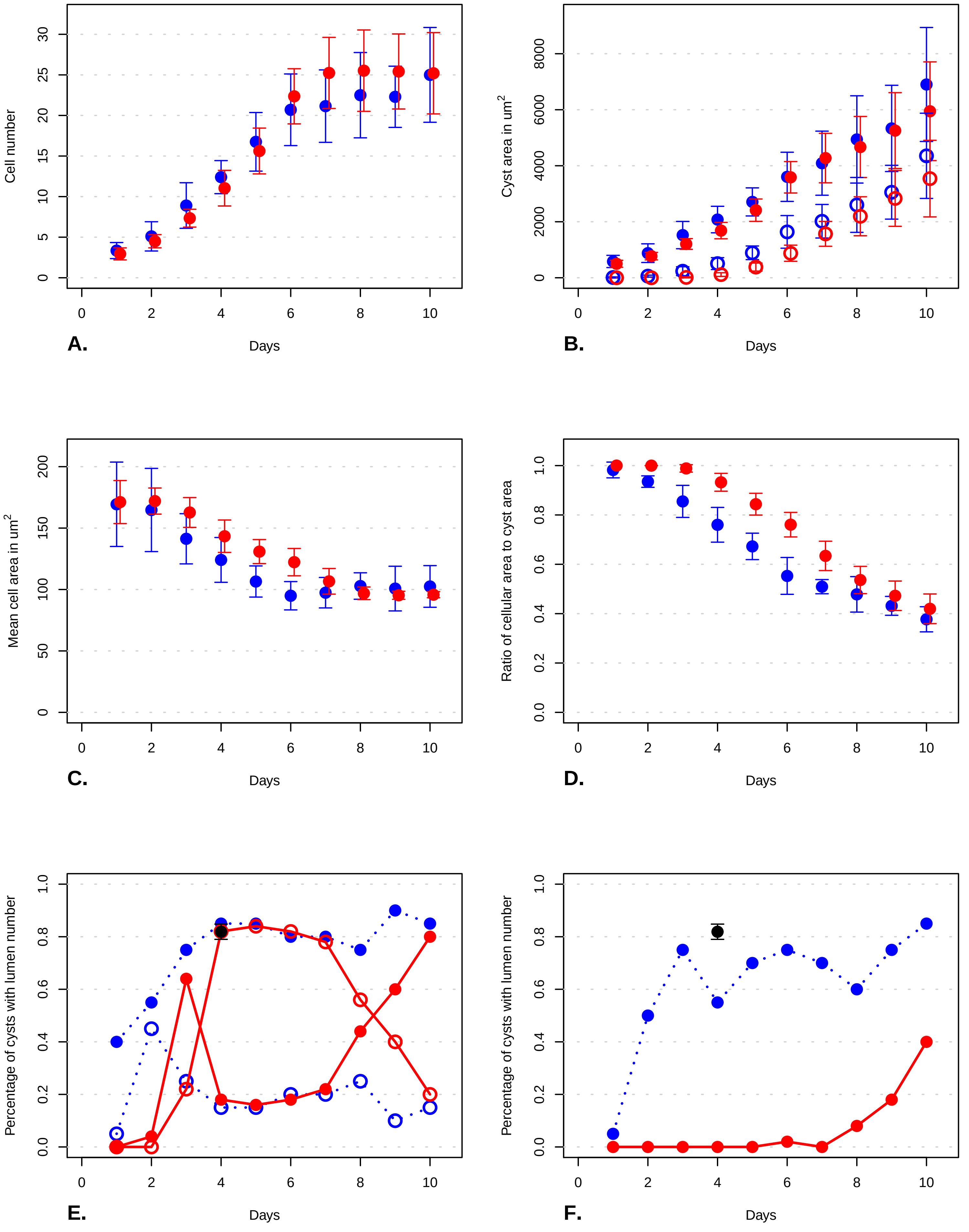

**Figure S11−5) TS ISMA with polarDelay = 130 and shiftDelay = 140**

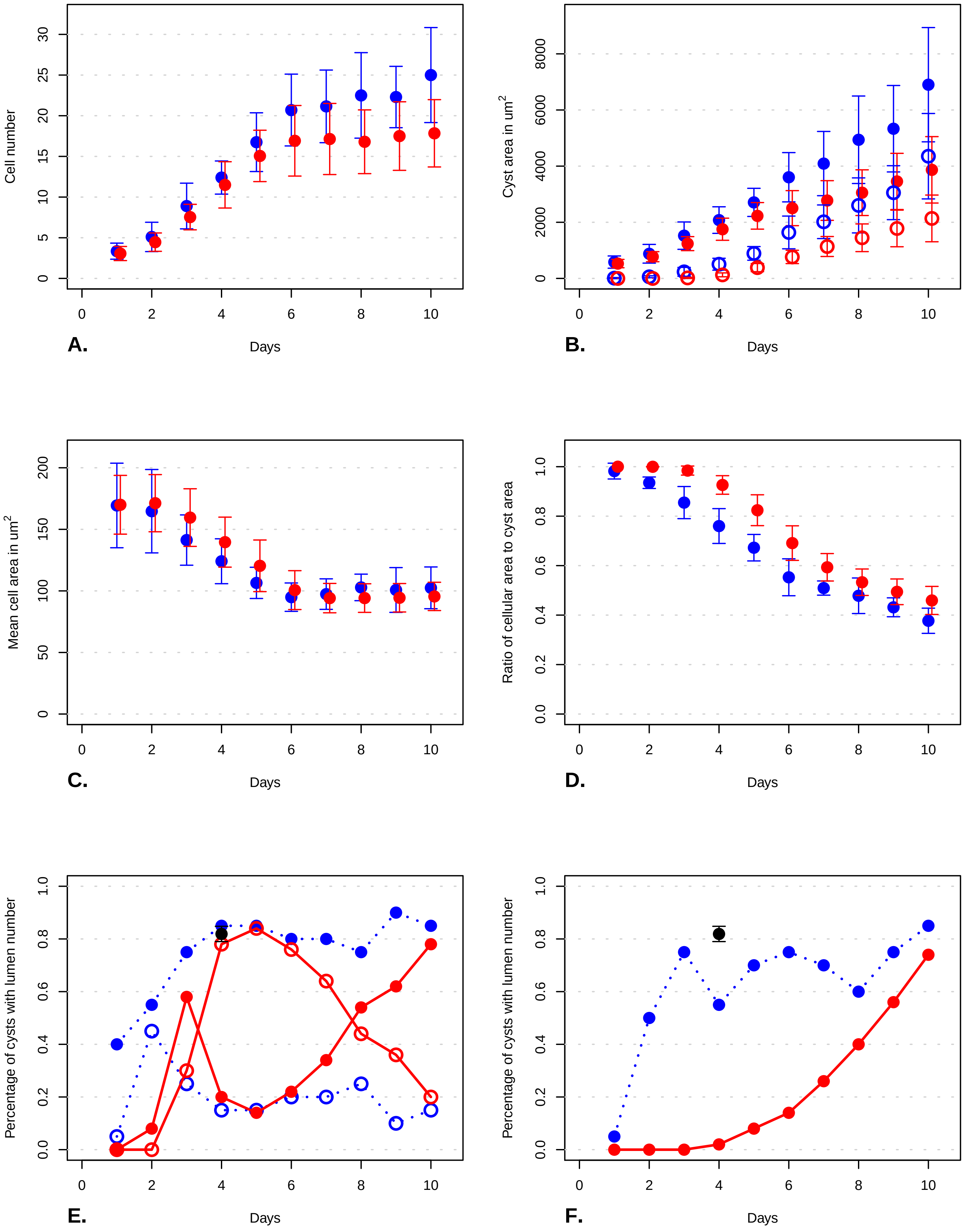

**Figure S11−6) Varied wedgeArea: w = 20**

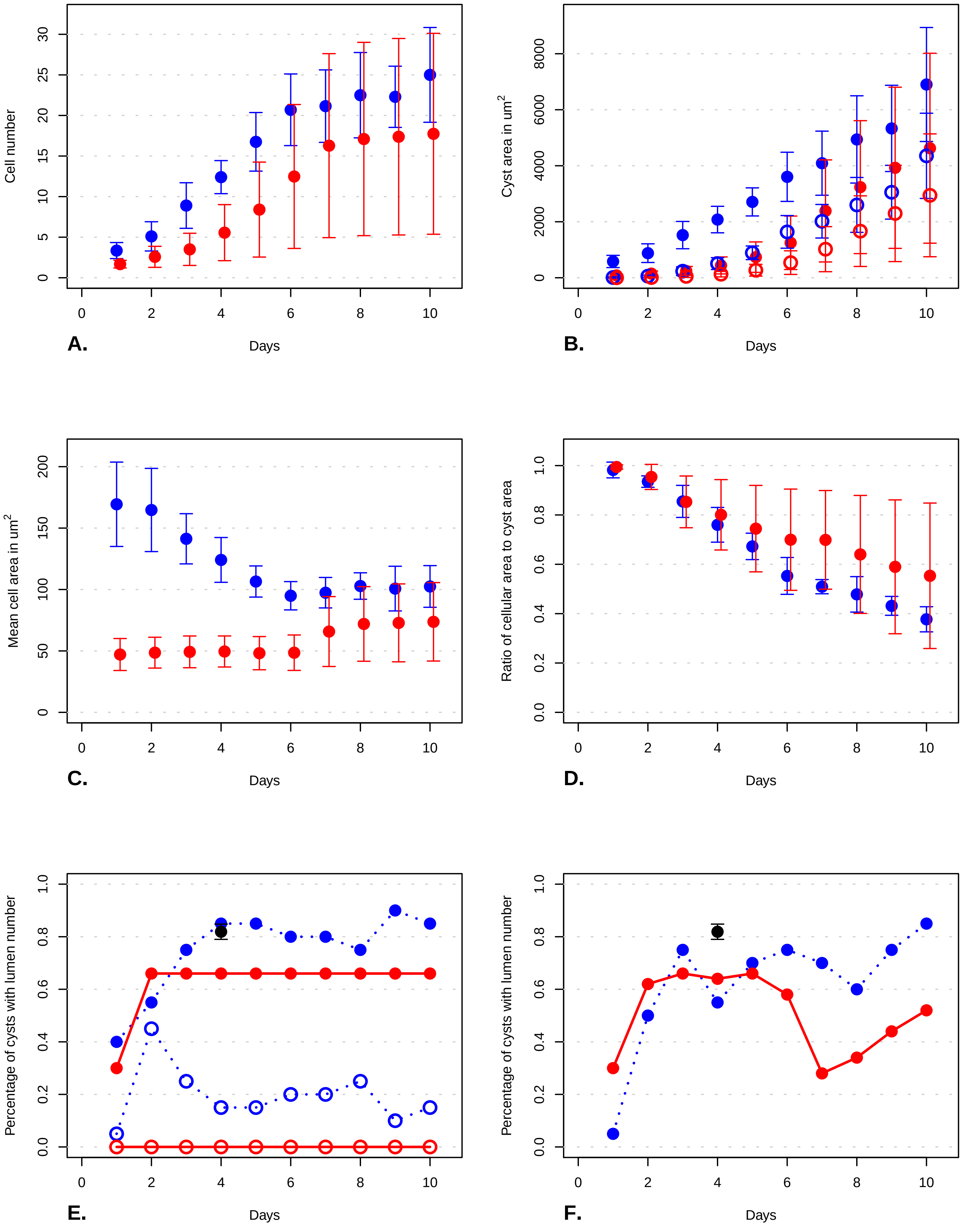

## Figure S11−7) Varied wedgeArea: w = 40

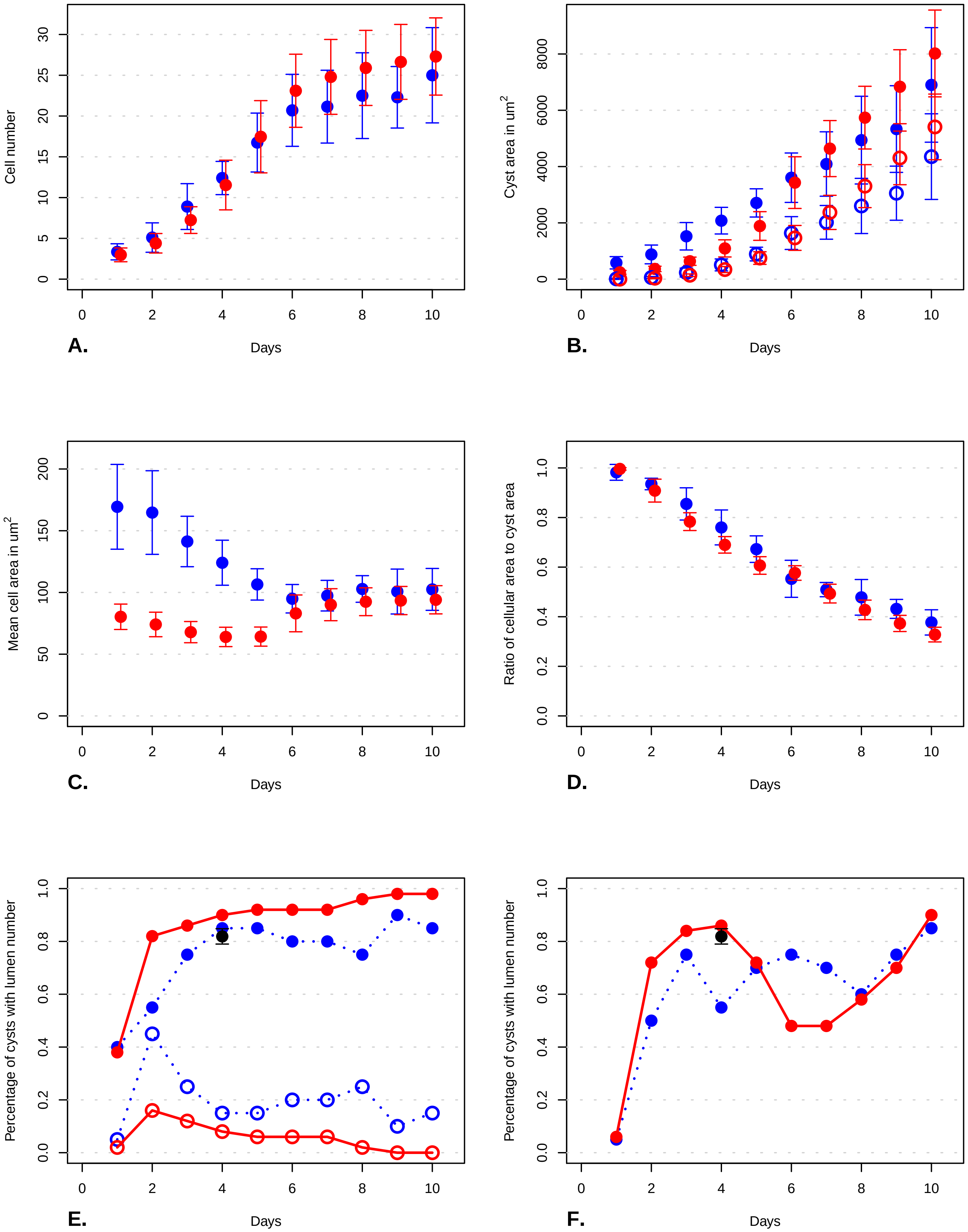

**Figure S11−8) Varied wedgeArea: w = 120**

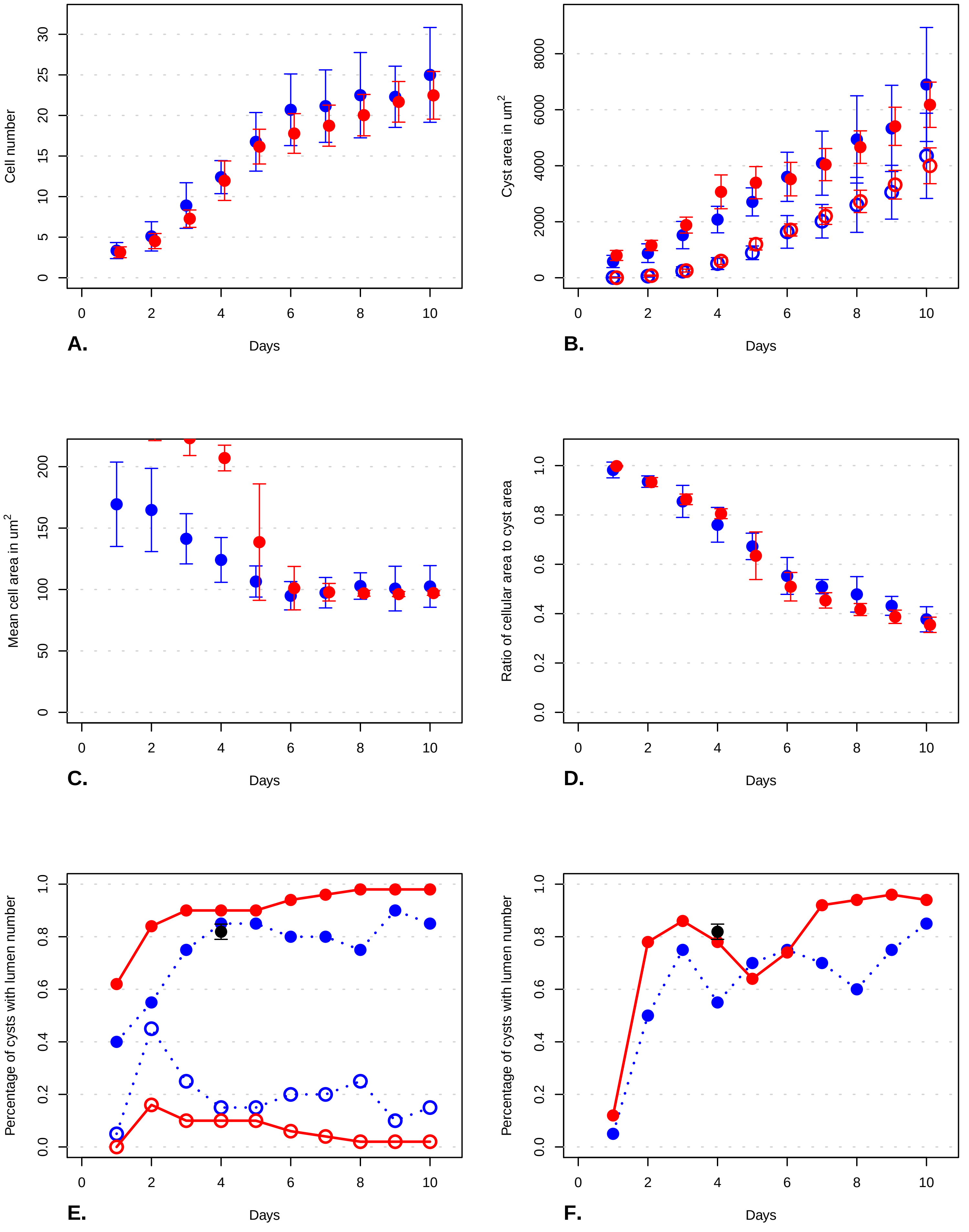

## Figure S11−9) Varied wedgeArea: w = 160

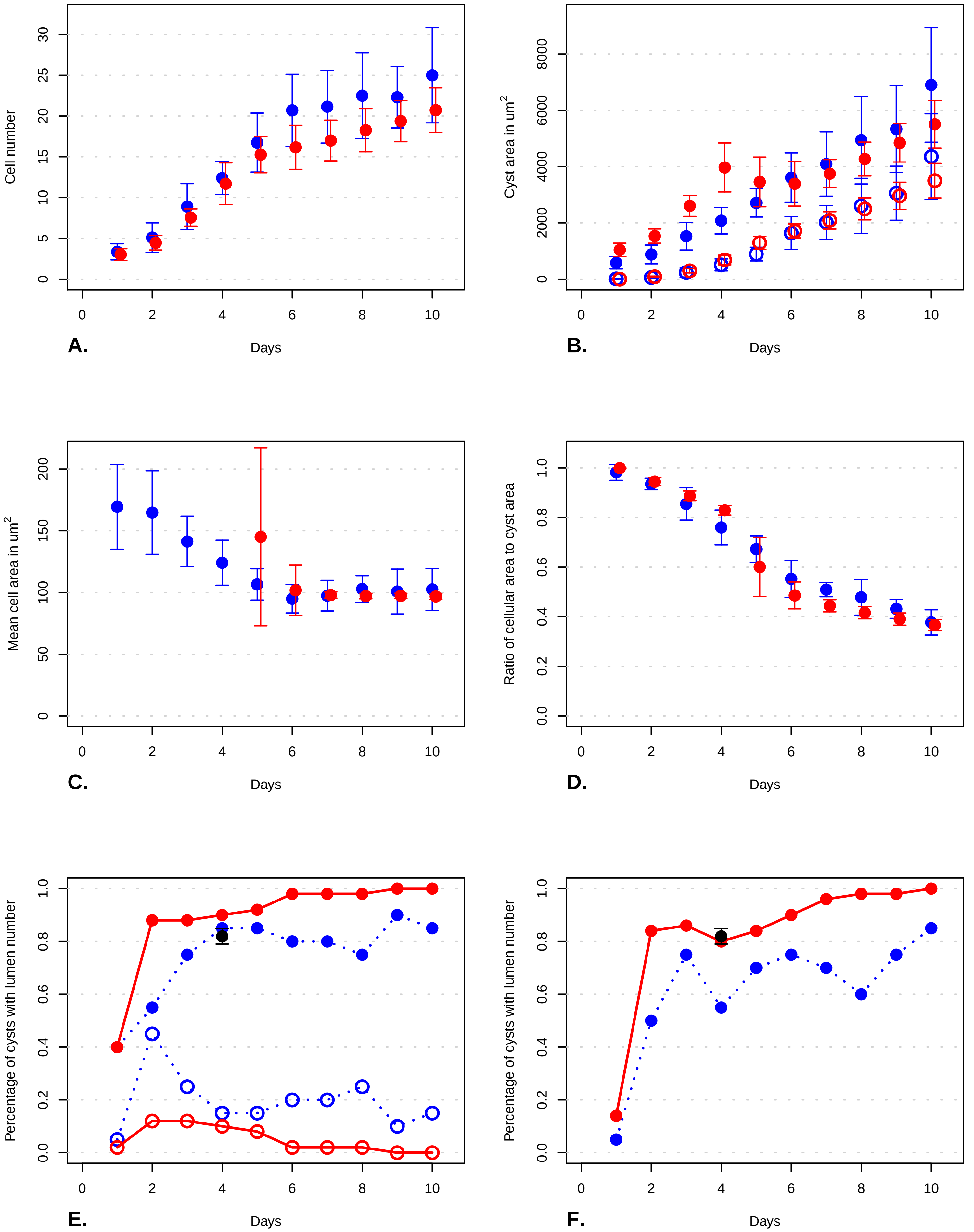

## Figure S11−10) Varied lambdaArea: la = 0.5

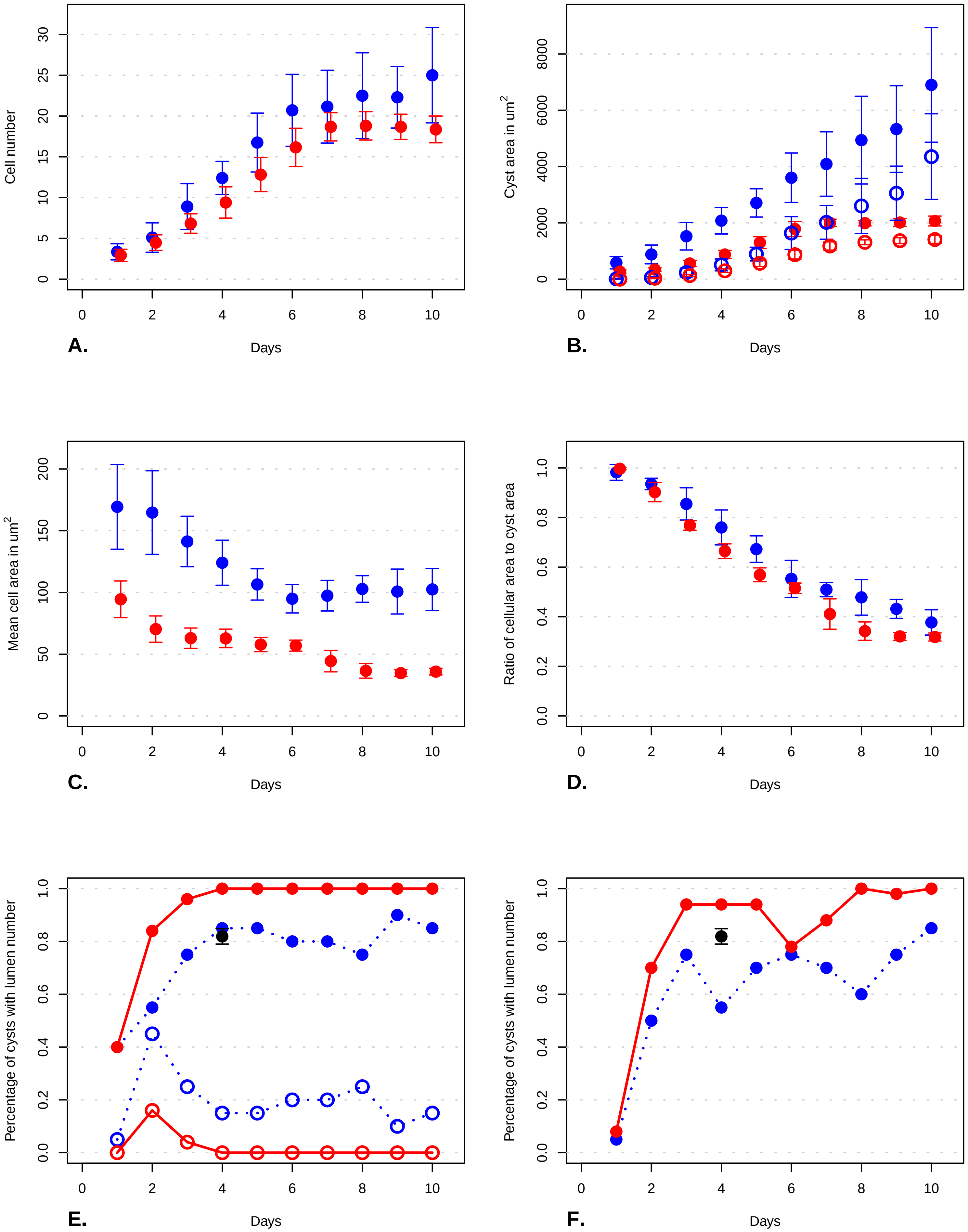

## Figure S11−11) Varied lambdaArea: la = 2.5

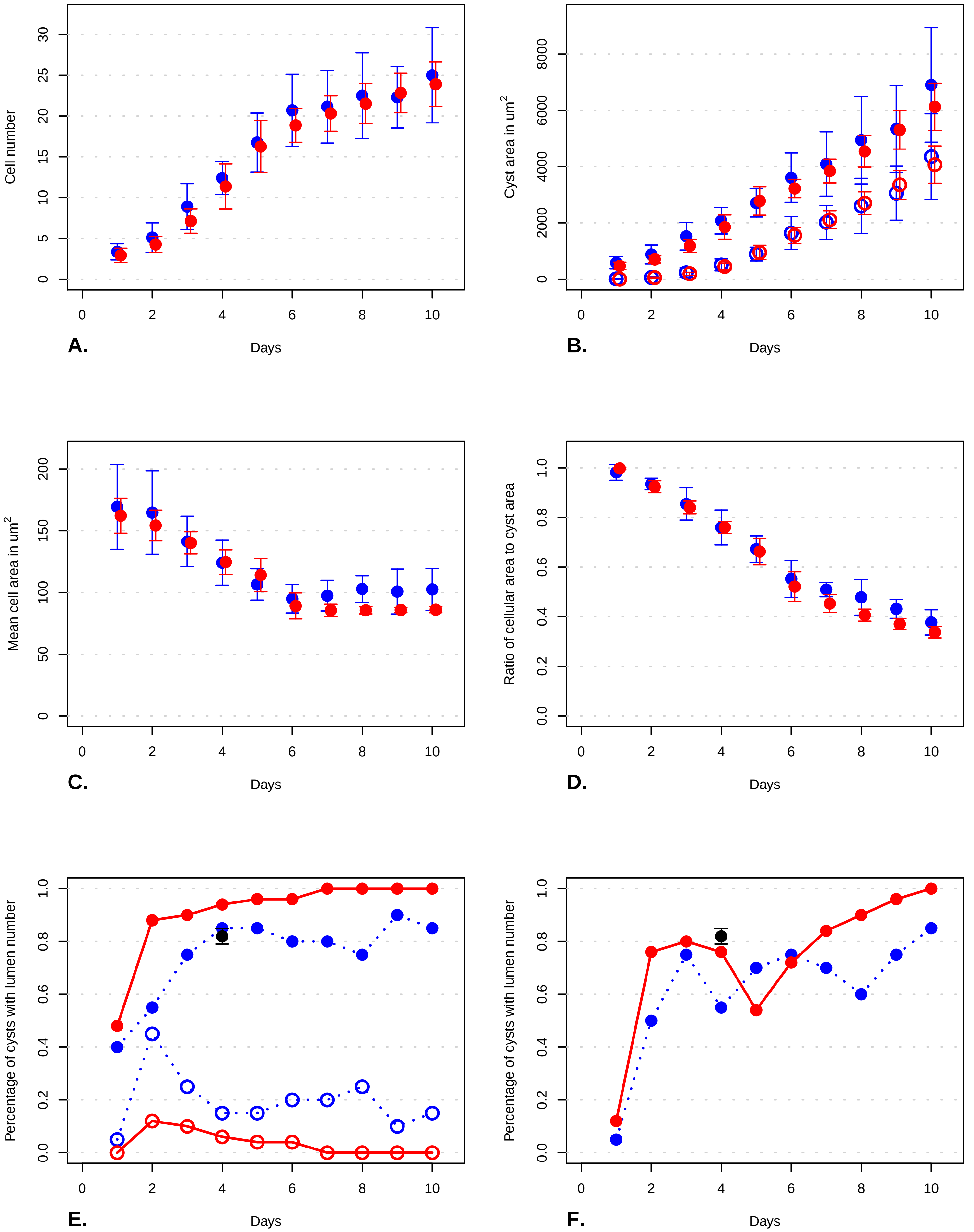

## Figure S11−12) Varied lambdaArea: la = 7.5

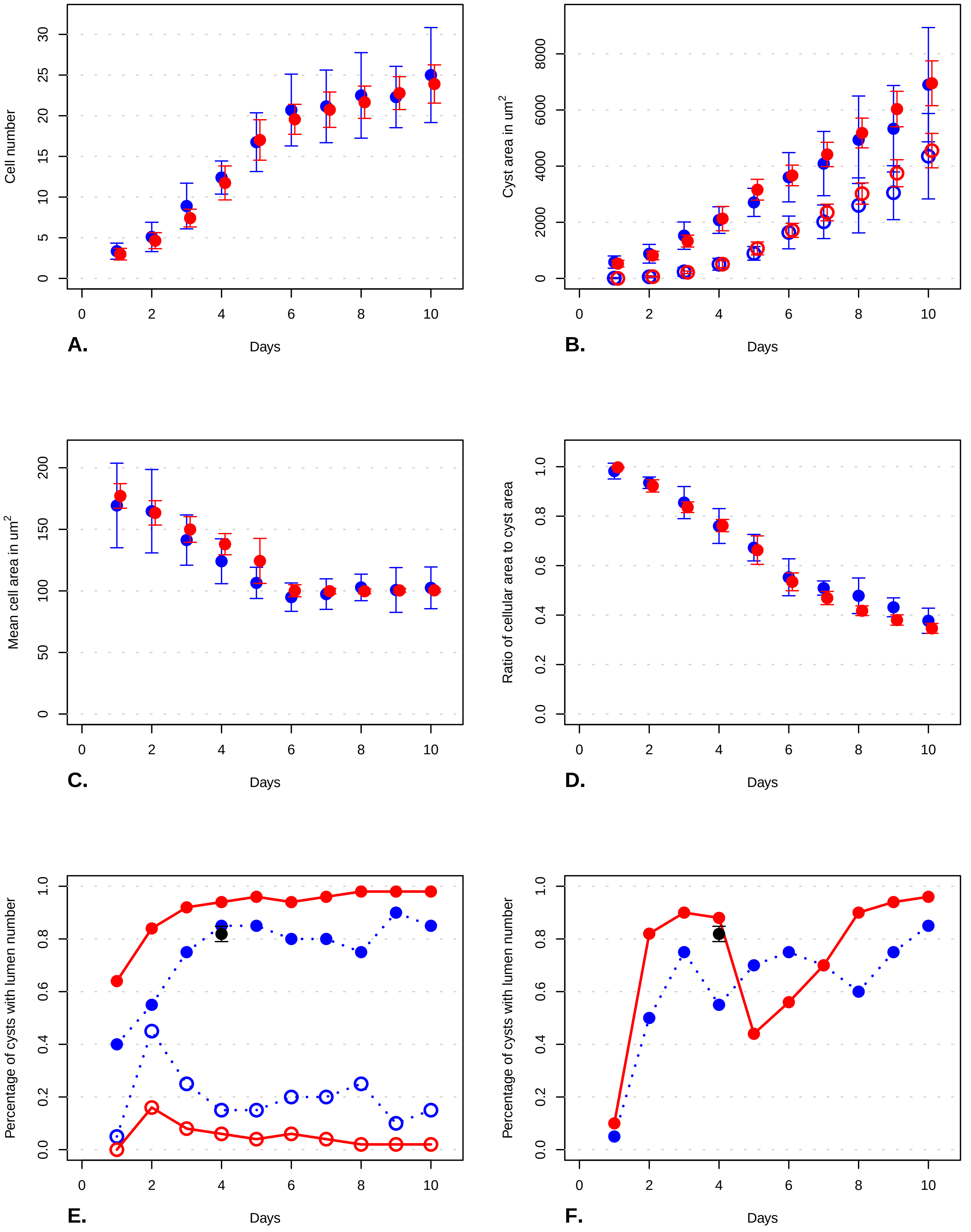

## Figure S11−13) Varied lambdaArea: la = 10

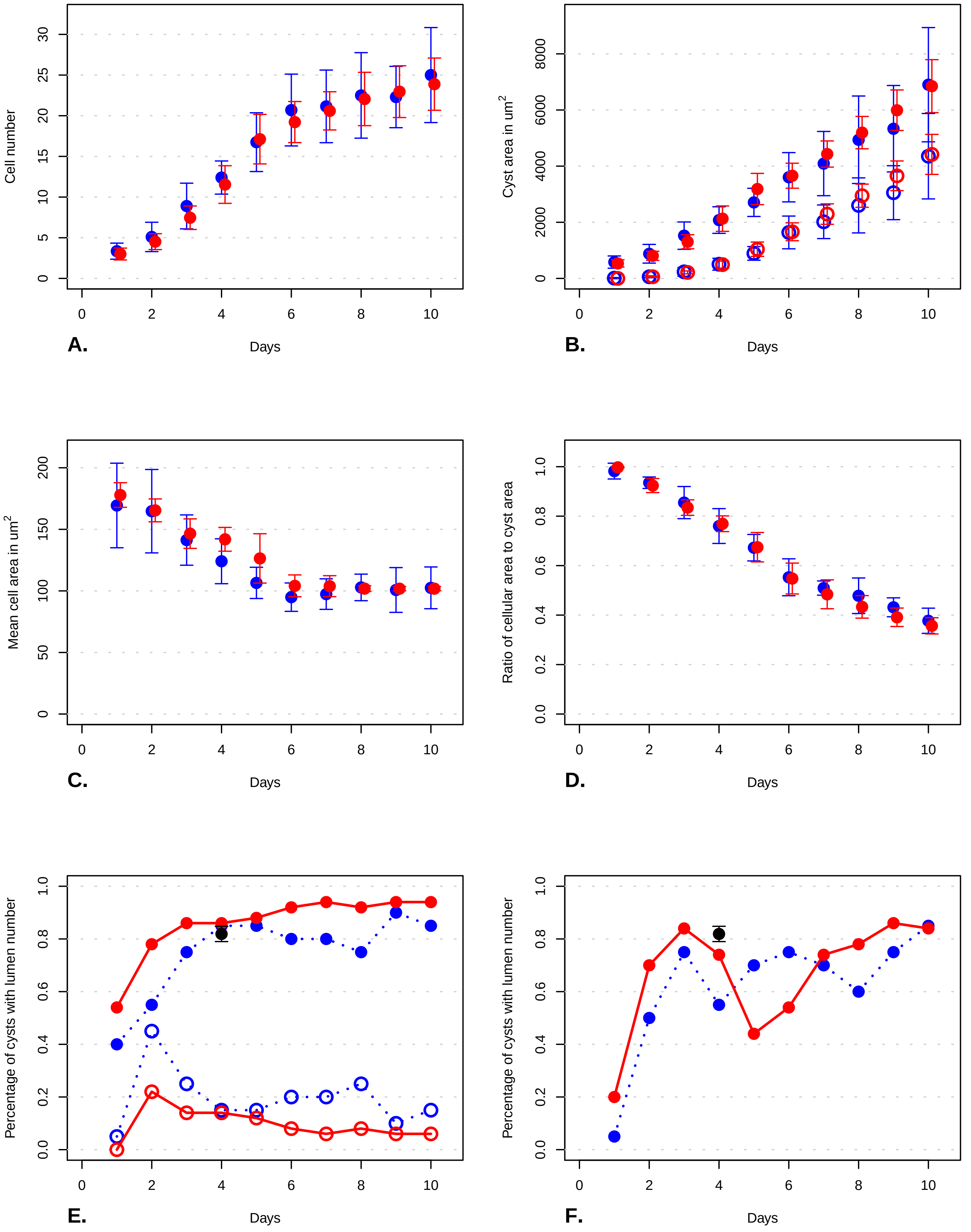

## Figure S11−14) Varied stableTargetArea: sta = 12

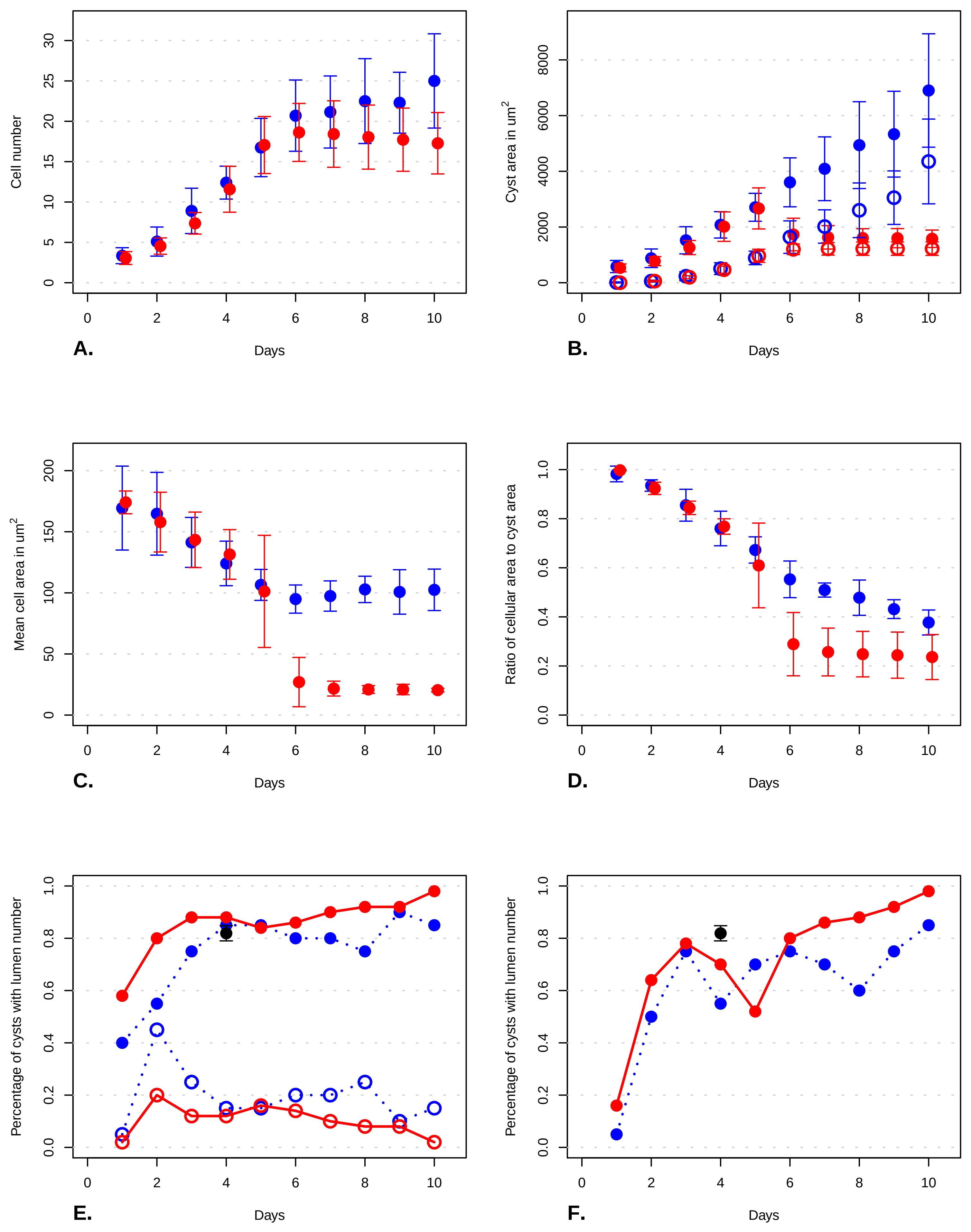

**Figure S11−15) Varied stableTargetArea: sta = 24**

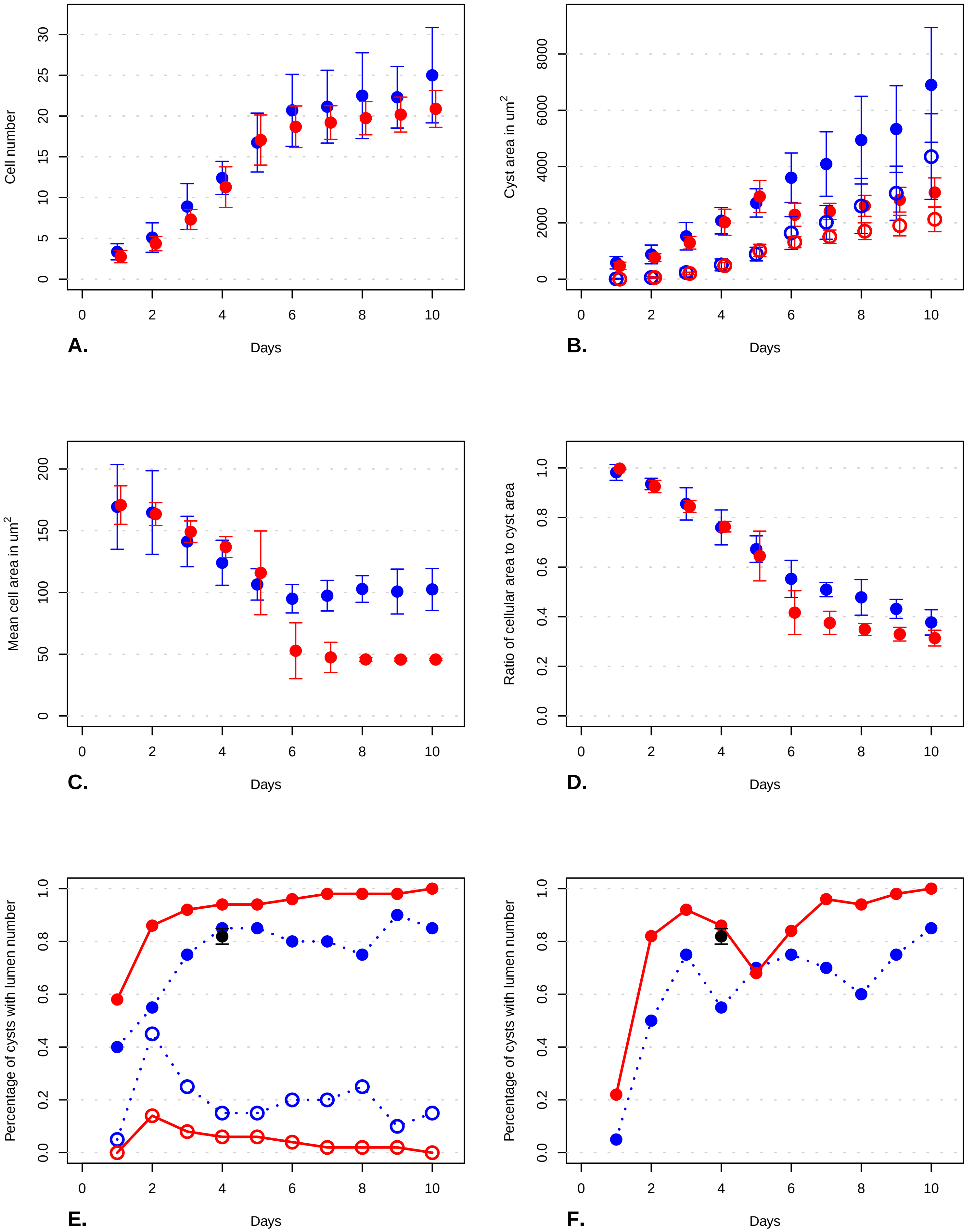

## Figure S11−16) Varied stableTargetArea: sta = 72

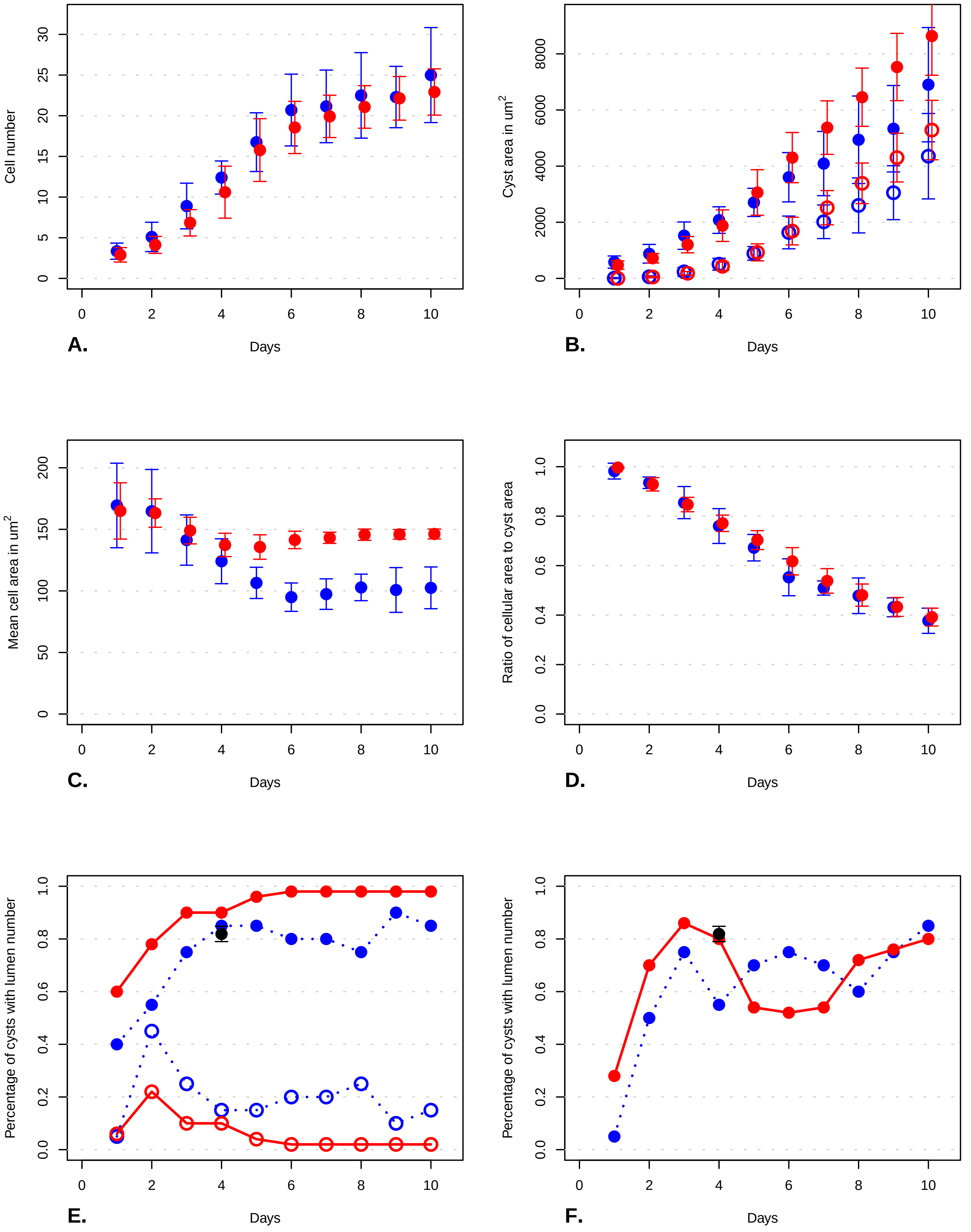

## Figure S11−17) Varied stableTargetArea sta = 96

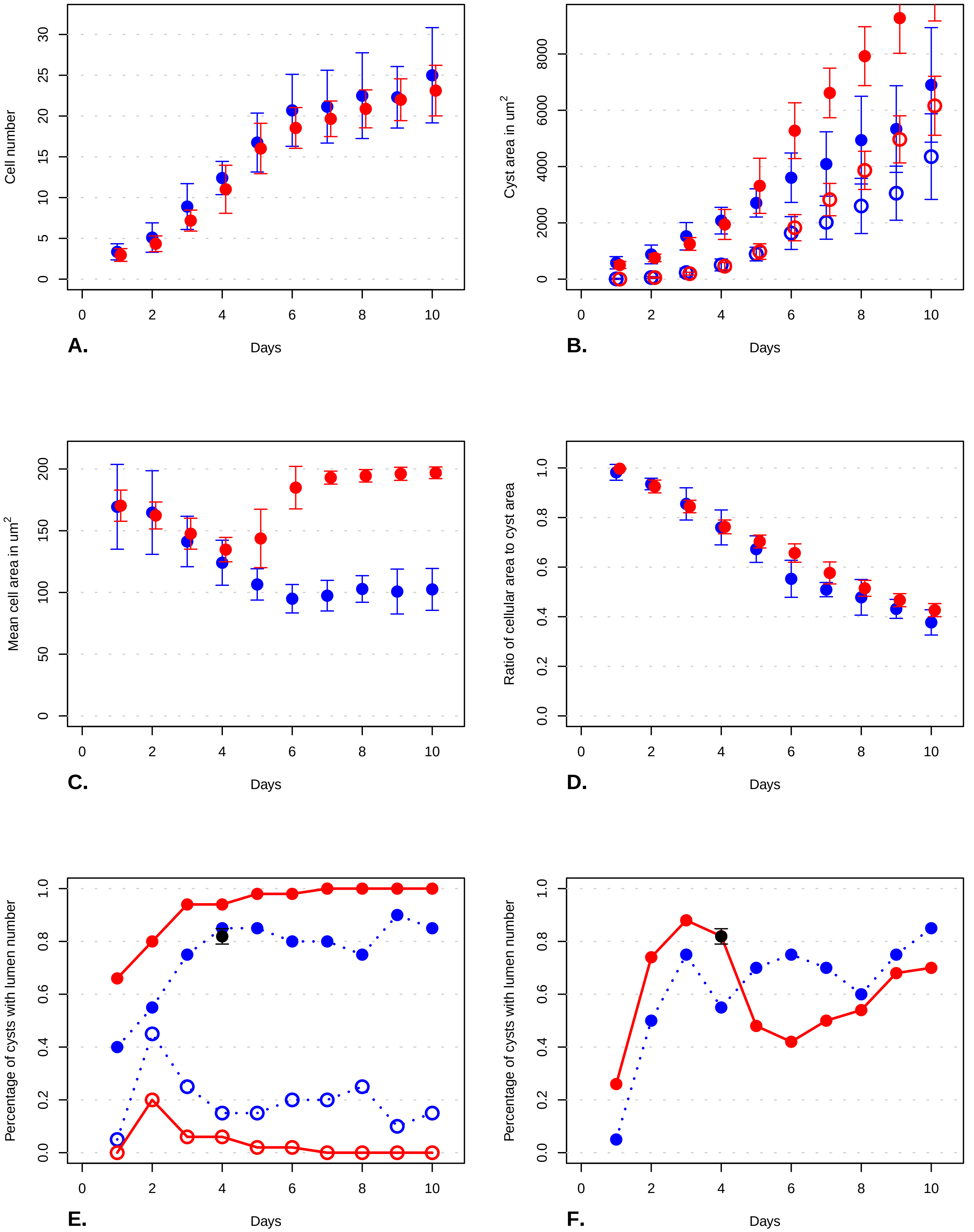

## Figure S11−18) Varied cellCycle: cc = 35

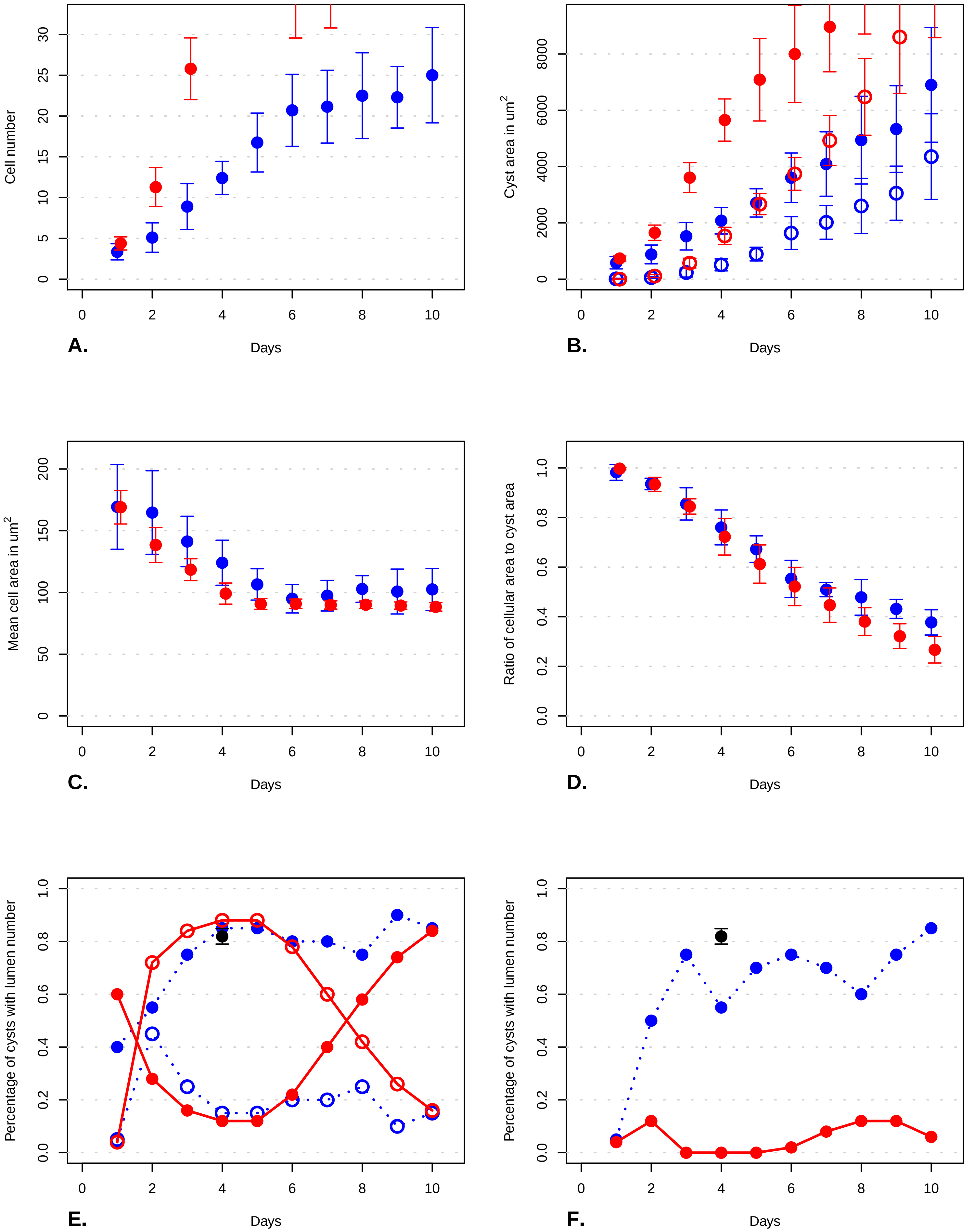

## Figure S11−19) Varied cellCycle: cc = 50

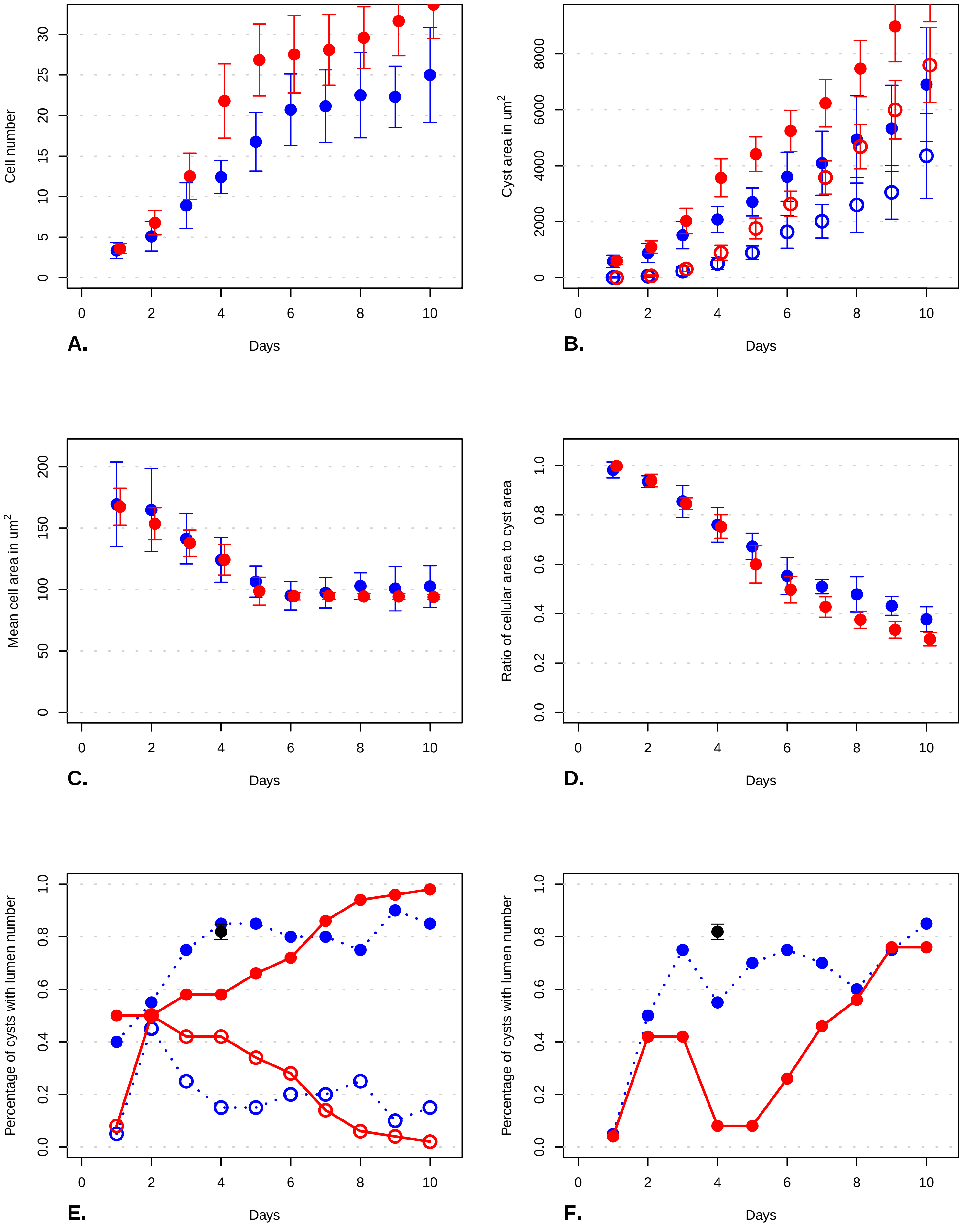

**Figure S11−20) Varied cellCycle: cc = 60**

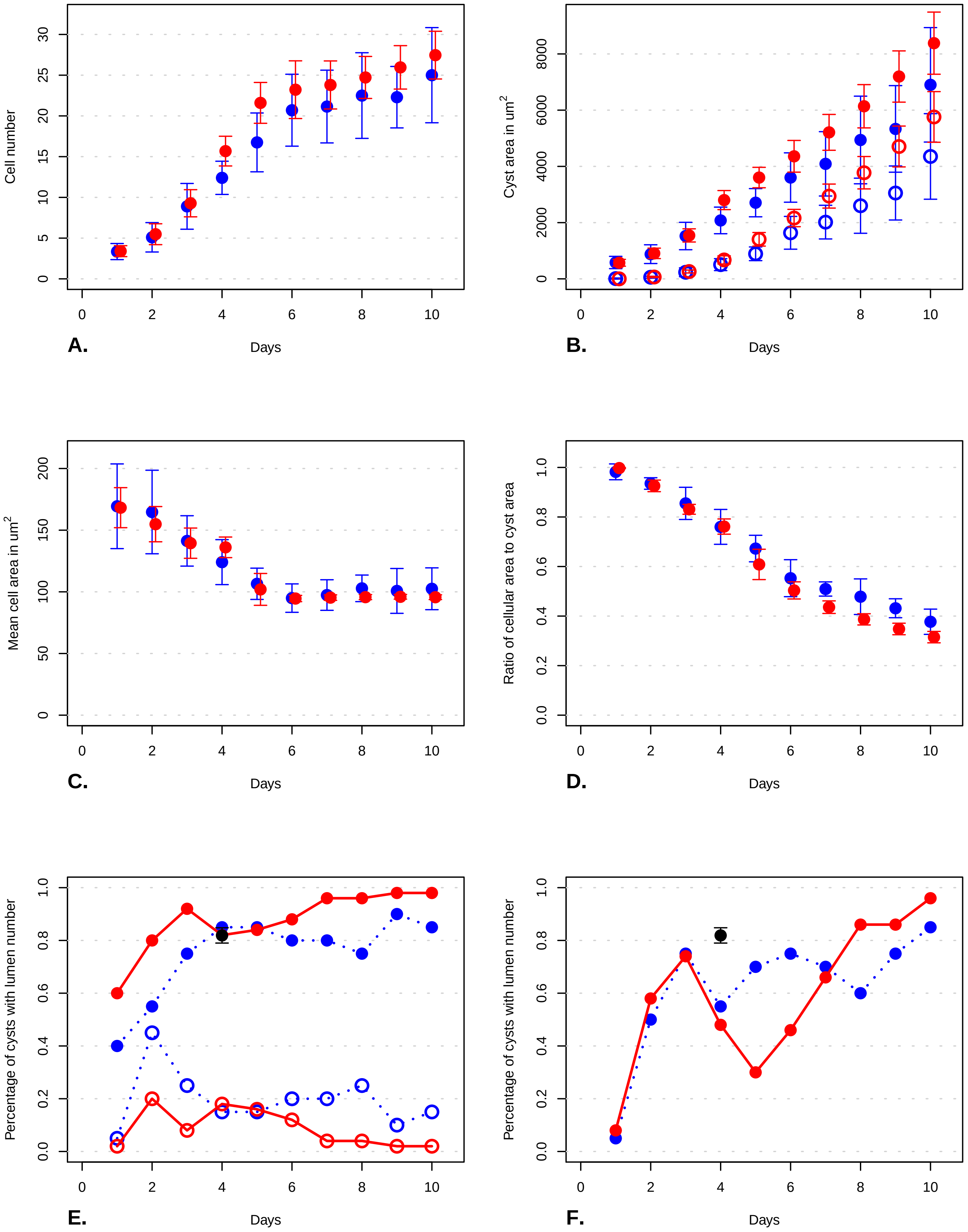

## Figure S11−21) Varied cellCycle: cc = 80

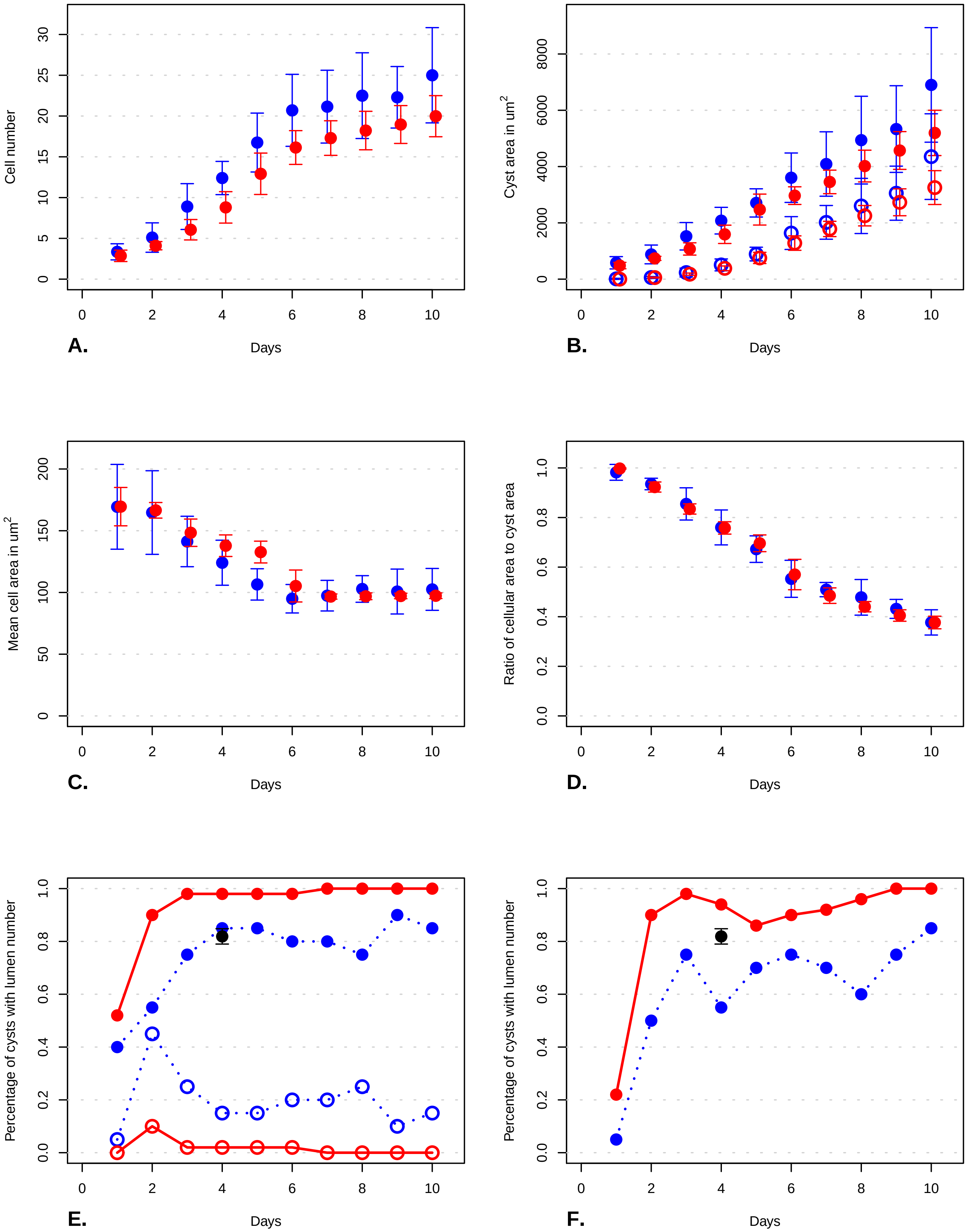

Figure S11−22) Varied cellCycle: cc = 100

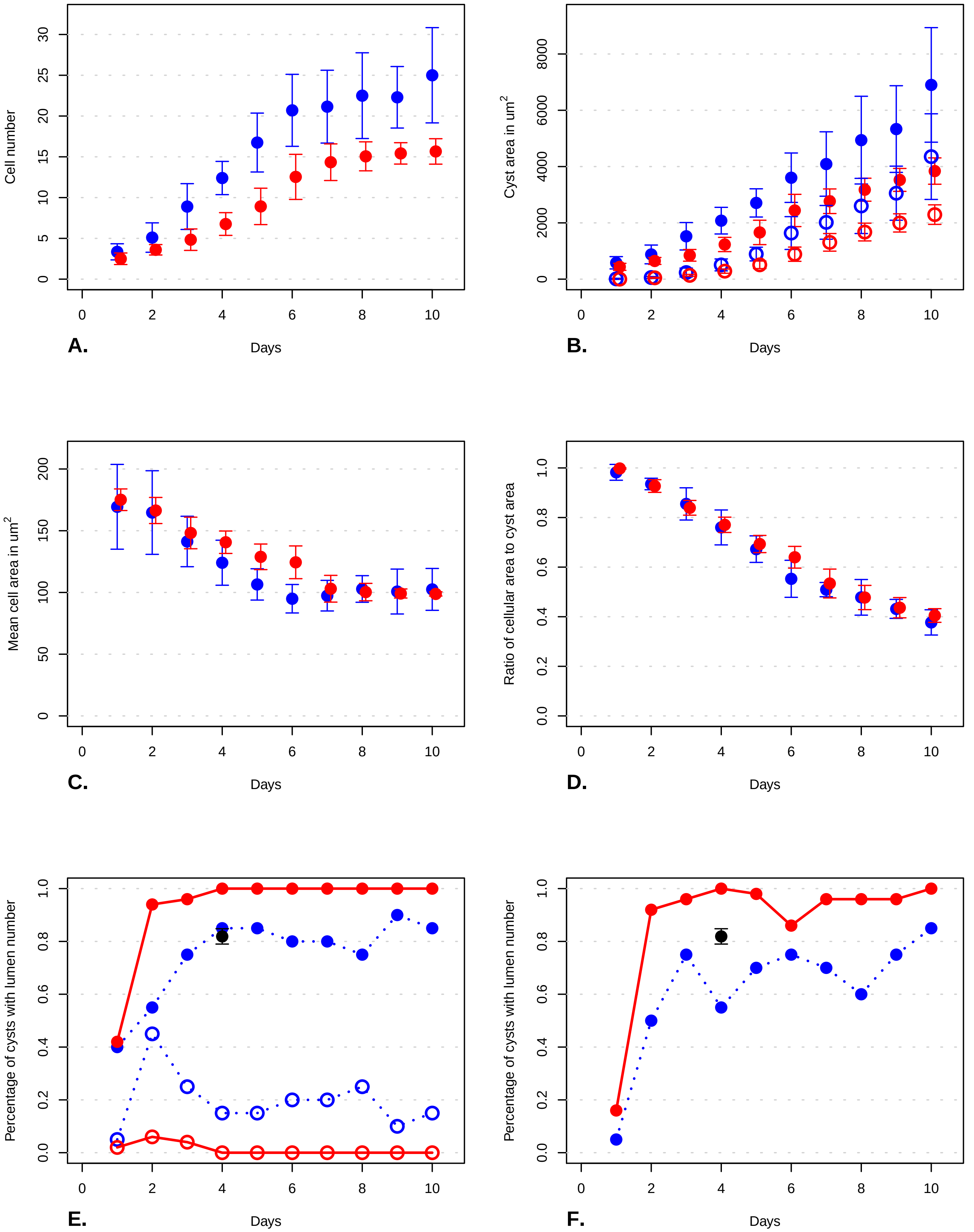

## Figure S11−23) Varied cellCycle: cc = 140

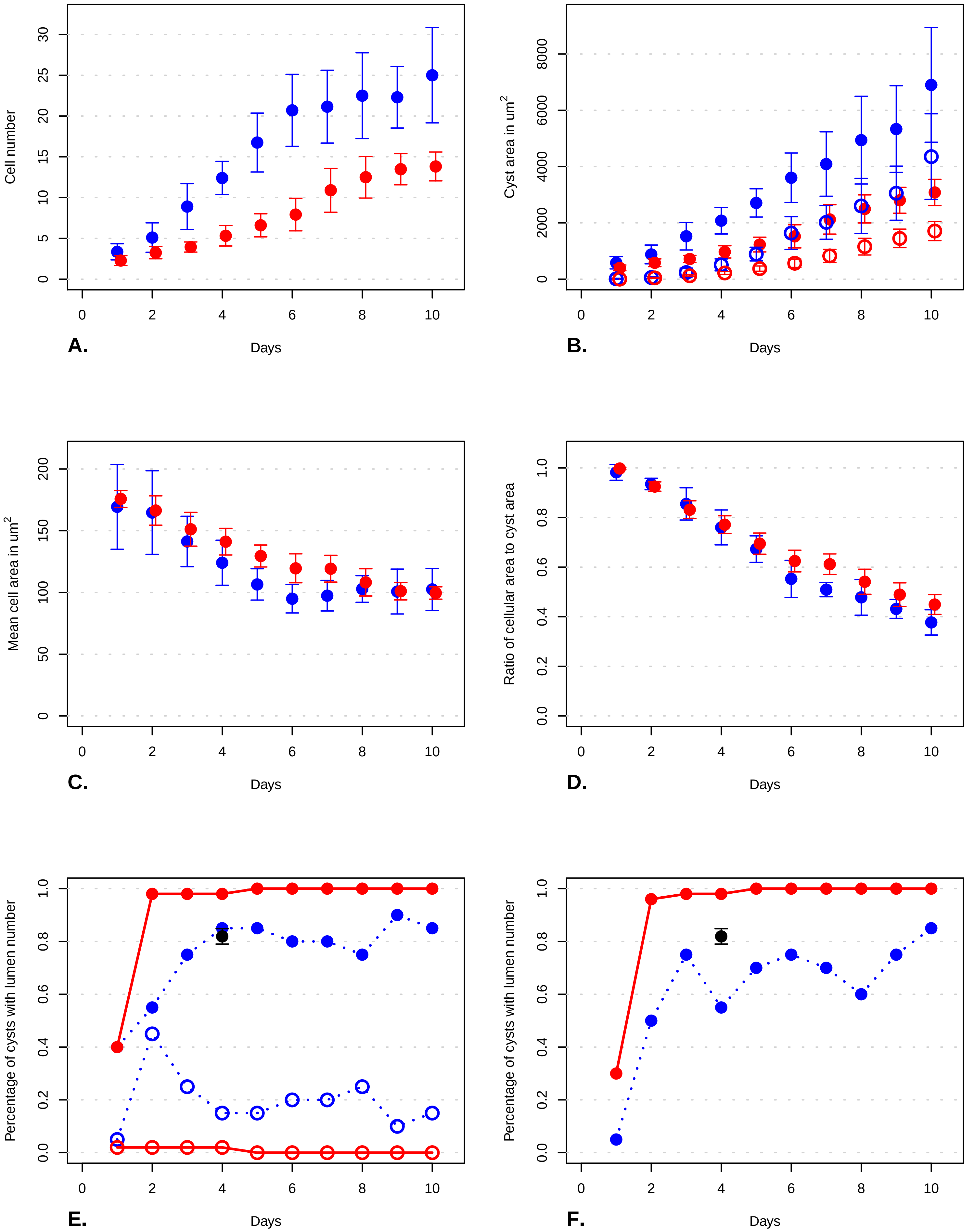

**Figure S11−24) Varied stableCycleDelay: scd = 0.5**

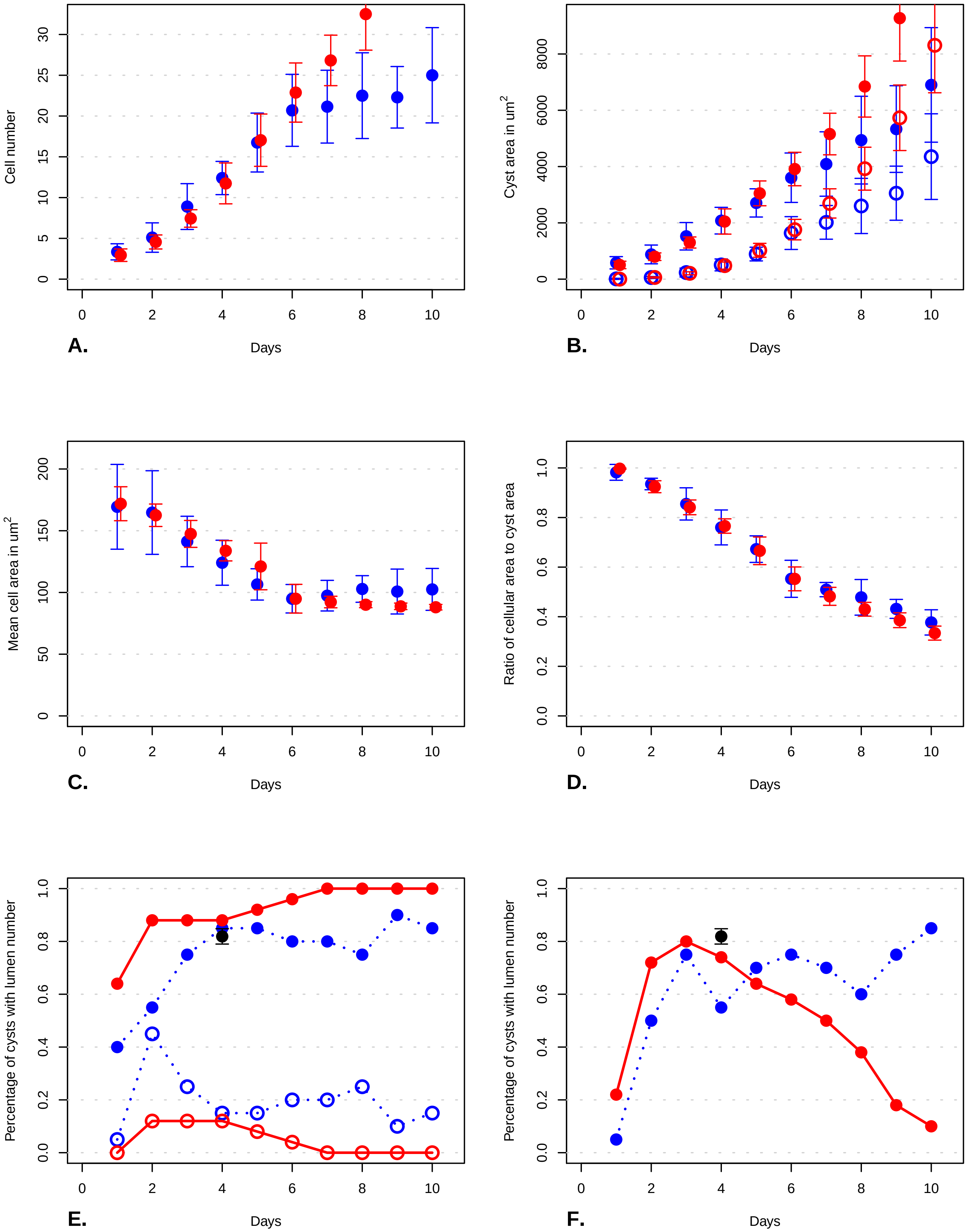

Figure S11−25) Varied stableCycleDelay: scd = 0.7

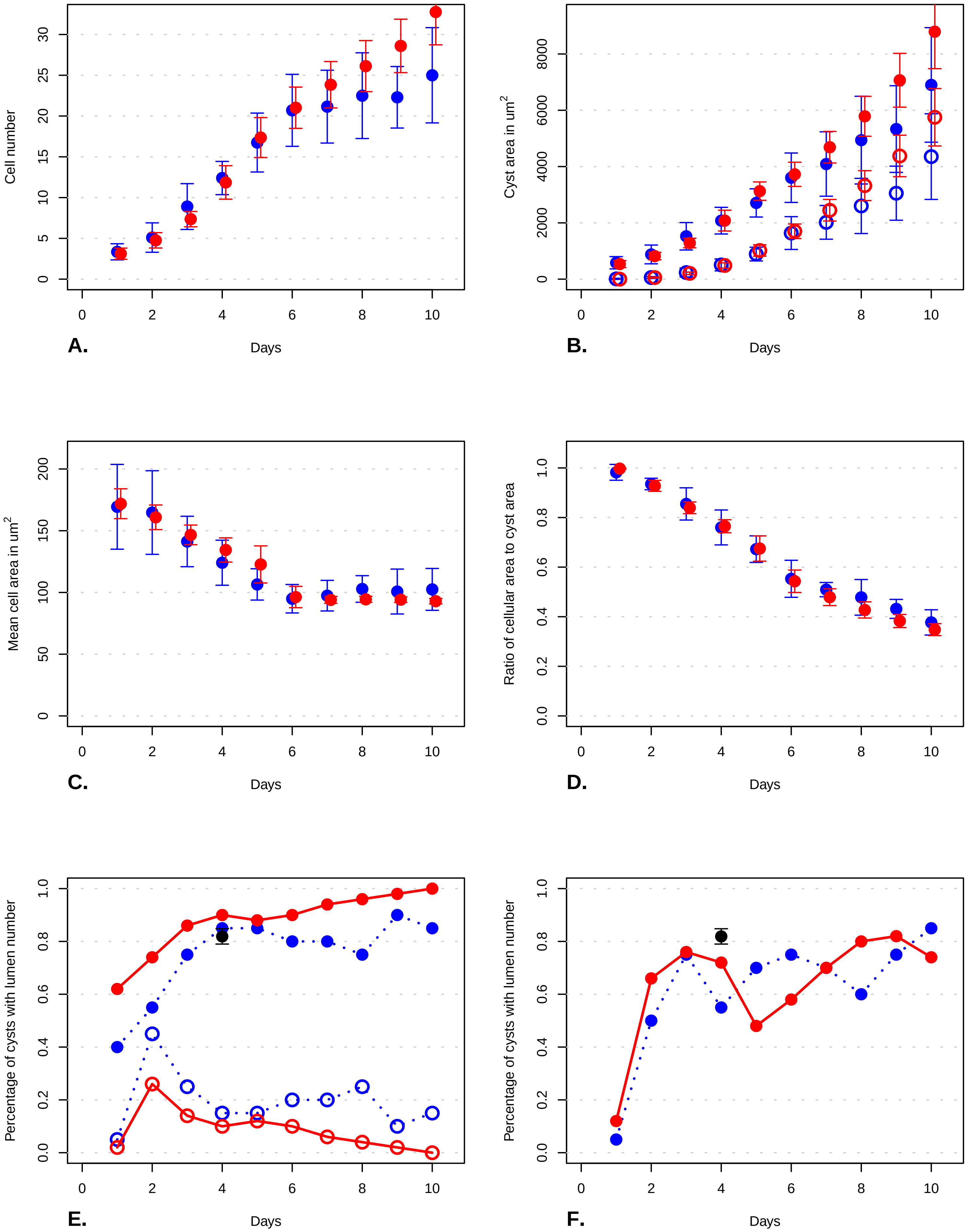

Figure S11−26) Varied stableCycleDelay: scd = 0.9

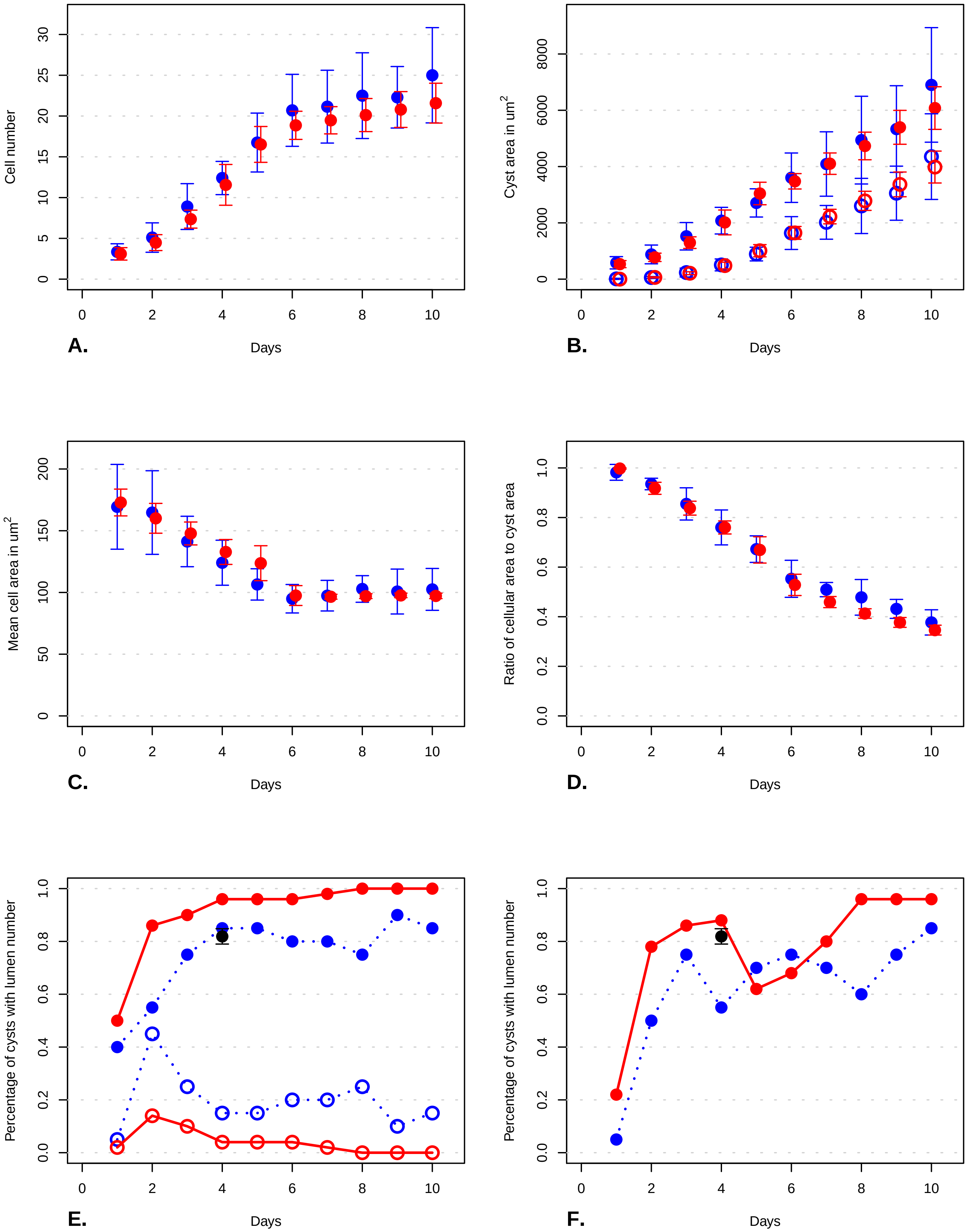

**Figure S11−27) Varied stableCycleDelay: scd = 0.95**

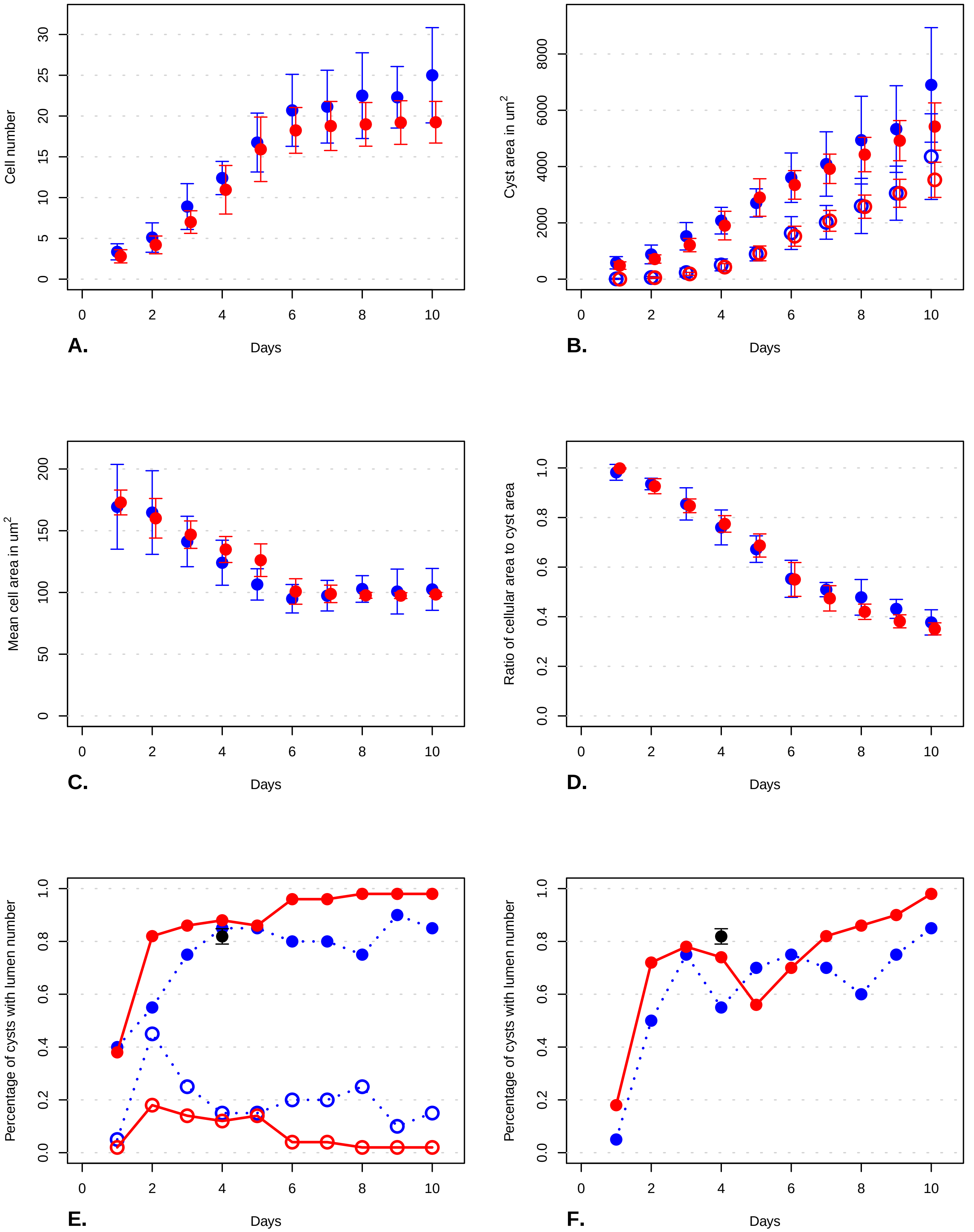

## Figure S11−28) Varied lambdaPerim: lp = .5

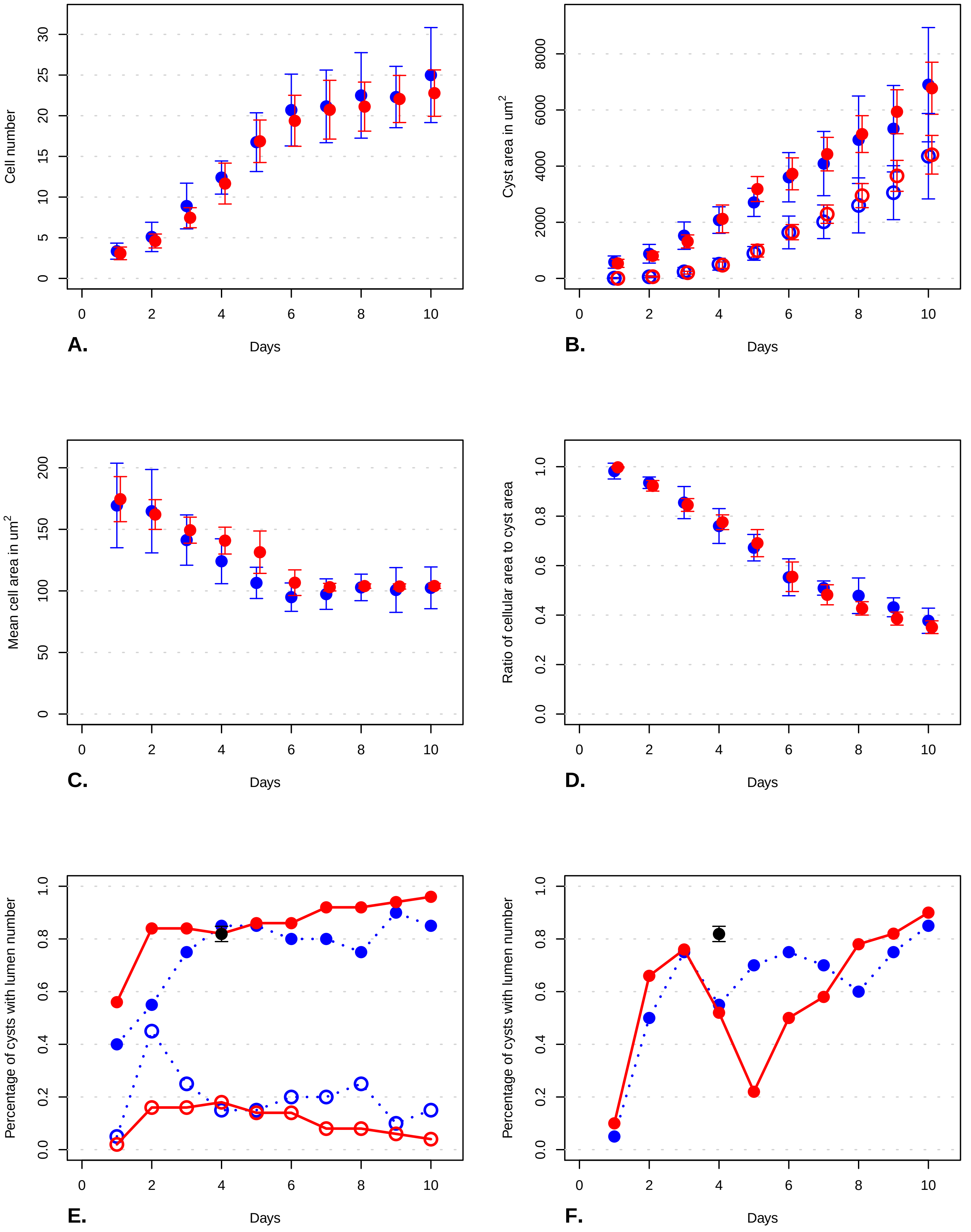

## Figure S11−29) Varied lambdaPerim: lp = 1.25

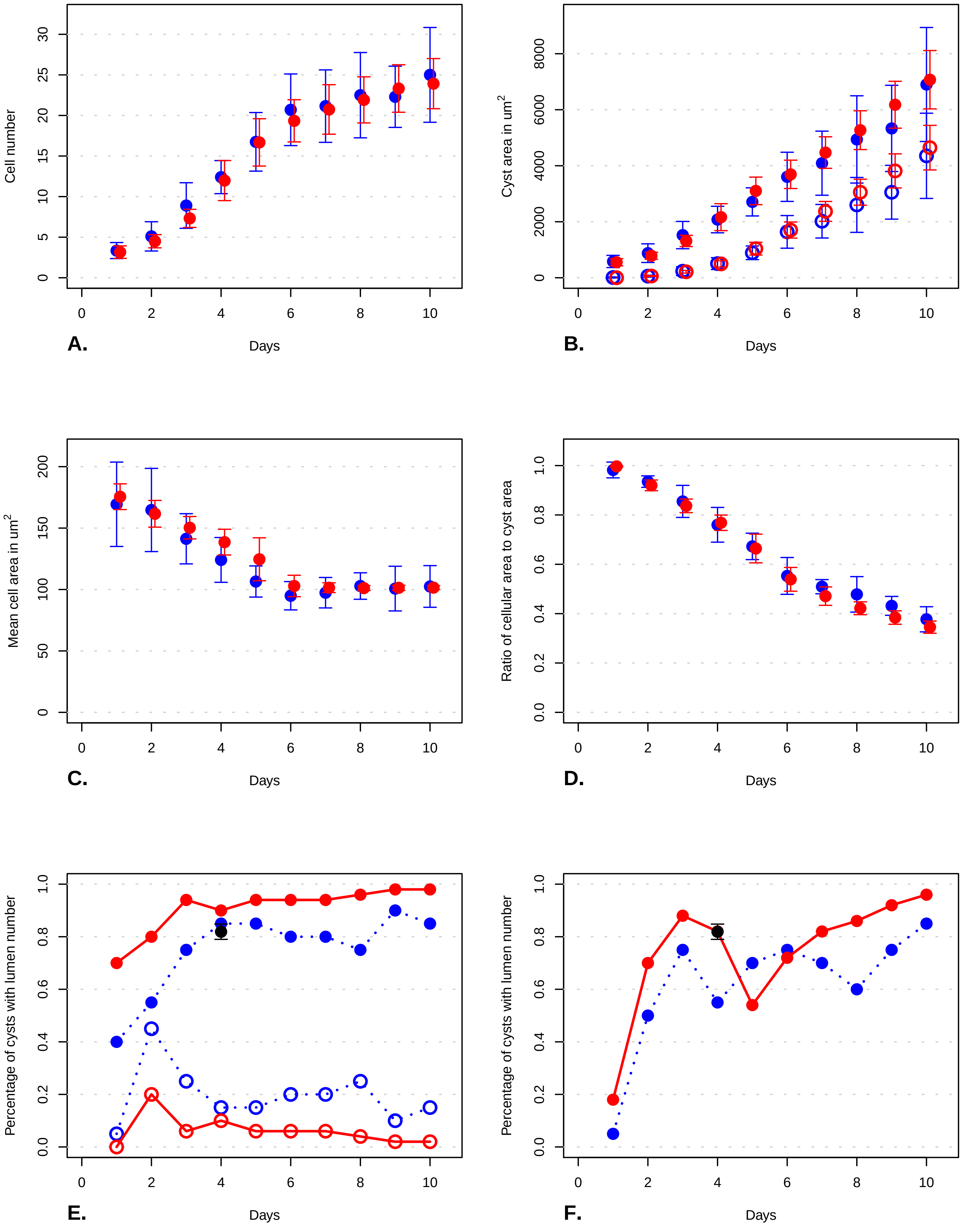

Figure S11−30) Varied lambdaPerim: lp = 5

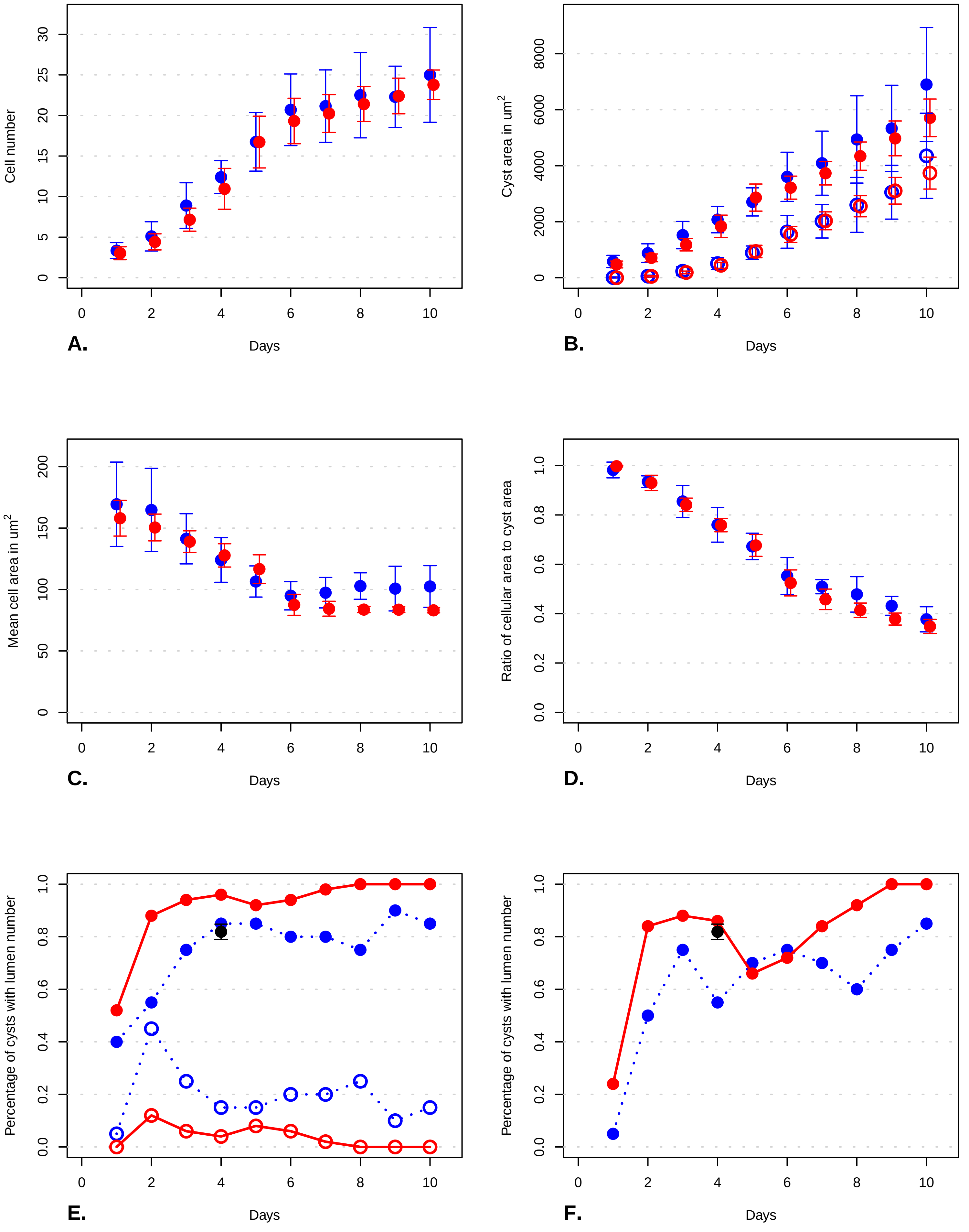

**Figure S11−31) Varied lambdaPerim: lp = 10**

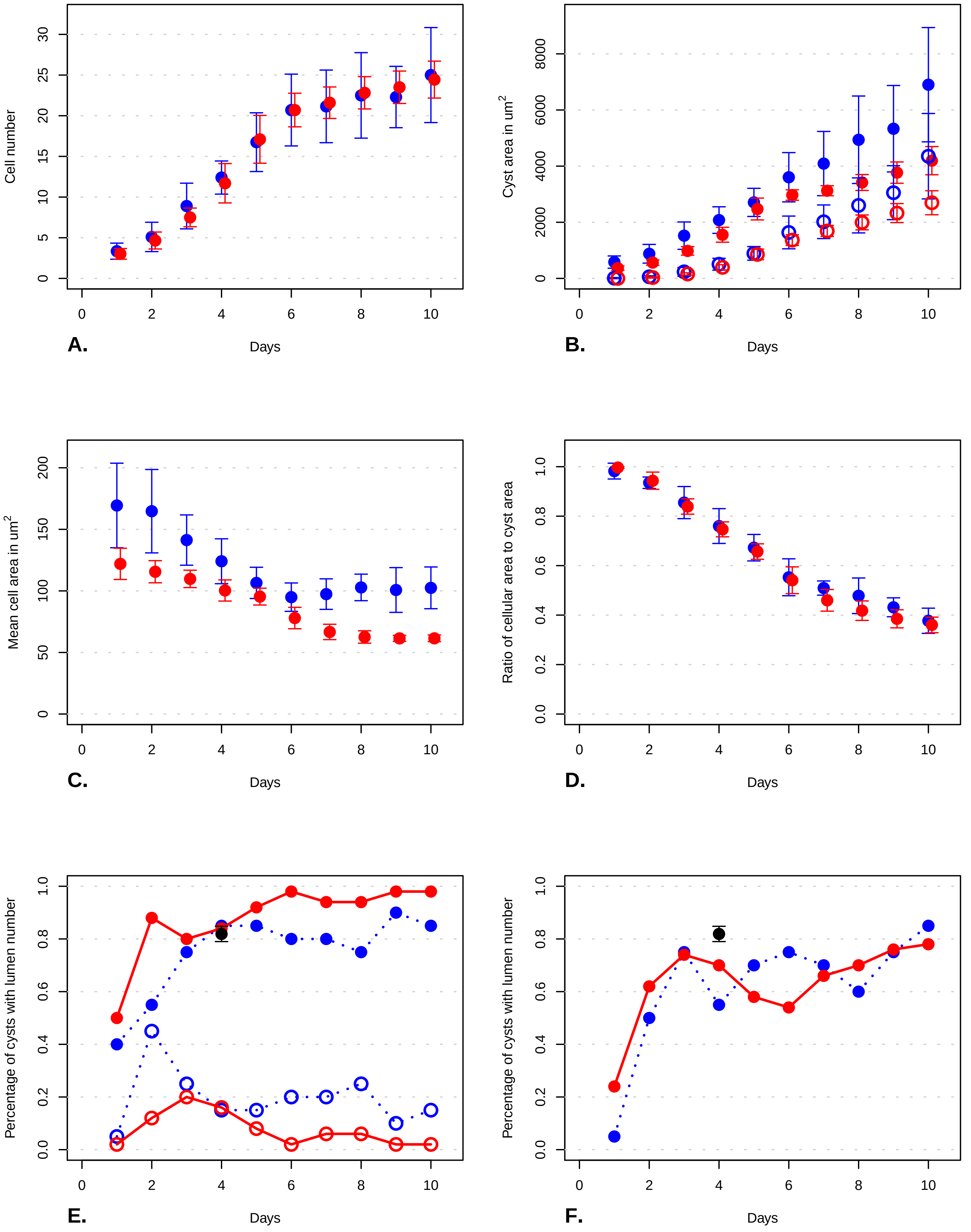

## Figure S11−32) Varied polarDelay: pd = 10

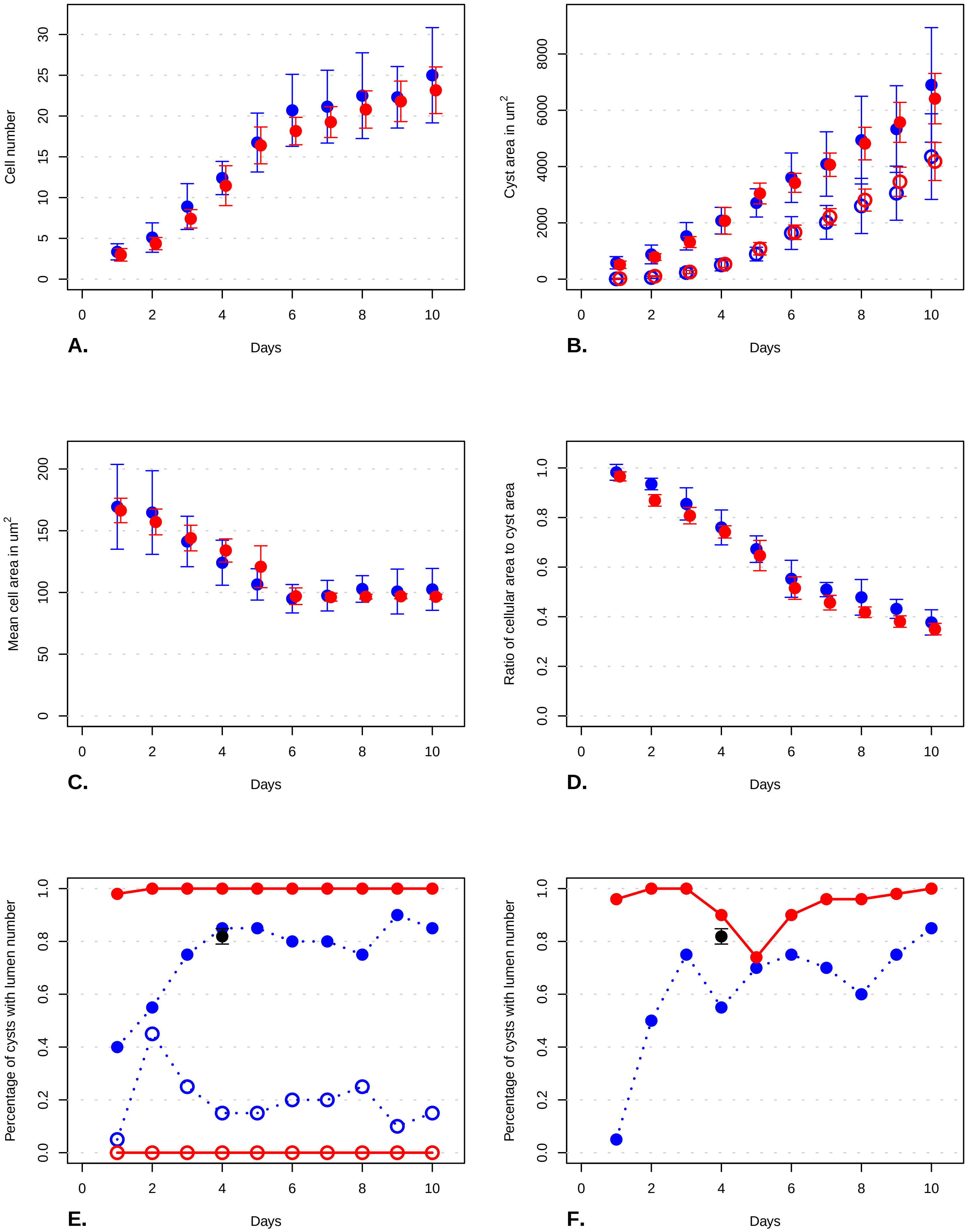

## Figure S11−33) Varied polarDelay: pd = 21

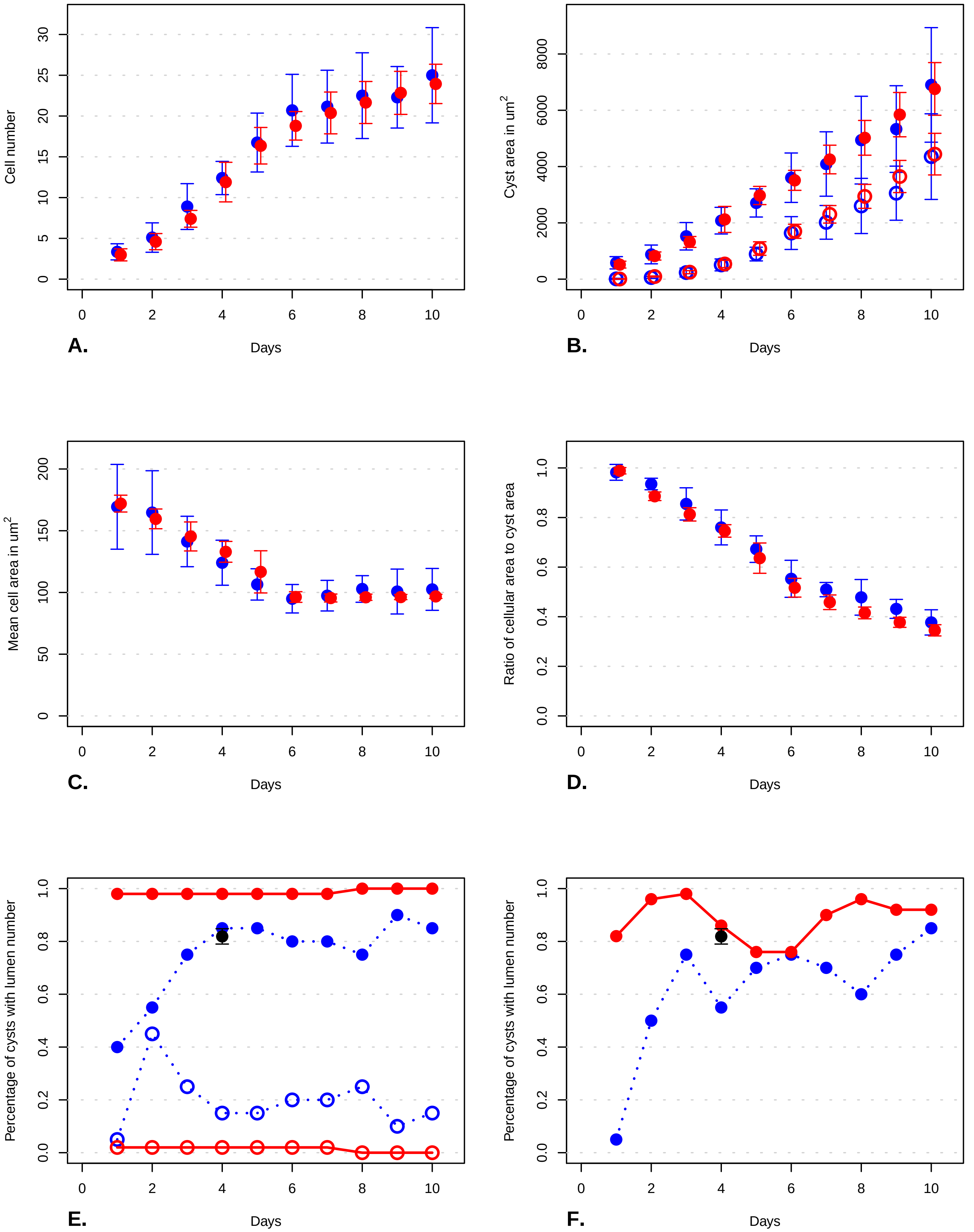

## Figure S11−34) Varied polarDelay: pd = 84

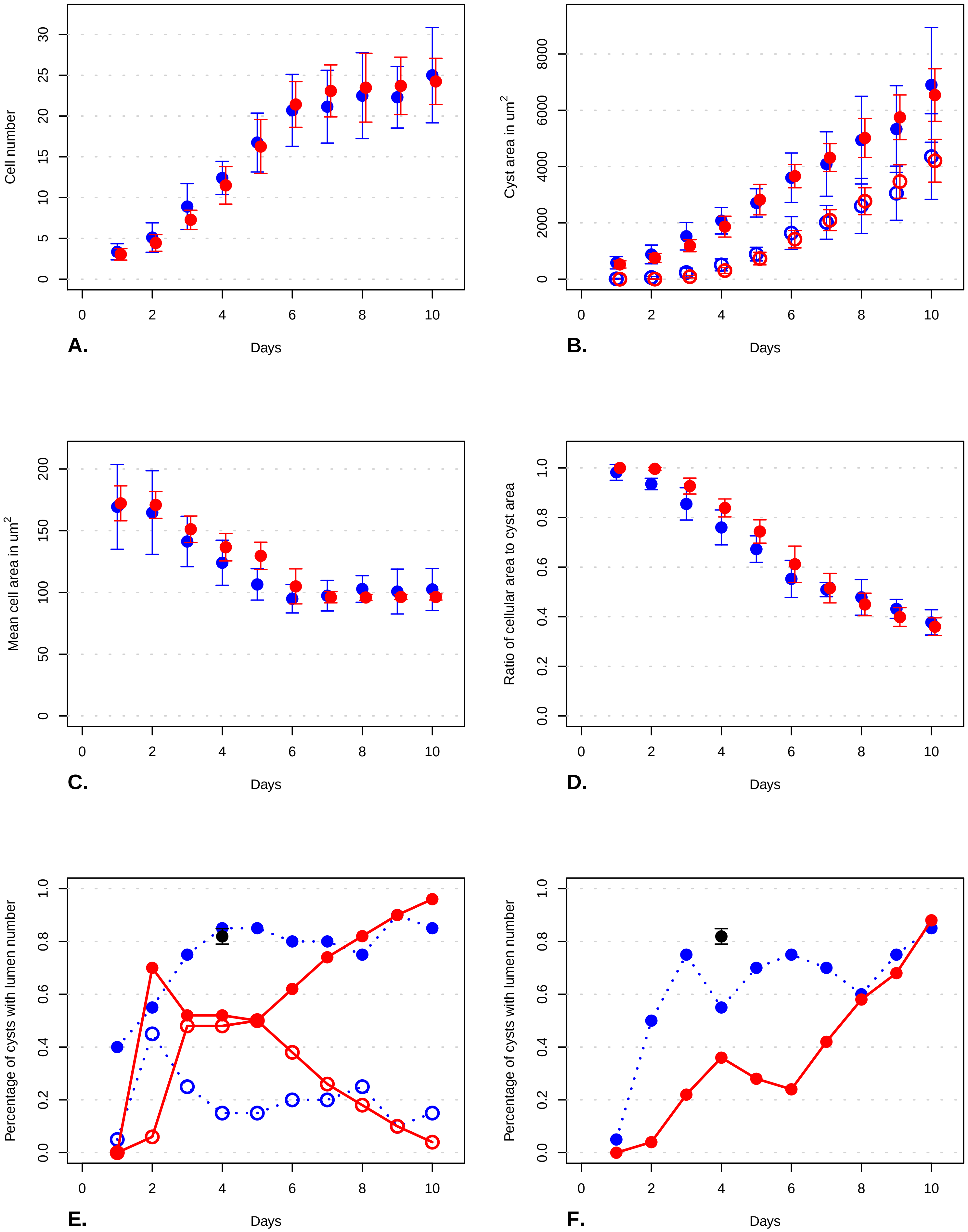

## Figure S11−35) Varied polarDelay: pd = 200

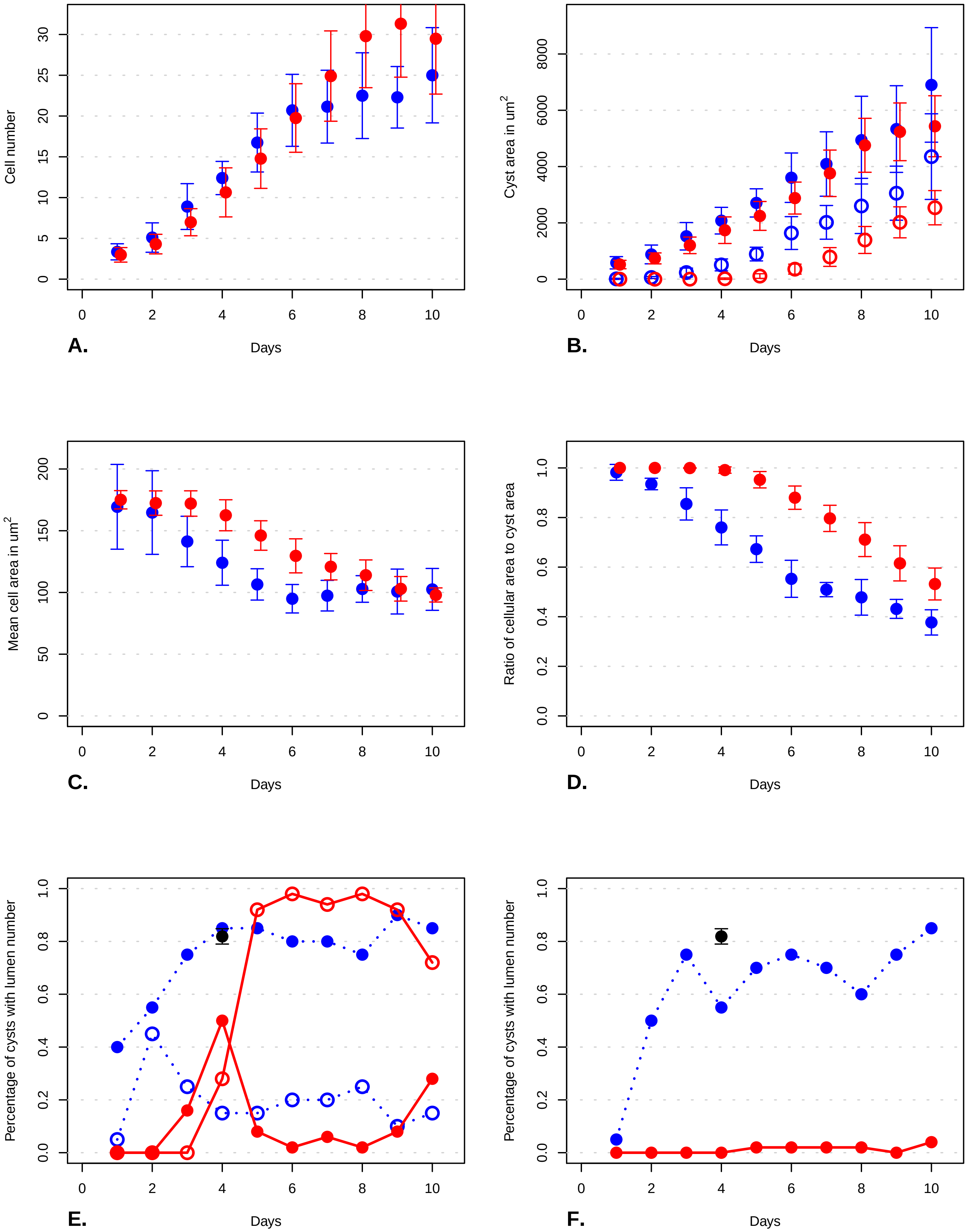

## Figure S11−36) Varied shiftDelay with stableRatio = 100000: sd = 50

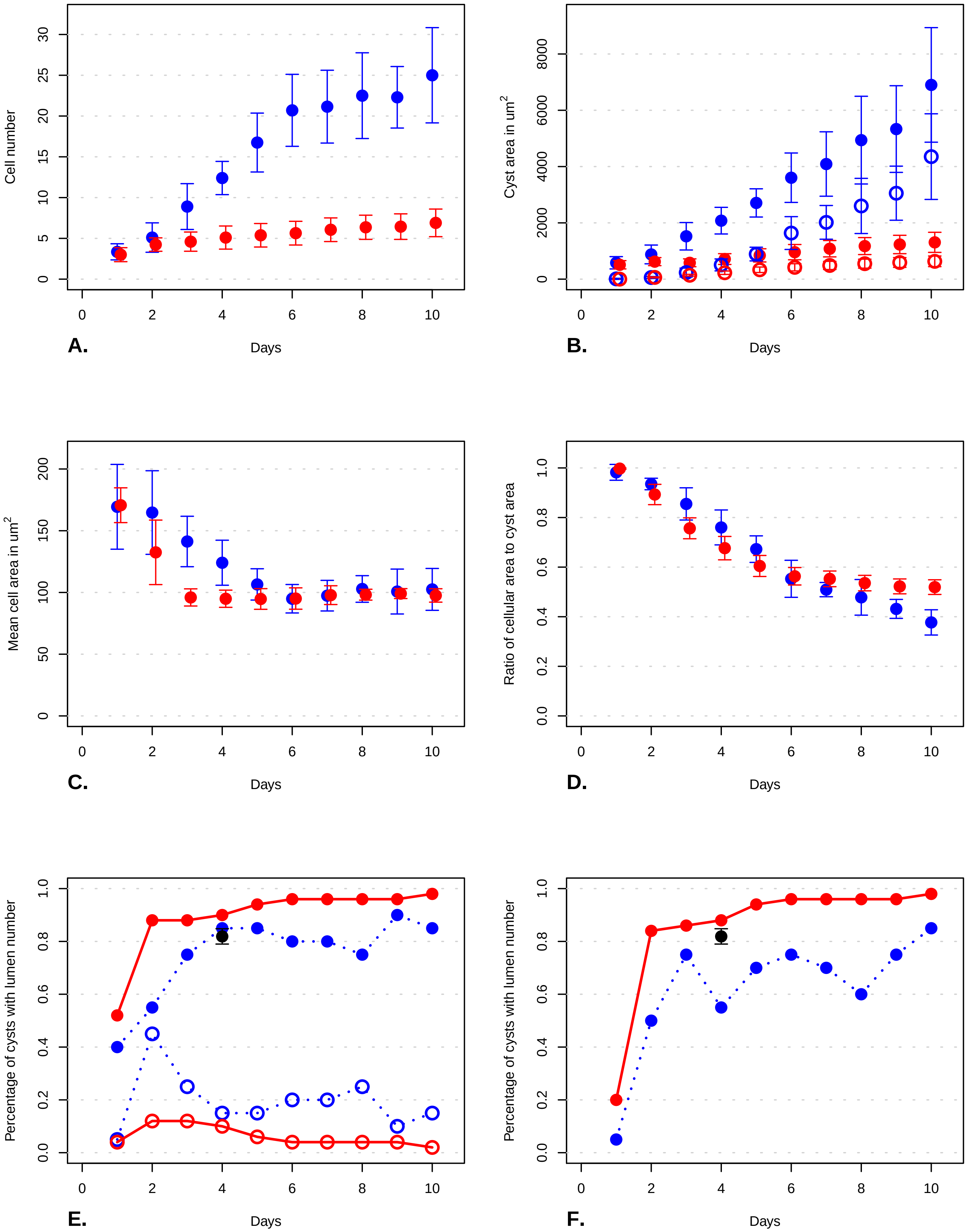

## Figure S11−37) Varied shiftDelay with stableRatio = 100000: sd = 100

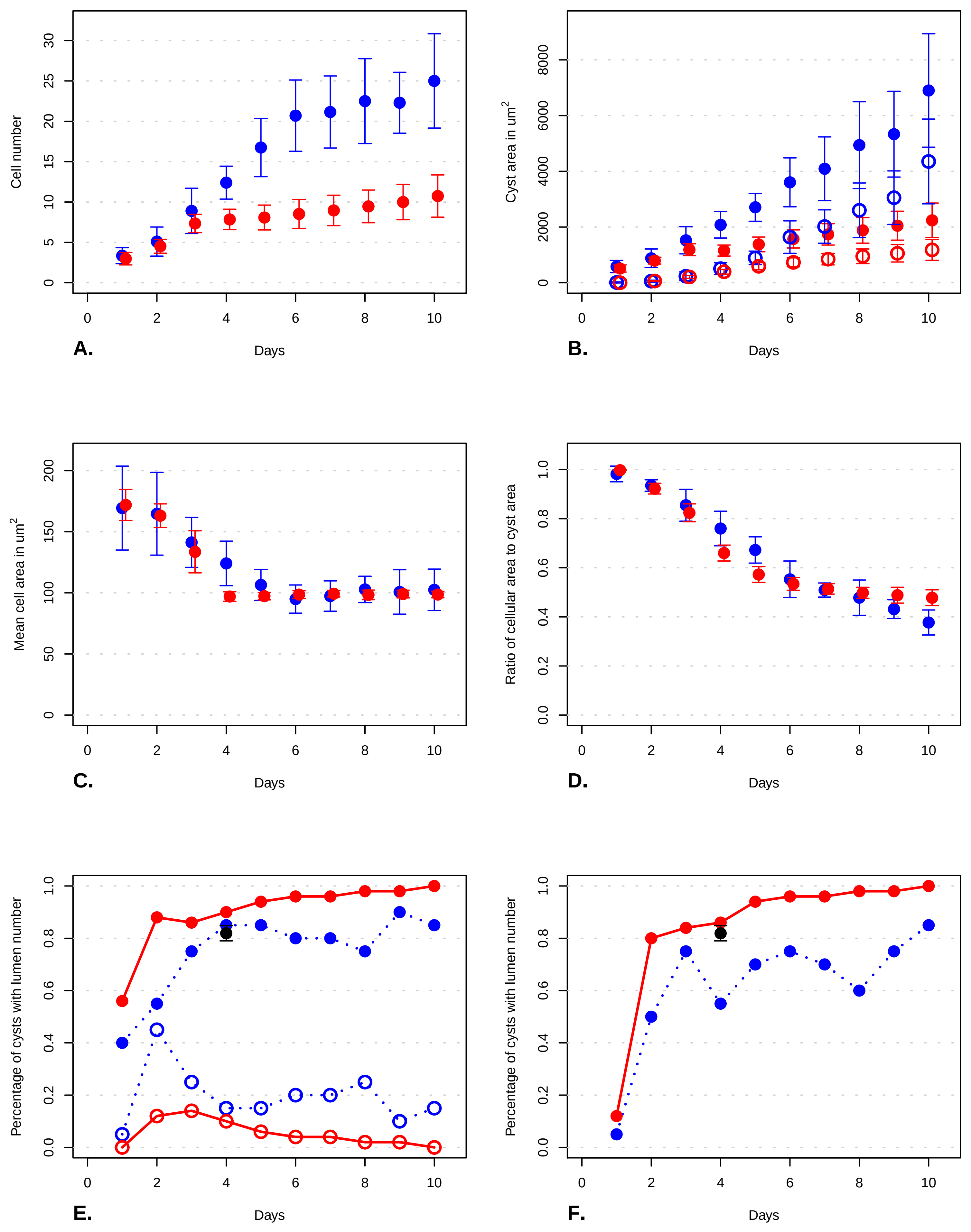

## Figure S11−38) Varied shiftDelay with stableRatio = 100000: sd = 250

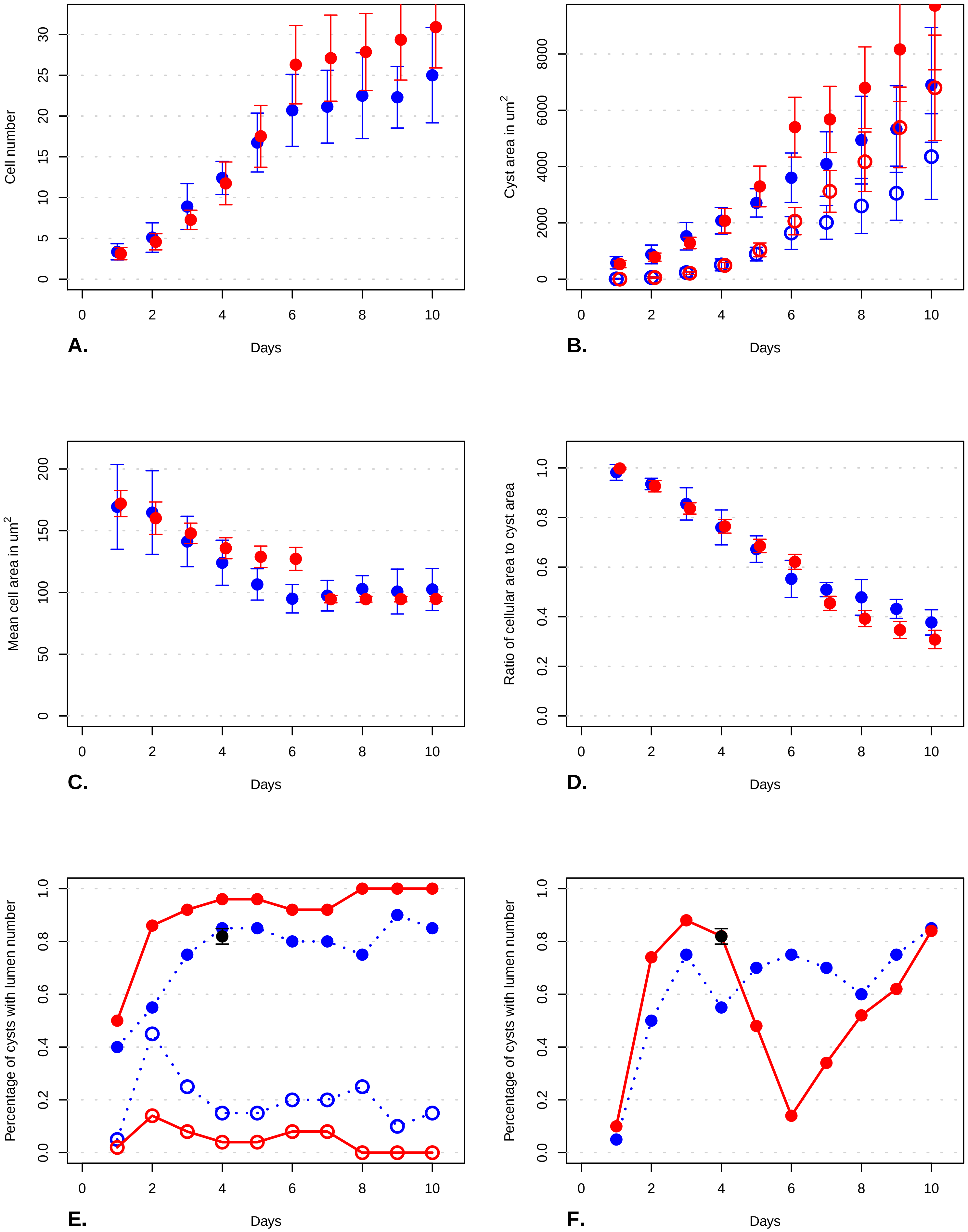

## Figure S11−39) Varied shiftDelay with stableRatio = 100000: sd = 300

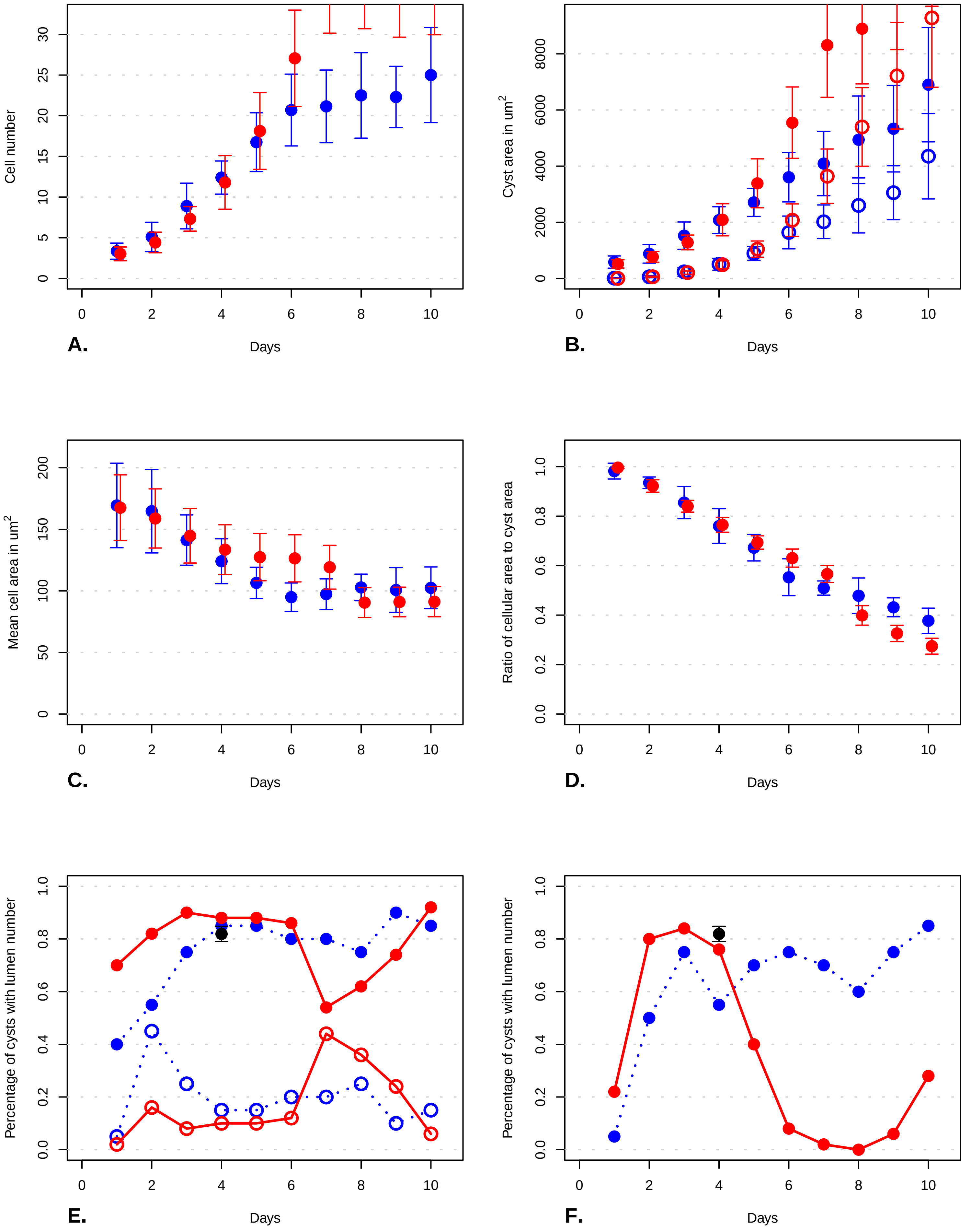

## Figure S11−40) Varied lgrSubtract: lgrs = 14

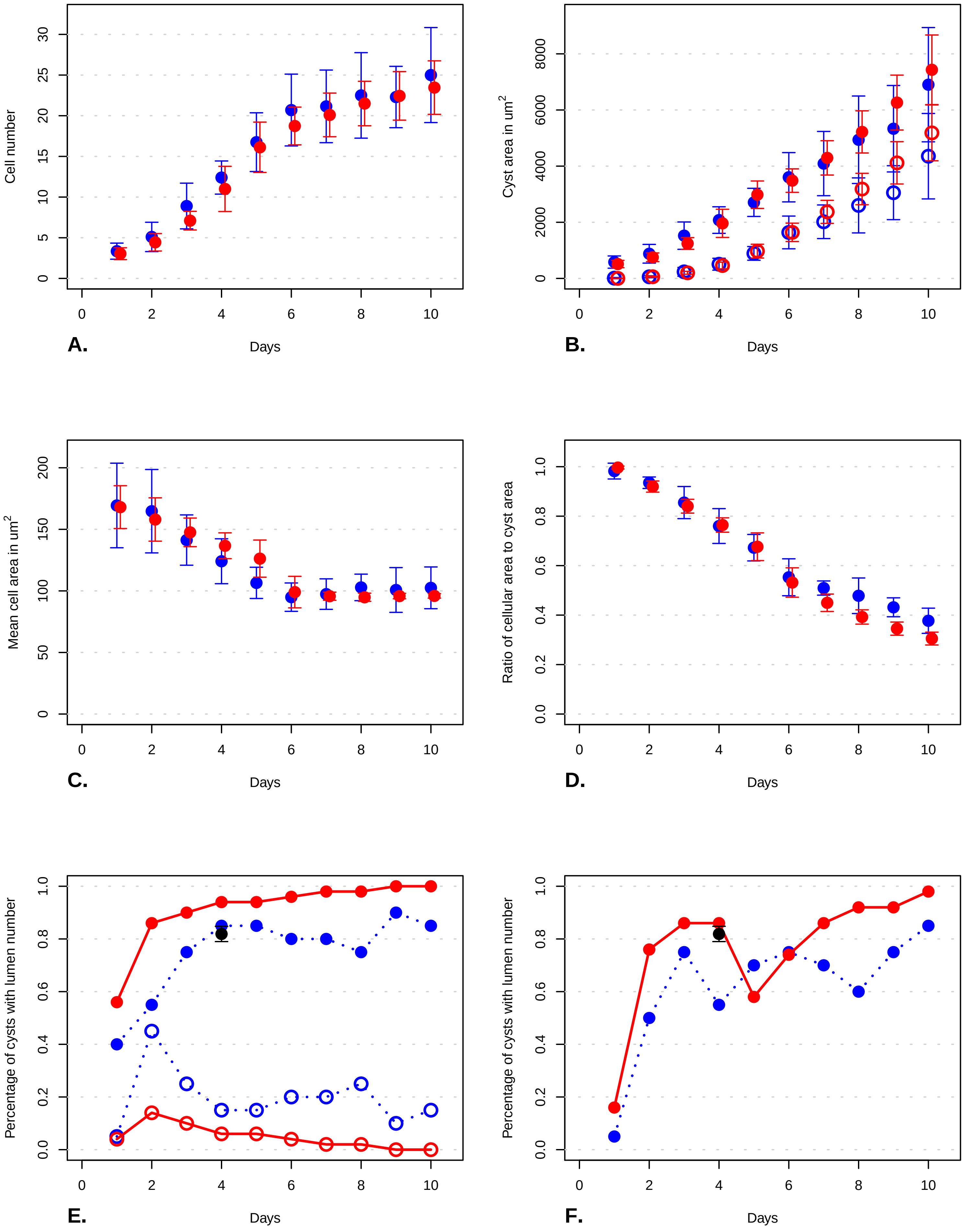

**Figure S11−41) Varied lgrSubtract: lgrs = 20**

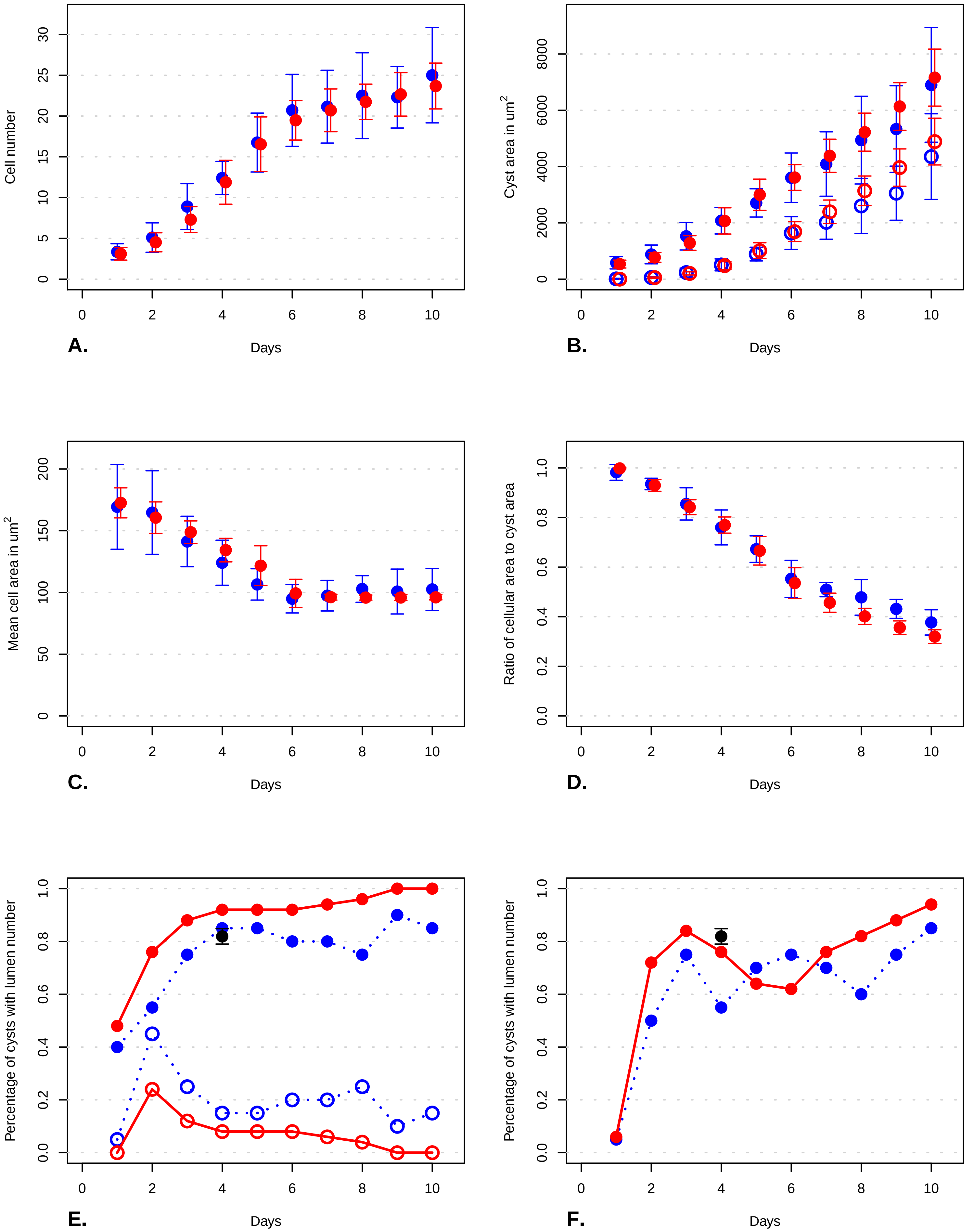

## Figure S11−42) Varied lgrSubtract: lgrs = 40

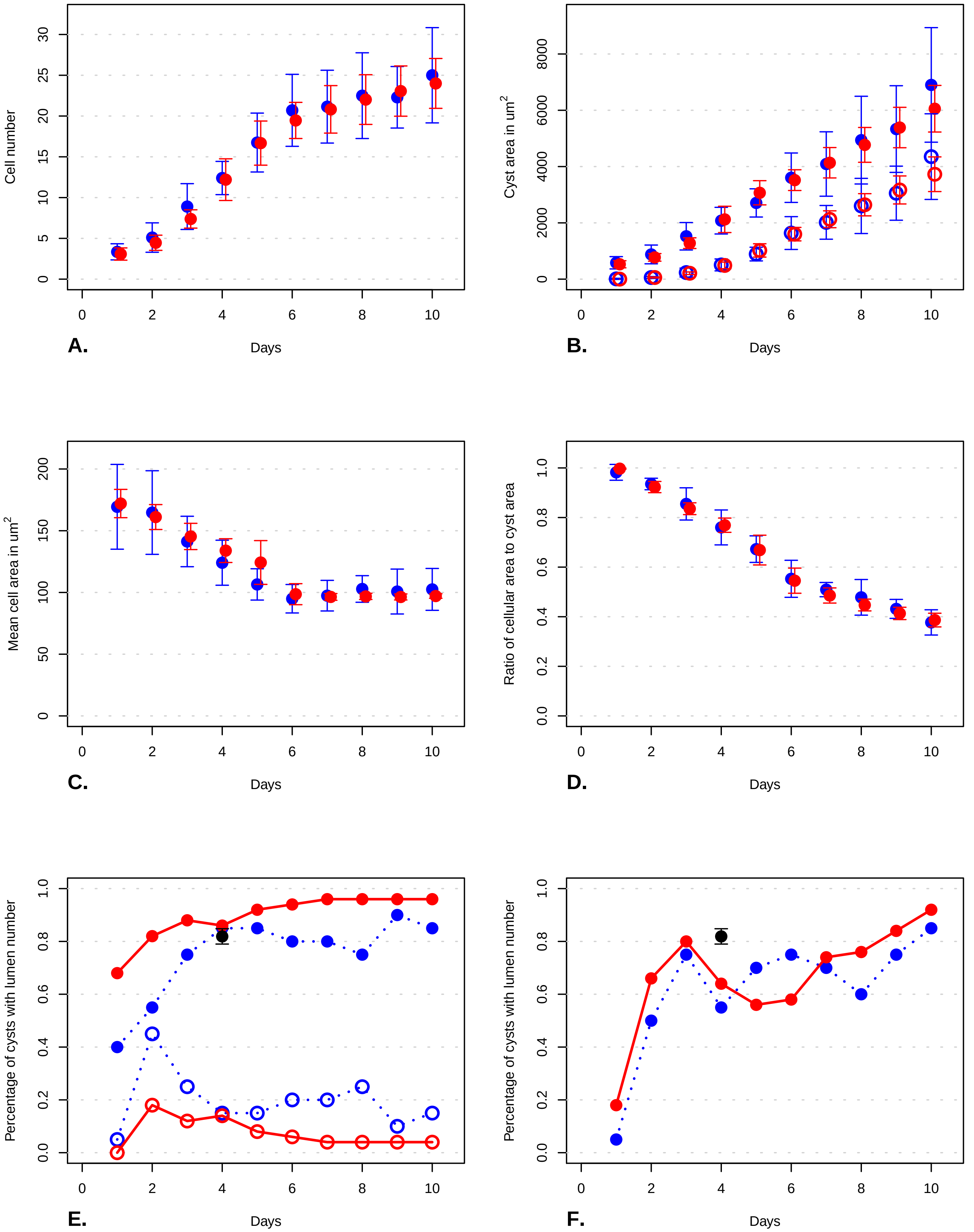

## Figure S11−43) Varied lgrSubtract: lgrs = 54

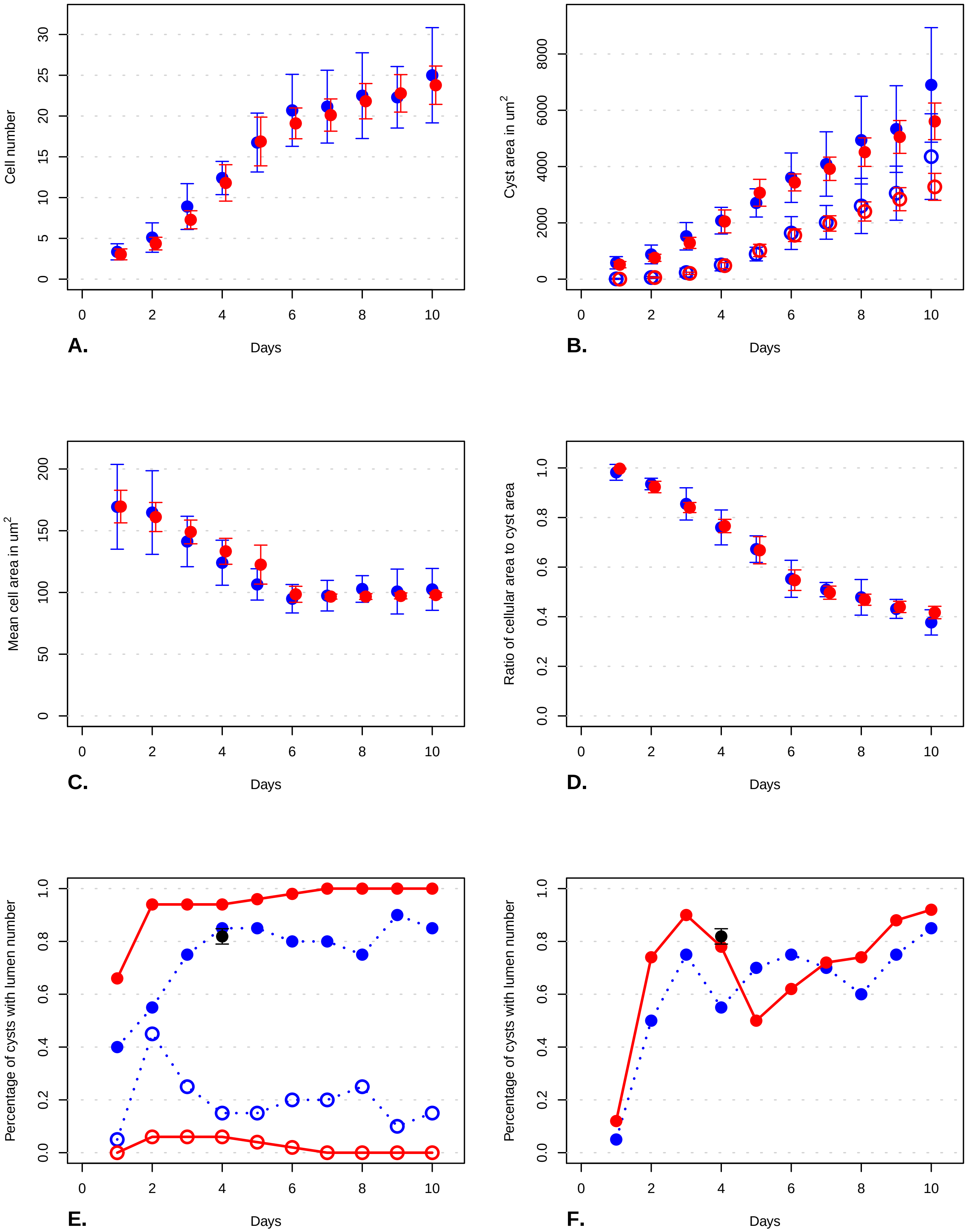

# Figure S11−44) Varied lgrSubtract: lgrs = 75

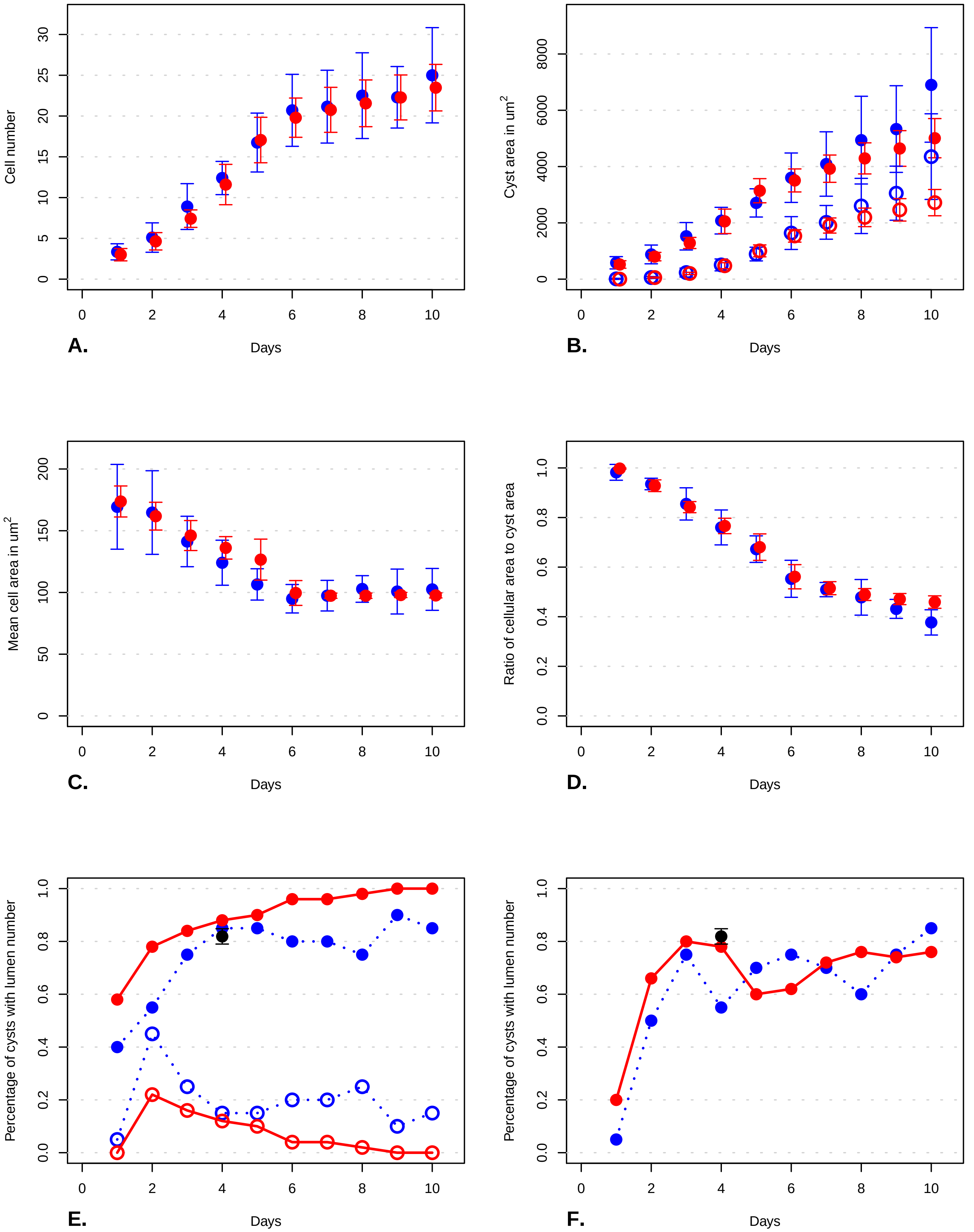

## Figure S11−45) Varied doublingArea: da = 20

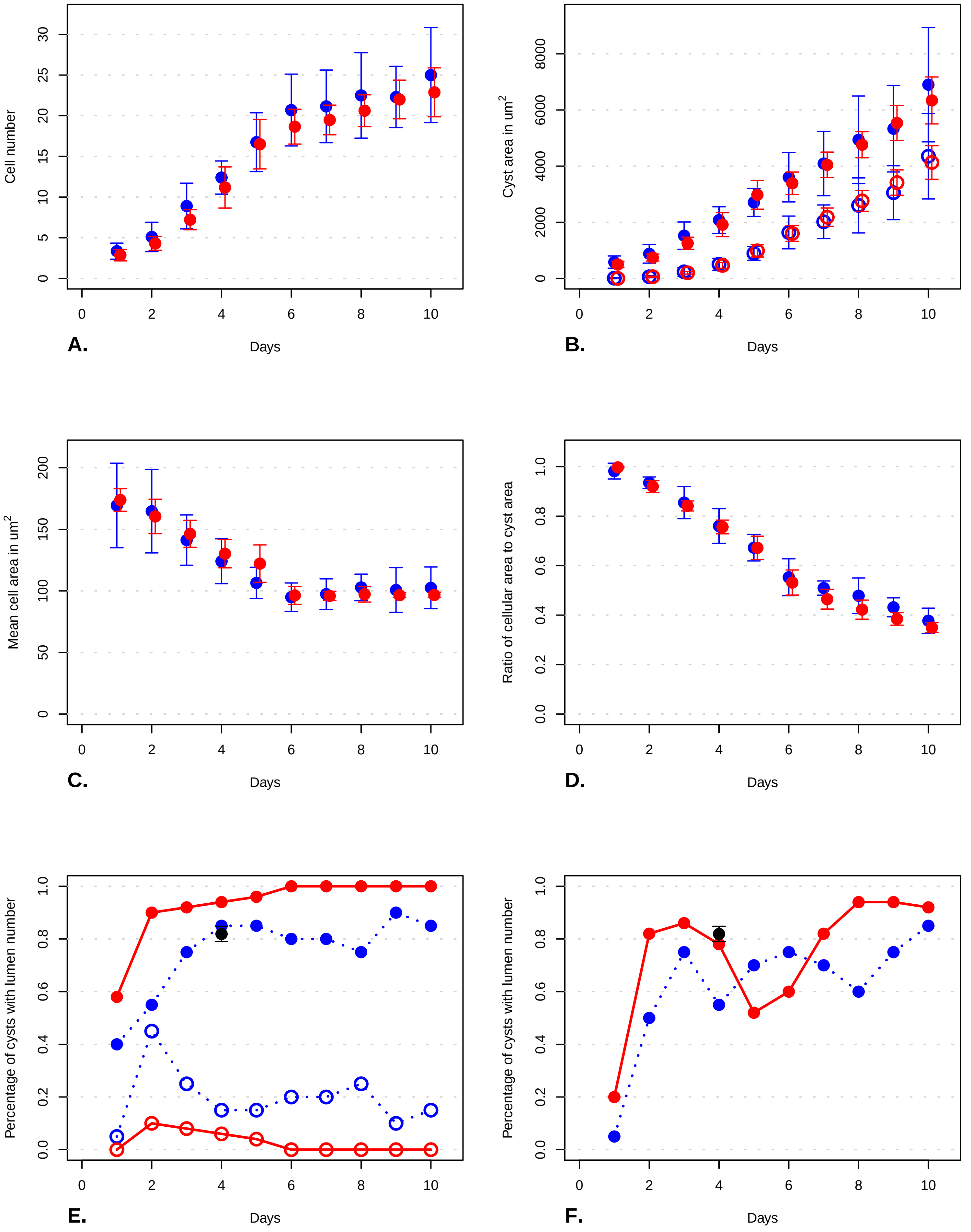

Figure S11−46) Varied doublingArea: da = 30

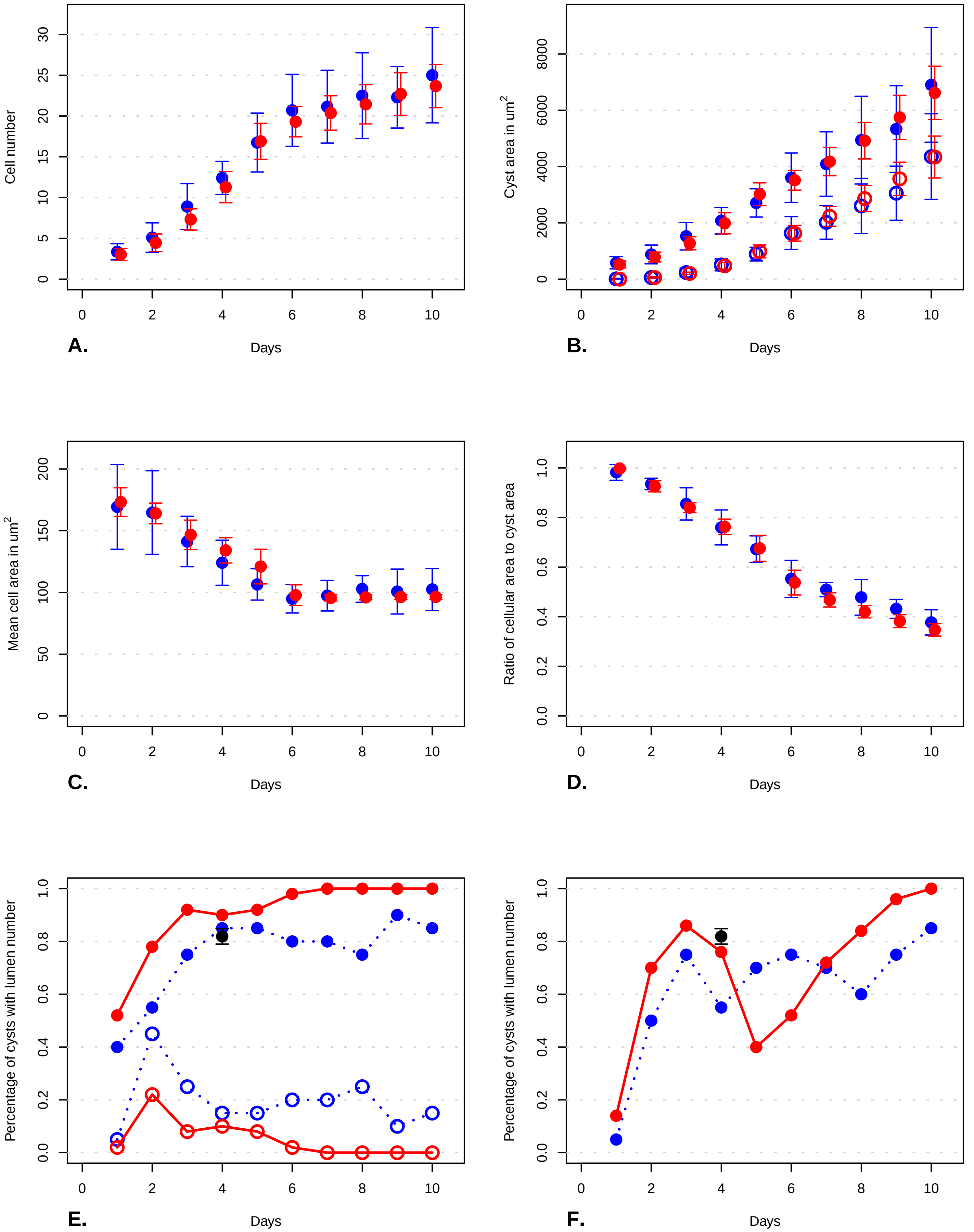

Figure S11−47) Varied doublingArea: da = 50

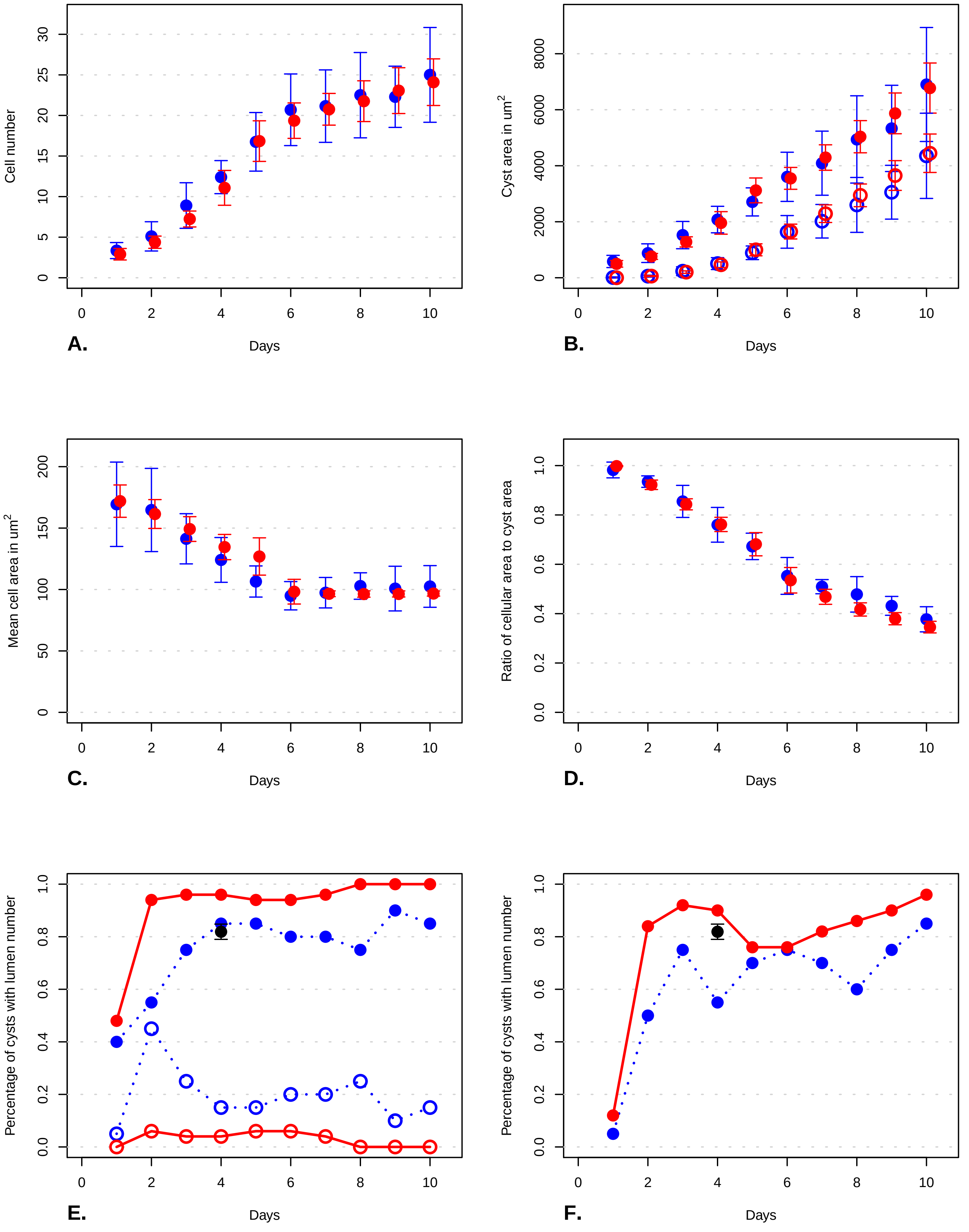

**Figure S11−48) Varied doublingArea: da = 80**

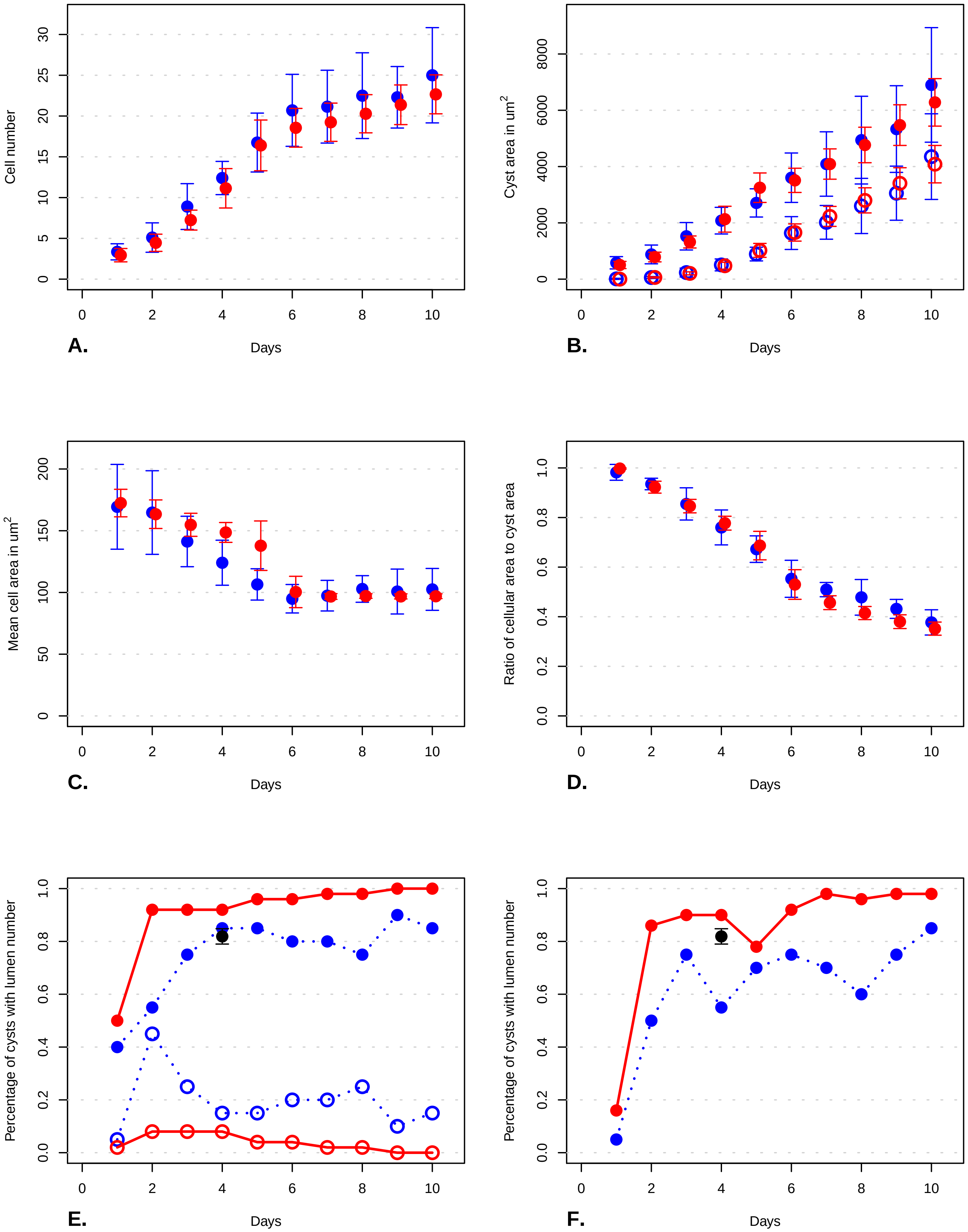

## Figure S11−49) Varied multiplier: m = 0.3

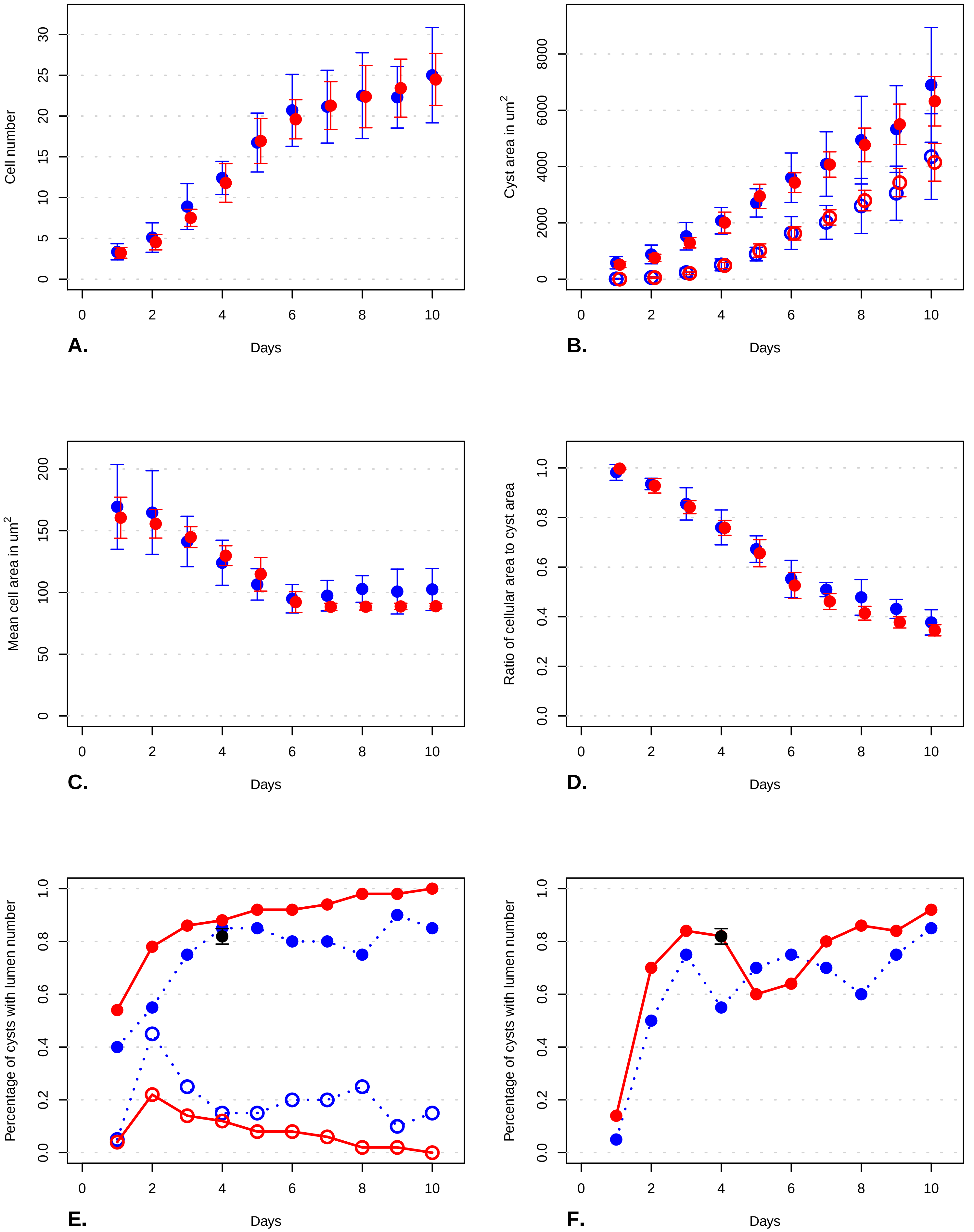

## Figure S11−50) Varied multiplier: m = 0.5

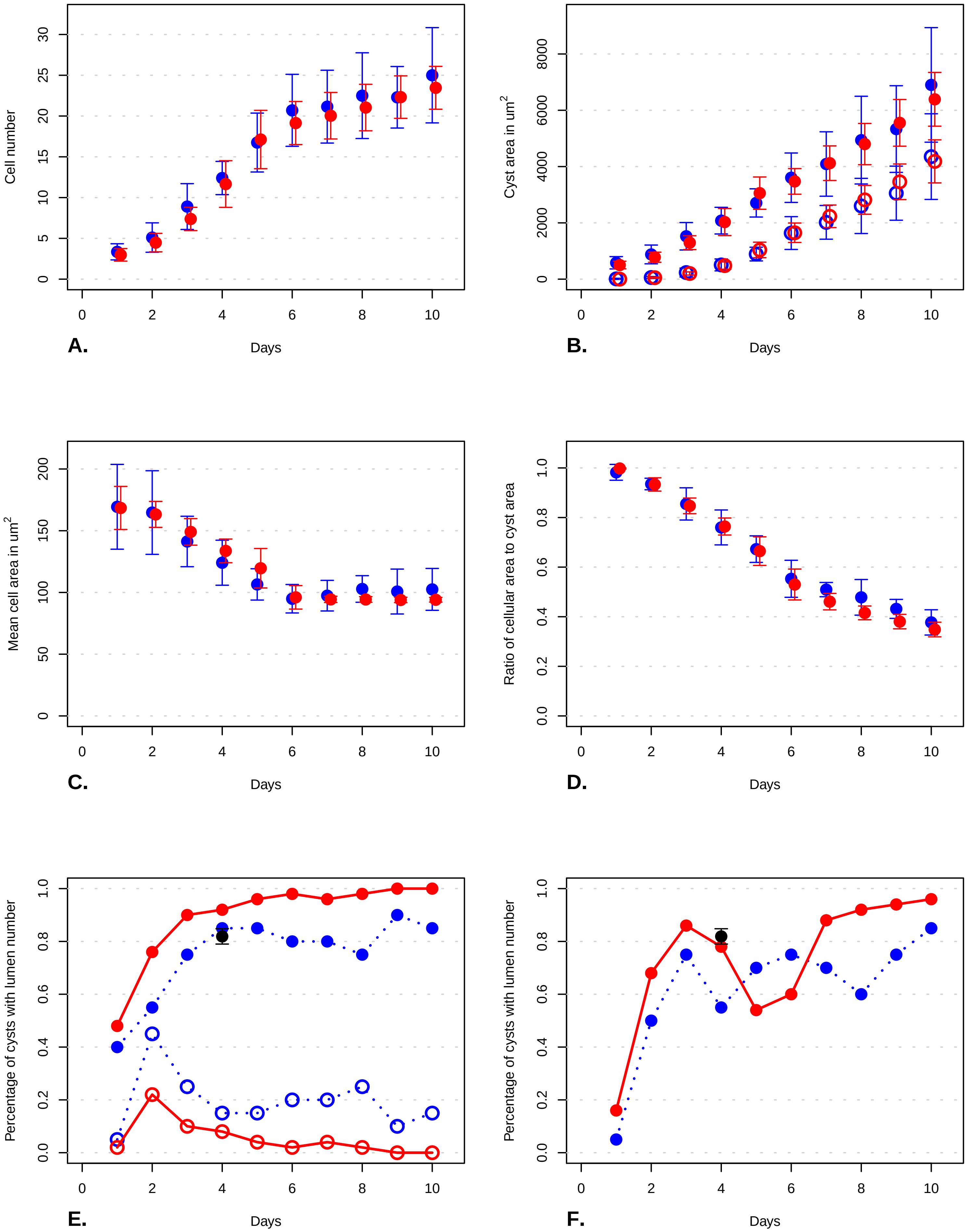

## Figure S11−51) Varied multiplier: m = 0.7

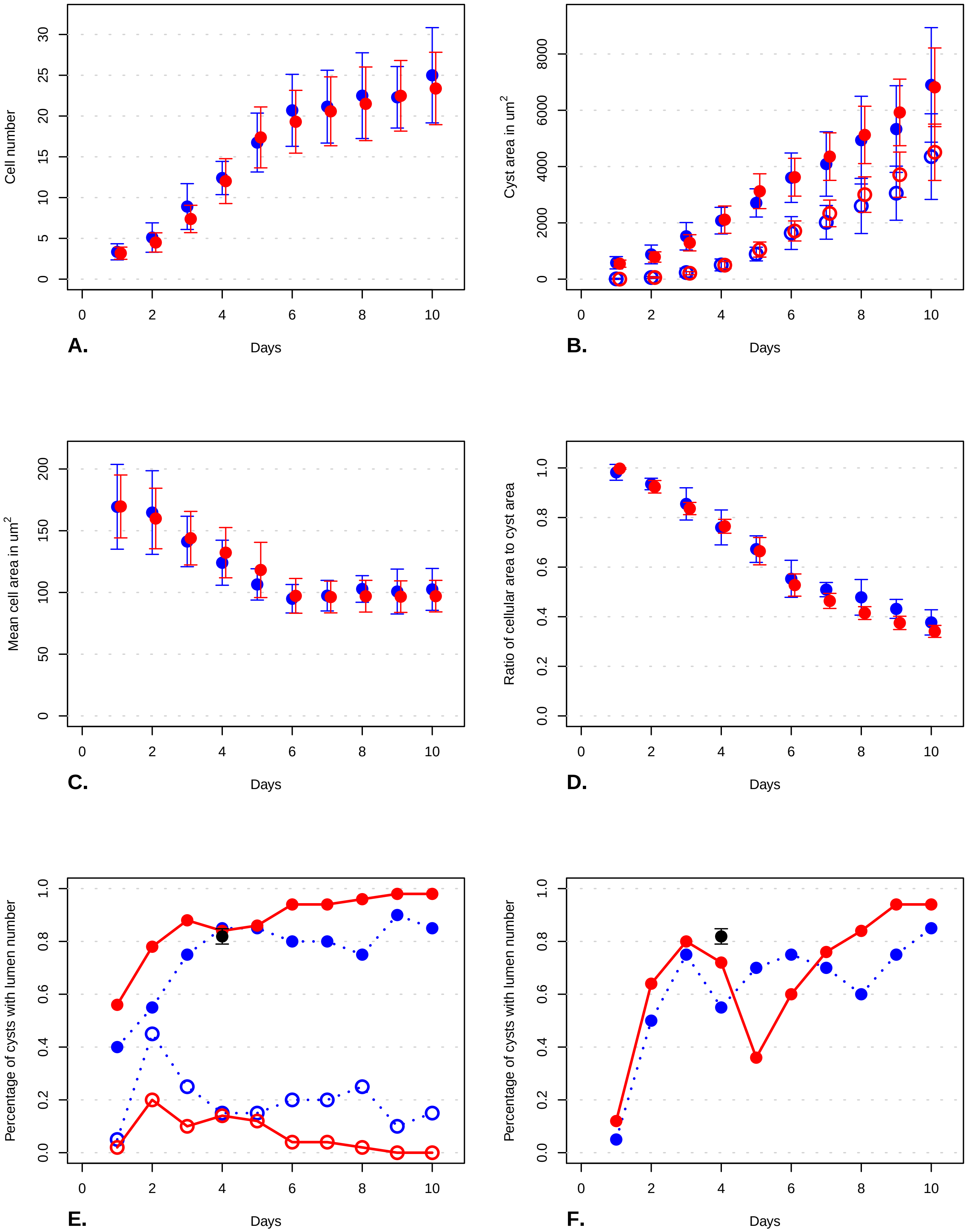

## Figure S11−52) Varied multiplier: m = 0.8

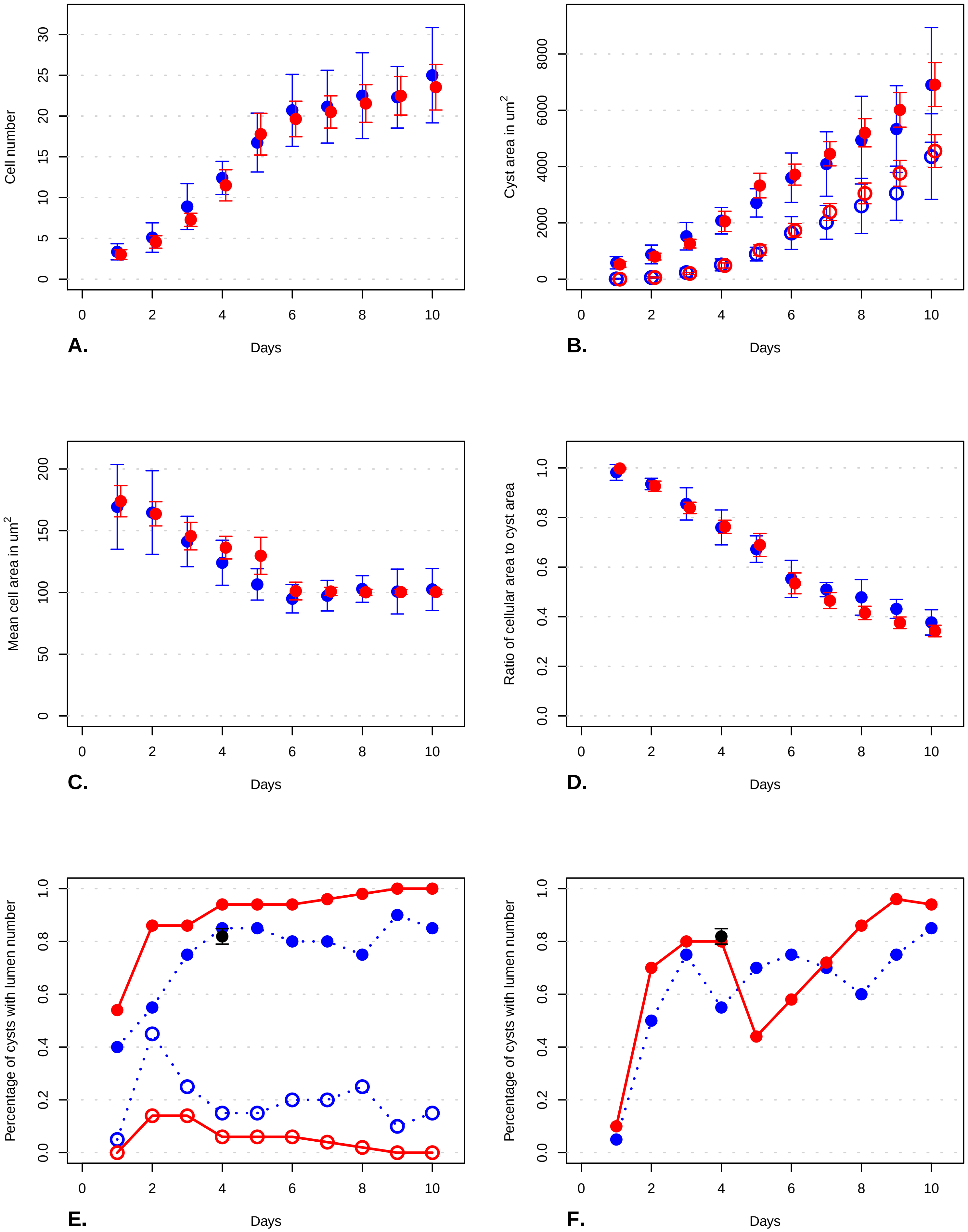

## Figure S11−53) Varied lumenGrowthRate: lgr = 0

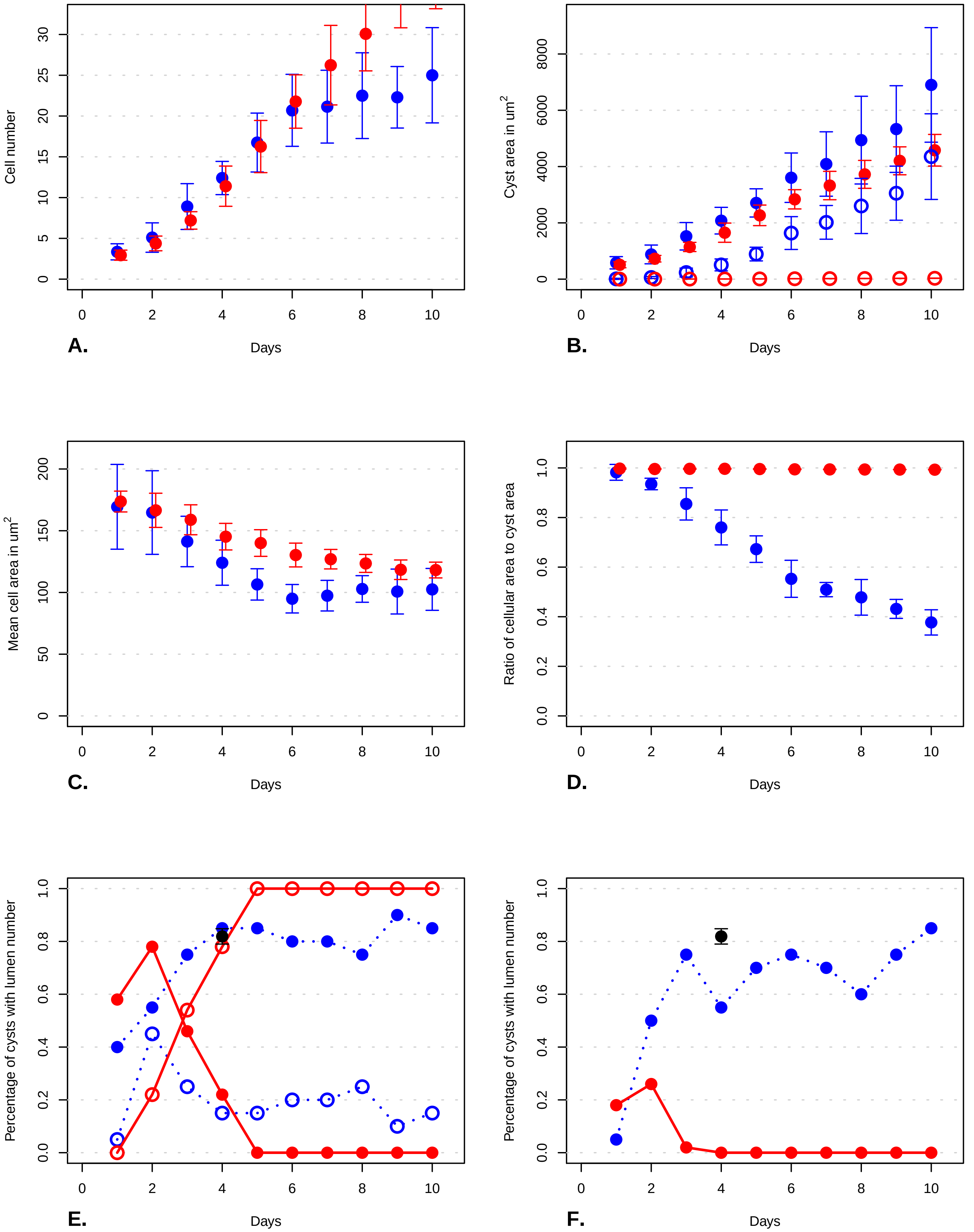

**Figure S11−54) Varied lumenGrowthRate: lgr = 0.001**

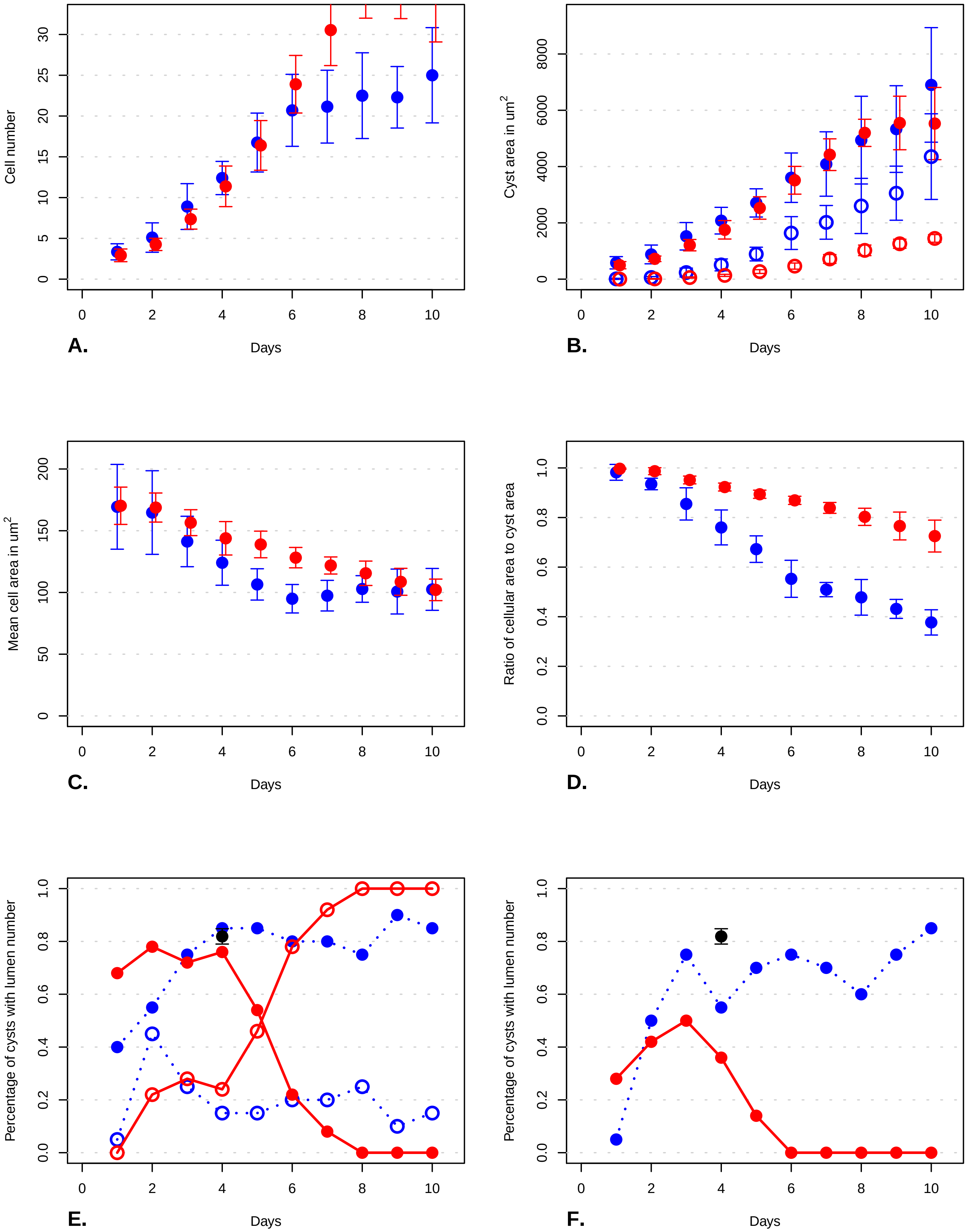

## Figure S11−55) Varied lumenGrowthRate: lgr = 0.002

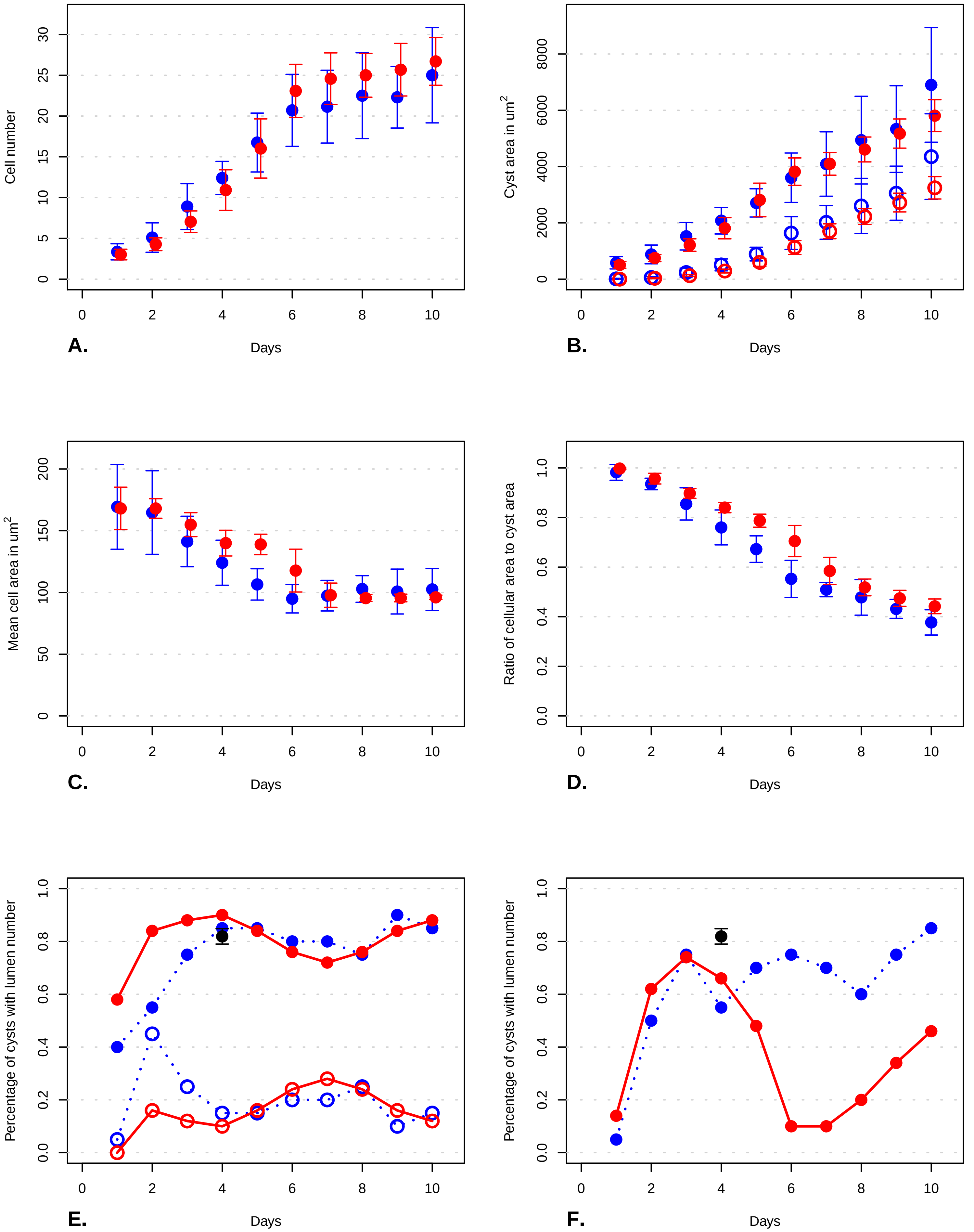

## Figure S11−56) Varied lumenGrowthRate: lgr = 0.0025

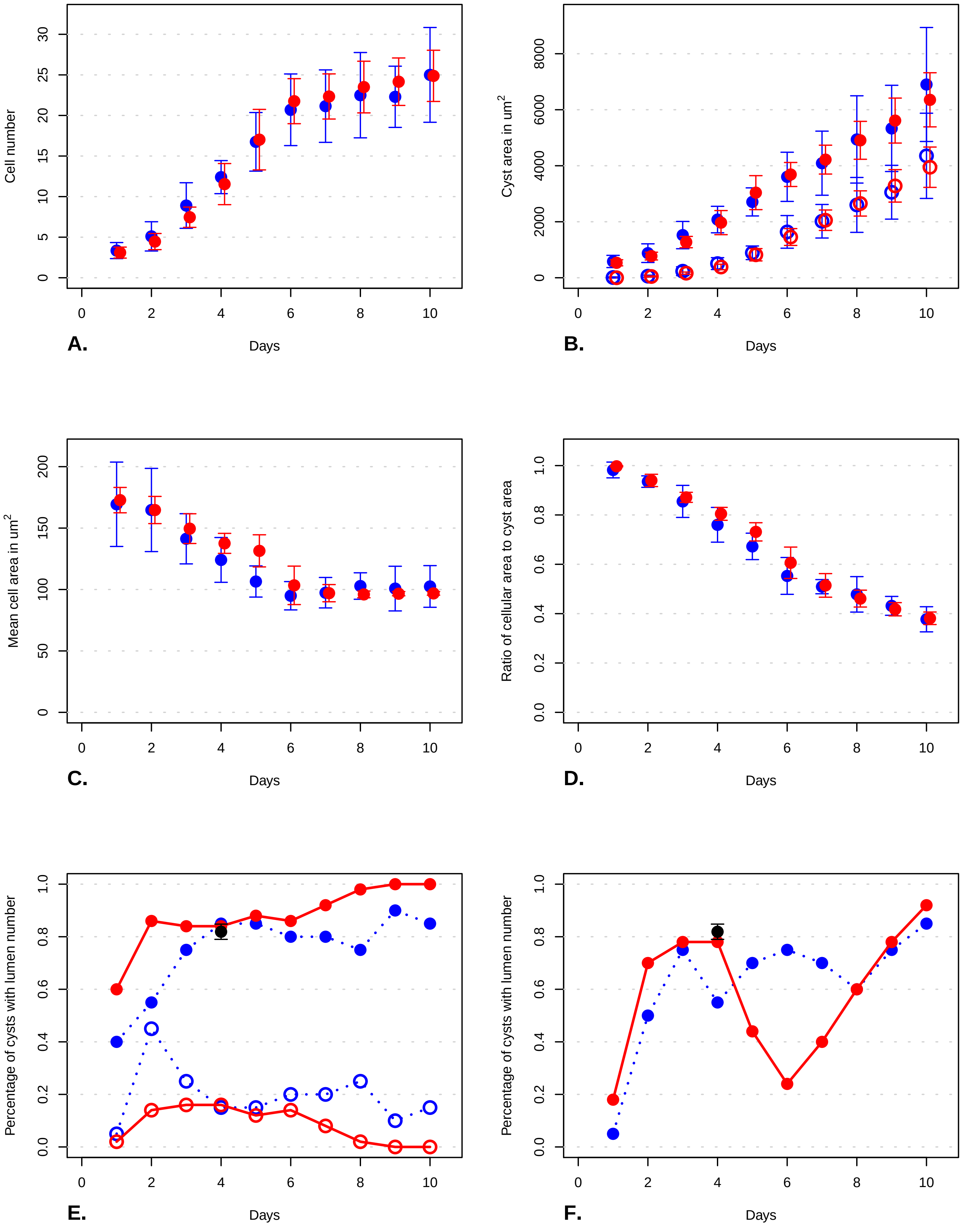

Figure S11−57) Varied lumenGrowthRate: lgr = 0.0035

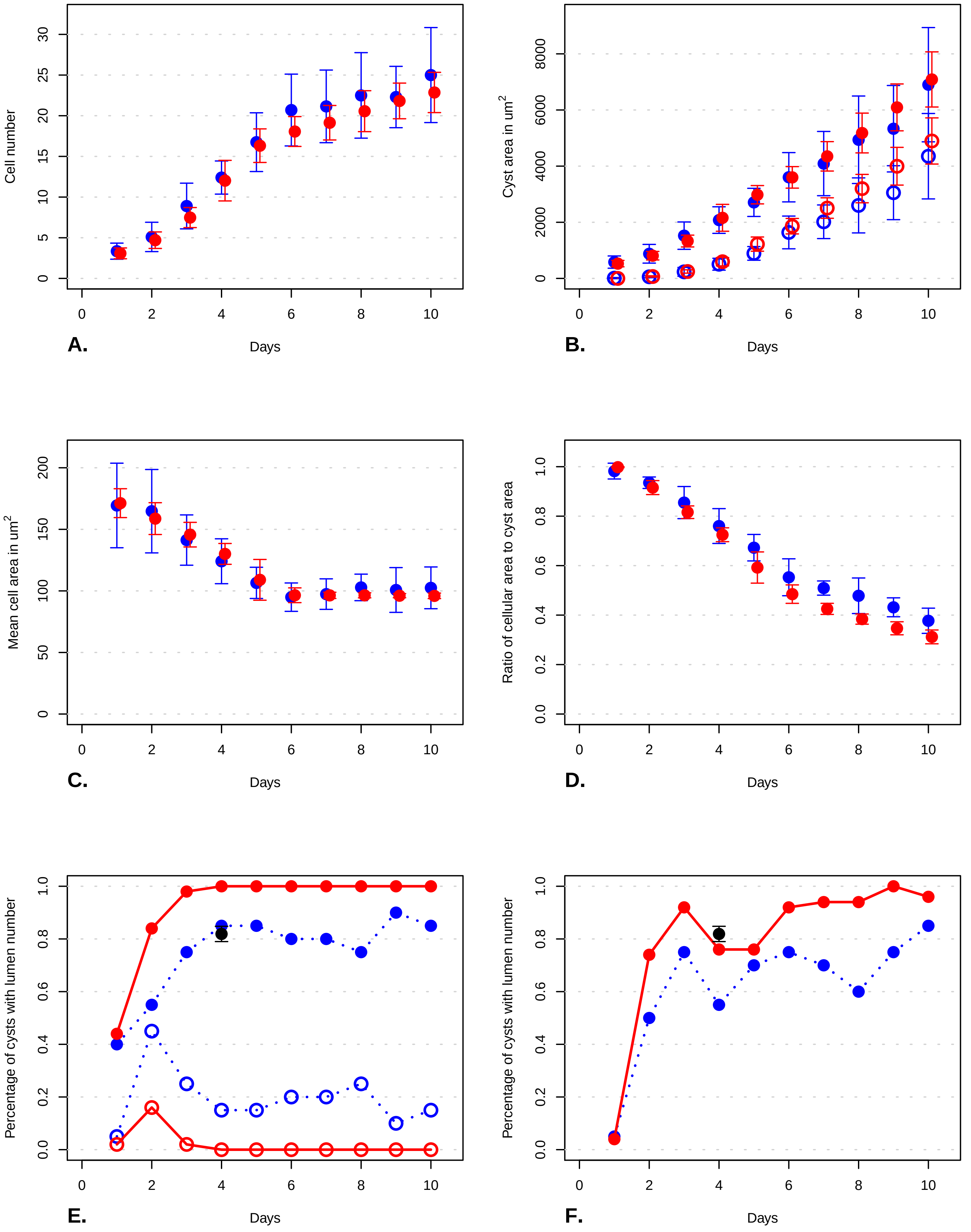

## Figure S11−58) Varied lumenGrowthRate: lgr = 0.004

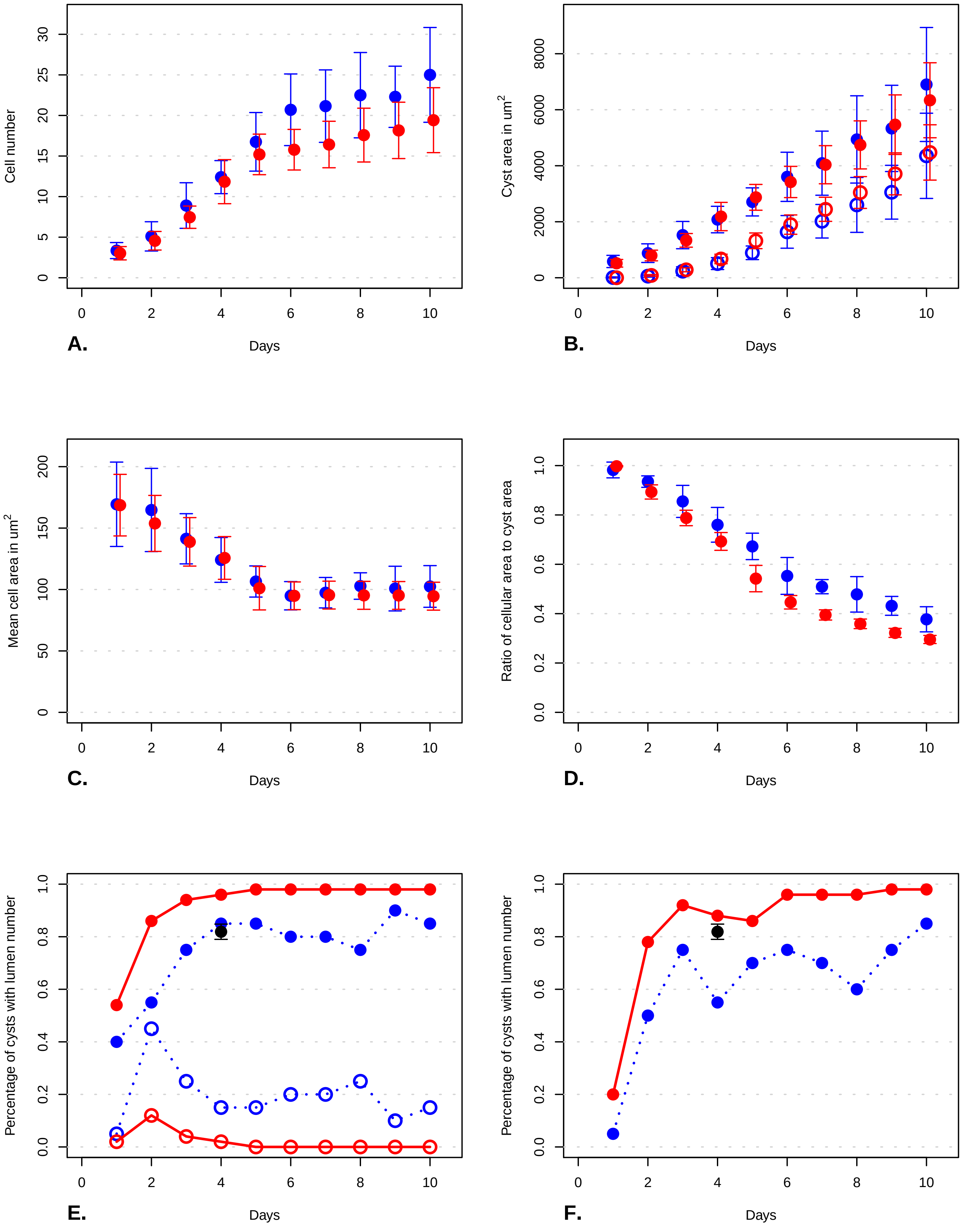

## Figure S11−59) Varied lumenGrowthRate: lgr = 0.005

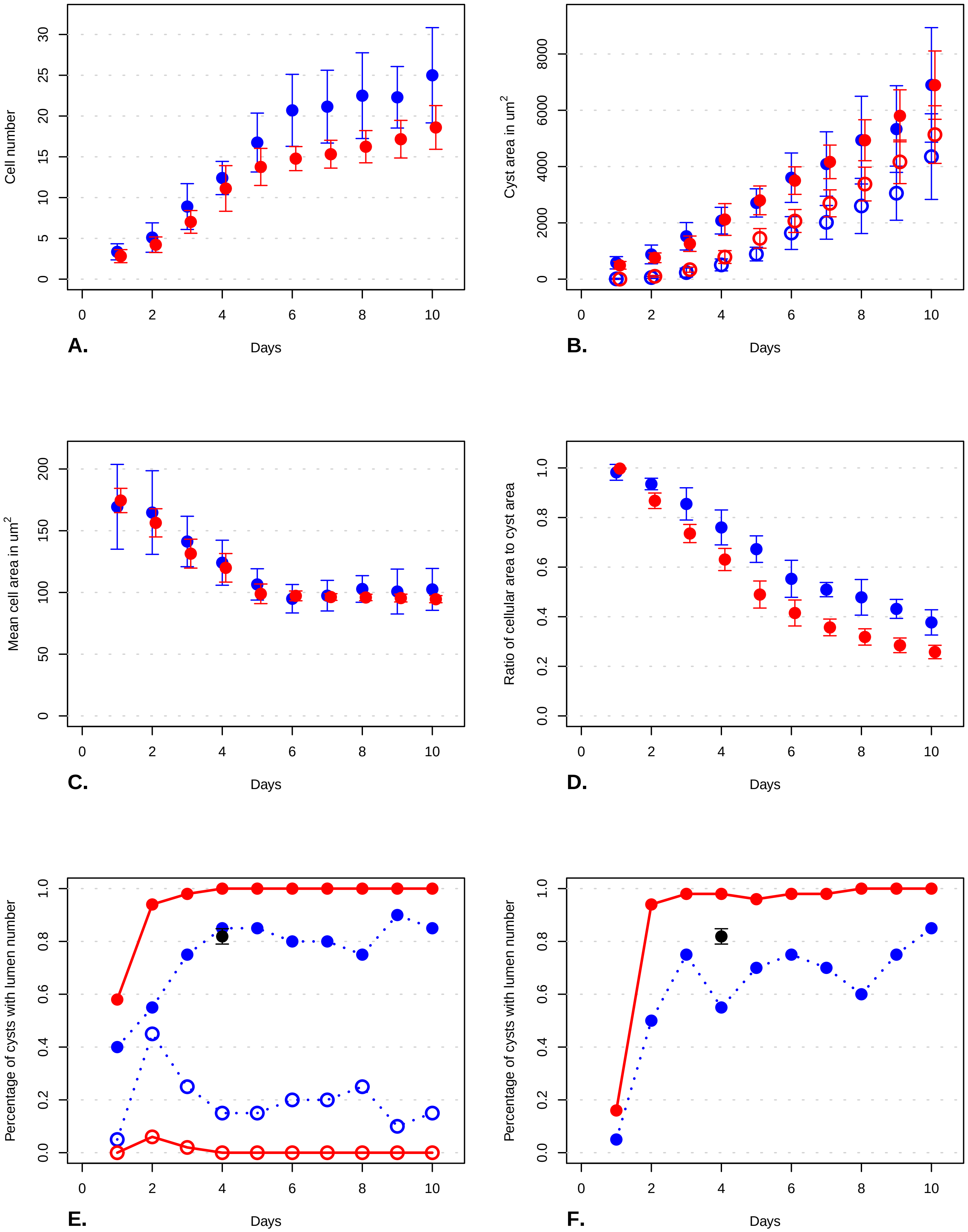

## Figure S11−60) Varied lumenGrowthRate: lgr = 0.006

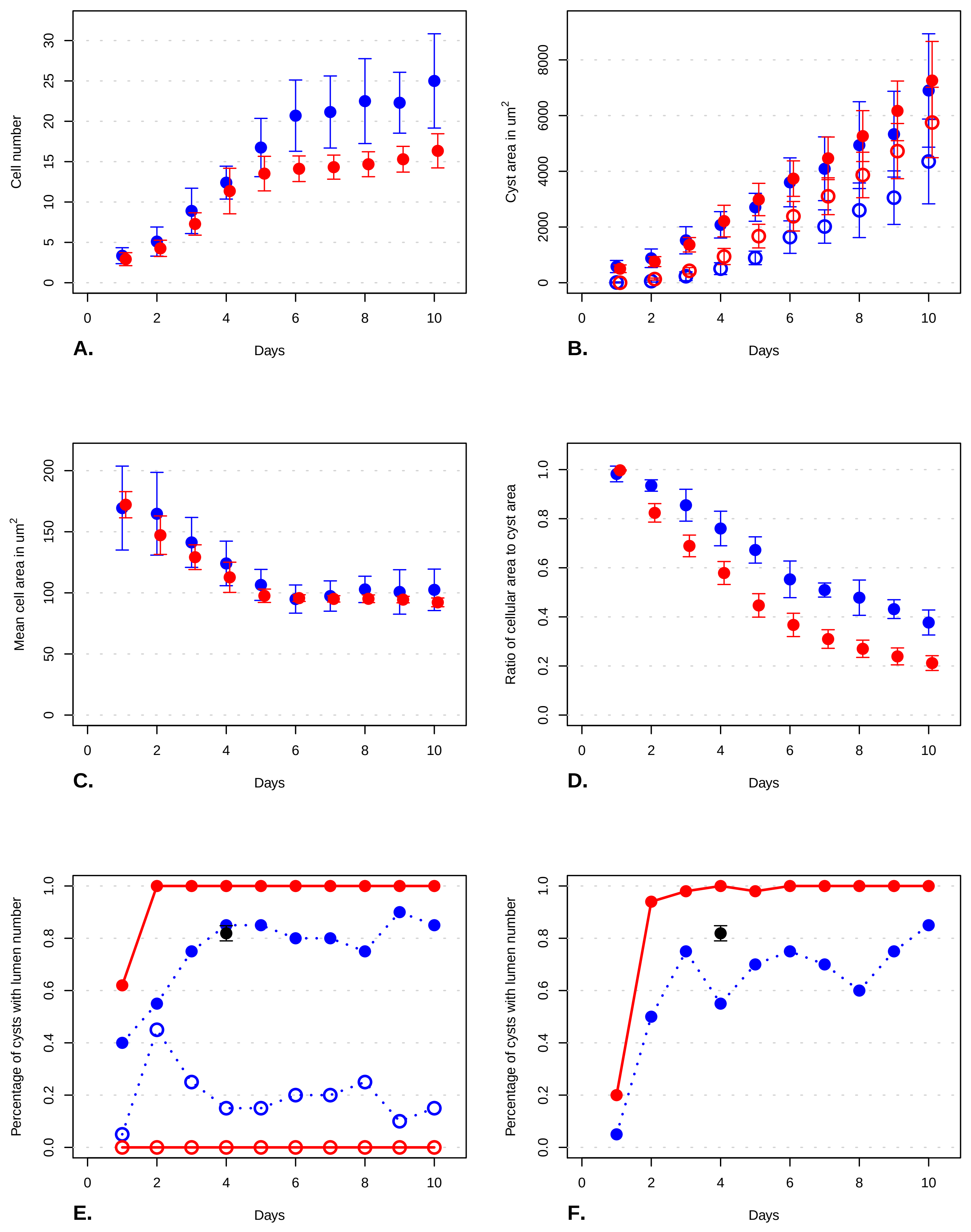

**Figure S11−61) Varied deathRateLumen: drl = 0.01**

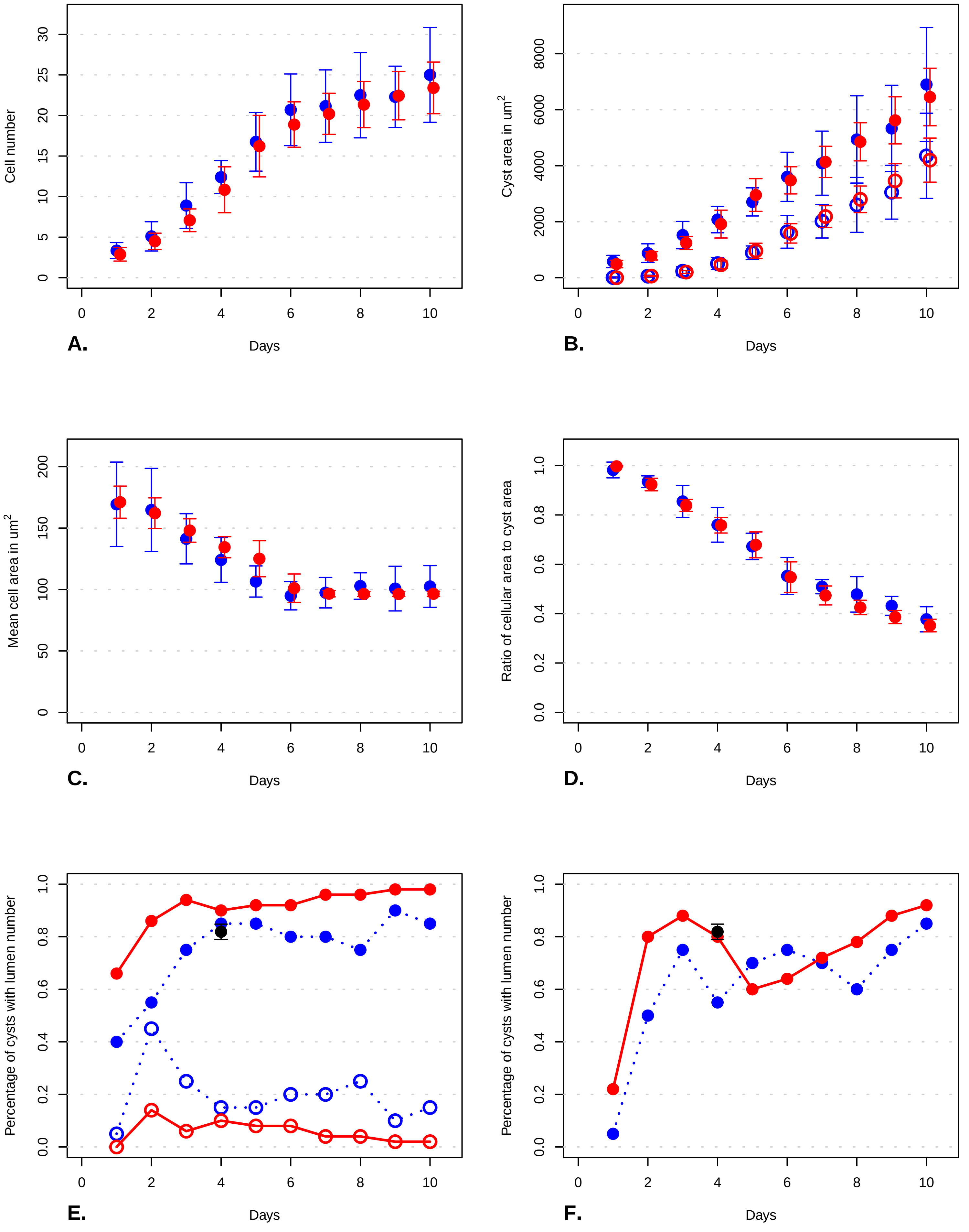

## Figure S11−62) Varied deathRateLumen: drl = 0.03

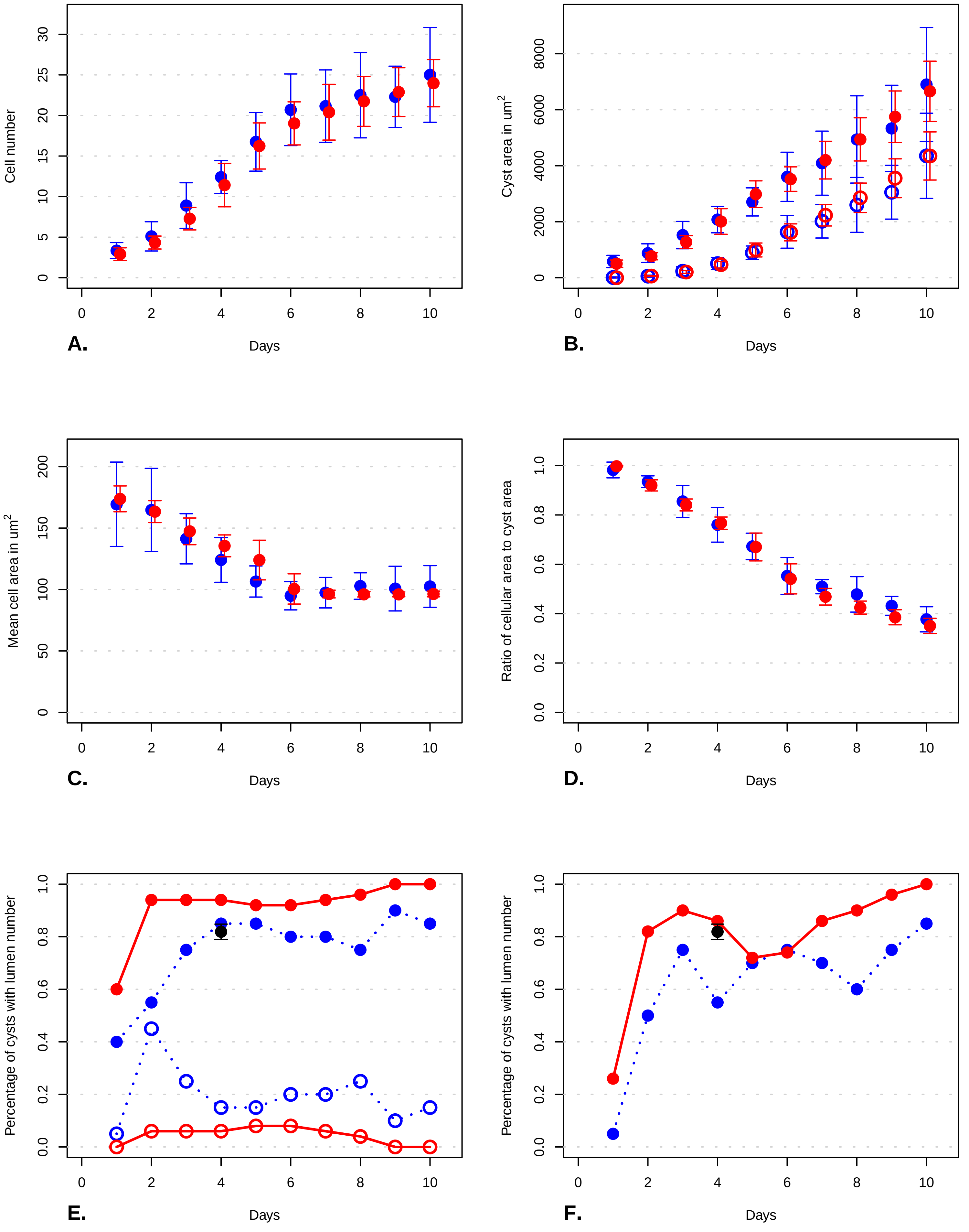

## Figure S11−63) Varied deathRateLumen: drl = 0.04

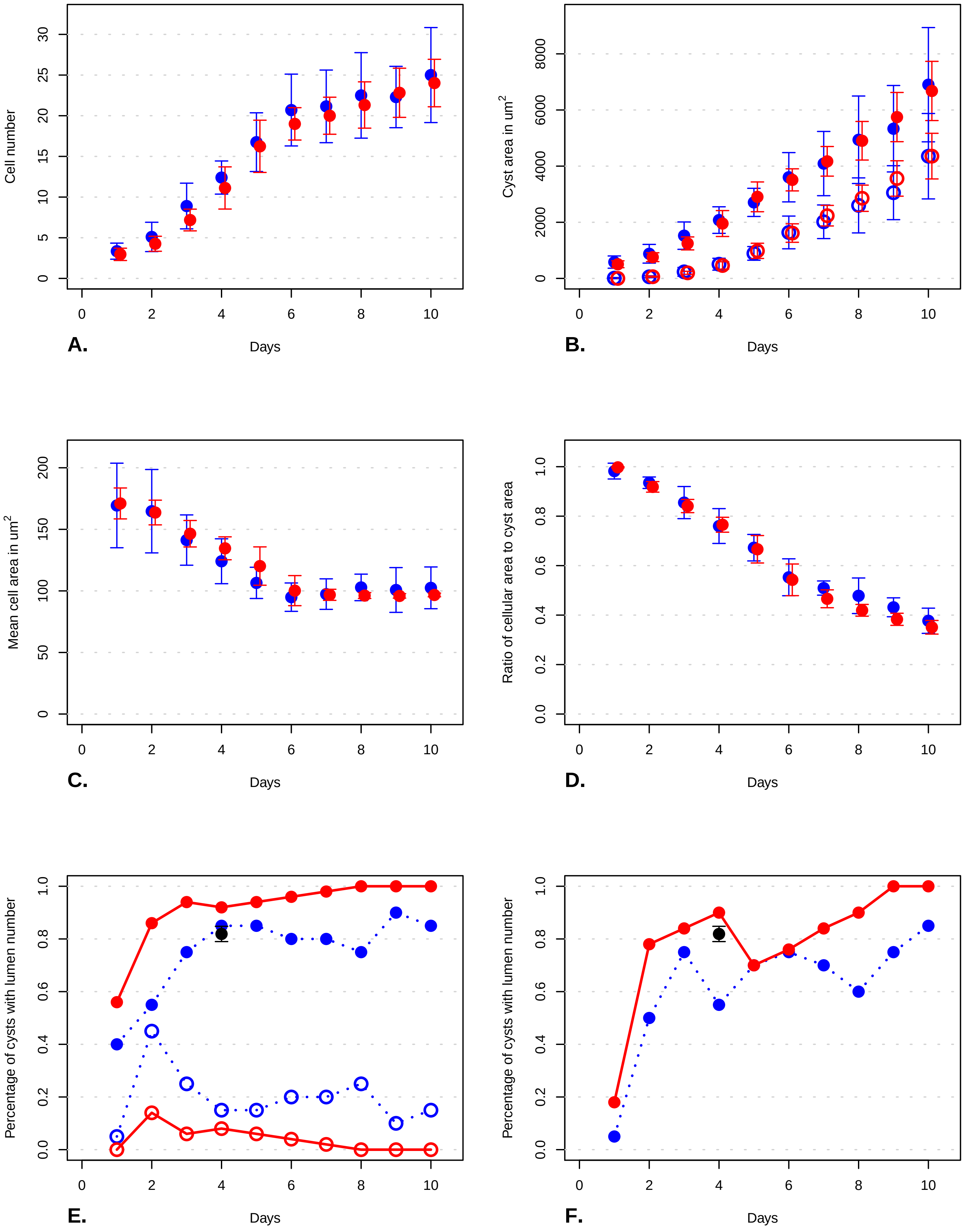

## Figure S11−64) Varied deathRateLumen: drl = 0.1

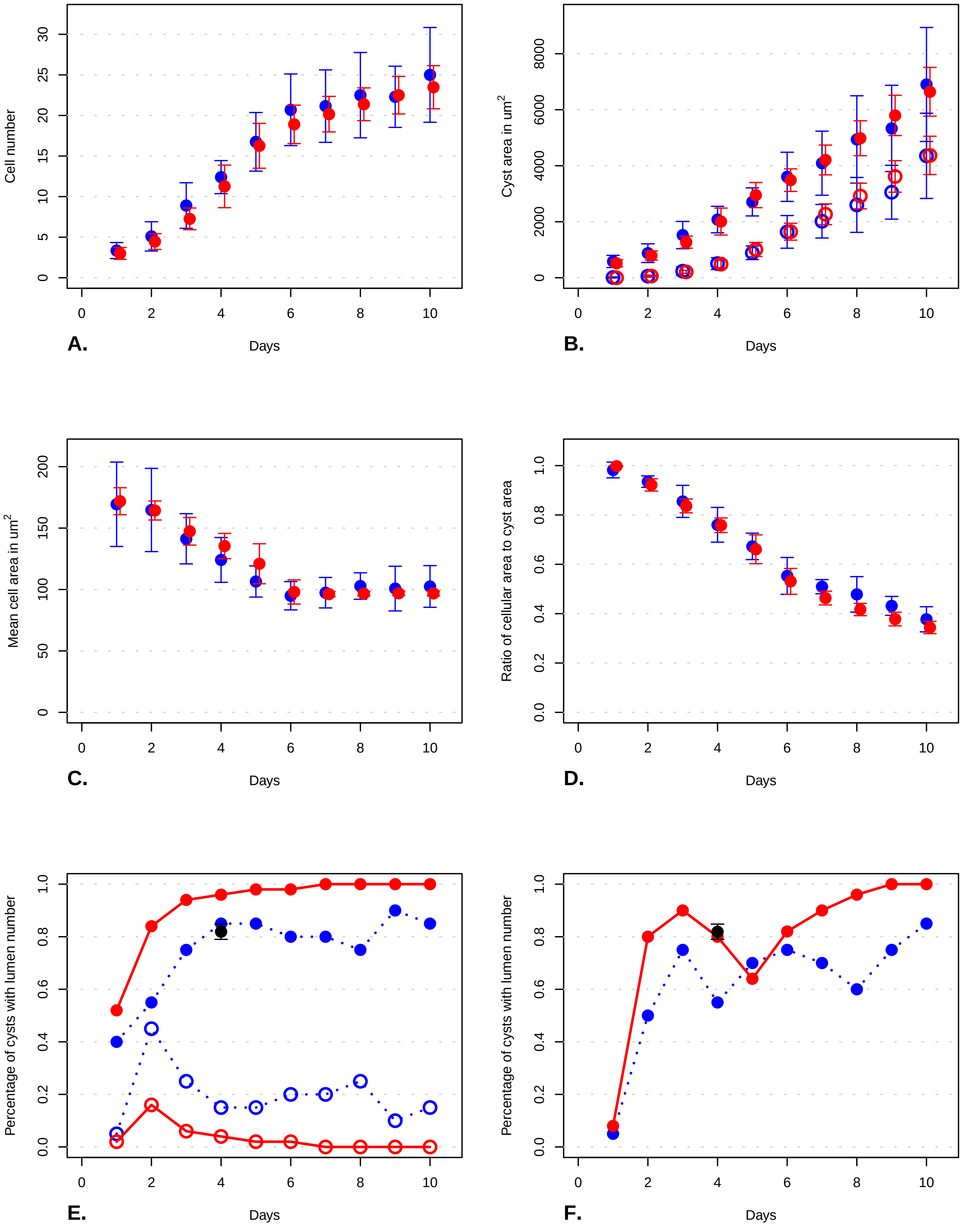

Figure S11−65) Varied deathRateEpi: dre = 0.0

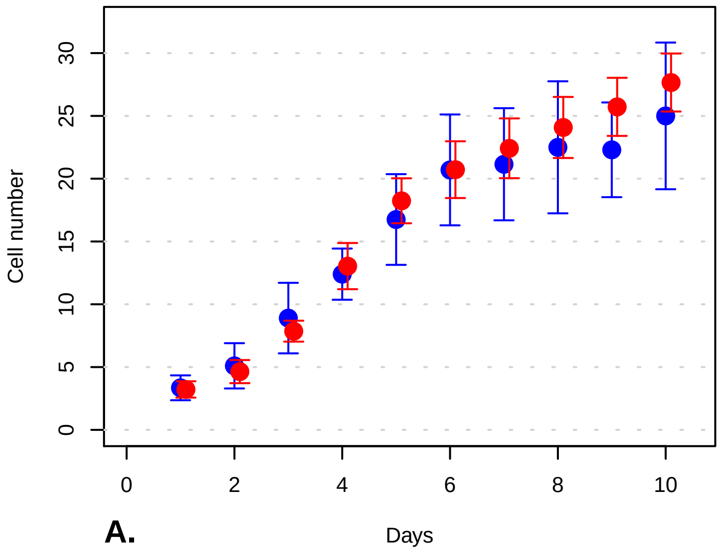


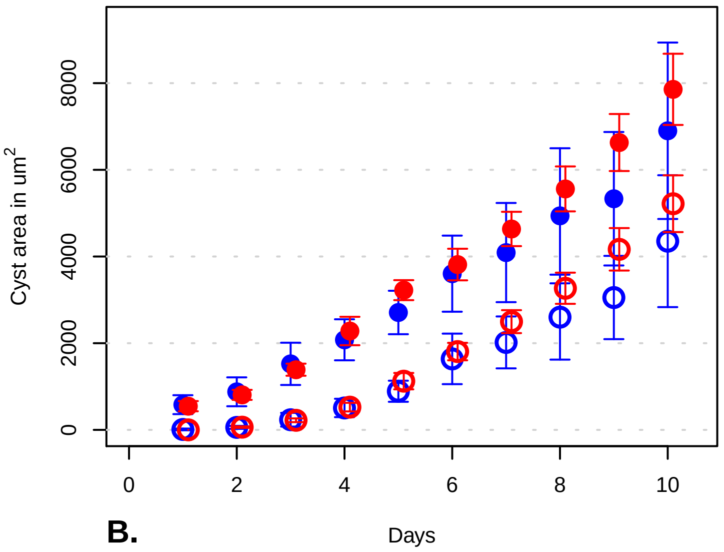


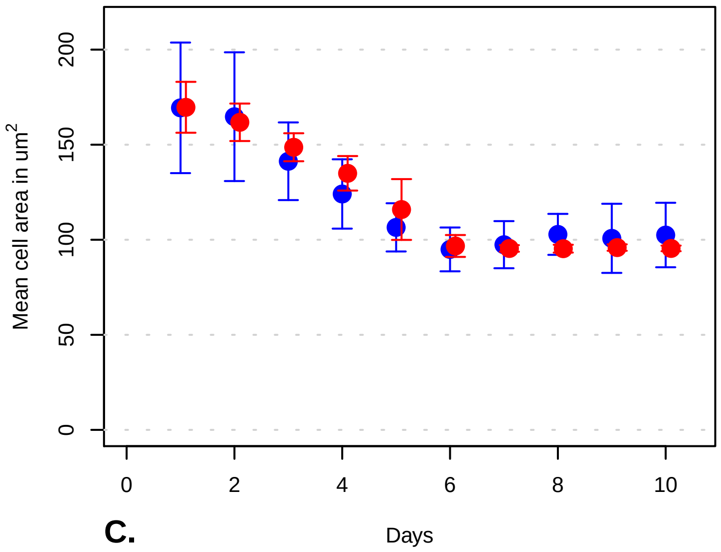


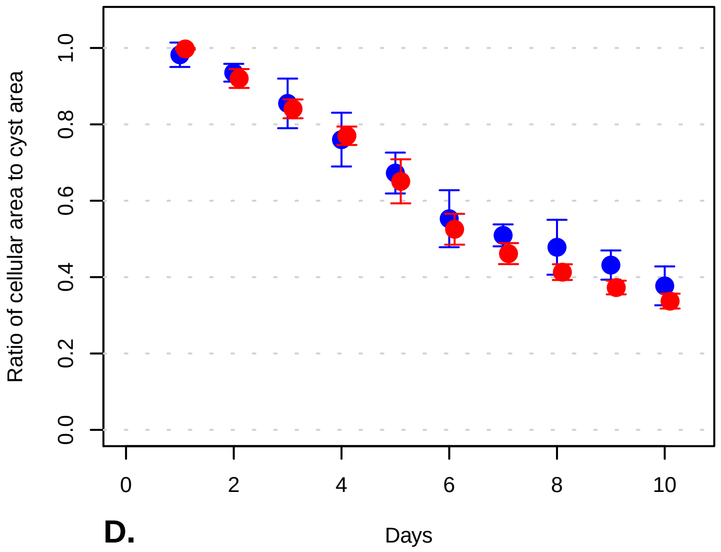


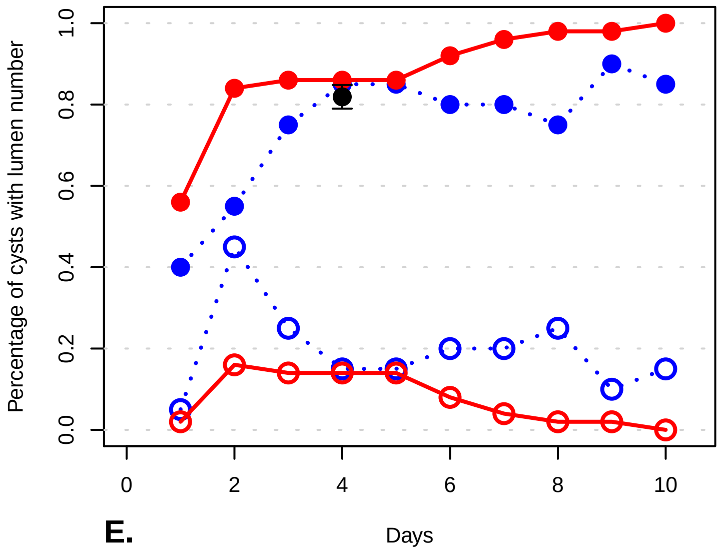


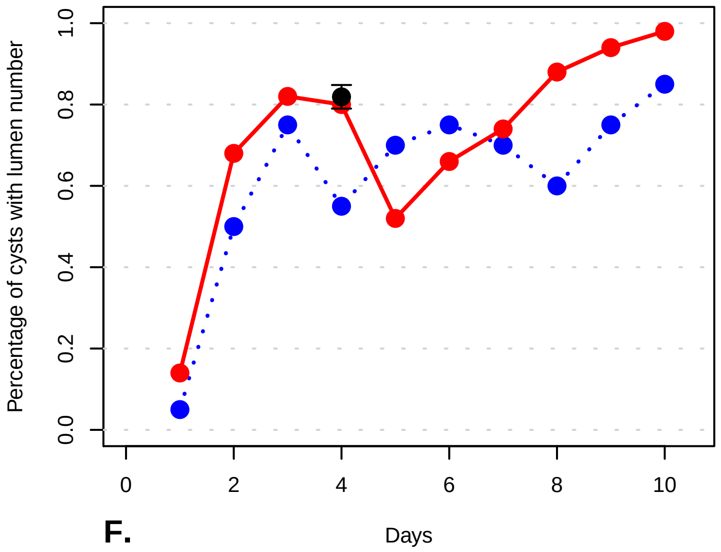

## Figure S11−66) Varied deathRateEpi: dre = 0.0002

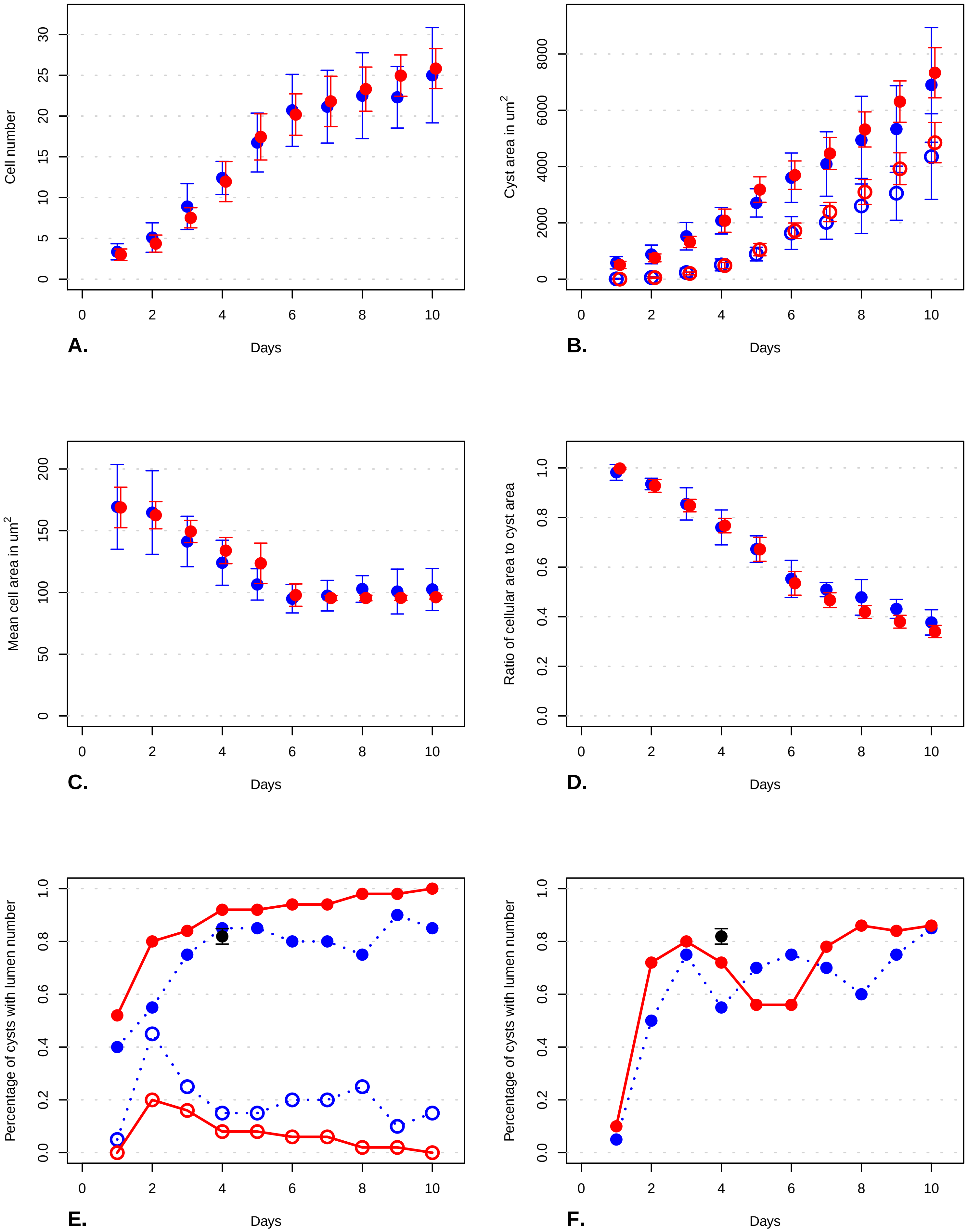

**Figure S11−67) Varied deathRateEpi: dre = 0.0008**

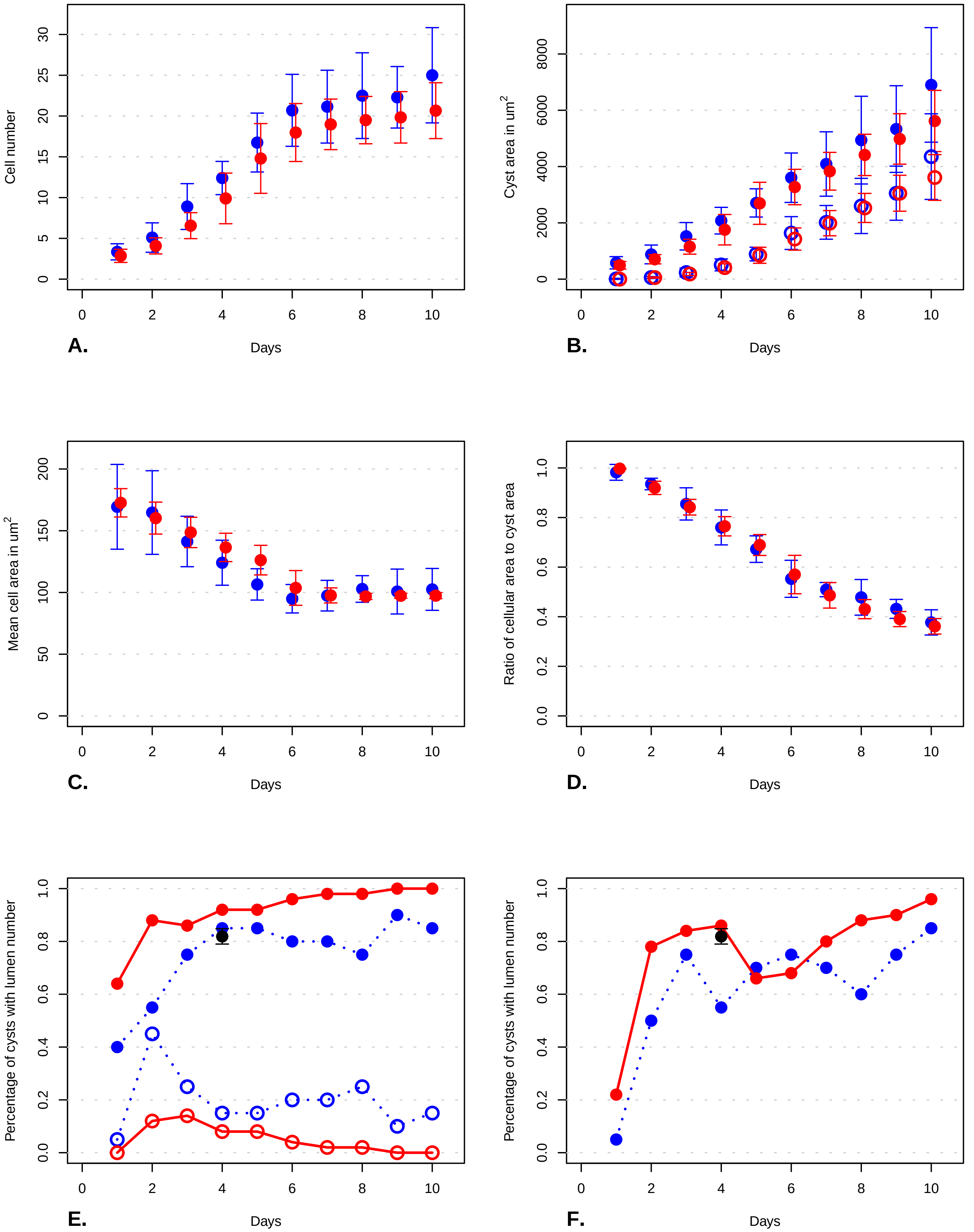

Figure S11−68) Varied deathRateEpi: dre = 0.0016

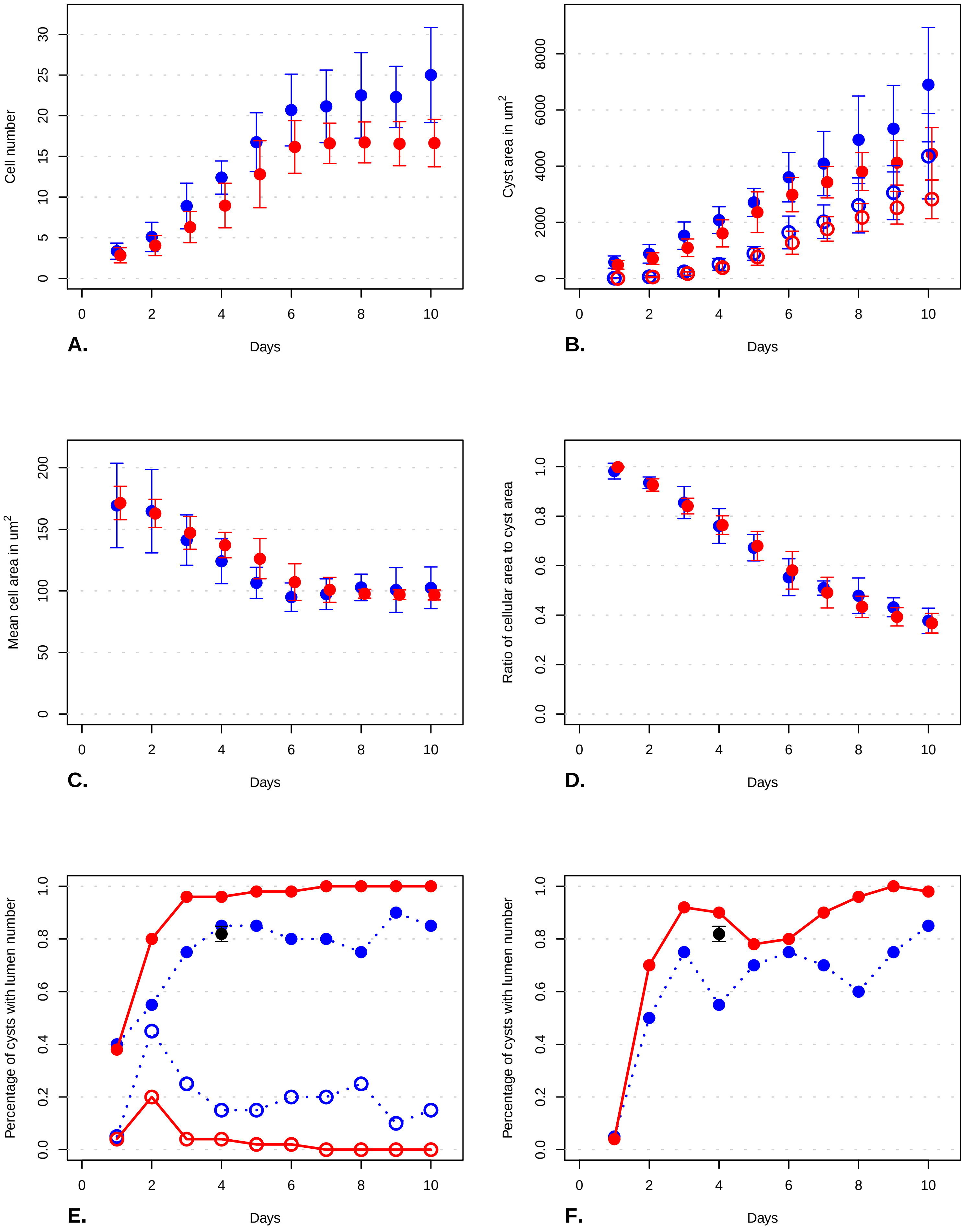

Figure S11−69) Varied dyingShrinkRate: dsr = 1.75

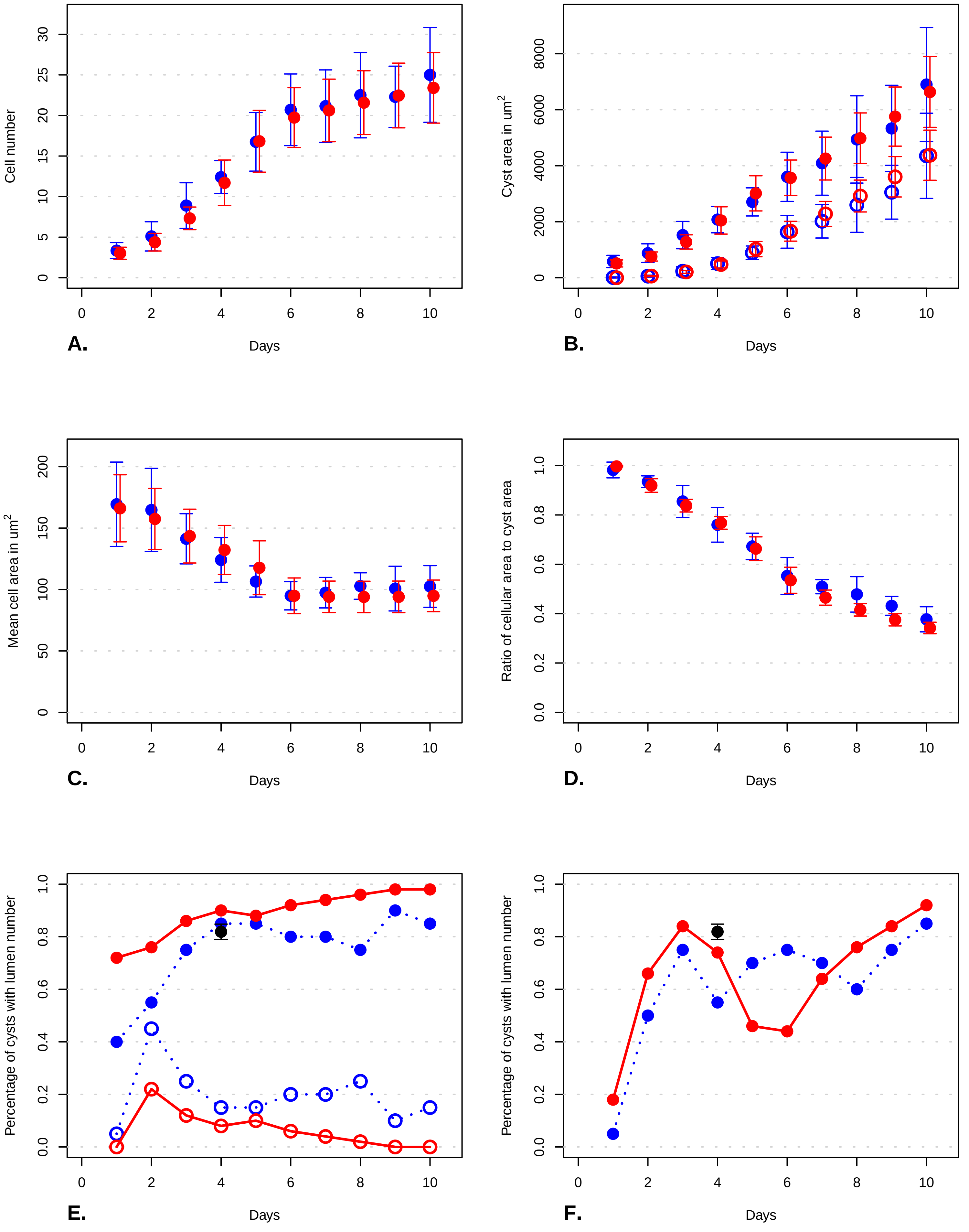

# Figure S11−70) Varied dyingShrinkRate: dsr = 4.5

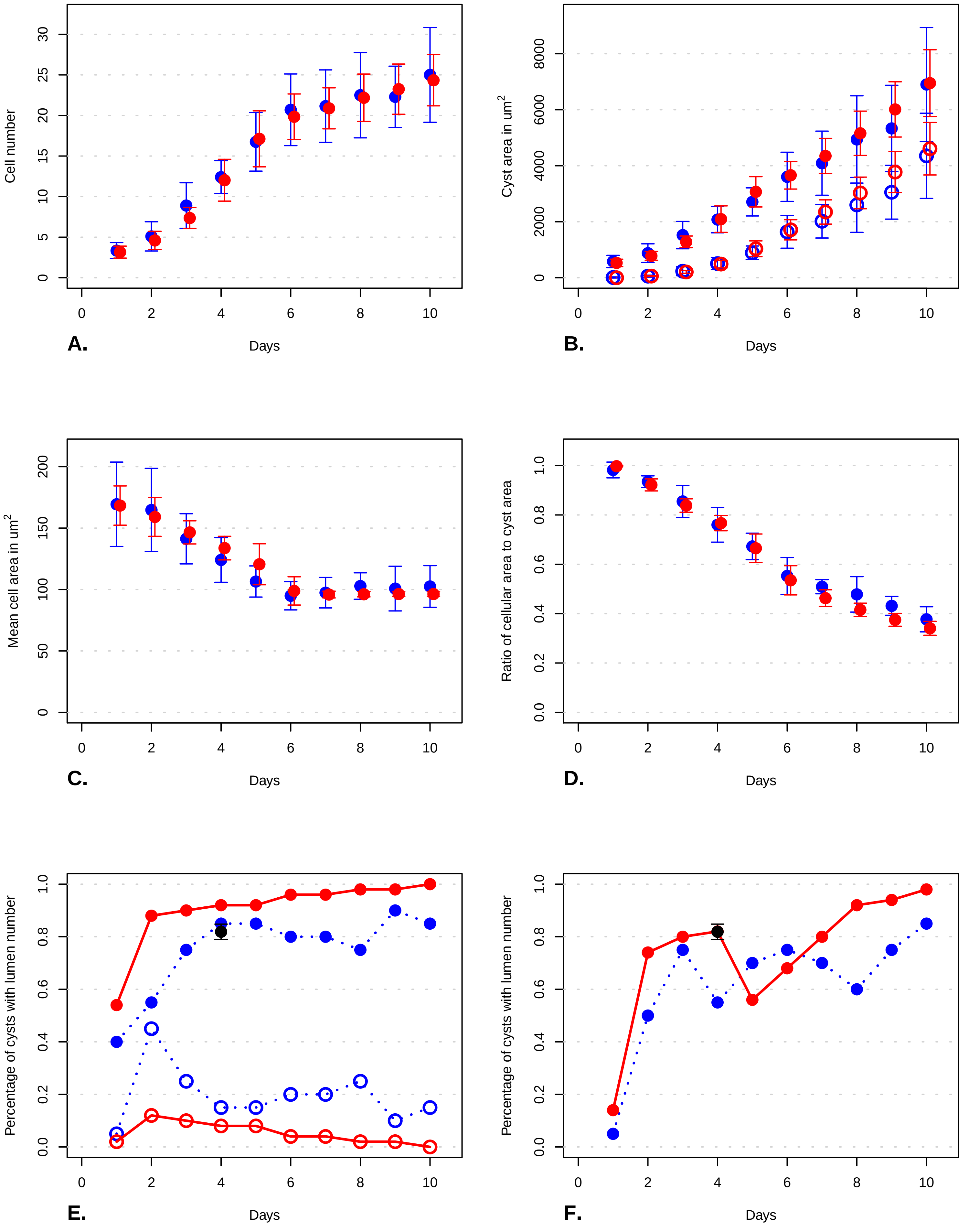

## Figure S11−71) Varied dyingShrinkRate: dsr = 13.5

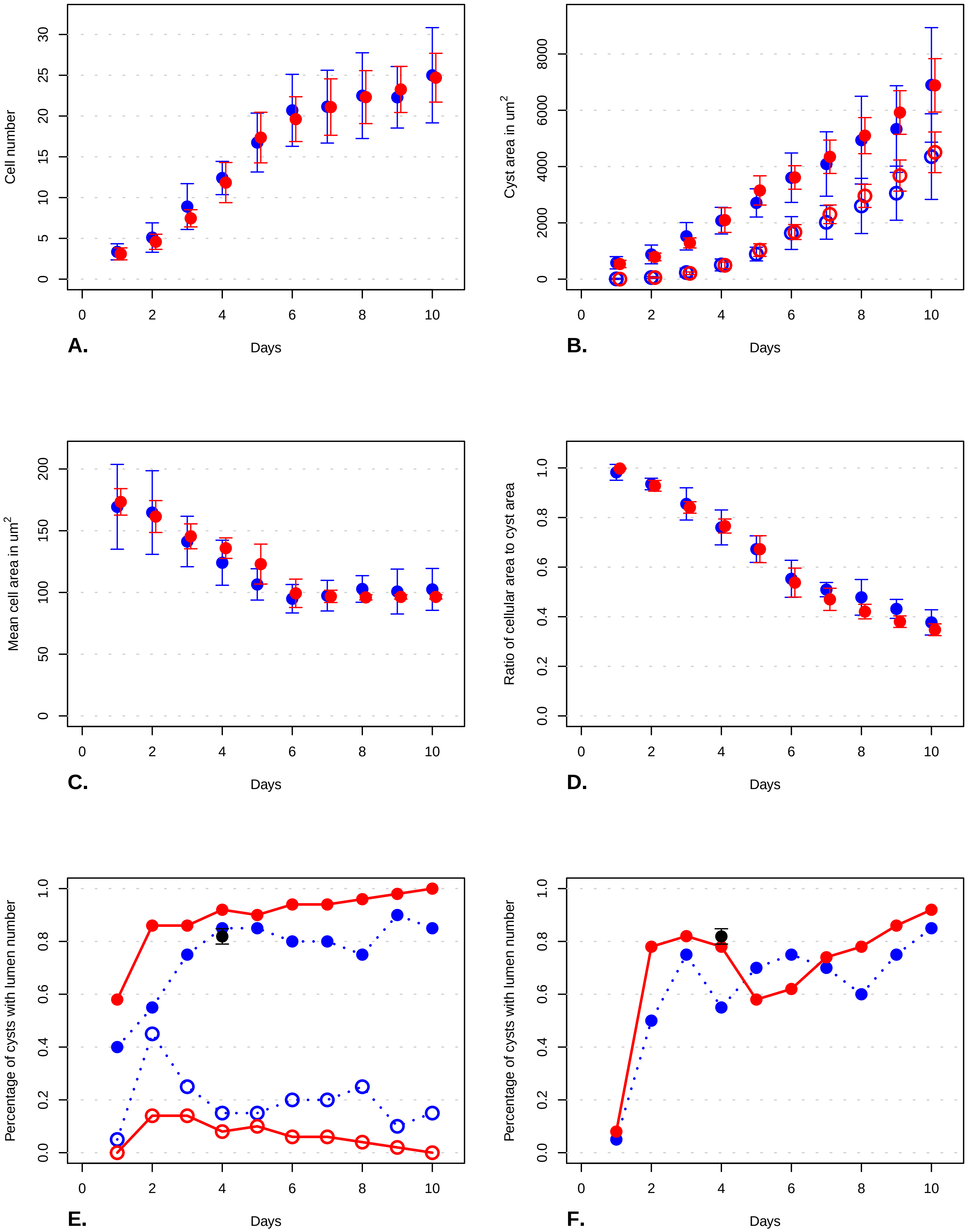

# Figure S11−72) Varied dyingShrinkRate: dsr = 18

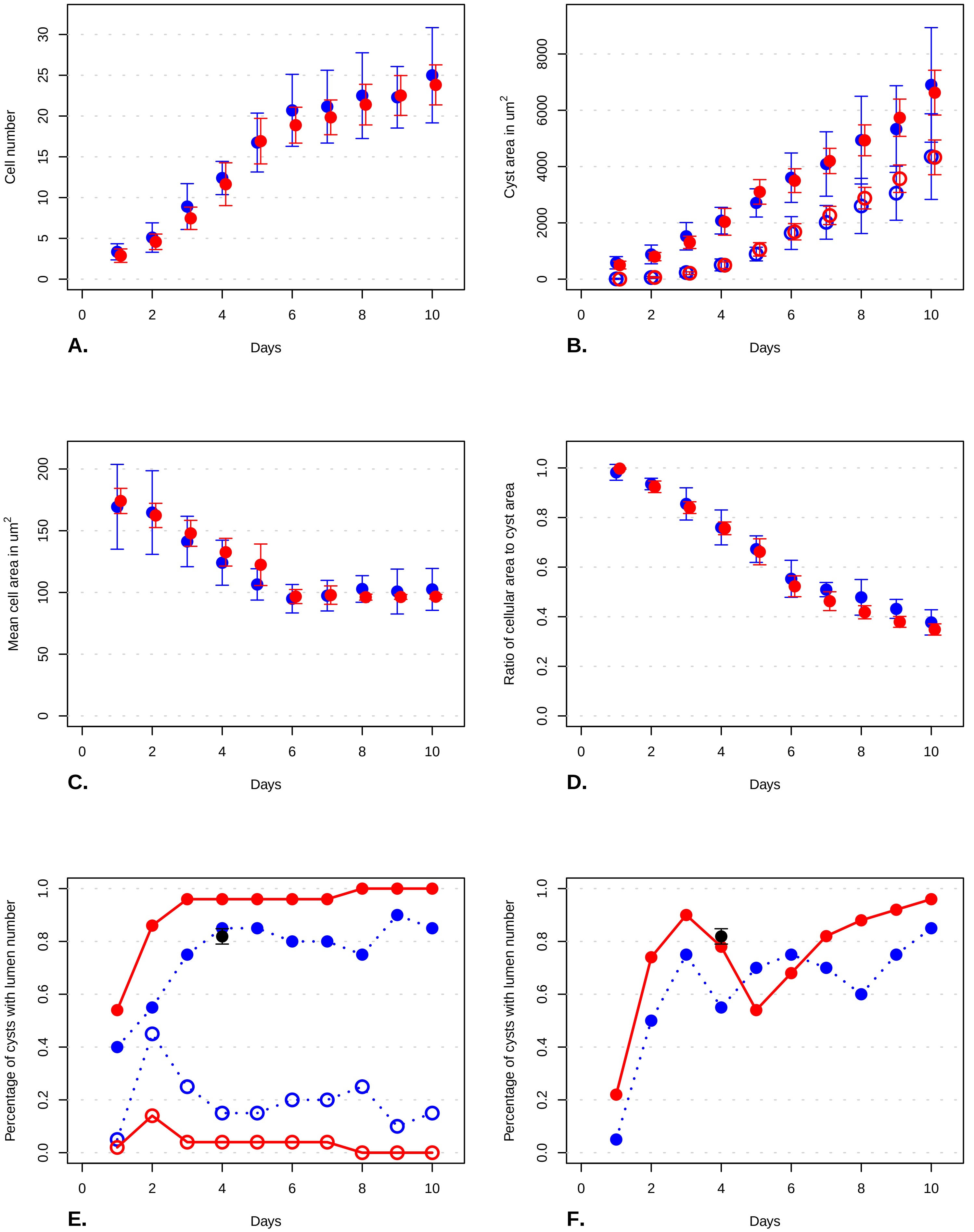

**Figure S11−73) Varied clusterProb: cp = 0**

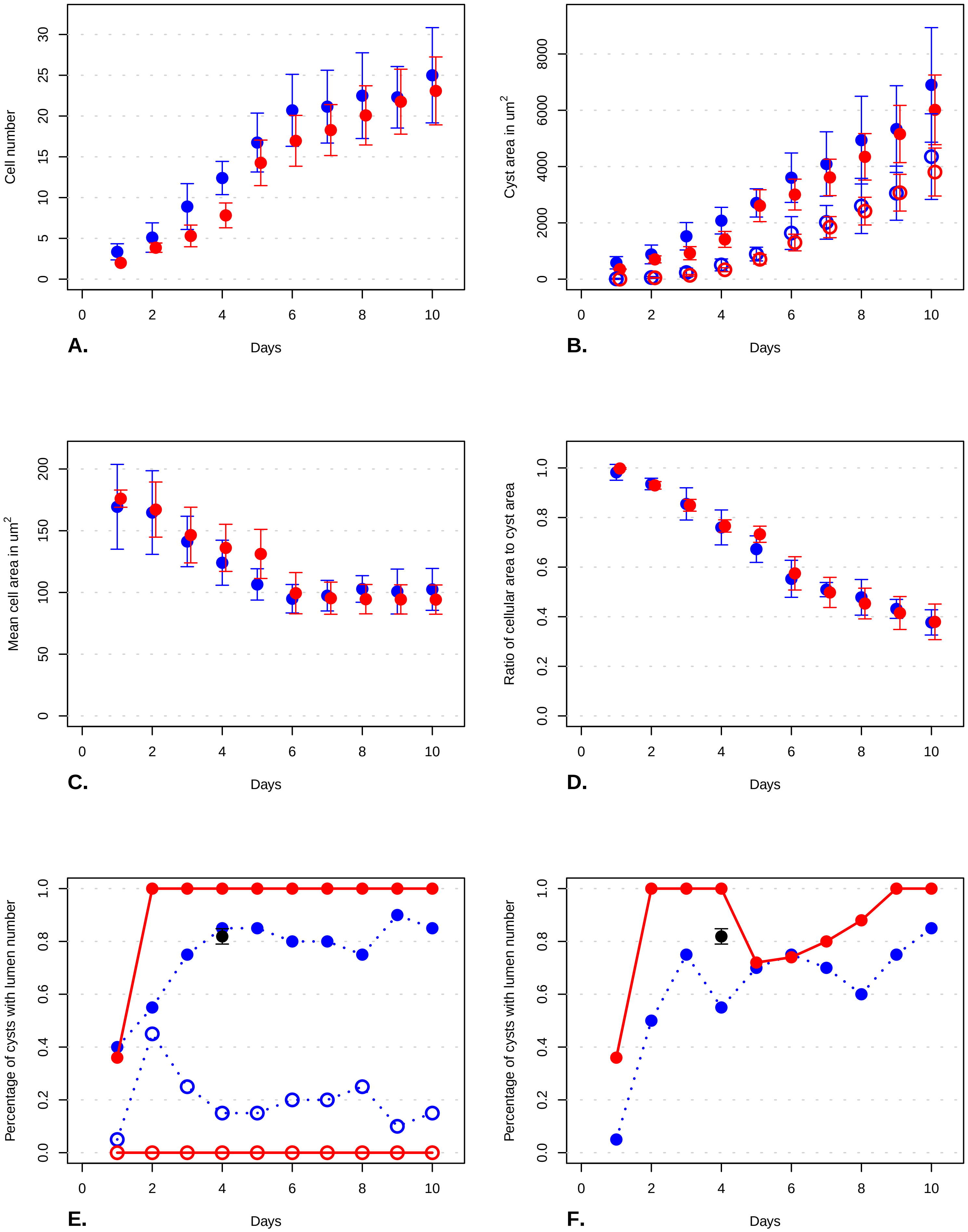

## Figure S11−74) Varied clusterProb: cp = 0.4

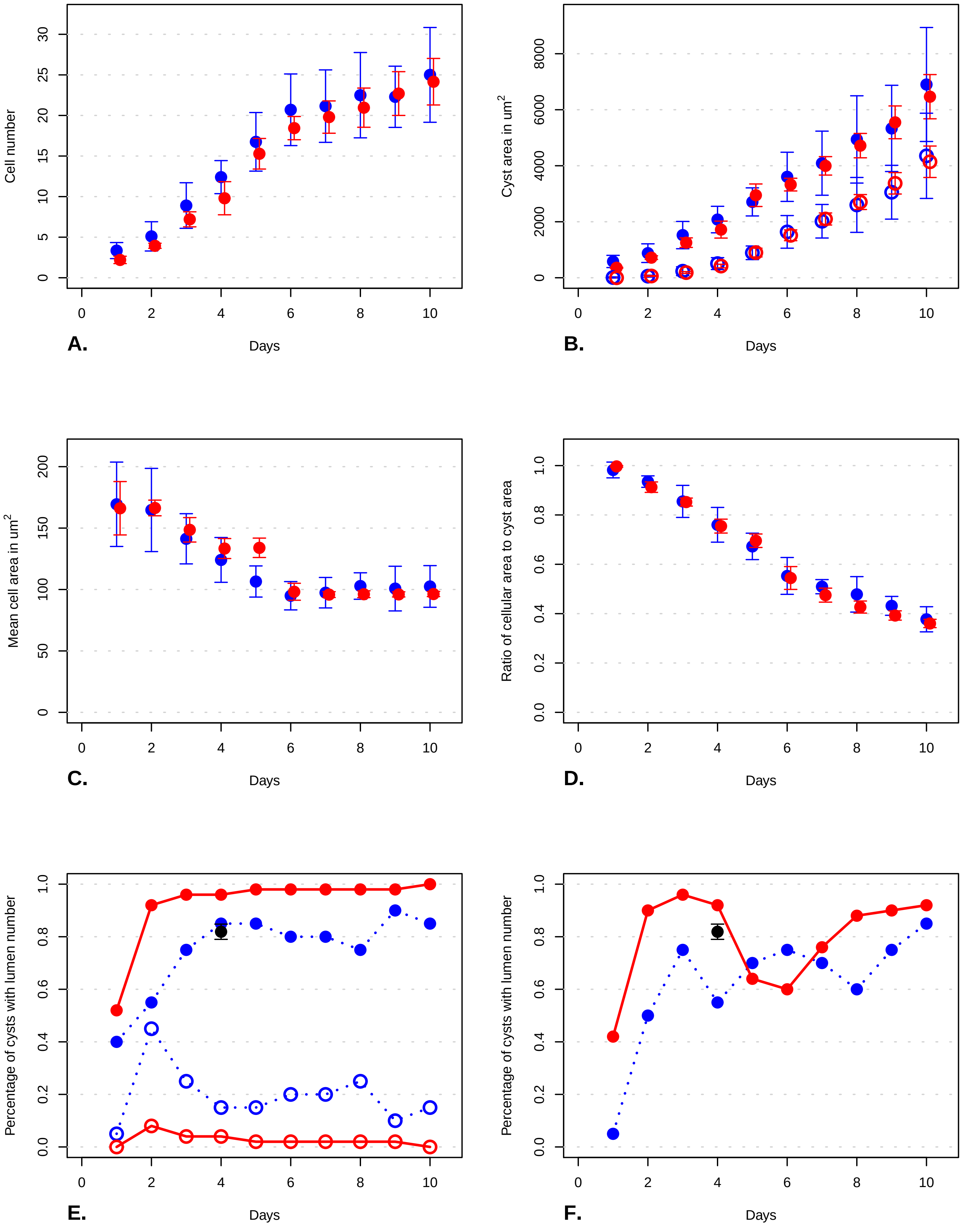

# Figure S11−75) Varied clusterProb: cp = 0.9

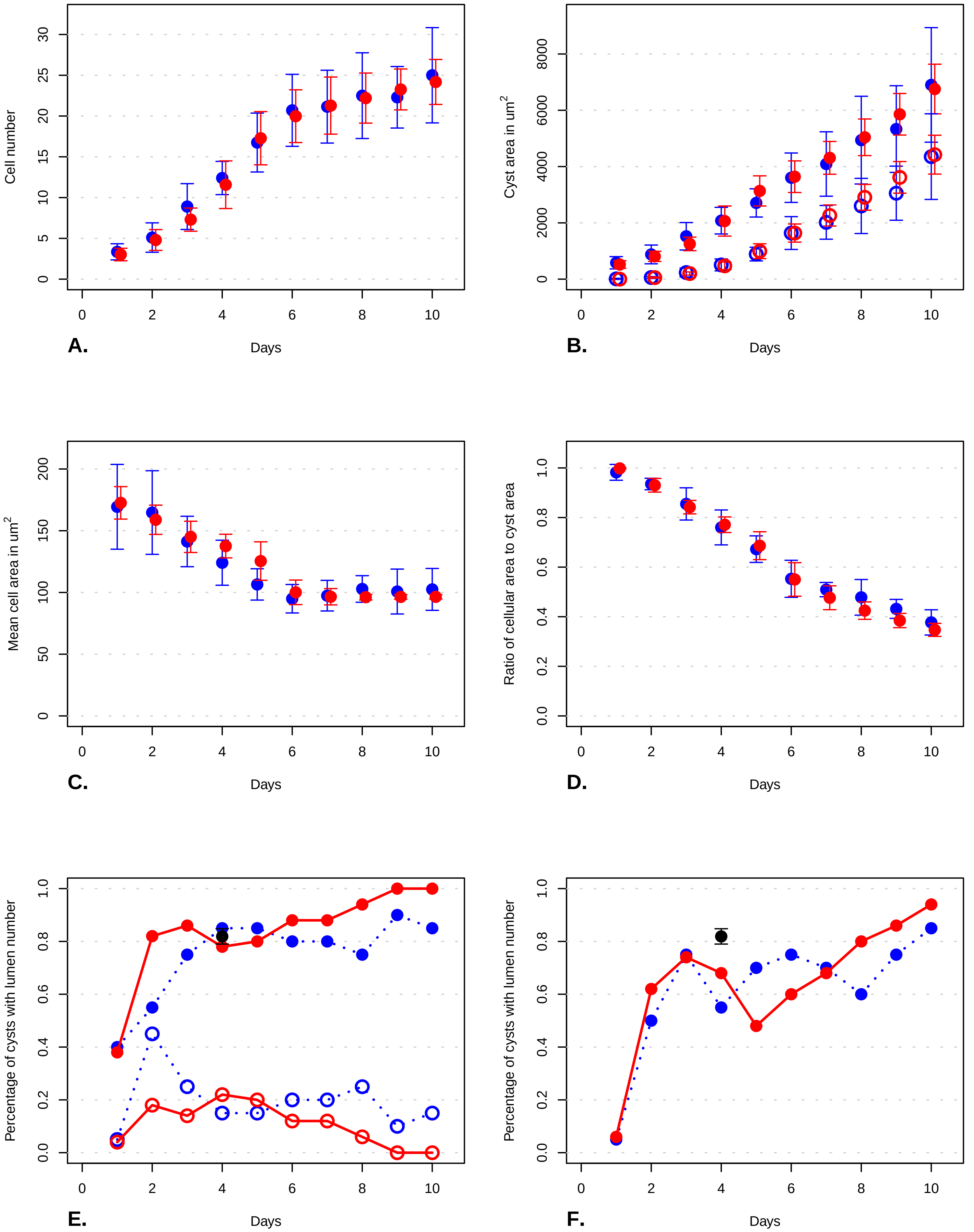

## Figure S11−76) Varied clusterProb: cp = 1

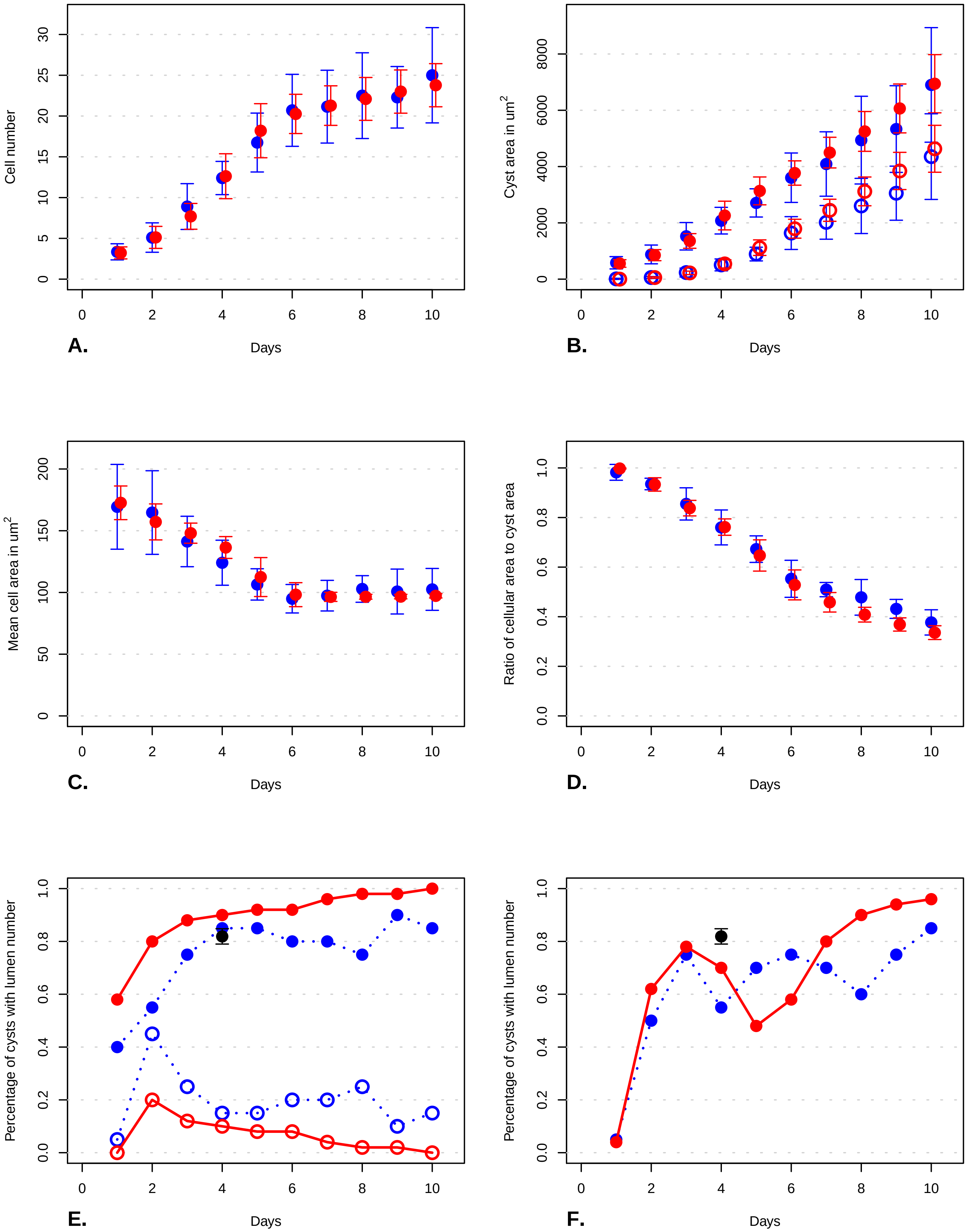

**Figure S11−77) Varied stableRatio: sr = 0.125**

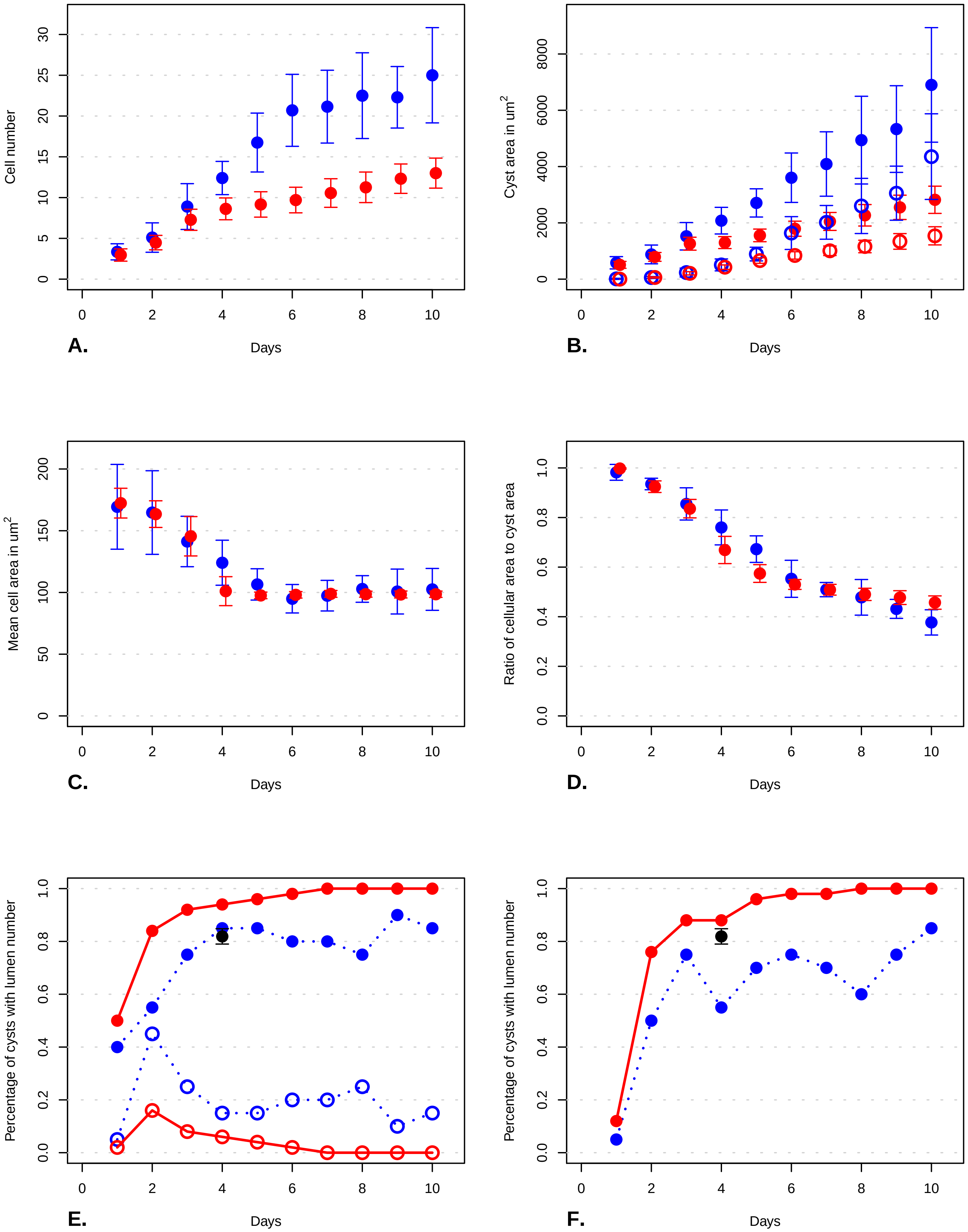

**Figure S11−78) Varied stableRatio: sr = 0.25**

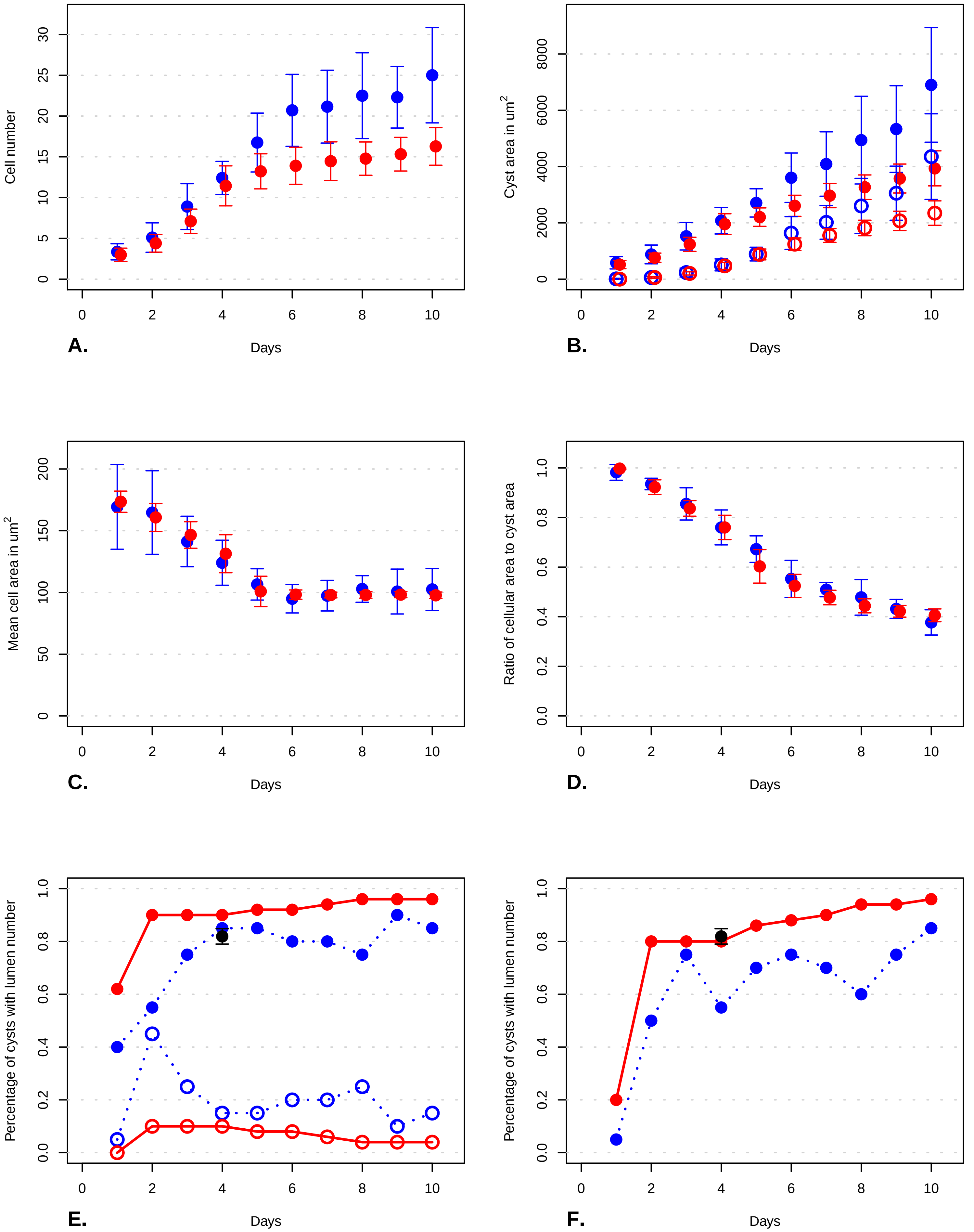

## Figure S11−79) Varied stableRatio: sr = 1.0

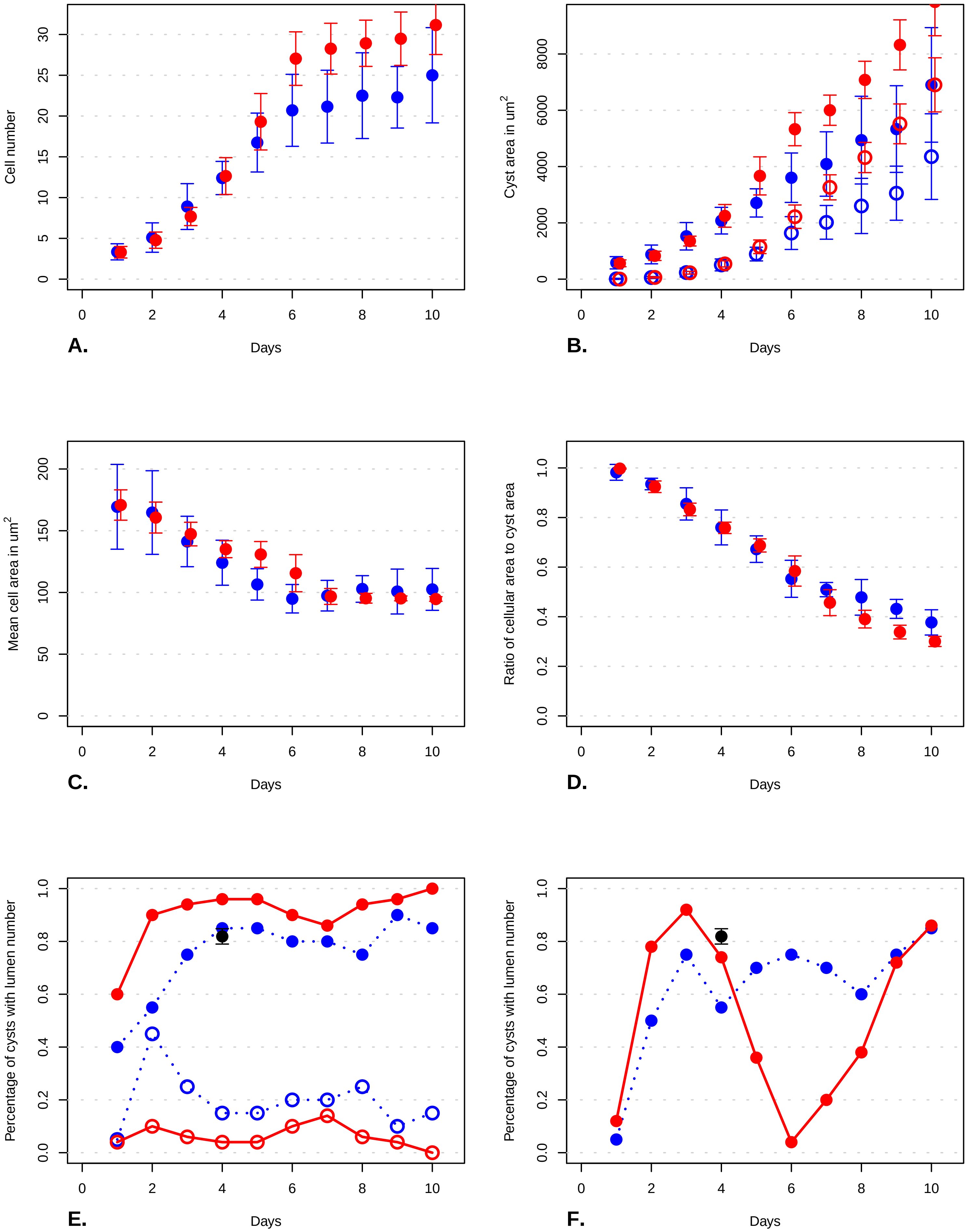

**Figure S11−80) Varied stableRatio: sr = 2.0**

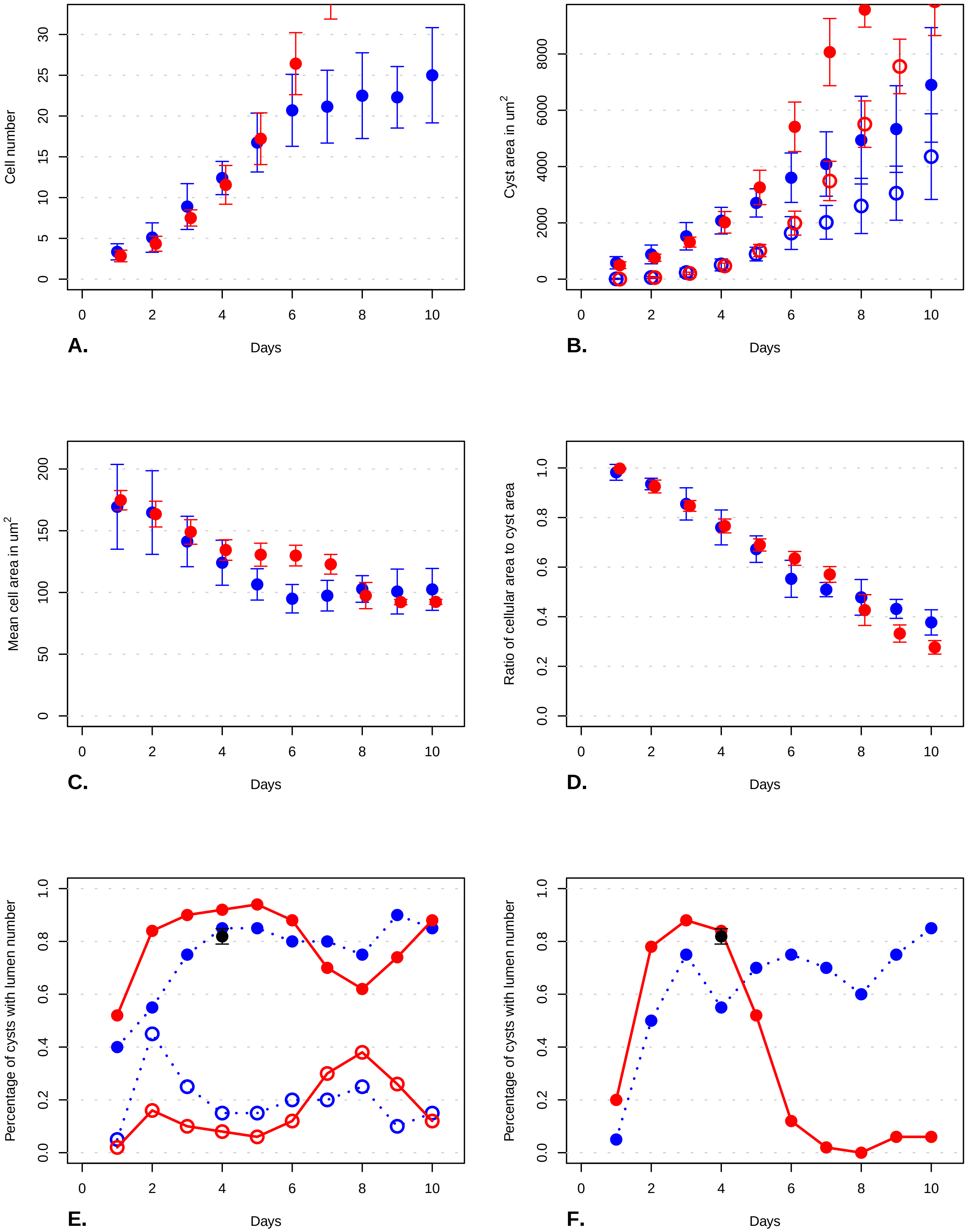

Supplement: Figure S11 — Parameter sweeping results. Experiments followed the same design as in Figures 2 and 3. Designations are the same as in Figures 2 and 3. Parameters changed from settings in Table 2 are listed within each Figure. S11-1 to S11-5: TS ISMA. S11-6 through S11-80 used the LS ISMA. Except for S11-1 to S11-5, all parameters were fixed except the single parameter being varied. S11-6 to S11-9: varied wedgeArea. S11-10 to S11-13: varied lambdaArea. S11-14 to S11-17: varied stableTargetArea. S11-18 to S11-23: varied cellCycle. S11-24 to S11-27: varied stableCycleDelay. S11-28 to S11-31: varied lambdaPerim. S11-32 to S11-35: varied polarDelay. S11-36 to S11-39: varied shiftDelay with high stableRatio. S11-40 to S11-44: varied lgrSubtract. S11-45 to S11-48: varied doublingArea. S11-49 to S11-52: varied multiplier. S11-53 to S11-60: varied lumenGrowthRate. S11-61 to S11-64: varied deathRateLumen. S11-65 to S11-68: varied deathRateEpi. S11-69 to S11-72: varied dyingShrinkRate. S11-73 to S11-76: varied clusterProb. S11-77 to S11-80: varied stableRatio. (PDF) [file pcbi.1002030.s011.pdf]
