# Supplementary material for: MDCK Cystogenesis Driven by Cell Stabilization within Computational Analogues
Source: PLoS Comput Biol. 2011 Apr 7;7(4):e1002030. doi: 10.1371/journal.pcbi.1002030 (PMC3072361; doi:10.1371/journal.pcbi.1002030)
Supplement: Table S1 — SM2 values for the ISMA. An SM2 value is the absolute value of the coefficient of variance (for a specific measure) subtracted from the in vitro coefficient of variance. Values over 0.25 (black) did not achieve the validation target described in the text. Values between 0.15 and 0.25 (gray) achieved the moderate validation target. Values less than 0.15 (white) achieved the strong validation target. (DOC) [file pcbi.1002030.s014.doc]

**Table S1. SM2 values for the ISMA.**

| **Day** | **Cell number** | **Cyst area** | **Lumen area** | **Mean cell area** | **Ratio: cellular to cyst area** |
| --- | --- | --- | --- | --- | --- |
| 1 | 0.06 | 0.15 | 0.77 | 0.10 | 0.03 |
| 2 | 0.13 | 0.20 | 0.25 | 0.14 | 0.00 |
| 3 | 0.14 | 0.16 | 0.44 | 0.09 | 0.04 |
| 4 | 0.05 | 0.01 | 0.16 | 0.09 | 0.05 |
| 5 | 0.04 | 0.03 | 0.04 | 0.01 | 0.00 |
| 6 | 0.09 | 0.14 | 0.19 | 0.03 | 0.04 |
| 7 | 0.11 | 0.17 | 0.16 | 0.11 | 0.00 |
| 8 | 0.13 | 0.21 | 0.24 | 0.08 | 0.09 |
| 9 | 0.06 | 0.18 | 0.18 | 0.16 | 0.03 |
| 10 | 0.13 | 0.18 | 0.21 | 0.14 | 0.07 |

An SM2 value is the absolute value of the CV (for a specific measure) subtracted from the in vitro CV. Values over 0.25 (black) did not achieve the validation target described in the text. Values between 0.15 and 0.25 (gray) achieved the moderate validation target. Values less than 0.15 (white) achieved the strong validation target.
