# Supplementary material for: MDCK Cystogenesis Driven by Cell Stabilization within Computational Analogues
Source: PLoS Comput Biol. 2011 Apr 7;7(4):e1002030. doi: 10.1371/journal.pcbi.1002030 (PMC3072361; doi:10.1371/journal.pcbi.1002030)
Supplement: Table S2 — Sources of stochasticity within ISMAs. The listed events or variable assignments provide behavior variability during cystogenesis. Note that index changes, which are random, contribute to many cell level events, including shape change and lumen expansion. (DOC) [file pcbi.1002030.s015.doc]

**Table S2. Sources of stochasticity within ISMAs.**

| **Description** | **Event or variable assignment** | **Range / Conditional** | **Parameters used** |
| --- | --- | --- | --- |
| Cells set *cycleCounter* if it is null | Variable | *r*[0.75 x *cellCycle*, 1.25 x *cellCycle*] | *cellCycle* |
| At simulation cycle 5, cells lower *cycleCounter* | Variable | *r*[1 – *clusterProb* x *cellCycle*, *cellCycle*] | *cellCycle, clusterProb* |
| Sets initial *polarCounter* | Variable | *r*[0.75 x *polarDelay* 1.25 x *polarDelay*] | *polarDelay* |
| Child cell sets *polarCounter* | Variable | 1. pVal = *polarDelay* – parent.pCounter  2. *r*[0.5 x pVal, 1.5 x pVal] | *polarDelay, parent.pCounter* |
| Cell selects random angle of division | Variable | *r*(0,1) x π / 2 |  |
| Stable cell decreases *cycleCounter* | Event | If *r*[0,1] < (1 – *stableCycleDelay*) | *shiftedCycleDelay* |
| Cell with matrix contact dies | Event | If *r*[0,1] < *deathRateLumen* | *deathRateLumen* |
| Cell without matrix contact dies | Event | If *r*[0,1] < *deathRateEpi* | *deathRateEpi* |
| Location changes index based on G | Event | If *r*[0,1] < AcceptanceProbability() | Many |

The listed events or variable assignments provide behavior variability during cystogenesis. Note that index changes, which are random, contribute to many cell-level events, including shape change and lumen expansion.
