# Supplementary material for: MDCK Cystogenesis Driven by Cell Stabilization within Computational Analogues
Source: PLoS Comput Biol. 2011 Apr 7;7(4):e1002030. doi: 10.1371/journal.pcbi.1002030 (PMC3072361; doi:10.1371/journal.pcbi.1002030)
Supplement: Table S3 — Additional ISMA parameters. Parameters used for the ISMA system and for the adhesion plug-in are listed along with descriptions, default values used for simulation, and parameter ranges that are expected to give normal results. (DOC) [file pcbi.1002030.s016.doc]

**Table S3. Additional ISMA parameters.**

| **System parameters** | **Description** | **Current value** | **Practical range** |
| --- | --- | --- | --- |
| *simNumber* | Simulation number |  | 0-N |
| *folderName* | Location of data stored for this experiment |  | 0-N |
| *dimX* | Size of simulation window in X direction | 100 | 50-300 |
| *dimY* | Size of simulation window in Y direction | 100 | 50-300 |
| *anneal* | Number of post-ending simulation cycles | 50 | 10-100 |
| *steps* | Number of simulation cycles in ISMA run | 481 | 200-1000 |
| *temperature* | Likelihood an index change will be accepted | 30 | 5-100 |
| *flip2DimRatio* | Number of index changes attempted is equal  to *dimx* x *dimy* x *flip2dimratio* | 3 | 1-10 |
| *neighborOrder* | Radius of neighbor grid used to calculate energy for index change attempts | 1 | 1-3 |
| *spindleRandom* | Random value added to axis of division when cells divide | 0 | 0-100 |
|  |  |  |  |
| **Adhesion parameters** |  |  |  |
| *polarcell_polarcell* | Energy generated by this type of interaction. Higher energy is less favorable to contact. | 5 | 0-10^5^ |
| *polarcell_cell* | As above | 20 | 0-10^5^ |
| *polarcell_lumen* | As above | 120 | 0-10^5^ |
| *polarcell_matrix* | As above | 40 | 0-10^5^ |
| *polarcell_stablecell* | As above | 5 | 0-10^5^ |
| *cell_cell* | As above | 20 | 0-10^5^ |
| *cell_lumen* | As above | 5x10^5^ | 0-10^5^ |
| *cell_matrix* | As above | 15 | 0-10^5^ |
| *cell_stablecell* | As above | 20 | 0-10^5^ |
| *lumen_lumen* | As above | 150 | 0-10^5^ |
| *lumen_matrix* | As above | 5x10^5^ | 0-10^5^ |
| *lumen_stablecell* | As above | 120 | 0-10^5^ |
| *matrix_matrix* | As above | 150 | 0-10^5^ |
| *matrix_stablecell* | As above | 40 | 0-10^5^ |
| *stablecell_stablecell* | As above | 5 | 0-10^5^ |

Parameters used for the ISMA system and for the adhesion plug-in are listed along with descriptions, default value used for simulation, and parameter ranges that are expected to give normal results.
