# Supplementary material for: MDCK Cystogenesis Driven by Cell Stabilization within Computational Analogues
Source: PLoS Comput Biol. 2011 Apr 7;7(4):e1002030. doi: 10.1371/journal.pcbi.1002030 (PMC3072361; doi:10.1371/journal.pcbi.1002030)
Supplement: Table S4 — Mean cell number per day for cysts grown in Matrigel. Numbers in bold italic are measured, mean values and non-bold numbers are projected values. Projected values were found by multiplying or dividing the measured mean values by the scaling factor of 1.56. During the first four days of growth, the number of cells increased by a constant factor of 1.4 to 1.8 per day, with a value of 1.56 minimizing the percent error between projected and measured mean values. Using that scaling factor, the number of cells at day 0 was estimated to be 2.1, indicating that some clustering took place within the Matrigel culture. To reflect this observation, ISMAs implemented cell clustering. (DOC) [file pcbi.1002030.s017.doc]

**Table S4. Mean cell number per day for cysts grown in Matrigel.**

|  | Projected from day 1 | Projected from day 2 | Projected from day 3 | Projected from day 4 |
| --- | --- | --- | --- | --- |
| Day 0 | 2.15 | 2.09 | 2.44 | 2.14 |
| Day 1 | ***3.35*** | 3.27 | 3.80 | 3.35 |
| Day 2 | 5.23 | ***5.1*** | 5.93 | 5.22 |
| Day 3 | 8.15 | 7.956 | ***9.25*** | 8.14 |
| Day 4 | 12.72 | 12.41 | 14.43 | ***12.7*** |

Numbers in bold italic are measured, mean values and non-bold numbers are projected values. Projected values were found by multiplying or dividing the measured mean values by the scaling factor of 1.56. During the first four days of growth, the number of cells increased by a constant factor of 1.4 to 1.8 per day, with a value of 1.56 minimizing the percent error between projected and measured mean values. Using that scaling factor, the number of cells at day 0 was estimated to be 2.1, indicating that some clustering took place within the Matrigel culture. To reflect this observation, ISMAs began with two to four cells.
