# Supplementary material for: MDCK Cystogenesis Driven by Cell Stabilization within Computational Analogues
Source: PLoS Comput Biol. 2011 Apr 7;7(4):e1002030. doi: 10.1371/journal.pcbi.1002030 (PMC3072361; doi:10.1371/journal.pcbi.1002030)
Supplement: Text S1 — Supporting methods. (PDF) [file pcbi.1002030.s018.pdf]

Text S1. Supporting Information

TIGHT JUNCTION reorganization

TIGHT JUNCTIONS (TJs) were implemented for three reasons: 1) To prevent CELLS from coming into contact with multiple LUMENS. 2) To allow two neighboring LUMENS to merge without violating (1). 3) To allow LUMENS to expand without violating (1). TJs are an accounting mechanism stored as pairs of points. A TJ is defined as pairs of points in adjacent CELLS in contact with the same LUMEN. TJs are counted around a point in two ways (Figure S12A). 1) If the location is within a LUMEN then the surrounding points are surveyed and any two adjacent points in different CELLS are considered to be TJs and stored. 2) If the location is within a CELL then the surrounding points are surveyed and if any two adjacent points are in a different CELL and a LUMEN, then the point within the other CELL and the current point are considered a TJ and stored.

During index change, any index change that would change the number of TJs surrounding that location will be rejected (Figure S12B). Also, any index change from a CELL to a LUMEN where there are two TJs before and after the index change will be rejected. The latter rule is included because in rare cases when a DYING CELL is shrinking it is possible for a neighboring CELL that contacts a LUMEN to come into contact with a second LUMEN without the number of TJs changing. During the execution of individual CELL logic, LUMEN merging and expansion through TJ reorganization may occur. All points within TJs are surveyed and the first possible merge or accepted expansion will be executed. Only a single merge or expansion can be executed during a simulation cycle because once one does there are new TJs and the TJ list is incorrect. Rather than update the list and redo it, the process ends and is begun again during the next simulation cycle.

LUMEN merging will always happen if it is allowed. For this to occur a point within a TJ must be neighboring another point within a TJ that is in contact with a different LUMEN (FIGURE S12C). If that occurs two of the points, each contacting a different LUMEN, will each change their index to that of one of the LUMENS, and then the two LUMENS, now in contact with each other, will merge to form a single LUMEN. Essentially, when four CELLS contacting two different LUMENS are in close proximity to each other then the two LUMENS will merge.

LUMEN expansion through TJ reorganization only occurs if it is energetically favorable as in the index change step detailed in the text, except that the TJ changing penalties are not assessed. For a point within a TJ to change its index into LUMEN, it first checks that no neighboring points are TJs contacting other LUMENS, that no neighboring points are contacting other LUMENS, and that no neighboring points are MATRIX or UNPOLARIZED CELLS (Figure S12D). Then the point will check to see if any of the neighboring points satisfy the conditions that will allow this point to change into a LUMEN. At least one neighboring point must be in a POLARIZED or stable CELL that is not already in a TJ and is not the same CELL as the current point. If this is the case the location will calculate the energy change generated by converting from CELL to LUMEN and find the resulting probability p by running the result through an Acceptance Function. If a pseudorandom number $r[0,1]$ is less than p the change will be accepted and the simulation updated accordingly (Figure S12E). This code allows LUMENS to expand as they would normally without the possibility of CELLS contacting multiple LUMENS. It also allows LUMENS separated by a single-location-wide area of CELLS to merge together into a single LUMEN.

Timed shift ISMA

The TS ISMA used an internal clock to determine when a CELL would stabilize. The internal clock was based on the variable *shiftCounter*, initialized to equal *shiftDelay* after a CELL

POLARIZED. *ShiftCounter* was decremented at each simulation cycle, and when it reached zero the CELL would change to the stabilized state.

Geometrical mechanism ISMA

The GM ISMA was an earlier version that contained a number of differences in its implementation. The foremost was the method used to determine when CELLS stabilized. In the GM ISMA the following differences existed:

- CELLS stabilized when their wedge area was more than twice their actual area instead of relying on LUMEN size.
- CELL clustering was calculated in a different fashion, in which two random number calculations were performed instead of one.
- LUMEN target area was calculated based on *lumenGrowthRate*, LUMEN perimeter, CELL stretch, and the number of stable CELLS bordering the LUMEN.
- CELLS had an increased likelihood of DYING if they did not contact the MATRIX but did contact stabilized CELLS.

Computational objects

The ISMA consists of a number of computational objects, the most significant of which are listed below:

- *Point*: a grid location
- CELL: contains variables like targetArea
- *MCell*: contains a pointer to a CELL, a list of points that the CELL occupies, and internal variables
- *CellInventory*: contains a list of CELLS
- *CellField*: contains a list of all points and maps points to CELLS
- *CellMap*: maps a CELL to each *MCell*

Technical specification

CC3D functions in either 2D or 3D and lets users choose between a square and hexagonal grid. It provides architecture for calculating changes in energy and accepting or rejecting changes in grid locations. The architecture is designed from a system-based perspective. Each simulation cycle, each aspect of the system is executed, from the index change step that selects random points, to the plug-ins that update aspects of the system. The CPM is straightforward and mathematically simple, making it easy to understand and its execution fast. The ISMAs were constructed using CompuCell3D 3.2.1 and custom code, as described in Methods. Simulation code and user manual are available at <http://biosystems.ucsf.edu/research_mdckcyst.html>. All simulations were executed on a Dell Poweredge 1900 server with 2 4-core 2.33GHz 64 bit Intel Xeon processors. The system had 8 GB of RAM and a 450 GB hard drive. The system software was Ubuntu 8.04 LTS (Linux kernel 2.6). Data from simulations was captured in a MySQL 5.0 database using MySQL++ <http://tangentsoft.net/mysql++/> 3.2 to bridge between CompuCell3D <http://www.compucell3d.org/> and the database.

Challenging ISMA predictions

If the results of the experiments of Zheng et al. had falsified the ISMAs, we would have had to look into simulation details during execution to see where and how failure occurred. Because we

designed the analogues to make mechanism change straightforward, it would have been simple to discover a new set of mechanisms that achieved all the targeted attributes described herein, as well as the new attributes from the challenge experiments. The results of these experiments demonstrated two important uses for this class of in silico models. 1) They can be used to execute in silico experiments designed to test hypotheses based on the expected consequences of a mechanistic outcome. Our experience has been that aspects of expected outcomes are typically wrong in one or more ways. 2) The results of simulations can be used to stand as hypotheses about outcomes of similarly designed in vitro experiments. The first use exercises our understanding of the networked relations between ISMA mechanisms and phenotype, including systemic behaviors, and that in turn facilitates thinking more deeply about MDCK cell biology. The phenotypes of descendents of current ISMAs will cover increasingly complex behaviors of MDCK cyst cultures. At that stage, it may become standard practice to conduct many exploratory intervention experiments in silico in order to better focus the design of in vitro experiments, marking a fundamental transition from reverse engineering to forward engineering of complex biological systems.[1]

Potential molecular counterparts to TS and LS ISMA mechanisms

The sirtuin protein SIRT1, a protein deacetylase, has been shown to be involved in cellular senescence, the early phases of which are reversible [2]. This is associated with downregulation of SIRT1 and an increased function of cyclic AMP-regulated kinase (AMPK) [3]. SIRT1 downregulation increases the stability of the AMPK regulator LKB1 and thus inhibits cell proliferation [4]. A possible cell autonomous timing mechanism that could cause cell stabilization might involve downregulation of SIRT1 and upregualtion of LKB1.

Evidence suggests is possible that the tension generated at the luminal membrane is transduced by the subapical F-actin network. It maintains luminal integrity and allows recycling endosomes to aggregate, regulating the protein and lipid composition of the apical plasma membrane. By regulating this F-actin network, cells can control lumen and cyst size.

The Rho family small GTPhase Cdc42 is an ideal candidate for such a regulatory role [5]. Cdc42 is recruited to the apical plasma membrane by the Posphatidylinositol (4,5) bisphosphate (PI(4,5)P2) binding protein Annexin 2, and the loss of Cdc42 disrupts MDCK lumen formation ([6]. The GTPase exchange factor Tuba activates Cdc42 at the apical membrane, allowing it to control apical exocytosis and thus expand and maintain the apical plasma membrane [7]. Cdc42, in concert with PI(4,5)P2, also helps to polymerize actin by regulating N-Wasp and Arp2/3, thus maintaining the subapical F-actin network [8]. Cdc42 also prevents excessive apical constriction by antagonizing the GTPase RhoA through p190RhoGAP [9, 10]. Through the multiple regulatory roles of apical vesicle exocytosis, F-actin scaffold maintenance and regulation of apical constriction, Cdc42 is in a central position to control lumen size.

Cdc42 depletion early in MDCK cystogenesis leads to a loss of central lumen formation, further supporting its central role in this process [6]. Overexpressing a WT Cdc42 in drosophila pupal eye cells increases the apical membrane area [10], possibly reducing luminal tension. This lower tensions and higher surface area allows more fluid to be pumped into the luminal space by transmembrane pumps, increasing lumen size until the apical membrane tension is restored. This molecular mechanism presents one potential explanation for how MDCK cells could sense luminal tension and react accordingly.

Experiments to validate molecular mechanisms behind the TS and LS ISMA

An additional experiment that could validate the LS ISMA would involve disrupting other regulators of luminal tension. Depletion of key regulators of apical tension such as Cdc42 and its partner Protein kinase C zeta/lambda, using RNA interference should decrease lumen size.

Increasing Cdc42 activity, either by overexpression of WT Cdc42 or a constitutively active form (Cdc42-G12V) would be predicted to increase lumen size. In addition, depletion of RhoA and its effectors ROCK I and ROCK II which regulate myosin contractility should also decrease lumen size, since there will be insufficient luminal tension to support lumen expansion. In addition to potentially validating the molecular mechanisms, if disrupting Cdc42 causes cysts to develop multiple lumens and these cysts with smaller lumens fail to stabilize, it will indicate that stabilization is not caused by a timing-dependent mechanism, and is instead caused by a lumen or geometry dependent mechanism.

An ectopic increase in SIRT1 by stable overexpression or knockdown in LKB1 by RNA interference should prevent cysts from stabilizing and promote continued cyst growth. Thus, if cysts grown with cells with overexpression of SIRT1 or LKB1-KD displayed higher cell number at later time points, it would suggest that the molecular mechanism of cell stabilization was linked to SIRT1 and LKB1.

References

1. Campisi J, d'Adda di Fagagna F. (2007) Cellular senescence: When bad things happen to good cells. Nat Rev Mol Cell Biol 8: 729-740.
2. Wang Y, Liang Y, Vanhoutte PM. (2010) SIRT1 and AMPK in regulating mammalian senescence: A critical review and a working model. FEBS Lett. Dec 2. [Epub ahead of print]
3. Narala SR, Allsopp RC, Wells TB, Zhang G, Prasad P, et al. (2008) SIRT1 acts as a nutrient-sensitive growth suppressor and its loss is associated with increased AMPK and telomerase activity. Mol Biol Cell 19: 1210-1219.
4. Zu Y, Liu L, Lee MY, Xu C, Liang Y, et al. (2010) SIRT1 promotes proliferation and prevents senescence through targeting LKB1 in primary porcine aortic endothelial cells. Circ Res 106: 1384-1393.
5. Etienne-Manneville S. (2004) Cdc42--the centre of polarity. J Cell Sci 117: 1291-1300.
6. Martin-Belmonte F, Gassama A, Datta A, Yu W, Rescher U, et al. (2007) PTEN-mediated apical segregation of phosphoinositides controls epithelial morphogenesis through Cdc42. Cell 128: 383-397.
7. Bryant DM, Datta A, Rodriguez-Fraticelli AE, Peranen J, Martin-Belmonte F, et al. (2010) A molecular network for de novo generation of the apical surface and lumen. Nat Cell Biol 12: 1035-1045.
8. Rohatgi R, Ma L, Miki H, Lopez M, Kirchhausen T, et al. (1999) The interaction between N-WASP and the Arp2/3 complex links Cdc42-dependent signals to actin assembly. Cell 97: 221-231.
9. Zhang H, Macara IG. (2008) The PAR-6 polarity protein regulates dendritic spine morphogenesis through p190 RhoGAP and the rho GTPase. Dev Cell 14: 216-226.
10. Warner SJ, Longmore GD. (2009) Cdc42 antagonizes Rho1 activity at adherens junctions to limit epithelial cell apical tension. J Cell Biol 187: 119-133.
